# An anti-PMEL antibody−drug conjugate with a G_q/1__1_ inhibitor payload in *GNAQ*/*GNA11*-mutant melanomas: a phase 1 trial

**DOI:** 10.1038/s41591-026-04518-z

**Published:** 2026-07-13

**Authors:** Matteo S. Carlino, Ellen Kapiteijn, Sophie Piperno-Neumann, Ryan J. Sullivan, Richard Carvajal, Reinhard Dummer, Tanja Gromke, Jessica C. Hassel, Damien Kee, Egle Ramelyte, Alexander N. Shoushtari, Alexander Z. Wei, Alexander Enk, Eleanor E. Handel, Kamaneh Montazeri, Jorge Ramon-Patino, Heike Richly, Manuel Rodrigues, Shahneen Sandhu, James Smithy, Frank M. Speetjens, Pedro Milanez-Almeida, Anwesha Chaudhury, Keith Hoffmaster, Mary Ising, Javier A. Otero, Thiruvamoor RamKumar, Aimee Reynolds, Sherif Sharaby, Lillian Werner, Padmaja Yerramilli-Rao, Emiliano Calvo

**Affiliations:** 1 Westmead and Blacktown Hospitals, University of Sydney, Sydney, New South Wales Australia; 2 Melanoma Institute Australia, Sydney, New South Wales Australia; 3 Leiden University Medical Center, Leiden, The Netherlands; 4 Institut Curie, Paris, PSL Research University, Paris, France; 5 Massachusetts General Hospital, Boston, MA USA; 6 Northwell Health Cancer Institute, Lake Success, NY USA; 7 University Hospital Zurich, Zurich, Switzerland; 8 University Hospital Essen, Essen, Germany; 9 Heidelberg University, Medical Faculty Heidelberg, Department of Dermatology and National Center for Tumor Diseases (NCT), NCT Heidelberg, a partnership between DKFZ and University Hospital Heidelberg, Heidelberg, Germany; 10 Peter MacCallum Cancer Centre, University of Melbourne, Melbourne, Victoria Australia; 11 Memorial Sloan Kettering Cancer Center, New York, NY USA; 12 Weill Cornell Medical College, New York, NY USA; 13 Columbia University Herbert Irving Comprehensive Cancer Center, New York-Presbyterian Hospital, New York, NY USA; 14 START Madrid-CIOCC, Centro Integral Oncologico Clara Campal, Madrid, Spain; 15 Biomedical Research, Novartis, Cambridge, MA USA; 16 Development, Novartis, Cambridge, MA USA; 17 Biomedical Research, Novartis, East Hanover, NJ USA

**Keywords:** Eye cancer, Targeted therapies

## Abstract

Metastatic uveal melanoma (mUM) is an aggressive cancer with limited treatment options; 85−90% of tumors harbor activating *GNAQ* and *GNA11* mutations. Uveal melanoma cells also express PMEL (also known as PMEL17 or gp100), a melanocyte lineage antigen. DYP688, a novel biology-matched antibody−drug conjugate, binds surface PMEL and delivers the potent Gα_q_/Gα_11_ (G_q/11_) inhibitor SDZ475 as payload by internalization. This dose-escalating first-in-human phase 1 study of DYP688 in patients with metastatic uveal melanoma and other *GNAQ*/*GNA11*-mutant melanomas assessed safety as the primary endpoint and pharmacokinetics and preliminary antitumor activity as secondary endpoints. Sixty-six patients received varying DYP688 doses and schedules. Grade 3 treatment-related adverse events occurred in five patients (7.6%), including one dose-limiting toxicity of grade 3 hypotension. Objective responses were seen in 13 out of 66 patients (19.7%) and tumor reduction in 47 out of 66 patients (71.2%). Median progression-free survival was 7.2 (95% CI: 5.3–7.8) months. In summary, DYP688 was well tolerated and showed preliminary efficacy, supporting this novel therapeutic approach. ClinicalTrials.gov identifier: NCT05415072.

## Abstract (web summary)

In a phase 1 trial, treatment with the antibody−drug conjugate DYP688, targeting PMEL and delivering a G_q/__11_ inhibitor, in patients with *GNAQ*/*GNA11*-mutant metastatic uveal melanoma or other *GNAQ*/*GNA11*-mutant melanomas, showed acceptable safety and encouraging preliminary efficacy, with supporting biomarker data for proof of mechanism.

## Main

Uveal melanoma (UM) is the most common primary intraocular malignancy in adults, accounting for 3−5% of all melanomas^1^. It originates from extracutaneous melanocytes residing within the uveal tract of the eye, with 85−90% of cases arising in the choroid and 9−15% in the iris or ciliary body^2,3^. The annual incidence of the disease varies globally, with higher rates reported in non-Hispanic white individuals and in regions such as northern Europe and Australia^4–8^.

Despite effective clinical management of primary uveal melanoma, including surgery and radiotherapy, 25−34% of patients will develop distant metastases in 5−10 years^9^. However, modern risk‑adapted surveillance strategies that incorporate high‑sensitivity cross‑sectional imaging detect metastatic recurrences earlier and at smaller volumes than older approaches, suggesting that earlier estimates may have under-detected metastatic disease. Consequently, the true lifetime risk is likely closer to up to 50%, depending on tumor genetics and other prognostic features^10,11^. Available regional and systemic therapies have limited efficacy, and the approximate median overall survival after detection of metastasis historically has been 1 year^12,13^.

Approximately 85−90% of uveal melanomas harbor mutually exclusive activating mutations in either *GNAQ* or *GNA11*, most commonly affecting conserved hotspot residues Gln209 and, less frequently, Arg183. These mutations result in constitutive activation of the GTPase domain, driving oncogenic signaling early in tumor development. These residues are shared across both proteins and may be affected in either gene^14,15^. Although these mutations are hallmark features of uveal melanoma, they occasionally are observed in mucosal and cutaneous melanomas. Direct inhibition of mutant Gα_q_/Gα_11_ (G_q/11_) proteins is hypothesized to offer superior therapeutic efficacy compared to targeting downstream effectors such as protein kinase C (PKC) or mitogen-activated protein kinase kinase (MEK)^15,16^.

However, despite promising preclinical activity, systemic G_q/11_ inhibition has been limited by on-target, off-tumor toxicities, owing to the widespread physiological roles of these G proteins^17,18^. In xenograft models, direct administration of the specific G_q__/__11_ inhibitor SDZ475 (also known as FR900359) caused platelet dysfunction, bleeding and hypotension^17^.

To overcome the limitations of systemic G_q/1__1_ inhibition, we explored tumor-specific payload delivery targeting PMEL (also known as PMEL17 or gp100), a melanocyte lineage-specific protein that is highly expressed in both cutaneous and uveal melanomas as well as in normal melanocytes of the retina, skin and substantia nigra^19,20^. PMEL is expressed mainly in the melanosome, an organelle within melanocytes involved in the synthesis and storage of melanin, and is also found on the cell surface^21^.

The clinical relevance of PMEL as a therapeutic target has been validated by the success of tebentafusp, a bispecific PMEL× CD3 T cell engager, which has demonstrated a significant overall survival benefit in human leukocyte antigen (HLA)-A*02:01-positive patients with metastatic uveal melanoma (mUM) and has received regulatory approval for this indication^22,23^. However, its mechanism of action relies on the presence of HLA allele to present the PMEL peptide to T cells, and so its use is limited to HLA-A*02:01-positive patients, excluding over half of the mUM population^22^ who currently have no approved systemic therapies for metastatic disease^22–25^.

The efficacy of tebentafusup highlights the potential of PMEL targeting to selectively engage tumor cells while sparing normal tissues, paving the way for novel approaches such as antibody−drug conjugates (ADCs) that deliver cytotoxic agents directly to PMEL-expressing tumor cells.

**Extended Data Fig. 1:**
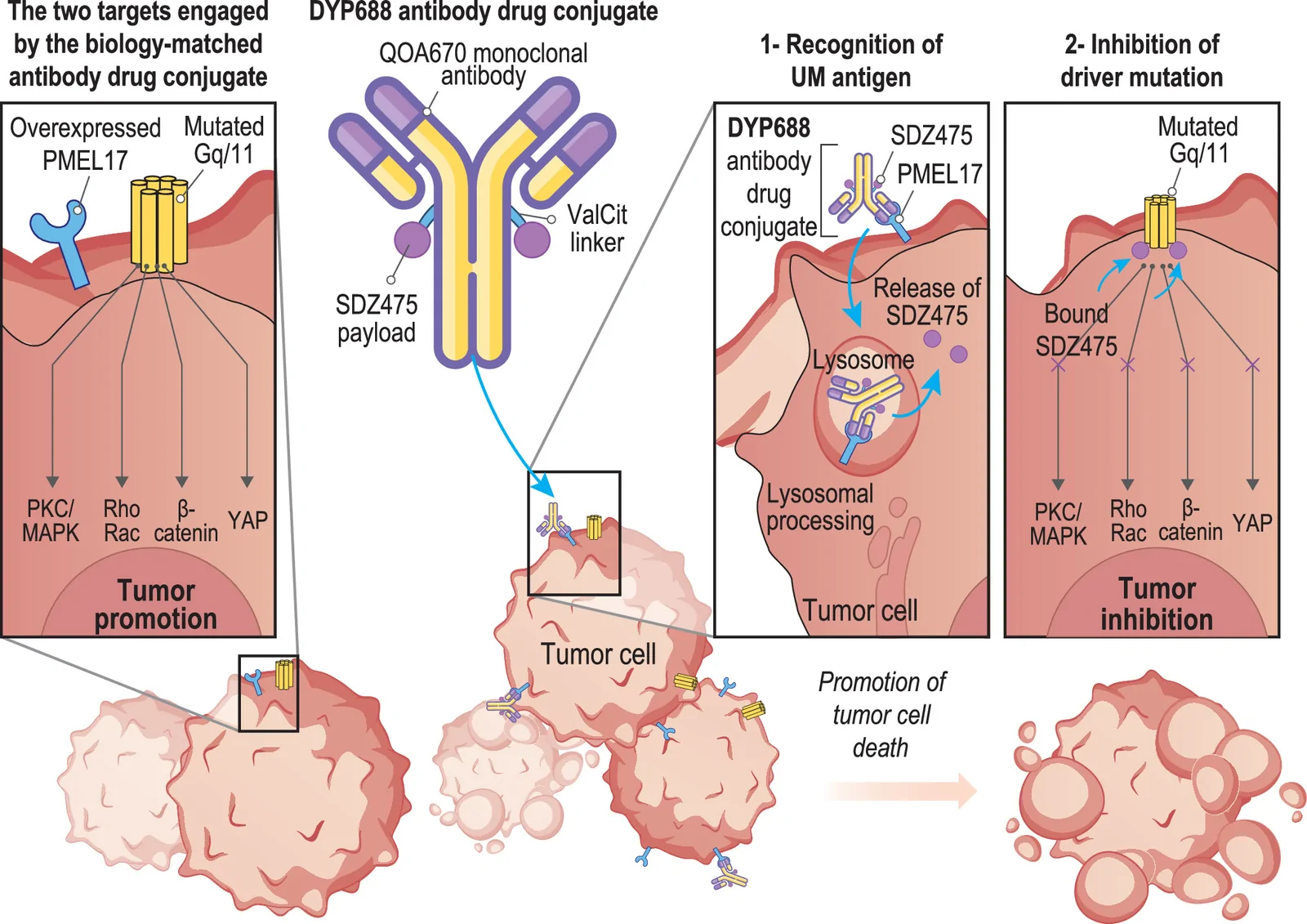
Mechanism of action of DYP688, a PMEL17-targeting antibody–drug conjugate. The biology matched- antibody–drug conjugate DYP688 engages two targets in uveal melanoma: overexpressed PMEL17 and mutant Gq-/11. SDZ475 is active against both mutant and wildtype Gαq/11 proteins; however, its antitumor effects are observed- selectively in tumor cells that are dependent on Gαq/11 signaling for proliferation (that is, those harboring driver mutations), while normal wildtype GNAQ/11 cells are largely unaffected. (Left) Tumor cells overexpress PMEL17 and harbor activating Gq/11 mutations, which drive oncogenic signaling through PKC/MAPK, Rho/Rac, β-catenin, and YAP pathways, promoting tumor growth. (Center) DYP688 comprises the QOA670 monoclonal antibody conjugated via a ValCit linker to the SDZ475 payload. Upon binding PMEL17 on tumor cells, the antibody–drug conjugate is internalized and undergoes lysosomal processing, releasing SDZ475. (Right) Released SDZ475 binds Gq/11 and inhibits downstream oncogenic signaling, resulting in tumor growth suppression and promotion of tumor cell death. β-catenin, beta-catenin; MAPK, mitogen-activated protein kinase; PKC, protein kinase C; PMEL17, pre-melanosome protein 17; Rac, rac GTPase; Rho, rho GTPase; UM, uveal melanoma; YAP, Yes-associated protein.

DYP688 is a first-in-class PMEL-targeting ADC composed of a highly selective humanized cysteine engineered PMEL monoclonal antibody, conjugated at a drug−antibody ratio (DAR) of 2 via a maleimide-based stable VaLCit linker^18^ to the G_q/11_ inhibitory payload SDZ475 (ref. ^17^). DYP688 is an ADC with a biology-matched payload that is first ‘localized to’ PMEL^19,20^. Upon ADC binding to PMEL on the target cells and internalization, the linker is cleaved to release the G_q__/__11_ inhibitor SDZ475, which inhibits *GNAQ/GNA11*-mediated oncogenic signaling, resulting in dose-dependent apoptosis^18^. The mechanism of action of DYP688 is illustrated in Extended Data Fig. 1. DYP688 showed dose-dependent antitumor activity across a panel of mUM xenografts, including mouse models expressing various levels of PMEL and patient-derived xenografts of mUM. Preclinical toxicology studies showed that DYP688 was well tolerated with minimal and reversible clinical and histopathological findings, supporting the notion that DYP688 can specifically target *GNAQ/GNA11*-mutant melanocytic tumors with a favorable safety profile in humans^18^.

Here we report the safety, tolerability, pharmacokinetics, pharmacodynamics and preliminary antitumor activity data from the completed phase 1 dose-escalation part of the first-in-human, open-label, multicenter study of DYP688 as a single agent in patients with mUM and other *GNAQ*/*GNA11*-mutant melanomas (NCT05415072)^26^.

## Results

### Patient characteristics

**Fig. 1:**
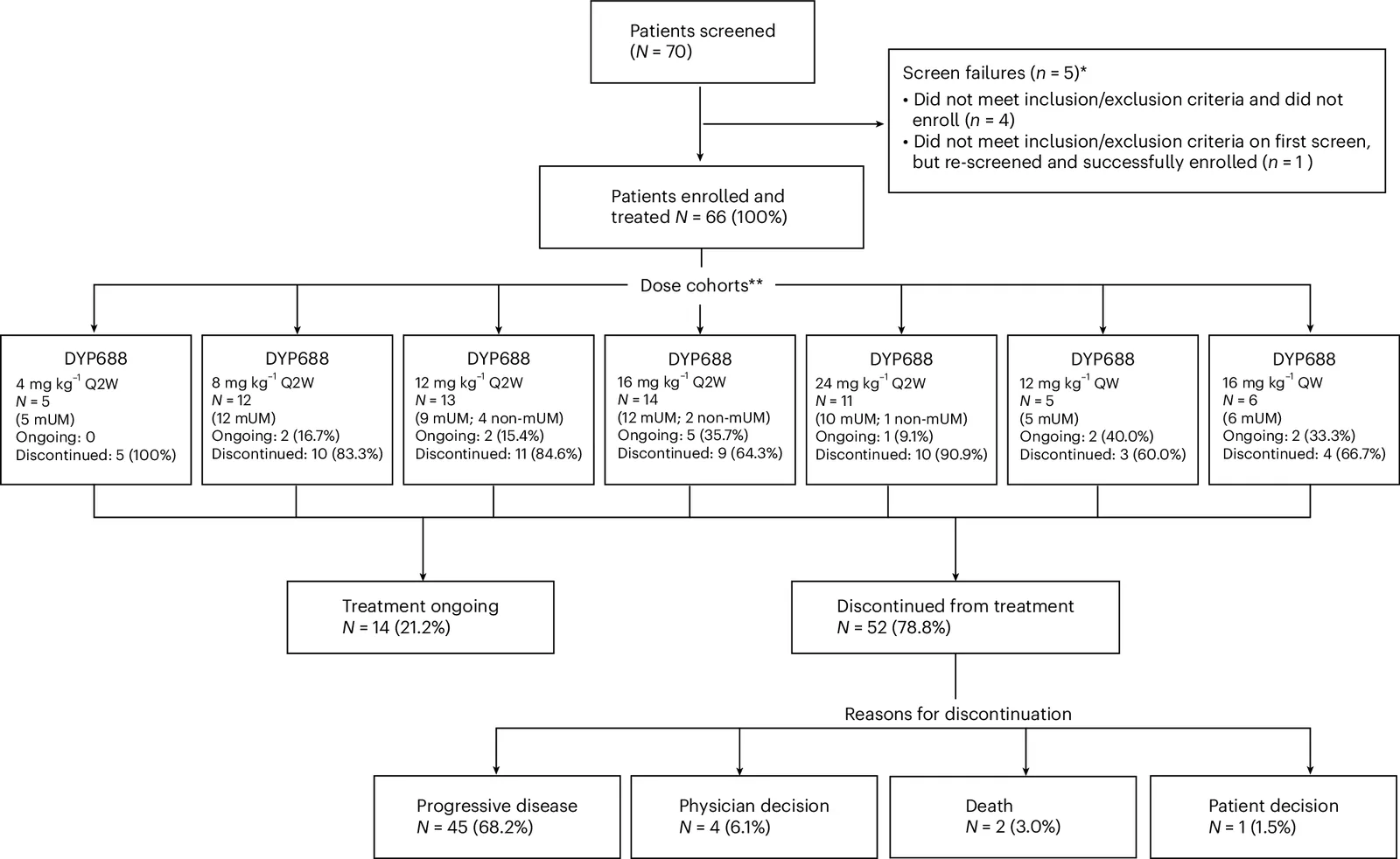
Patient disposition and treatment status. Flow diagram showing the number of patients enrolled and treated with DYP688 across various dose levels and schedules (*n* = 66). *One additional patient was allowed to enter treatment. **Patients were permitted to undergo intrapatient dose escalation to a higher dose deemed safe after four cycles of treatment; cohort assignment reflects the initial dose.

A total of 66 patients were treated with DYP688 in the dose-escalation part of this study, which began enrolling patients on 4 July 2022, with doses ranging from 4 mg kg^−1^ to 24 mg kg^−1^ every 2 weeks (Q2W; *n* = 55) and 12 mg kg^−1^ to 16 mg kg^−1^ once weekly (QW; *n* = 11). Enrollment was halted on 8 July 2025 after completion of the phase 1 portion of the study. Patient disposition is shown in Fig. 1 and Supplementary Table 1. At the data cutoff date (1 May 2025), treatment was ongoing in 14 patients (21.2%), and 52 patients (78.8%) had discontinued treatment, most (*n* = 45; 68.2%) due to disease progression, four (6.1%) due to physician decision, two (3.0%) due to death (due to disease progression) and one (1.5%) due to patient decision. No patients discontinued due to treatment-related adverse events (TRAEs). Deviations from the protocol were reviewed on an ongoing basis by the sponsor and the principal investigators. Protocol deviations are not uncommon in early-phase clinical trials, given their exploratory nature and the need for real-time clinical decision-making. These deviations did not impact the overall scientific validity or interpretation of the study. A summary of all protocol deviations is provided in Supplementary Table 2.

Demographic and baseline characteristics are summarized in Table 1. The median age was 56.5 years (range, 25−82). In total, 60 patients (90.9%) had mUM. *GNAQ* or *GNA11* mutant melanomas arising outside the uveal tract included cutaneous primary (*n* = 4; 6.1%), central nervous system (*n* = 1; 1.5%) and unknown primary site (*n* = 1; 1.5%). Most patients (*n* = 61, 92.4%) had received prior antineoplastic therapy; most of them were heavily pretreated, with 39 patients (59.1%) having received at least two prior lines of systemic therapy. In total, 48 patients (72.7%) had received prior checkpoint inhibitors, 22 (33.3%) had received prior tebentafusp, 9 (13.6%) had received prior darovasertib and 5 (7.6%) had received prior darovasertib and crizotinib (Supplementary Table 3). Baseline American Joint Committee on Cancer (AJCC) metastatic categories for patients with uveal melanoma by dose cohort are summarized in Extended Data Table 1. Overall, four patients (6.1%) were reported to have responded to any prior therapy. Most patients (*n* = 30, 40.5%) had progressive disease as the best overall response (BOR) to their last prior therapy, and, in these patients, the median time from last treatment to progression had been 2.76 months (range, 0.0−19.3). At study entry, most patients had liver metastases (*n* = 57, 86.4%); 43 patients (65.2%) had lactate dehydrogenase (LDH) levels above the upper limit of normal (ULN); and 16 patients (24.2%) had LDH levels >2× ULN.

**Table 1:** Demographic and other baseline characteristics

Baseline characteristics	4 mg kg^−1^ Q2W *N* = 5	8 mg kg^−1^ Q2W *N* = 12	12 mg kg^−1^ Q2W *N* = 13	16 mg kg^−1^ Q2W *N* = 14	24 mg kg^−1^ Q2W *N* = 11	12 mg kg^−1^ QW *N* = 5	16 mg kg^−1^ QW *N* = 6	All patients *N* = 66
Age (years), median (range)	68.0 (63−78)	55.5 (35−74)	39.0 (25−70)	57.5 (33−82)	52.0 (35−69)	56.0 (50−80)	60.0 (37−67)	56.5 (25−82)
Sex, *n* (%)								
Male	2 (40.0)	5 (41.7)	7 (53.8)	8 (57.1)	5 (45.5)	1 (20.0)	4 (66.7)	32 (48.5)
Female	3 (60.0)	7 (58.3)	6 (46.2)	6 (42.9)	6 (54.5)	4 (80.0)	2 (33.3)	34 (51.5)
ECOG PS, *n* (%)								
0	2 (40.0)	9 (75.0)	11 (84.6)	11 (78.6)	8 (72.7)	4 (80.0)	5 (83.3)	50 (75.8)
1	3 (60.0)	3 (25.0)	2 (15.4)	3 (21.4)	3 (27.3)	1 (20.0)	1 (16.7)	16 (24.2)
LDH, *n* (%)								
>2× ULN	2 (40.0)	2 (16.7)	1 (7.7)	4 (28.6)	5 (45.5)	1 (20.0)	1 (16.7)	16 (24.2)
>ULN	5 (100)	7 (58.3)	7 (53.8)	10 (71.4)	9 (81.8)	3 (60.0)	2 (33.3)	43 (65.2)
≤ULN	0	5 (41.7)	6 (46.2)	4 (28.6)	2 (18.2)	2 (40.0)	4 (66.7)	23 (34.8)
Diagnosis of disease, *n* (%)								
UM	5 (100)	12 (100)	9 (69.2)	13 (92.9)	10 (90.9)	5 (100)	6 (100)	60 (90.9)
Cutaneous melanoma	0	0	2 (15.4)	1 (7.1)	1 (9.1)	0	0	4 (6.1)
Other/unknown	0	0	2 (15.4)	0	0	0	0	2 (3.0)
Maximum target lesion diameter (mm), median (range)	62.0 (28−88)	48.5 (11−91)	30.5 (11−121)	47.5 (20−116)	51.0 (22−115)	26.0 (21−102)	55.5 (20−100)	50.0 (11−121)
Current extent of the disease (metastatic sites), *n* (%)								
Liver	5 (100)	12 (100)	9 (69.2)	10 (71.4)	11 (100)	5 (100)	5 (83.3)	57 (86.4)
Lung	4 (80.0)	6 (50.0)	4 (30.8)	7 (50.0)	3 (27.3)	2 (40.0)	3 (50.0)	29 (43.9)
Other	4 (80.0)	9 (75.0)	7 (53.8)	12 (85.7)	9 (81.8)	2 (40.0)	4 (66.7)	47 (71.2)
Skin	1 (20.0)	1 (8.3)	0	2 (14.3)	5 (45.5)	0	1 (16.7)	10 (15.2)
Number of patients with prior antineoplastic regimens, *n* (%)								
Yes	5 (100)	12 (100)	12 (92.3)	13 (92.9)	11 (100)	3 (60.0)	5 (83.3)	61 (92.4)
Number of prior antineoplastic regimens, *n* (%)								
≥2	4 (80.0)	7 (58.3)	8 (61.5)	7 (50.0)	7 (63.6)	2 (40.0)	4 (66.7)	39 (59.1)
1	1 (20.0)	5 (41.7)	4 (30.8)	6 (42.9)	4 (36.4)	1 (20.0)	1 (16.7)	22 (33.3)
0	0	0	1 (7.7)	1 (7.1)	0	2 (40.0)	1 (16.7)	5 (7.6)
Responded to any prior therapy, *n* (%)	0	1 (8.3)	0	1 (7.1)	2 (18.2)	0	0	4 (6.1)
Best response at last treatment, *n* (%)								
Complete response	0	1 (8.3)	0	0	0	0	0	1 (1.5)
Partial response	0	0	0	1 (7.1)	2 (18.2)	0	0	3 (4.5)
Stable disease	0	2 (16.7)	2 (15.4)	3 (21.4)	2 (18.2)	3 (60.0)	1 (16.7)	13 (19.7)
Progressive disease	3 (60.0)	4 (33.3)	9 (69.2)	6 (42.9)	5 (45.5)	0	3 (50.0)	30 (45.5)
Unknown	2 (40.0)	5 (41.7)	1 (7.7)	3 (21.4)	2 (18.2)	0	1 (16.7)	14 (21.2)
Time from the last treatment to progression (months), median (range)	2.07 (0.6−3.8)	1.54 (0.8−14.5)	3.02 (0.0−12.1)	2.76 (0.8−11.3)	2.27 (0.6−18.7)	4.53 (4.3−19.3)	5.26 (1.5−16.4)	2.76 (0.0−19.3)

### Safety

Of the 66 patients evaluable for safety and maximum tolerated dose (MTD)/recommended dose determination, only one experienced a dose-limiting toxicity (DLT). This patient received DYP688 at 24 mg kg^−1^ Q2W and experienced grade 3 hypotension 2 days after the first infusion. The patient was treated with supportive care, and the event resolved within 24 h. The DYP688 dose was reduced to 16 mg kg^−1^ Q2W for subsequent infusions without further hypotensive episodes.

Sixty-five patients (98.5%) experienced at least one treatment-emergent adverse event (TEAE). The TEAEs are summarized in Table 2.

**Table 2:** Overview of TEAEs

	4 mg kg^−1^ Q2W *N* = 5		8 mg kg^−1^ Q2W *N* = 12		12 mg kg^−1^ Q2W *N* = 13		16 mg kg^−1^ Q2W *N* = 14		24 mg kg^−1^ Q2W *N* = 11		12 mg kg^−1^ QW *N* = 5		16 mg kg^−1^ QW *N* = 6		All patients *N* = 66	
	All grades	Grade ≥ 3	All grades	Grade ≥ 3	All grades	Grade ≥ 3	All grades	Grade ≥ 3	All grades	Grade ≥ 3	All grades	Grade ≥ 3	All grades	Grade ≥ 3	All grades	Grade ≥ 3
Category	*n* (%)	*n* (%)	*n* (%)	*n* (%)	*n* (%)	*n* (%)	*n* (%)	*n* (%)	*n* (%)	*n* (%)	*n* (%)	*n* (%)	*n* (%)	*n* (%)	*n* (%)	*n* (%)
TEAEs	5 (100)	1 (20.0)	12 (100)	5 (41.7)	12 (92.3)	3 (23.1)	14 (100)	7 (50.0)	11 (100)	5 (45.5)	5 (100)	2 (40.0)	6 (100)	3 (50.0)	65 (98.5)	26 (39.4)
TRAEs	4 (80.0)	0	10 (83.3)	0	11 (84.6)	0	14 (100)	0	11 (100)	2 (18.2)	5 (100)	1 (20.0)	5 (83.3)	2 (33.3)	60 (90.9)	5 (7.6)
DLTs	0	0	0	0	0	0	0	0	1 (9.1)	1 (9.1)	0	0	0	0	1 (1.5)	1 (1.5)
SAEs	0	0	5 (41.7)	5 (41.7)	4 (30.8)	2 (15.4)	7 (50.0)	6 (42.9)	6 (54.5)	4 (36.4)	2 (40.0)	2 (40.0)	2 (33.3)	2 (33.3)	26 (39.4)	21 (31.8)
Treatment related	0	0	0	0	2 (15.4)	0	0	0	2 (18.2)	1 (9.1)	0	0	0	0	4 (6.1)	1 (1.5)
TRAEs leading to study discontinuation	0	0	0	0	0	0	0	0	0	0	0	0	0	0	0	0
AEs leading to dose adjustment/interruption	0	0	3 (25.0)	0	2 (15.4)	2 (15.4)	3 (21.4)	0	3 (27.3)	1 (9.1)	3 (60.0)	1 (20.0)	3 (50.0)	2 (33.3)	17 (25.8)	6 (9.1)

Numbers (*n*) represent counts of patients; a patient with multiple severity grades for an adverse effect (AE) is only counted under the maximum grade. Medical Dictionary for Regulatory Activities (MedDRA) version 27.1, CTCAE version 5.0.

The most frequently reported TEAEs (occurring in ≥20% of patients) were fatigue (*n* = 30, 45.5%); asymptomatic hypercalcemia (*n* = 24, 36.4%); dry mouth and peripheral edema (*n* = 20 each, 30.3%); constipation (*n* = 17, 25.8%); abdominal pain, anemia and nausea (*n* = 16 each, 24.2%); and dyspnea (*n* = 14, 21.1%).

TRAEs of any grade were reported in 60 patients (90.9%). All TRAEs were of grade 2 or lower severity, except for five grade 3 events: hypotension, asymptomatic hypercalcemia, anemia, increased gamma-glutamyl transferase (GGT) and decreased lymphocyte count. The most common TRAEs (all grades, all doses) were hypercalcemia (*n* = 19, 28.8%); dry mouth and fatigue (*n* = 15 each, 22.7%); peripheral edema (*n* = 12, 18.2%); anemia (*n* = 10, 15.2%); increased aspartate aminotransferase and constipation (*n* = 8 each, 12.1%); increased alanine aminotransferase, epistaxis and nasal congestion (*n* = 7 each, 10.6%); and asthenia, hypophosphatemia, nausea and thrombocytopenia (*n* = 6 each, 9.1%). Dermatological TRAEs were infrequent and nonserious, with all events reported as grade 2 or lower. Vitiligo was observed in three patients (4.5%), all grade 1. TRAEs of any grade reported in at least 5% of patients in treatment groups 12, 16 and 24 mg kg^−1^ Q2W, in at least 5% of patients in treatment groups 12 and 16 mg kg^−1^ QW and in all patients are shown in Supplementary Fig. 1.

Treatment-emergent serious adverse events (SAEs) were reported in 26 patients (39.4%). Of these, grade 3 or higher events were reported in 21 patients (31.8%). Treatment-related SAEs were reported in four patients (6.1%). Bradycardia (grade 1), sinus tachycardia (grade 2), infusion-related reaction (grade 2) and hypotension (grade 3, DLT) were reported in one patient each (1.5%). No treatment-related SAEs higher than grade 3 were reported in the study.

No patients discontinued the study treatment owing to adverse events. TRAEs that led to dose reduction were reported in three patients (grade 3 hypotension, grade 2 anemia and grade 1 hypercalcemia), and TRAEs that led to interruption of study drug were reported in eight patients (12.1%) (Table 2).

### Pharmacokinetics

Pharmacokinetics data were available for 66 patients, who received DYP688 at doses of 4, 8, 12, 16 or 24 mg kg^−1^ intravenous infusion Q2W or 12 or 16 mg kg^−1^ intravenous infusion QW. A dose-dependent increase in pharmacokinetics exposure (area under the concentration–time curve (AUC)) was observed across total antibody, conjugated active payload, conjugated inactive phosphorylated payload and free payload. Exposure to free payload in blood was markedly lower compared to conjugated active payload in plasma, with approximately 80-fold lower AUC_0−336 h_ at DYP688 24 mg kg^−1^ intravenous infusion Q2W at steady state.

**Fig. 2:**
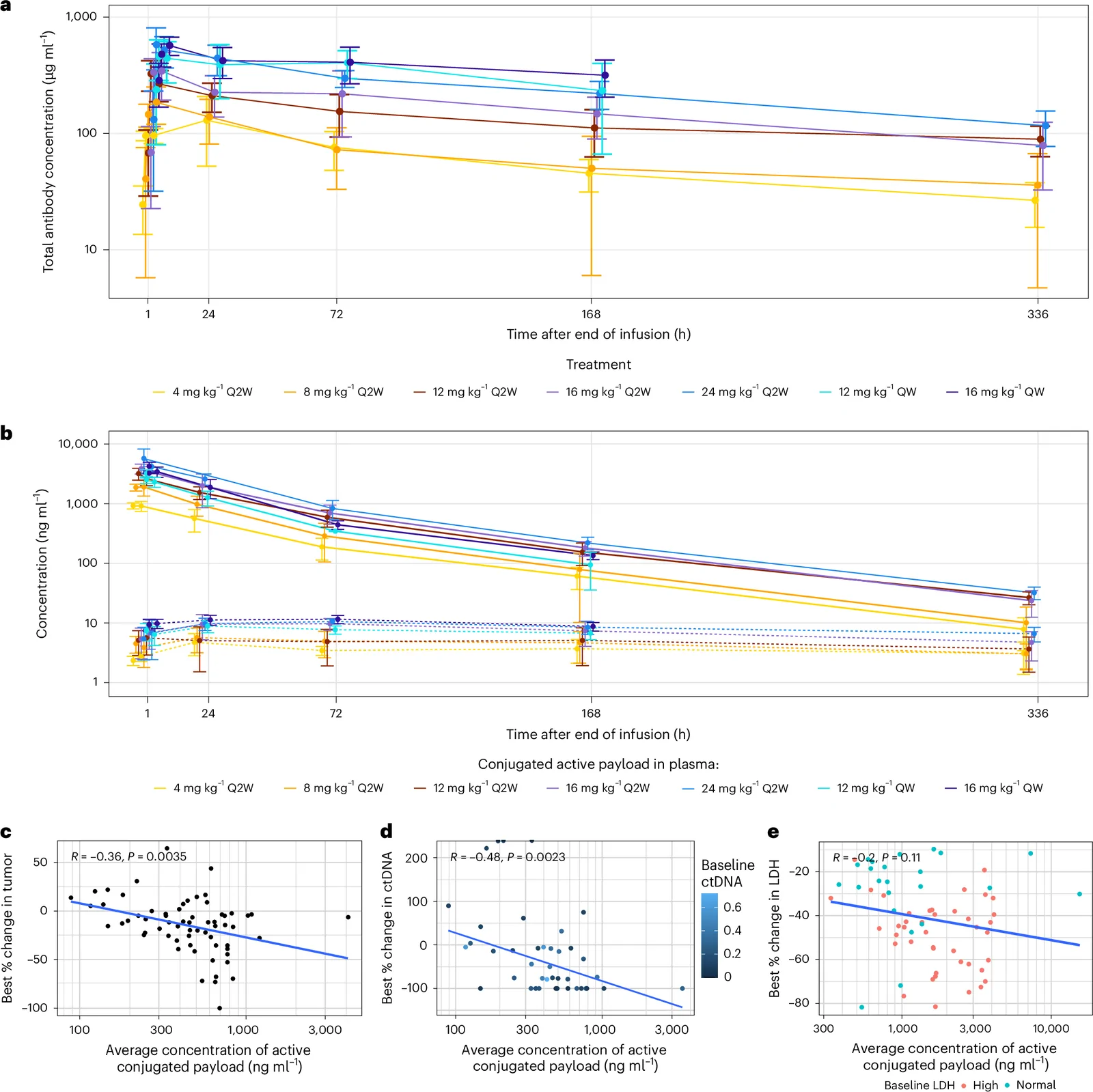
Pharmacokinetics and exposure−response of DYP688. **a**, Mean (s.d.) serum concentration−time profiles of DYP688 total antibody on C3D1 (*n* = 66). **b**, Mean (s.d.) concentration–time profiles of DYP688-conjugated active payload (plasma) and free payload (blood) on C3D1 (*n* = 66). **c**, Best percentage change in tumor diameters by active conjugated payload concentration. **d**, Best percentage change in ctDNA by active conjugated payload concentration. **e**, Best percentage change in LDH levels by active conjugated payload concentration. **c**–**e**, *P* values are from two-sided correlation tests.

The geometric mean effective half-life of the total antibody (serum) and free payload (blood) was 8.6 days and 12 days, respectively. The geometric mean apparent terminal half-life of the conjugated active payload (plasma) was approximately 2 days. Mean concentration−time profiles of the total antibody, active conjugated payload and free payload are shown in Fig. 2a,b.

An apparent relationship was observed between average concentration (*C*_avg_) of the active conjugated payload and tumor size change, with increased tumor shrinkage seen with higher exposure in patients (Fig. 2c). A similar trend was observed between exposure (*C*_avg_ of the active conjugated payload) and circulating tumor DNA (ctDNA) reduction with absence of detectable ctDNA in several patients, on-treatment with higher exposure (Fig. 2d). Higher exposure was also associated with greater reduction in blood LDH levels in patients (Fig. 2e), although the trend is relatively weak. The *C*_avg_ was calculated using a population pharmacokinetics model for the active conjugated payload and simulating the pharmacokinetics profiles up to the time at which the best change in tumor, ctDNA or LDH was observed. The model-based calculation of exposure accounts for changes in the patientʼs dosing history—for example, dose interruptions and intrapatient dose escalation.

Immunogenicity was assessed using a three-tiered approach comprising screening, confirmatory and titer assays. Based on available data as of the data cutoff date, DYP688 demonstrates low immunogenicity, with an ADA incidence of less than 10%.

### Efficacy

**Fig. 3:**
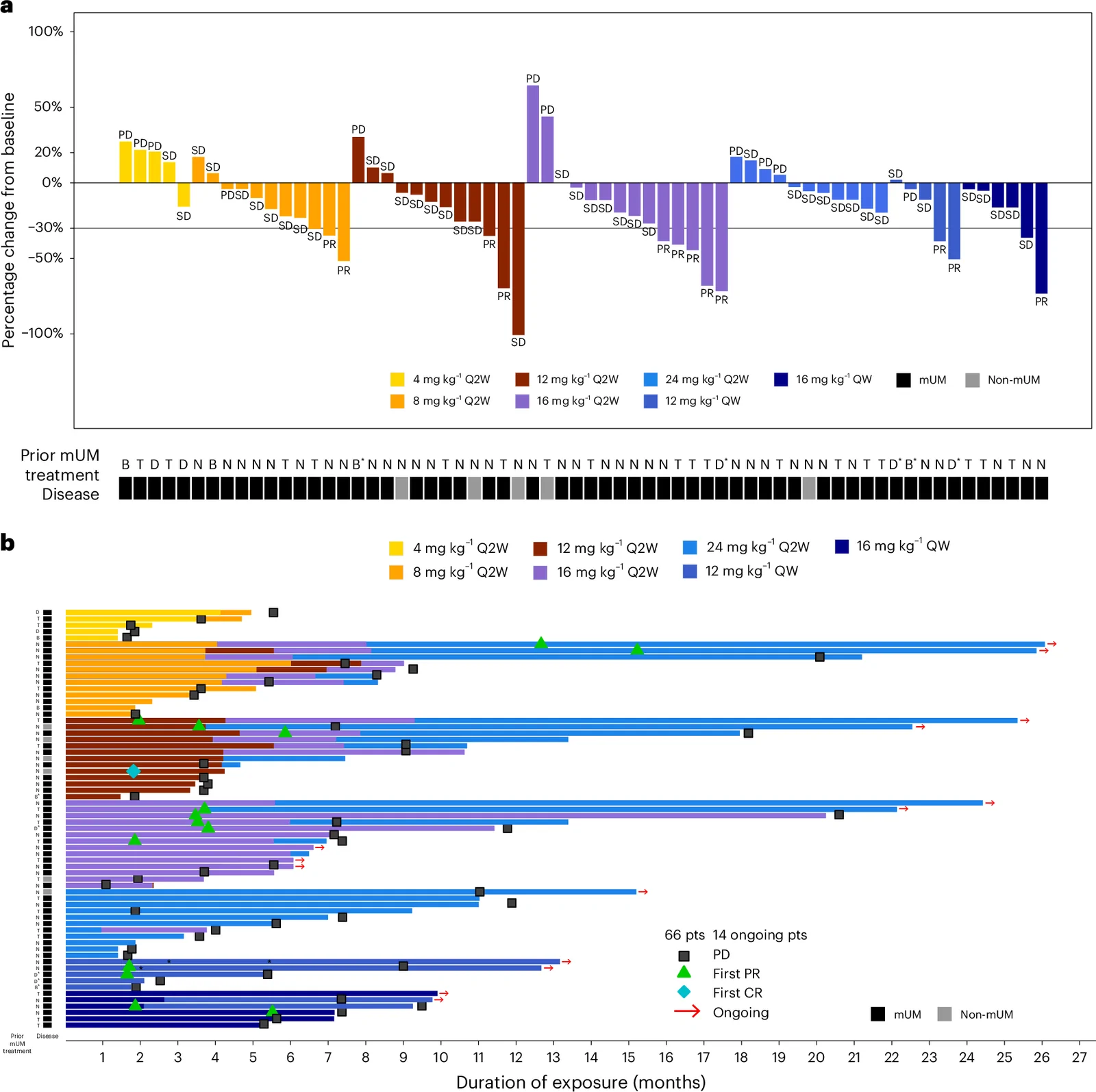
Tumor response and duration of exposure. **a**, Percentage change from baseline in sum of the longest diameters by radiology review. The percentage of tumor size reduction is illustrated on the *y* axis. Every bar is one patient. The horizontal line represents at least a 30% decrease in the sum of the diameters of target lesions in patients. One patient was non-evaluable at the only post-baseline scan as one of the target lesions could not be measured, and one patient had non-measurable disease at baseline. **b**, Duration of treatment. The *x* axis denotes time in months; each horizontal bar on the *y* axis represents a single patient. B, darovasertib and tebentafusp prior treatment; B*, darovasertib plus crizotinib and tebentafusp prior treatment; D, darovasertib prior treatment; D*, darovasertib plus crizotinib prior treatment; N, no prior darovasertib/crizotinib or tebentafusp treatment; PD, progressive disease; PR, partial response; pts, patients; SD, stable disease; T, tebentafusp prior treatment.

Of the 66 patients treated, 65 had measurable disease at baseline (Extended Data Table 2). The median duration of treatment was 7.2 months (range, 1.8−26.6). Confirmed objective responses were seen in 13 out of 66 patients (19.7%), with 2 out of 12 (16.7%) at 8 mg kg^−1^ Q2W, 3 out of 13 (23.1%) at 12 mg kg^−1^ Q2W, 5 out of 14 (35.7%) at 16 mg kg^−1^ Q2W, 2 out of 5 (40.0%) at 12 mg kg^−1^ QW and 1 out of 6 (16.7%) at 16 mg kg^−1^ QW, with evidence of deepening response over time in some patients. One patient with cutaneous melanoma with non-measurable disease per Response Evaluation Criteria in Solid Tumors (RECIST) version 1.1 at baseline (enrolled outside protocol-specified criteria) achieved a confirmed complete response at 12 mg kg^−1^ Q2W. Three patients achieved a partial response after intrapatient dose escalation: two patients who were escalated from 8 mg kg^−1^ Q2W to 24 mg kg^−1^ Q2W and another who was escalated from 12 mg kg^−1^ Q2W to 24 mg kg^−1^ Q2W. Progressive disease was seen in 11 out of 66 patients (16.7%), and stable disease was seen in 41 out of 66 patients (62.1%). Efficacy outcomes for patients with uveal melanoma are summarized in Supplementary Table 4. Median (95% confidence interval (CI)) duration of response by Kaplan−Meier analysis was 10.5 months (3.7−not evaluable); disease control rate (DCR) for the 66 patients was 81.8% (95% CI: 70.4−90.2). Best percentage change from baseline in sum of longest target lesion diameters based on local radiology review is shown in Fig. 3a. Tumor size reduction was observed in 47 out of 66 patients (71.2%). An analysis of the best percentage change in target lesion size by anatomical location showed tumor shrinkage across liver, lung, lymph node and other sites, with no evidence of preferential response by organ site (Supplementary Fig. 2). Fourteen patients (21.2%) remained on-treatment for at least 12 months (Fig. 3b).

In total, 14 patients were censored, and 52 patients (78.8%) had progression-free survival (PFS) events. Of the patients with PFS events, 49 (74.2%) had disease progression and 3 (4.5%) died. The estimated median PFS (95% CI) was 7.2 (5.3−7.8) months.

**Extended Data Fig. 2:**
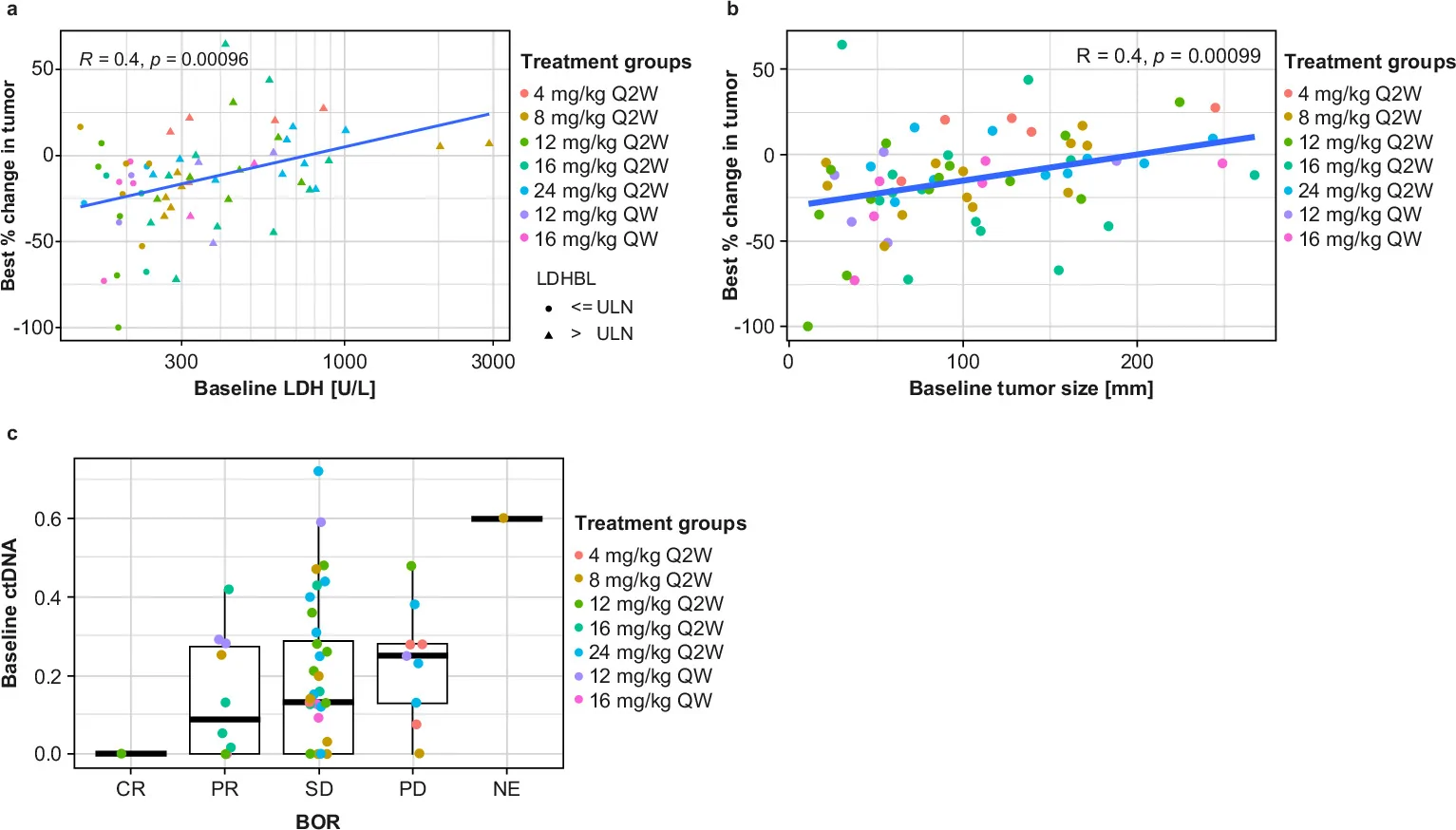
Baseline Predictors of Tumor Response. (**a-b**) Scatter plot illustrates (**a**) the relationship between baseline lactate LDH levels (x-axis) and the best percentage change in tumor size (y-axis) among patients enrolled in various treatment arms. Each data point represents an individual patient, color-coded and shaped according to treatment arm and LDH baseline category. blue trend line is fitted to the data, indicating a positive correlation between baseline LDH and tumor response. The correlation coefficient is R = 0.4, with a p-value = 0.00096, denoting statistical significance. (**b**) the relationship between baseline tumor size (in mm) and the best percentage change in tumor size across different treatment groups. A linear regression line (blue) is fitted to the data, showing a positive correlation with a coefficient R = 0.4 and a statistically significant p-value of 0.00099. (**c**) Box plot illustrating baseline ctDNA levels stratified by BOR. Each colored dot represents an individual patient, with colors corresponding to different treatment groups. BOR, best overall response; CR, complete response; ctDNA, circulating tumor DNA D, day; NE, not evaluable; PD, progressive disease; PR, partial response; SD, stable disease; LDH, lactate dehydrogenase; QW, once weekly; Q2W, every 2 weeks.; LDH, lactate dehydrogenase; QW, once weekly; Q2W, every 2 weeks.

**Extended Data Fig. 3:**
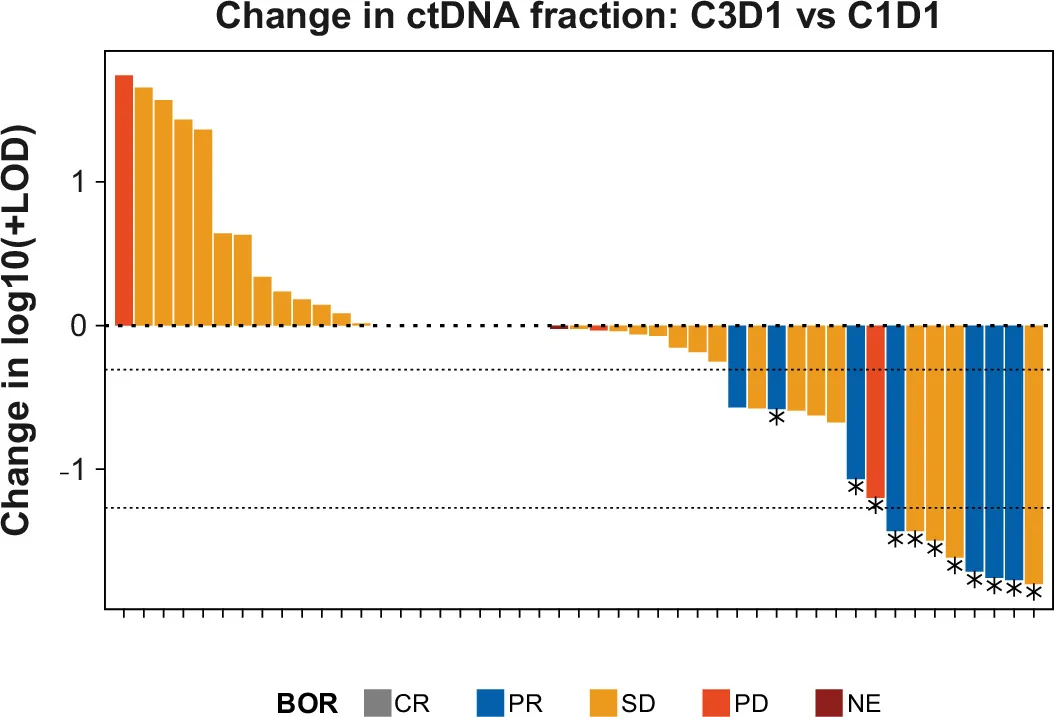
ctDNA Change from Baseline (C3D1 vs C1D1). Partial responders more frequently exhibit reductions in ctDNA fraction compared with patients with stable disease or disease progression. Change in circulating tumor DNA (ctDNA) fraction from baseline to Cycle 3 Day 1. Waterfall plot showing the change in log10‑transformed ctDNA fraction between Cycle 1 Day 1 (C1D1) and Cycle 3 Day 1 (C3D1) for individual patients with paired samples at both timepoints. ctDNA fractions were log10‑transformed after addition of the assay limit of detection (LOD; 0.005) to enable inclusion of low or undetectable values. Bars are ordered by magnitude of change and colored by best overall response (BOR). Negative values indicate reduction in ctDNA fraction relative to baseline. The dotted horizontal line at 0 indicates no change from baseline; additional dotted lines indicate approximate 50% and 95% reductions in ctDNA fraction. Asterisks denote patients with ctDNA clearance at C3D1, defined as ctDNA fraction below the LOD with a reduction relative to baseline. Only patients with evaluable ctDNA measurements at both C1D1 and C3D1 are shown. The CR patient had undetectable baseline ctDNA with no change on treatment. C, cycle; D, day; ctDNA, circulating tumor DNA; BOR, best overall response; CR, complete response; PR, partial response; SD, stable disease; PD, progressive disease; NE, not evaluable; LOD, limit of detection.

**Extended Data Fig. 4:**
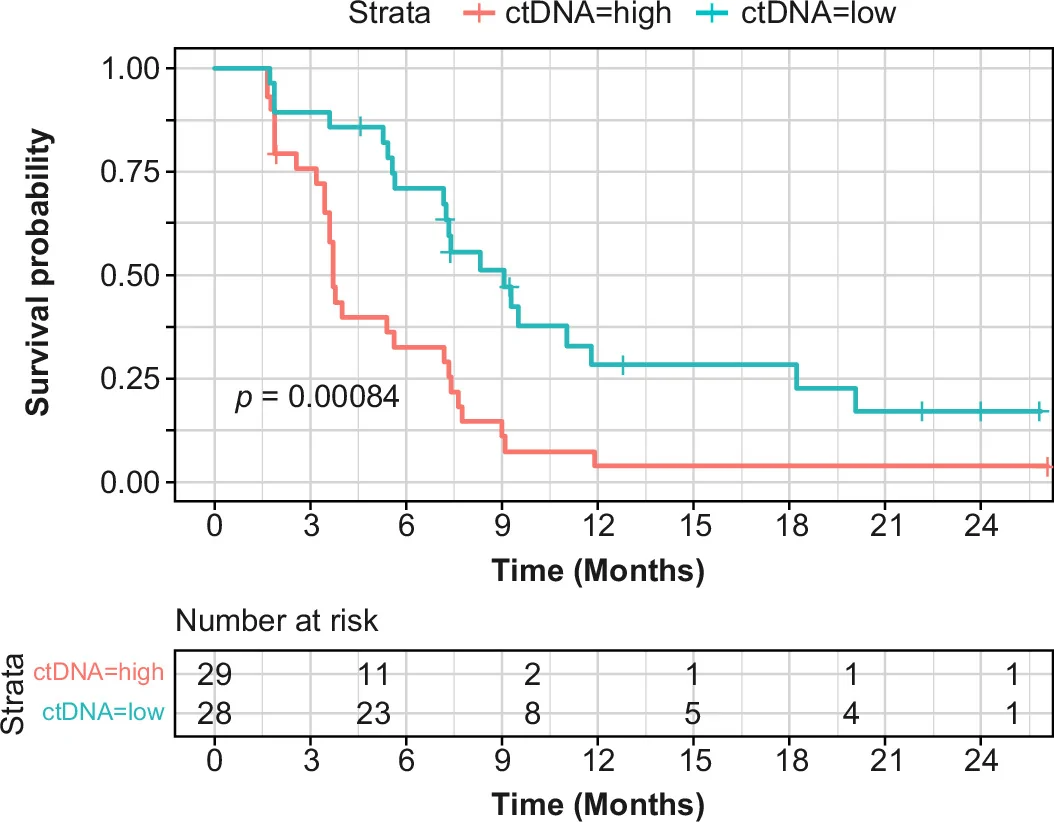
Kaplan-Meier curve illustrating the baseline circulating tumor DNA (ctDNA) as a prognostic marker of PFS. Patients with high ctDNA levels are represented by the red curve, while those with low ctDNA levels are shown in blue. Patients were stratified into ctDNA high or low at baseline depending on whether their ctDNA was lower or greater than or equal to the median value (ctDNA = 0.132) among all patients enrolled in the trial. A statistically significant difference in PFS is observed between the two groups (p = 0.00084). The table below the curves indicates the number of patients at risk at each time point for both ctDNA groups. Patients with a lower baseline ctDNA have a longer PFS. The group with a low ctDNA level comprises of patients with undetectable ctDNA and those with detectable ctDNA with a value less than the median (median ctDNA: 0.132). ctDNA, circulating tumor DNA; PFS, progression-free survival.0.132). ctDNA, circulating tumor DNA; PFS, progression-free survival.

**Extended Data Fig. 5:**
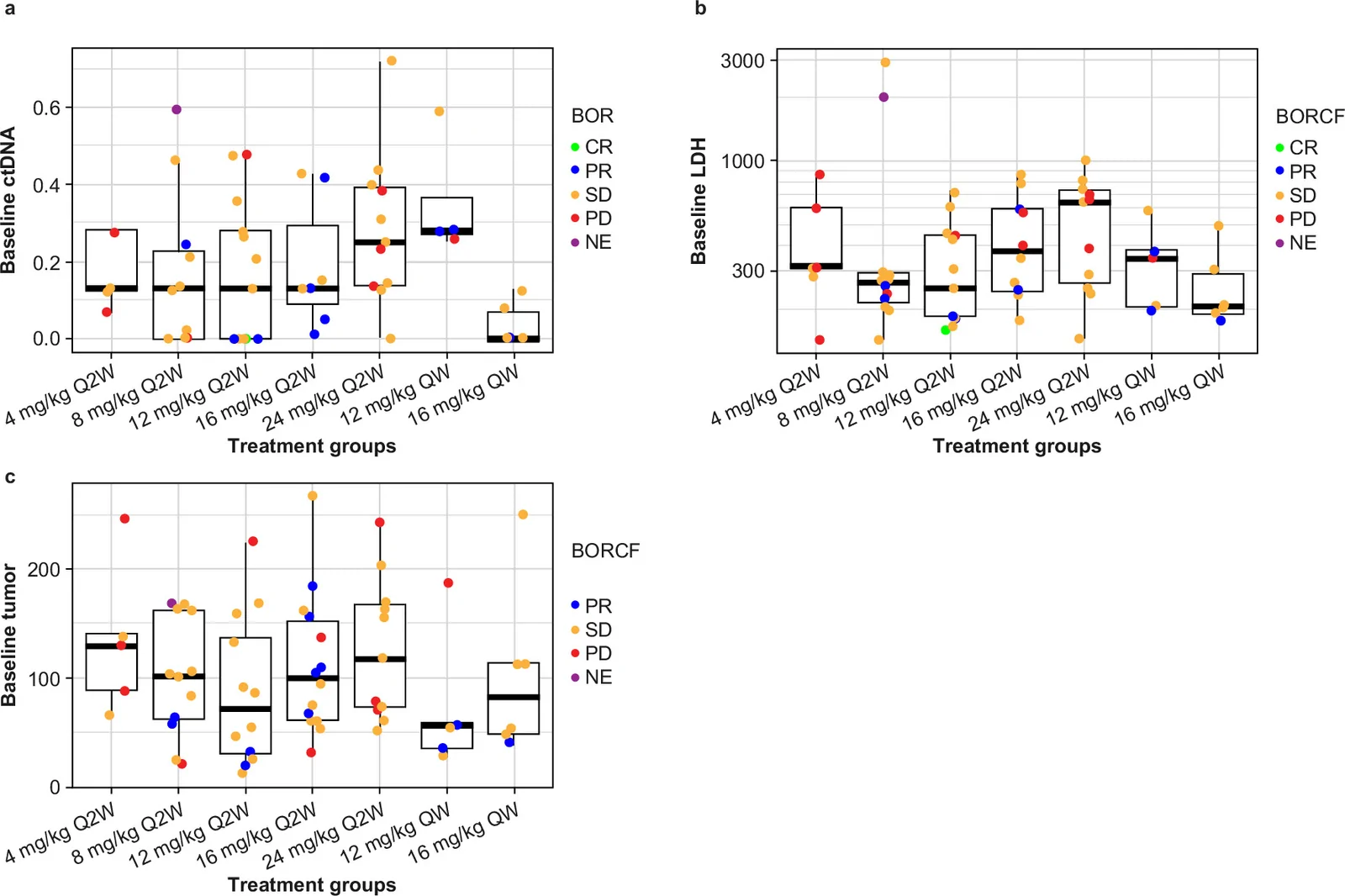
Baseline Biomarkers and Tumor Burden. (**a**) ctDNA, (**b**) LDH, and (**c**) tumor size by treatment doses. Patients enrolled at 24 mg/kg Q2W generally had worse results for prognostic markers compared with those in the other cohorts. BOR, best overall response; BORCF, best overall response with confirmation; CR, complete response; ctDNA, circulating tumor DNA; LDH, lactate dehydrogenase; NE, not evaluable; PD, progressive disease; PR, partial response; QW, once weekly; Q2W every 2 weeks; SD, stable disease.

**Extended Data Fig. 6:**
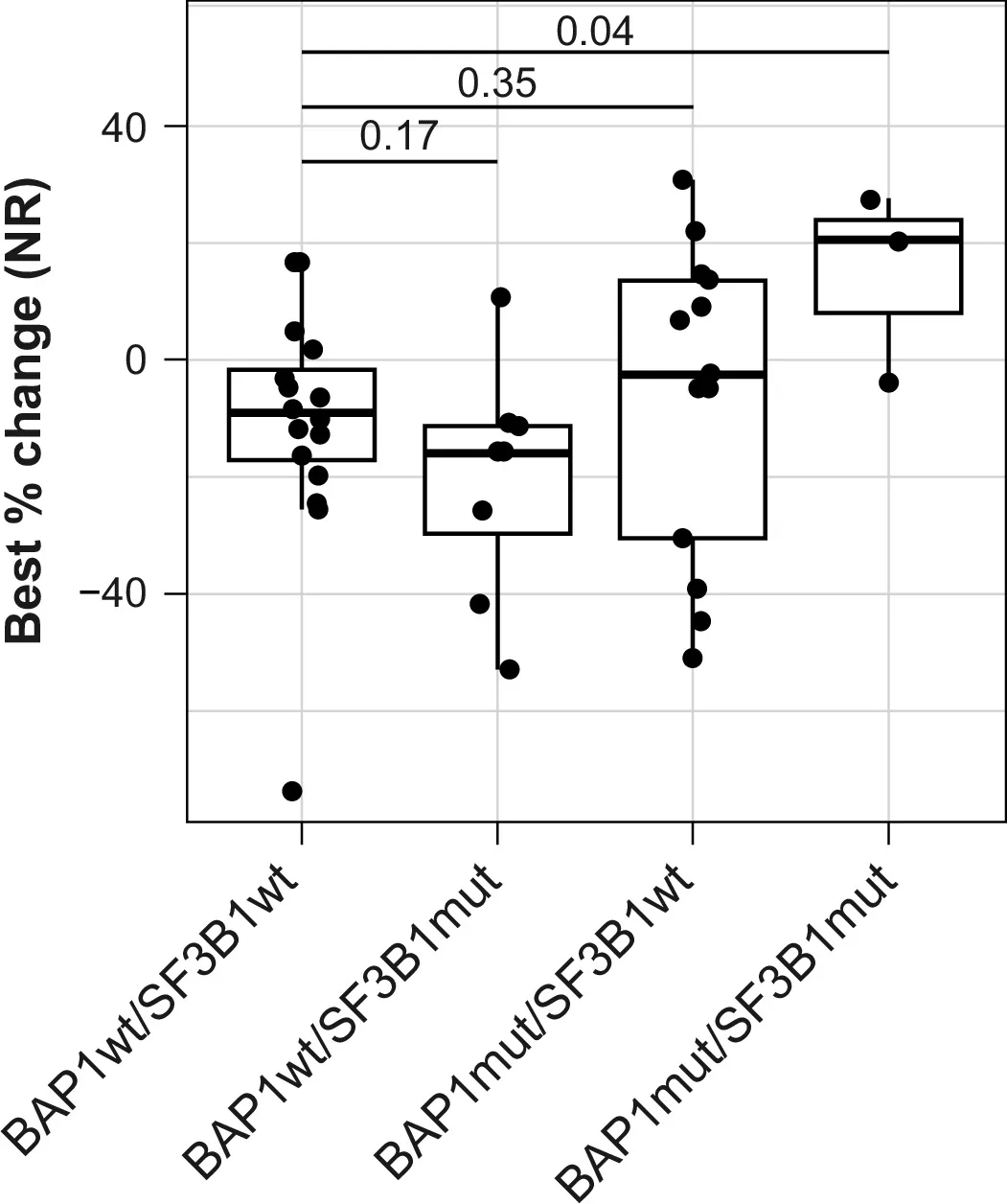
Baseline *BAP1/SF3B1* Mutation Status and Tumor Response. Box plot illustrates the relationship between baseline *BAP1/SF3B1* mutation (mut) status in ctDNA and best percentage change in nadir response (NR; only a single patient showed *EIF1AX* mutation here, precluding the inclusion of *EIF1AX* mutation status as additional columns in the plot). In particular in rare combination with *SF3B1* mutation, *BAP1* mutation appears to be associated with attenuated depth of response, with the double‑mutant group showing a shift toward tumor growth compared with *BAP1/SF3B1* wildtype tumors. We included only patients with a baseline ctDNA sample showing a GNAQ/11 mutation, ensuring that all selected patients had detectable ctDNA and minimizing issues related to low detection rate in ctDNA from patients with low levels of DNA shedding. A two-sample Wilcoxon rank sum test was used to derive p-values for each comparison depicted by the horizontal lines. N = 16 (BAP1wt/SF3B1wt), 9 (BAP1wt/SF3B1mut), 13 (BAP1mut/SF3B1wt) and 3 (BAP1mut/SF3B1mut.

**Extended Data Fig. 7:**
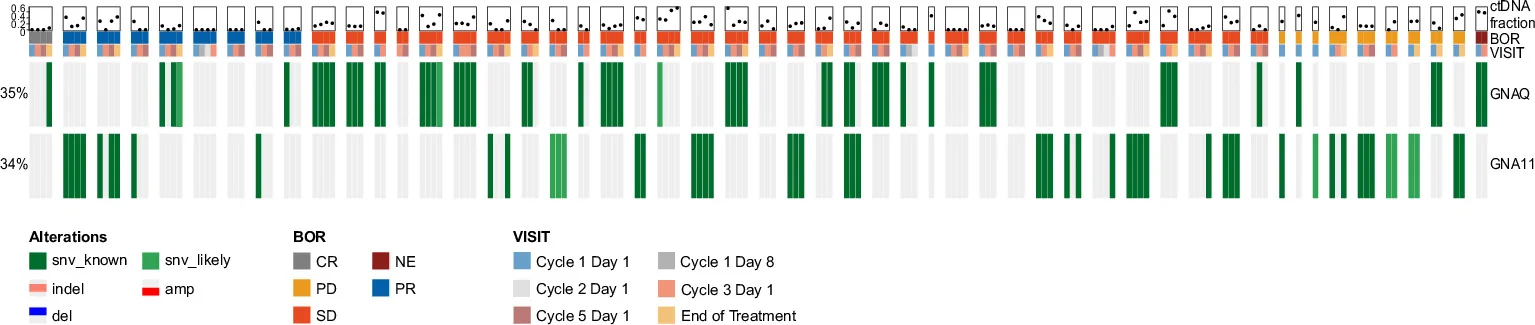
Stable GNAQ/11 Mutations During Treatment. GNAQ/11mut detected in plasma cell-free DNA suggests limited evidence of mutation status change with treatment. In OncoPrint, each column represents an individual patient sample, with each row indicating GNAQ (top) or GNA11 (bottom) mutation status. Across baseline and on-treatment samples, there is limited evidence of mutation status change in GNAQ/11, suggesting stability of these driver mutations during therapy. Please note that apparent changes in the mutation status seem more likely to be explained by the low ctDNA fraction (found at the top of the figure) in nearly every case. BOR, best overall response; CR, complete response; ctDNA, circulating tumor DNA; NE, not evaluable; PD, progressive disease; PR, partial response; SD, stable disease; snv, single nucleotide variant; indel, insertion/deletion; amp, amplification; del, deletion.

**Extended Data Fig. 8:**
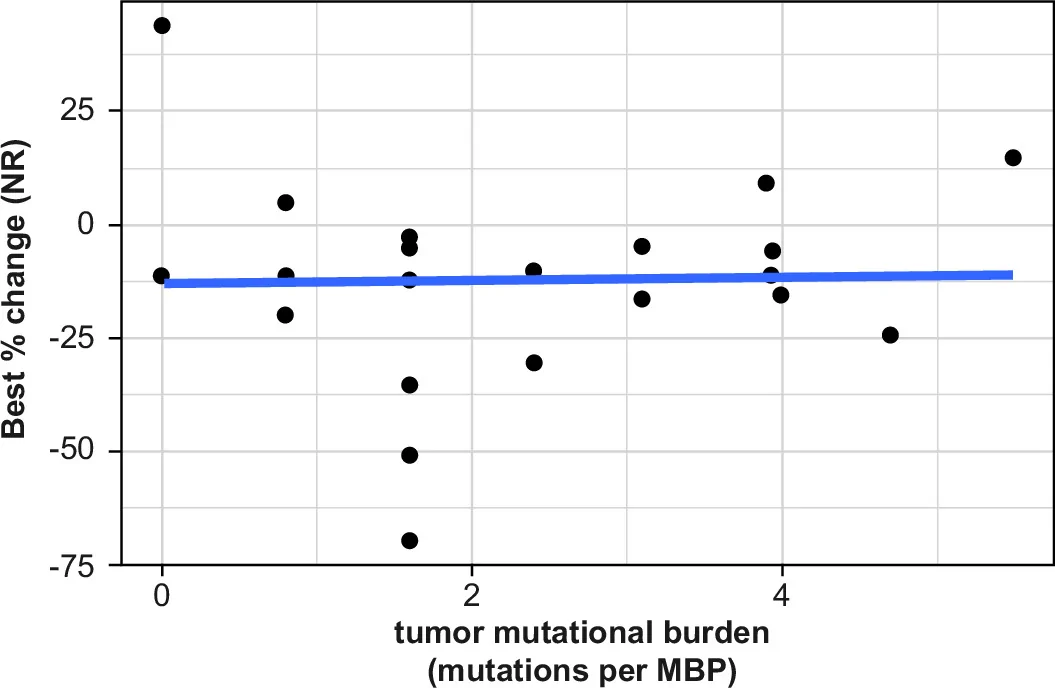
Tumor Mutational Burden and Tumor Response. TSO500 data scatter plot illustrating the relationship between tumor mutational burden (mutations per MBP) and the best percentage change in NR. Each black dot represents an individual patient’s data point. Mutational burden did not seem associated with best percent change in nadir response. MBP, megabase pair; NR, nadir response; TSO500, TruSight Oncology 500.

Patients with high baseline tumor burden, high LDH and high ctDNA fraction were less likely to respond/show tumor regression to treatment (Extended Data Fig. 2a–c). Treatment with DYP688 appeared to induce a reduction in ctDNA fraction at cycle 3, day 1 (C3D1) among patients who achieved a partial response, compared to those with stable disease or progressive disease (Extended Data Fig. 3). The baseline ctDNA levels were noted as a prognostic marker of PFS. Patients with a lower baseline ctDNA have a significantly longer PFS compared to patients with higher baseline ctDNA (*P* = 0.00084; Extended Data Fig. 4). Although an overall positive exposure–response relationship was observed, a diminished response was unexpectedly noted at the 24 mg kg^−1^ Q2W dose level (Extended Data Fig. 5). Additionally, exploratory analyses suggest differences in the distribution of best percentage change in tumor diameters across *BAP1* and *SF3B1* mutation groups, with attenuated reductions observed in tumors harboring *BAP1* mutations, particularly in rare combination with *SF3B1* mutations (Extended Data Fig. 6). *GNAQ*/*GNA11* mutation status, as detected in cell-free DNA (cfDNA), remained largely stable throughout treatment (Extended Data Fig. 7), and tumor mutational burden, as assessed by TruSight Oncology 500 (TSO500), was not correlated with best percentage change in tumor size (Extended Data Fig. 8).

### Biomarker analysis

**Fig. 4:**
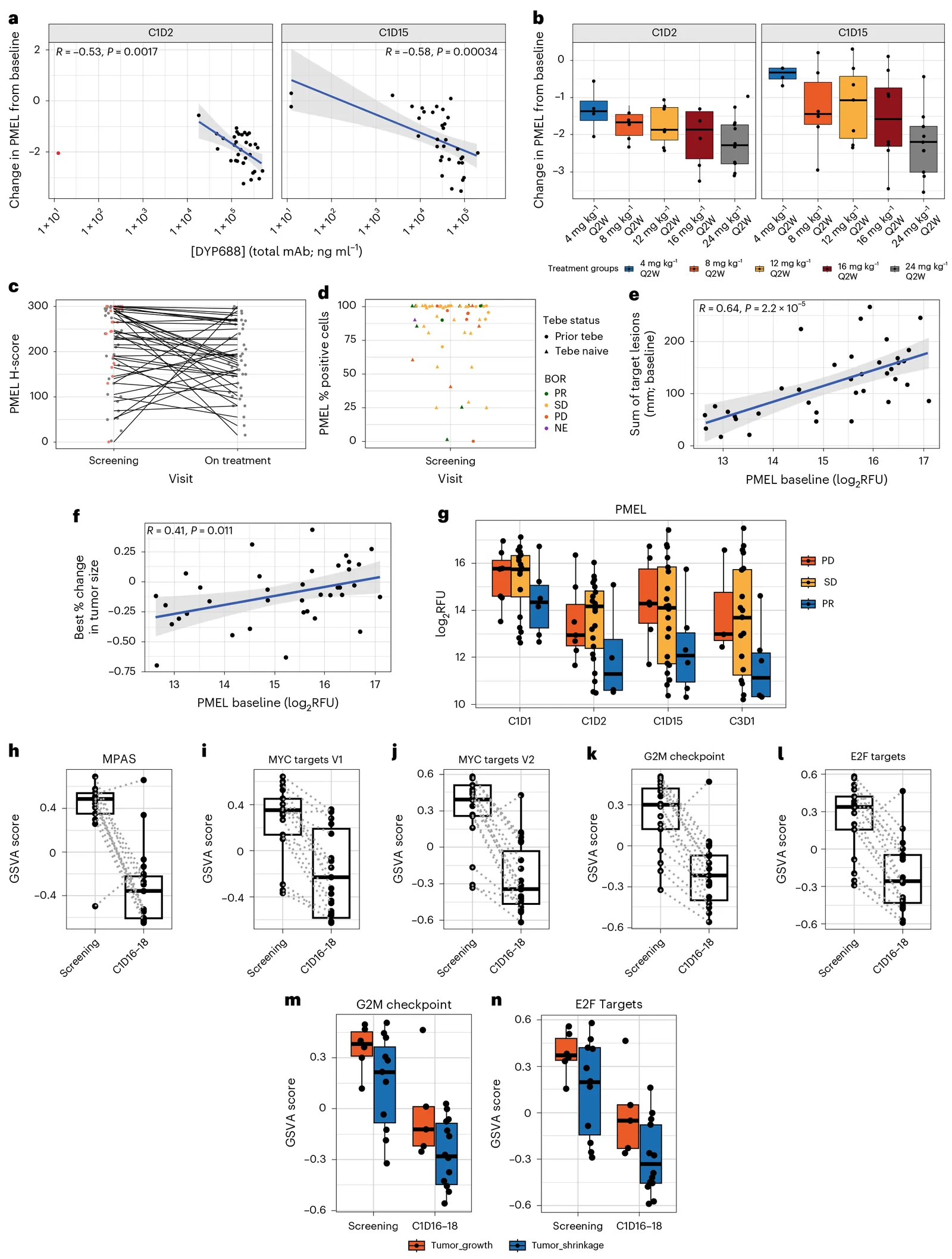
Biomarker data analysis suggesting the DYP688–PMEL interaction and key in situ molecular pathway modulation. **a**,**b**, Scatter plots (**a**) and box plots (**b**) showing the relationship between the change in circulating PMEL levels and drug exposure measured as an instantaneous concentration of total monoclonal antibody (mAb; **a**) and dose group (**b**). Pearsonʼs correlation coefficient and its two-sided test *P* value are indicated. Gray area denotes 95% CI. *n* = 33 (C1D2) and *n* = 34 (C1D15) biological replicates. **c**, PMEL H-scores on paired tumor biopsies at baseline and C1D16−C1D18 indicated no consistent change in PMEL expression in tumors after DYP688 exposure. Patients who progressed on prior tebentafusp treatment (red dots) did not differ in their PMEL expression at baseline compared to those who were naive to PMEL-targeted therapy (*n* = 57 at screening and *n* = 42 on-treatment). **d**, Scatter plot showing the percentage of PMEL-positive tumor cells at screening for individual patients. Each symbol represents one patient and is annotated by prior tebentafusp (tebe) regimen (shape) and BOR (color). **e**−**g**, Baseline PMEL concentrations in plasma (log_2_-transformed normalized relative fluorescence unit values (log_2_RFU)) plotted against the sum of the target lesion diameters (**e**, shortest and longest diameters for nodal and non-nodal lesions, respectively), best percentage change in tumor size measured by sum of target lesions (**f**) and categorized by treatment response (**g**). Pearsonʼs correlation coefficient and its two-sided test *P* value are indicated (**e**,**f**). Gray area denotes 95% CI. *n* = 37 biological replicates (**e**,**f**). *n* = 7 (PD C1D1), 24 (SD C1D1), 6 (PR C1D1), 7 (PD C1D2), 24 (SD C1D2), 4 (PR C1D2), 6 (PD C1D15), 24 (SD C1D15), 6 (PR C1D15), 3 (PD C3D1), 23 (SD C3D1) and 6 (PR C3D1) biological replicates (**g**). **h**−**l**, Box plots depicting gene set variation analysis (GSVA)-derived gene set scores for key molecular pathways, including MAPK pathway activity score (MPAS)^48^ (h) as well as hallmark MYC targets V1 (**i**), MYC targets V2 (**j**), G2M checkpoint (**k**) and E2F targets (**l**). *n* = 17 (screening) and *n* = 18 (C1D16−C1D18, 15 pairs) biological replicates. **m**,**n**, Box plots show GSVA scores for G2M checkpoint (**m**) and E2F targets (**n**) pathways at baseline (screening) and after treatment initiation (C1D16−C1D18). Non-responders (tumor growth; denoted in red) exhibited a higher baseline expression of proliferation-related pathways than responders (tumor shrinkage; denoted in blue). *n* = 6 (tumor_growth, screening), 11 (tumor_shrinkage, screening), 5 (tumor_growth, C1D16−C1D18) and 13 (tumor_shrinkage, C1D16−C1D18) biological replicates. All box plots show median (line), first and third quantiles (25th and 75th percentiles; hinges) as well as whiskers to the highest/lowest values no farther than ±1.5× the interquartile range from the hinge (all points are shown, and outliers are not duplicated). NE, not evaluable.

Proteomic profiling on plasma samples collected from 38 patients was performed using the SomaScan assay^27,28^. Circulating unbound plasma PMEL showed a dose-dependent reduction after DYP688 administration, consistent with target binding (Fig. 4a). At lower DYP688 doses, a rebound in unbound PMEL was observed at cycle 1, day 15 (C1D15) (Fig. 4b), indicating that there is insufficient DYP688 to bind and engage the newly synthesized and/or shed PMEL.

Core needle biopsies were collected from tumor tissue at baseline and at cycle 1 between days 16 and 18 (C1D16−C1D18). PMEL expression in biopsies was analyzed by immunohistochemistry (IHC). Levels of PMEL in situ at baseline (*n* = 56 evaluable) were measured by IHC varied across the patient population. Furthermore, prior treatment with PMEL-targeted tebentafusp did not seem to affect this variability in expression. When comparing PMEL H-scores at baseline with those from the C1D16−C1D18 timepoint (*n* = 41 evaluable pairs), no clear directionality of change was observed, indicating that treatment with DYP688 did not uniformly affect the PMEL status of the sampled tumors at C1D16−C1D18 (Fig. 4c), supporting the hypothesis that changes in circulating PMEL likely stem from engagement of the protein by DYP688 rather than changes in expression of PMEL by the tumor. In addition to the IHC H-score analysis, the percentage of PMEL-positive cells at baseline is shown in Fig. 4d. This information is presented alongside BOR and prior tebentafusp therapy for each patient, enabling a more granular evaluation of the relationship between baseline PMEL expression and clinical outcome. Most patients exhibited high levels of PMEL expression at screening. Clinical responses appeared independent of baseline PMEL expression, and no clear differences in PMEL expression were observed according to prior treatment history.

Of note, baseline PMEL concentrations in circulation were positively associated with tumor burden (sum of target lesion diameters) and treatment response (Fig. 4e–g), supporting its potential as a pharmacodynamic marker and a potential additional biomarker for tumor burden and clinical outcome^29^.

In line with the mechanism of action of DYP688, RNA-sequencing data from liver metastasis biopsy samples showed a significant downregulation in gene signature scores for mitogen-activated protein kinase (MAPK) pathway activity and pathways associated with cell cycle and proliferation after treatment compared to baseline samples (Fig. 4h–l). Stratification of patients based on treatment response revealed notable differences in transcriptomic profiles from baseline and after start of treatment. Non-responders exhibited higher baseline expression of proliferation-related pathways, suggesting that intrinsic pathway activation may limit therapeutic efficacy. By contrast, responders demonstrated a marked downregulation of glycolysis-related pathways after treatment, a pattern not observed in non-responders (Fig. 4m,n). These findings suggest distinct molecular mechanisms underlying therapeutic response, with potential implications for patient stratification and the development of personalized therapeutic strategies.

## Discussion

We show here that a novel therapeutic strategy of an ADC with a biology-matched ‘targeted therapy’ payload is feasible and can achieve clinical efficacy with minimal toxicity in patients with mUM or other *GNAQ*/*GNA11*-mutant melanomas. Using PMEL-directed trafficking and an ADC platform, DYP688 successfully delivers the potent G_q/11_ inhibitor payload SDZ475, which is otherwise undeliverable because of its toxicity and pharmacokinetics profile. The drug demonstrated a favorable safety profile at a dose range of 4−24 mg kg^−1^ Q2W and 12−16 mg kg^−1^ QW. Antitumor activity was observed at doses ≥8 mg kg^−1^ with objective responses in patients who commenced treatment at doses ≥12 mg kg^−1^ or after intrapatient dose escalation to 24 mg kg^−1^ Q2W. Although the overall response rate (ORR) was approximately 20%, the DCR was high (approximately 82%), with many patients experiencing stable disease and measurable tumor shrinkage, indicating clinically meaningful benefit beyond confirmed responses. The modest ORR likely reflects several factors, including the inclusion of patients treated in early dose-escalation cohorts who received doses below the biologically active range, intratumoral and intertumoral heterogeneity influencing response despite the presence of *GNAQ*/*GNA11* driver mutations and the potential emergence of resistance, among other factors. As this study represents the dose-escalation phase of a phase 1 clinical trial, with primary objectives focused on safety, tolerability and determination of a recommended dose, the evaluation of the antitumor activity of DYP688 remains preliminary. Consequently, the interpretation of these findings is limited by the small sample size, heterogeneity in dosing regimens and other inherent limitations of early-phase studies.

The MTD was not identified, as DYP688 demonstrated an acceptable safety and tolerability profile across all administered dose levels and schedules. The recommended dose was established at 16 mg kg^−1^ Q2W, based on safety, preliminary antitumor activity, pharmacokinetics, positive target engagement and biomarker data. At the recommended dose of 16 mg kg^−1^ Q2W, the median duration of treatment (range) was 7.2 (2.8−24.8) months, and objective responses were seen in five of 14 patients (35.7%). The median PFS (95% CI) was 7.4 (3.7−20.6) months.

Baseline tumor burden, ctDNA and LDH are well-known prognostic factors in mUM^30,31^. This was reaffirmed in patients treated with DYP688, where higher baseline tumor burden, ctDNA and LDH were associated with lower likelihood of response. The antitumor activity of DYP688 is further supported by ctDNA kinetics analysis, which showed a rapid and early reduction in ctDNA levels after treatment initiation. Notably, a dose-dependent relationship was observed between DYP688 exposure and ctDNA reduction as well as between DYP688 exposure and tumor size reduction, reinforcing the link between drug activity and molecular response. Patients with lower baseline ctDNA also demonstrated longer PFS, suggesting that ctDNA may serve as both a predictive and a prognostic biomarker. Although a positive exposure−response relationship was observed overall, the response rates were slightly inferior at the 24 mg kg^−1^ Q2W dose level. This may be partially explained by the higher baseline LDH and ctDNA in this cohort, suggesting a poorer prognosis. This confounding effect of baseline factors could have contributed to the rapid progression observed in patients treated at this dose level.

Exploratory analyses suggested that tumors harboring BAP1 mutations detected in ctDNA at baseline, particularly in rare combination with SF3B1 mutations, tend to exhibit attenuated depth of response, although substantial overlap was observed across mutation groups. These findings should be interpreted cautiously given the limited sample size and the analytical constraints of ctDNA‑based mutation assessment. Nevertheless, they are directionally consistent with previous literature showing that germline or tissue‑based BAP1 alterations are associated with more aggressive disease biology in uveal melanoma, including earlier onset, larger tumors, increased ciliary body involvement and higher metastatic risk. Indeed, BAP1 mutations have been associated with significantly reduced survival and an increased risk of metastasis compared to BAP1‑intact tumors^32–34^.

Reflecting the pharmacodynamic activity of DYP688, transcriptomic analysis of paired liver metastasis samples (baseline and on-treatment) revealed a significant downregulation of MAPK pathway activity scores, along with other cell proliferation-associated pathways within the tumor. These findings are consistent with the known mechanism of action of DYP688, which delivers a G_q/11_ inhibitor intracellularly via PMEL targeting. Non-responders exhibited higher baseline expression of proliferation pathways, suggesting that elevated proliferative signaling may be associated with resistance. By contrast, responders showed a marked suppression of glycolysis-related pathways after treatment. The upstream targeting by DYP88 contrasts with previous strategies that focused on downstream effectors. Data from previous studies showed that MEK inhibition alone failed to mimic *GNAQ* silencing, whereas combined MEK and AKT inhibition induced synergistic cell death, underscoring the need to simultaneously block parallel survival pathways^35^. Similarly, PKC inhibitors such as darovasertib and sotrastaurin have aimed to suppress MAPK signaling downstream of *GNAQ* and *GNA11*, with variable efficacy and emerging resistance^36,37^. More recently, PKC inhibition in combination with the tyrosine kinase inhibitor crizotinib has been investigated as a means to deepen pathway suppression and overcome adaptive resistance. Crizotinib has activity against ALK, ROS1 and MET^38^, and, whereas the precise contributors to clinical benefit remain uncertain, preclinical studies suggest that the addition of a tyrosine kinase inhibitor to PKC blockade more effectively attenuates compensatory signaling through MAPK and PI3K pathways, resulting in enhanced apoptotic responses compared to PKC inhibition alone. Consistent with this, recent clinical data from darovasertib plus crizotinib have demonstrated a significant improvement in efficacy compared to investigator’s choice, supporting the rationale for targeting both PKC-dependent signaling and MET-mediated pathways^39,40^.

Epigenetic modulation using histone deacetylase inhibitors in combination with MEK inhibitors has shown promise in overcoming such resistance^41^. Compared to these approaches, DYP688 offers a more direct and potentially durable strategy by targeting the root oncogenic driver, thereby attenuating multiple downstream pathways simultaneously without the toxicity challenges typically seen with targeted therapy combinations. However, as with other targeted approaches, resistance can still develop.

DYP688 is well tolerated, with most adverse events considered related to the study drug being grade 2 or lower, and no MTD was determined. Only five grade 3 events were reported: hypotension (lasting 1 day at 24 mg kg^−1^ Q2W), asymptomatic hypercalcemia, anemia, increased GGT and decreased lymphocyte count. The episodes of hypercalcemia are hypothesized to result from potential effects of SDZ475 on the calcium‑sensing receptor (CaSR) pathway, which signals through Gα11 to regulate calcium homeostasis^42^.

The relatively good tolerance is likely related to the pharmacokinetics characteristics of DYP688. DYP688 demonstrated a dose-dependent increase in systemic pharmacokinetics exposure (AUC) in all analyses, including total antibody, conjugated active payload and free payload, over the investigated dose range. This trend was consistent across both QW and Q2W dosing regimens. The conjugated active payload demonstrated a shorter elimination half-life compared to the total antibody (2 days versus 9 days), in line with preclinical predictions. This is attributed to the payload inactivation by enzymatic ring opening while still attached to the antibody backbone. Crucially, the free payload, representing the unconjugated pharmacologically active moiety, showed markedly lower systemic exposure compared to the conjugated form. At the highest tested dose on the Q2W regimen (24 mg kg^−1^), the AUC_0−336 h_ of free payload in blood was approximately 80-fold lower than that of the conjugated active payload in plasma. This substantial differential suggests that the ADC design effectively limits systemic exposure to the free toxic agent, minimizing toxicity while maintaining therapeutic efficacy. The low systemic levels of free payload are particularly important from a safety standpoint, as they suggest that the ADC exhibits good stability in systemic circulation, with the payload largely remaining conjugated to the antibody for targeted delivery to the tumor. This aligns with the favorable safety profile of DYP688 observed in this study. At the doses of DYP688 tested, we observed evidence of target engagement, as demonstrated by a dose-dependent decrease in circulating PMEL levels in plasma. We hypothesize that the decrease in measured plasma PMEL is due to the competitive interference of the ligand binding assay by DYP688.

Patients with mUM have limited therapeutic options. Monotherapy with immune checkpoint inhibitors (ICIs), such as programmed cell death protein 1 (PD-1), programmed death ligand 1 (PD-L1) or cytotoxic T-lymphocyte-associated protein 4 (CTLA-4) inhibitors, has shown little clinical benefit in patients with mUM, with response rates typically below 10% and median overall survival often under 12 months^43^. Similarly, recent studies of ICI combination regimens for mUM have also revealed modest clinical activity. In studies of nivolumab plus ipilimumab, the ORR was of 11−16%, and the median overall survival was 15−18 months^44,45^. In another study, nivolumab combined with relatlimab was associated with a low response rate in mUM^46^. ICI combination regimens can be toxic; for example, ipilimumab plus nivolumab has been associated with rates of immune-related adverse events up to 80%^43,44^. Targeting downstream of *GNAQ* and *GNA11* has been explored in mUM, with multiple targets, including PKC, MEK and AKT, investigated^47^. Most of these agents have had modest activity and challenging toxicity profiles that have limited both dose escalation and exploration of combinations. Perhaps the most attractive of these agents is the selective PKC inhibitor darovasertib (IDE196, formerly LXS196). Darovasertib monotherapy has a response rate of 9%, with dosing limited by hypotension and 25% of patients experiencing a grade 3 or 4 TRAE. In a phase 2 study, the combination of darovasertib and crizotinib, a c-MET inhibitor, was more active than darovasertib alone, with an ORR of 30%. The combination, however, was associated with higher toxicity, including significant gastrointestinal toxicities, with a grade 3/4 TRAE rate of over 30% and 7.4% of patients requiring a dose discontinuation^39^. DYP688 has broadly similar efficacy with excellent tolerance across all tested doses. The DCR rate was 81.8%, and the ORR and median PFS (95% CI) at the recommended dose of 16 mg kg^−1^ Q2W were 35.7% and 7.4 (3.7−20.6) months, respectively.

The continued expression of PMEL in tumors from patients who had progressed on prior PMEL-targeting therapy with tebentafusp suggests that DYP688 may remain a viable treatment option for this population. Notably, objective responses were observed in both HLA-A02:01-positive patients previously treated with tebentafusp and HLA-A02:01-negative patients. TRAEs were predominantly grade 2 or lower; no grade 4 or 5 TRAEs were observed; and no patients discontinued treatment due to TRAEs. The observed activity and favorable safety profile support further investigation of DYP688 as both a single agent and, potentially, in combination regimens for patients with mUM.

## Methods

### Ethical approval and consent

This study was conducted in accordance with the International Council for Harmonization of Technical Requirements for the Registration of Pharmaceuticals for Human Use (ICH) Harmonized Tripartite Guidelines for Good Clinical Practice, with applicable local regulations, including European Directive 2001/20/EC and US Code of Federal Regulations Title 21, and the Declaration of Helsinki. The protocol (provided as Supplementary Information) and all amendments were reviewed and approved by appropriately constituted institutional review boards/independent ethics committees/research ethics boards (IRBs/IECs/REBs) prior to study initiation. The approving institutions/organizations and their respective IRBs/IECs/REBs included Bellberry Human Research Ethics Committee; Peter MacCallum Cancer Centre Ethics Committee–Melbourne; the Ethics Committee assigned under the EU Clinical Trials Regulation (France); Ethik-Kommission der Medizinischen Fakultät der Universität Duisburg-Essen; Medisch-Ethische Toetsingscommissie–Leiden, Den Haag, Delft; Columbia University IRB–Human Research Protection Office; Dana-Farber Cancer Institute–Office of Human Research Studies; Memorial Sloan Kettering Cancer Center IRB/Privacy Board; HM Hospitales CEIm–Comité de Ética de la Investigación con Medicamentos; and Kanton Zurich–Kantonale Ethikkommission. Eligible patients provided written IRB/IEC/REB-approved informed consent before study initiation. Patients received reasonable travel reimbursement per local contracts.

### Study design

This phase 1/2, open-label, multicenter, dose-escalating study evaluated DYP688 as a single agent in patients with mUM and other *GNAQ-* or *GNA11*-mutant melanomas.

During dose escalation, patients received DYP688 intravenously at doses ranging from 4 mg kg^−1^ to 24 mg kg^−1^ Q2W, or 12 mg kg^−1^ to 16 mg kg^−1^ QW, in 28-day cycles. Treatment continued until disease progression, intolerable toxicity or withdrawal of consent.

The starting dose of DYP688 was 4 mg kg^−1^ Q2W. Thereafter, cohorts of up to six patients received DYP688 at one of the following doses on a Q2W schedule: 8 mg kg^−1^, 12 mg kg^−1^, 16 mg kg^−1^ and 24 mg kg^−1^, or at 12 mg kg^−1^ and 16 mg kg^−1^ QW. Dose escalation was guided by safety, pharmacokinetics data and a two-parameter Bayesian logistic regression model (BLRM) employing escalation with overdose control (EWOC) criteria. The DLT period lasted for 28 days after the first dose of DYP688. A patient from a cohort was evaluable for the dose-escalation decision if they received at least two doses on the Q2W schedule or three doses on the QW schedule or if they experienced a DLT. Additional cohorts of up to six patients were enrolled at dose levels of interest to generate further safety, pharmacokinetics, pharmacodynamics and preliminary efficacy data. Intrapatient dose escalation was permitted per study protocol for patients who had tolerated their originally assigned dose for at least four cycles and had not experienced any TRAE of grade 2 or higher. The higher dose with which the patient was to be treated must be a dose that had completed evaluation and been shown to satisfy the EWOC principle. A second intrapatient dose escalation was permitted for such patients after at least two further cycles and if they continued to tolerate treatment with no TRAE of grade 2 or higher. Being a phase 1 study, no hypothesis testing was planned, and, therefore, no formal sample size calculation was needed. The sample size was selected to ensure that the BLRM had adequate operating characteristics when selecting the MTD and/or recommended dose(s). If an MTD was to be identified, it was defined in the protocol as the highest dose estimated to have less than 25% risk of causing a DLT during the DLT period (first 28 days of treatment) in more than 33% of patients treated. However, any dose(s) less than the MTD could be selected as the recommended dose(s) for further evaluation in the dose-expansion part of the study, taking into consideration all available safety, pharmacokinetics, pharmacodynamics and efficacy data generated in the dose-escalation part.

Adverse events were assessed according to National Cancer Institute Common Terminology Criteria for Adverse Events (NCI-CTCAE) version 5. For patients who did not tolerate their assigned dosing schedule due to TRAEs, dose adjustments were permitted to allow the patient to continue study treatment once the adverse event had resolved to grade 1 or lower. Tumor response was assessed locally by computed tomography and/or magnetic resonance imaging of the chest, abdomen and pelvis, according to RECIST version 1.1 at baseline, on C3D1 and every two cycles thereafter.

Serial blood samples for pharmacokinetics analysis were collected from all patients at multiple timepoints through C6D1. Intensive pharmacokinetics sampling (baseline; 30 min; 1, 4, 24 and 72 h; 168 h (7 days); and up to 336 h (14 days) after dose) was conducted after the first dose of DYP688 and during cycle 3 of treatment. DYP688 concentrations (as total antibody) in human serum were quantified using a validated ligand binding assay. Conjugated active and inactive phosphorylated payload concentrations in plasma were measured using liquid chromatography−tandem mass spectrometry (LC−MS/MS). Unconjugated payload (SDZ475) concentrations in blood were determined with a separate validated LC−MS/MS assay. Pharmacokinetics parameters were determined by non-compartmental methods, using Phoenix WinNonlin version 8.3 (Certara).

Metastatic tumor biopsies were collected at baseline (prior to first dose) and on-treatment (C1D16−C1D18) to evaluate the modulation of proteins downstream of G_q/11_ in tumors after exposure to DYP688 and to perform exploratory transcriptome and targeted DNA sequencing. Details of sample collection and preparation of DNA and RNA extraction are provided in the Supplementary Information.

For exploratory cfDNA sequence analysis, blood samples were collected from all patients at multiple timepoints while on-treatment (C1D1 pre-infusion, C1D8, C2D1, C3D1, C5D1 and end of treatment) to explore whether early reduction of ctDNA fraction correlated with response.

### Participants

The study recruited male or female patients at least 18 years of age with mUM and other *GNAQ*/*GNA11*-mutant melanomas. Eligible patients had an Eastern Cooperative Oncology Group performance status (ECOG PS) score of ≤1, with histologically or cytologically confirmed metastatic disease measurable by RECIST version 1.1. Patients with mUM were either treatment naive or had progressed on their most recent prior therapy. Patients with advanced cutaneous or other melanoma with *GNAQ*/*GNA11* mutations had to have progressed on all available standard-of-care treatments. Most patients had received prior local treatment (radiotherapy and/or surgery) for the primary uveal melanoma in accordance with standard of care; therefore, DYP688 treatment response in primary lesions could not be evaluated.

Key exclusion criteria included patients with symptomatic brain metastases or leptomeningeal disease, evidence of active bleeding or bleeding diathesis or significant coagulopathy and history of anaphylactic or other severe hypersensitivity/infusion-related reactions to ADCs or monoclonal antibodies.

### Study objectives and endpoints

For the dose-escalation (phase 1) part, the primary objective was to characterize safety and tolerability and define the MTD and/or recommended dose(s) and regimen(s) of DYP688. The primary safety endpoint was the occurrence of DLTs during the first 28 days of treatment. Additional safety endpoints included the incidence and severity of adverse events and SAEs, including changes in laboratory values, vital signs and electrocardiogram. Tolerability endpoints included dose interruptions, dose reductions and study drug discontinuations.

Secondary objectives of the study were to (1) characterize the pharmacokinetics of DYP688; (2) evaluate the preliminary antitumor activity of DYP688 as a single agent with the assessment of the BOR rate and ORR (defined as the proportion of patients with BOR of either confirmed complete response or confirmed partial response per RECIST version 1.1); and (3) assess the immunogenicity of DYP688. Exploratory efficacy endpoints included PFS and overall survival rates. Additional exploratory objectives were related to the pharmacodynamic effect of DYP688 and included assessments of (1) the presence of the ADC target in the tumor by measuring PMEL protein expression at baseline and its changes, on-treatment and at the end of treatment; (2) pharmacokinetics/pharmacodynamics relationship and quantifying the extent of target inhibition by correlating DYP688 exposure and the changes; and (3) mutations in cancer driver genes by transcriptome and targeted DNA sequencing of baseline tumor biopsies.

### Statistical analysis

Data are summarized using descriptive statistics (continuous data) and/or contingency tables (categorical data) for demographic and baseline characteristics, efficacy, safety and pharmacokinetics measurements. Patients who received at least one dose of study treatment were included in safety/tolerability and efficacy analyses. Pharmacokinetics analyses were conducted in all patients who had data for at least one evaluable pharmacokinetics concentration. For the concentration to be evaluable, a patient (1) must have received at least one of the planned treatments and (2) must have at least one primary pharmacokinetics parameter.

PFS is described using the Kaplan−Meier analysis. Pharmacodynamics analysis for the proximal pharmacodynamic markers was performed in newly obtained pre-treatment and on-treatment tumor biopsies (on C1D16−C1D18) from patients who received DYP688. Changes from baseline on these pharmacodynamic markers were used to investigate the effect of DYP688 to evaluate how they might relate to systemic exposure and clinical outcomes. See the Supplementary Information for details on the biomarker methodology.

Owing to the exploratory nature of first-in-human studies, formal statistical tests were not planned to be performed on pharmacodynamics data, which are presented using descriptive statistics and visualizations. Continuous variables are summarized as median and range. Categorical variables are summarized using counts and percentages.

Study data were analyzed using the most updated version of SAS software. Statistical analyses were performed using R (version 3.4.3 or higher).

### Reporting summary

Further information on research design is available in the Nature Portfolio Reporting Summary linked to this article.

## Online content

Any methods, additional references, Nature Portfolio reporting summaries, source data, extended data, supplementary information, acknowledgements, peer review information; details of author contributions and competing interests; and statements of data and code availability are available at https://doi.org/10.1038/s41591-026-04518-z.

## Supplementary information

Supplementary Information Methods, Tables 1−4, Figs. 1 and 2, References, Clinical Study Protocol and Statistical Analysis Plan.

Reporting Summary

Peer Review File

## Data Availability

All data associated with this study are in the paper or the Supplementary Information, with the exception of individualized patient genomic and other exploratory biomarker data (including SomaScan and bulk RNA sequencing). No new code was generated for this study. Novartis is committed to sharing with qualified external researchers access to patient-level clinical data and supporting clinical documents from the clinical study. These requests are reviewed and approved by an independent review panel on the basis of scientific merit. All data provided are anonymized to respect the privacy of patients who have participated in the trial, in line with applicable laws and regulations. However, IRB approval and patient consent to share individualized patient genomic data (including SomaScan and bulk RNA sequencing) are not available; therefore, in accordance with the Health Insurance Portability and Accountability Act, these data cannot be reported in a public data repository. It is also Novartis policy for small phase 1 studies to avoid sharing this data type (that is, patient-level exploratory biomarker/genomic results) due to the risk of patient reidentification. Upon request, Novartis can provide gene-set-level aggregate values. In addition, clinical data, in some cases, have been collected under contractual or consent provisions that prohibit transfer to third parties. Such restrictions may preclude granting access under these provisions. Where co-development agreements or other legal restrictions prevent sharing particular data, Novartis will work with qualified requestors to provide summary information where possible. The data availability of these trials is according to the criteria and process described at https://www.clinicalstudydatarequest.com/, which is also where requests to access data, including gene-set-level aggregate data, may be made. Any proprietary or patient-derived materials used or generated in this study are not commercially available and cannot be shared. All other materials in this study are commercially available.

## Extended data

**Extended Data Table 1:** Baseline metastatic category (AJCC) of UM patients by dose cohort

Baseline characteristics	4 mg/kg Q2W N=5	8 mg/kg Q2W N=12	12 mg/kg Q2W N=13	16 mg/kg Q2W N=14	24 mg/kg Q2W N=11	12 mg/kg QW N=5	16 mg/kg QW N=6	All patients N=66
Number of UM patients	5	12	9	13	10	5	6	60
Metastatic category at baseline (AJCC), n (%)								
M0	0	0	0	1 (7.7)	0	0	0	1 (1.7)
M1	1 (20.0)	0	0	0	0	0	0	1 (1.7)
M1a	1 (20.0)	2 (16.7)	4 (44.4)	4 (30.8)	3 (30.0)	2 (40.0)	3 (50.0)	19 (31.7)
M1b	1 (20.0)	5 (41.7)	2 (22.2)	5 (38.5)	5 (50.0)	2 (40.0)	1 (16.7)	21 (35.0)
M1c	2 (40.0)	5 (41.7)	3 (33.3)	3 (23.1)	1 (10.0)	1 (20.0)	2 (33.3)	17 (28.3)
Unknown	0	0	0	0	1 (10.0)	0	0	1 (1.7)

AJCC, American Joint Committee on Cancer; QW, once weekly; Q2W, every 2 weeks; UM, uveal melanoma; N, number of patients.

**Extended Data Table 2:** Best overall response summary

	4 mg/kg Q2W N=5 n (%)	8 mg/kg Q2W N=12 n (%)	12 mg/kg Q2W N=13 n (%)	16 mg/kg Q2W N=14 n (%)	24 mg/kg Q2W N=11 n (%)	12 mg/kg QW N=5 n (%)	16 mg/kg QW N=6 n (%)	All Patients N=66 n (%)
Number of evaluable patients	5	12	13	14	11	5	6	66
Subject with measurable disease at baseline	5 (100)	12 (100)	12 (92.3)	14 (100)	11 (100)	5 (100)	6 (100)	65 (98.5)
Subject with non-measurable disease only at baseline	0	0	1 (7.7)	0	0	0	0	1 (1.5)
Best overall response* - n (%)								
CR	0	0	1 (7.7)	0	0	0	0	1 (1.5)
PR	0	2 (16.7)	2 (15.4)	5 (35.7)	0	2 (40.0)	1 (16.7)	12 (18.2)
SD	2 (40.0)	8 (66.7)	9 (69.2)	7 (50.0)	8 (72.7)	2 (40.0)	5 (83.3)	41 (62.1)
PD	3 (60.0)	1 (8.3)	1 (7.7)	2 (14.3)	3 (27.3)	1 (20.0)	0	11 (16.7)
NE	0	1 (8.3)	0	0	0	0	0	1 (1.5)
ORR: CR+PR	0	2 (16.7)	3 (23.1)	5 (35.7)	0	2 (40.0)	1 (16.7)	13 (19.7)
95% CI	(0.0,52.2)	(2.1,48.4)	(5.0,53.8)	(12.8,64.9)	(0.0,28.5)	(5.3,85.3)	(0.4,64.1)	(10.9,31.3)
DCR: CR+PR+SD	2 (40.0)	10 (83.3)	12 (92.3)	12 (85.7)	8 (72.7)	4 (80.0)	6 (100)	54 (81.8)
95% CI	(5.3,85.3)	(51.6,97.9)	(64.0,99.8)	(57.2,98.2)	(39.0,94.0)	(28.4,99.5)	(54.1,100.0)	(70.4,90.2)

*Assessed by local radiology review. CI, confidence interval; CR, complete response; DCR, disease control rate; NE, not evaluable; ORR, overall response rate; PD, progressive disease; PR, partial response; QW, once weekly; Q2W, every 2 weeks; SD, stable disease.
