# Supplementary material for: An anti-PMEL antibody−drug conjugate with a Gq/11 inhibitor payload in GNAQ/GNA11-mutant melanomas: a phase 1 trial
Source: Nat Med. 2026 Jul 13;32(9):3248–58. doi: 10.1038/s41591-026-04518-z (PMC13577922; doi:10.1038/s41591-026-04518-z)
Supplement: Supplementary file 1 [file 41591_2026_4518_MOESM1_ESM.pdf]

nature medicine

Supplementary information

https://doi.org/10.1038/s41591-026-04518-z

An anti-PMEL antibody–drug conjugate with a $G_{q/11}$ inhibitor payload in *GNAQ*/*GNA11*-mutant melanomas: a phase 1 trial

In the format provided by the authors and unedited

Supplementary Information

Supplemental Methods

Sample collection and preparation of DNA and RNA extraction

Formalin-fixed paraffin-embedded (FFPE) tumor tissue blocks were obtained and sectioned at 5 µm thickness. In each sample, a minimum tumor tissue area of 100 mm^2 was manually micro dissected to ensure sufficient nucleic acid yield. Hematoxylin and eosin–stained slides were prepared to estimate tumor content and cellularity. These slides also served as reference guides to accurately target tumor-rich regions during manual microdissection. Genomic DNA was extracted from the dissected FFPE sections according to a standard protocol optimized for downstream sequencing applications. The quantity and integrity of the extracted nucleic acids were assessed before library preparation. DNA purity was evaluated spectrophotometrically using the Nanodrop ND-1000 instrument (Thermo Fisher Scientific, Waltham, MA, USA). The DNA concentration was quantified using the Quant-iT™ PicoGreen® dsDNA Reagent Kit (Thermo Fisher Scientific) following the manufacturer's protocol. Through this procedure, we ensured accurate measurement of double-stranded DNA content to meet the input requirements for the assay.

Cell-free DNA

Plasma samples were thawed for ~15-30 min at room temperature, and any remaining cell debris was pelleted by centrifugation. The resulting supernatant was transferred to a new tube, followed by digestion with proteinase K and purification using the QIAamp Circulating Nucleic Acids purification kit (Qiagen). The construction of the next-generation sequencing (NGS) library enables the capture and sequencing of the extracted DNA. The extracted DNA was subjected to end repair, A-tailing, UMI-containing adaptor ligation, and amplification through the polymerase chain reaction (PCR) assay using

the TruSeq Nano Library Preparation kit (Illumina). The prepared NGS library was denatured and hybridized to a set of RNA baits designed to bind to fragments from specific genomic regions. In a series of washing steps, unbound material was removed, and the total amount of the captured library was increased through PCR amplification. Hybrid capture was performed using SureSelect reagents (Agilent). The captured library was pooled with other libraries, each having a unique adaptor index sequence and applied to a sequencing flow cell. The flow cell then was subjected to cluster amplification and massively parallel sequencing by synthesis using a standard approach (Illumina). Library pooling and flow cell loading were coordinated to target a median of 300 million sequencing reads per library. Sequencing data were aligned to the reference human genome (build hg38) using Burrows-Wheeler Aligner (BWA-MEM)(1) [, followed by the application of UMI-based consensus building and error suppression using the fgbio tools (2). The Genome Analysis Toolkit was used for local realignment and base quality score recalibration (3-4). The final output consisted of an error-suppressed, unique set of aligned reads suitable for variant calling and further analysis. Single-nucleotide variants (SNVs) and multi-nucleotide variants were identified with MuTect (5) and short insertion/deletion (Indel) events using Pindel (6). Focal and arm-level copy number events and tumor mutation burden were called using PureCN (7), while structural variants (SVs) including chromosomal rearrangements were called using Socrates (8). Artifacts and germline variants were filtered out using a pool of normal samples and using databases such as dbSNP and gnomAD, respectively. Annotation of called variants was performed using dbSNP and COSMIC databases.

RNA-Seq

Total RNA was extracted from the samples using an extraction kit compatible with the FFPE material. The extracted RNA was assayed for quantity (RiboGreen) and for RNA fragment length (Agilent TapeStation). For the NGS library construction, the RNAseH approach was used to deplete ribosomal RNA (rRNA) from

the extracted total RNA. The rRNA-depleted sample was then fragmented, converted to cDNA, and then carried through the remaining steps of NGS library construction: end repair, A-tailing, indexed adaptor ligation, and PCR amplification using the TruSeq RNA v2 Library Preparation Kit (Illumina). The captured library was pooled with other libraries, each having a unique adaptor index sequence, and applied to a sequencing flow cell. The flow cell then underwent cluster amplification and massively parallel sequencing by synthesis using a standard approach (Illumina). Library pooling and flow cell loading were coordinated to target a median of 50 million sequencing reads per library. Sequence data were aligned to the reference human genome (build hg19) using STAR (9). Mapped reads were assembled into transcripts, and transcript abundances were estimated and normalized using Cufflinks (10). Finally, HTSeq was used to count the number of reads mapped to each gene (11). Log counts per million data normalized to GSVA-based scoring and linear modeling were assessed using *voom* (4.8.2) and *limma* (3.66.0) in R (4.5.3; 12-15). Gene sets used in this study were collected from MSigDB using the msigdbr package (24.1.0; 16). For statistical testing, only liver samples were included because healthy tissue cells on the slide could not be excluded from the assay and their gene expression data could lead to extensive signal unrelated to the tumor gene expression. Importantly, 20 of 23 patients with known sampling location had liver samples in the data set.

TSO500

DNA libraries were prepared using the Illumina TruSight™ Oncology 500 high-throughput (TSO 500 HT) assay (Illumina, San Diego, CA, USA) according to the manufacturer's instructions for use. Briefly, 80 ng of DNA input per sample (≥6.7 ng/µL) was used for library construction. The library preparation workflow included DNA fragmentation, end repair, A-tailing, adapter ligation, and PCR amplification with unique dual indexes. Safe stopping points were incorporated at key steps to enable workflow flexibility and maintain sample integrity.

Following library amplification and cleanup, the concentration of each final library was determined using the Qubit™ dsDNA high-sensitivity assay kit (Thermo Fisher Scientific). Libraries were required to have a concentration of ≥3 ng/µL to proceed with bead-based normalization. Normalized libraries were pooled before sequencing. Pooled libraries were sequenced on an Illumina sequencing platform using a 2×100 base pair (bp) paired-end configuration. Sequencing run metrics and quality control parameters were evaluated to ensure sufficient coverage and data quality for variant detection. Sequencing data were processed using the Illumina DRAGEN™ TSO500 RUO Tissue HT v2.5.2 pipeline deployed on the Illumina Connected Analytics (ICA) platform. Briefly, FASTQ files were generated locally and synchronized to AWS cloud storage. The DRAGEN pipeline was initiated on ICA using the FASTQ files, which included automated run quality control, FASTQ validation, alignment to the hg19 human reference genome, and generation of variants and biomarker outputs. Specifically, the pipeline produced microsatellite instability status, gene amplification calls, SNV and insertion/deletion (indel) results, fusion and splice variant data, and tumor mutational burden scores.

IHC

Formalin-fixed, paraffin-embedded (FFPE) tissue blocks were sectioned at 4–5 µm and bleached prior to staining using a proprietary method that was tested to be comparable to Sigma Aldrich Melanin Bleach Kit (Cat# SHH0021). The IHC was performed using a mouse monoclonal anti PMEL (gp100) antibody clone HMB45 (Dako Systems Cat# M0634) on the Ventana Benchmark Ultra at 1:50 dilution. Tissue sections were assigned an H-Score based on percent positivity and staining intensity. Cytomembranous staining of PMEL in tumor cells was examined by a pathologist and assigned an H score of between 0 and 300.

SomaScan

Proteomic profiling on plasma samples collected from 38 subjects was performed using the SomaScan® Assay version 5 (SomaLogic, Inc., Boulder, CO), a high-throughput, aptamer-based platform designed to quantify protein abundance of 10,070 unique human proteins in biological samples. Signal measured for modified aptamers (SOMAmer) in relative fluorescence units (RFU) reflects the level of protein antigenicity in the original sample. Changes in SOMAmer signal can be interpreted as changes in protein abundance (17). Both raw and normalized data were analyzed. Normalization methods included median normalization, quantile normalization, and SomaLogic's proprietary normalization procedures (18). Open-source microarray analysis software from the R/Bioconductor consortium was used to analyze the microarray data (19). The arrayQualityMetrics package was employed for technical quality assessment of the microarray data (20). Differentially expressed SOMAmers were identified using the limma package (12).

Supplementary Tables

Supplementary Table 1. Patient disposition

	**4 mg/kg Q2W N=5 n (%)**	**8 mg/kg Q2W N=12 n (%)**	**12 mg/kg Q2W N=13 n (%)**	**16 mg/kg Q2W N=14 n (%)**	**24 mg/kg Q2W N=11 n (%)**	**12 mg/kg QW N=5 n (%)**	**16 mg/kg QW N=6 n (%)**	**All patients N=66 n (%)**
Subjects treated								
Treated	5 (100)	12 (100)	13 (100)	14 (100)	11 (100)	5 (100)	6 (100)	66 (100)
Treatment ongoing	0	2 (16.7)	2 (15.4)	5 (35.7)	1 (9.1)	2 (40.0)	2 (33.3)	14 (21.2)
Discontinued from treatment	5 (100)	10 (83.3)	11 (84.6)	9 (64.3)	10 (90.9)	3 (60.0)	4 (66.7)	52 (78.8)
Reason for								

discontinuation								
Death	0	0	1 (7.7)	1 (7.1)	0	0	0	2 (3.0)
Physician decision	0	1 (8.3)	2 (15.4)	0	0	1 (20.0)	0	4 (6.1)
Progressive disease	5 (100)	8 (66.7)	8 (61.5)	8 (57.1)	10 (90.9)	2 (40.0)	4 (66.7)	45 (68.2)
Subject decision	0	1 (8.3)	0	0	0	0	0	1 (1.5)

QW, once weekly; Q2W, every 2 weeks.

Supplementary Table 2. Summary of protocol deviations

Category	4 mg/kg Q2W N=5 n (%)	8 mg/kg Q2W N=12 n (%)	12 mg/kg Q2W N=13 n (%)	16 mg/kg Q2W N=14 n (%)	24 mg/kg Q2W N=11 n (%)	12 mg/kg QW N=5 n (%)	16 mg/kg QW N=6 n (%)	All Patients N=66 n (%)
Any protocol deviation	5 (100)	7 (58.3)	7 (53.8)	7 (50.0)	4 (36.4)	4 (80.0)	2 (33.3)	36 (54.5)
Any exclusion criteria deviation	0	1 (8.3)	0	0	0	0	0	1 (1.5)
Any inclusion criteria deviation	1 (20.0)	0	1 (7.7)	0	0	0	0	2 (3.0)
Other	4 (80.0)	7 (58.3)	6 (46.2)	6 (42.9)	4 (36.4)	3 (60.0)	2 (33.3)	32 (48.5)
Treatment deviation	0	0	1 (7.7)	3 (21.4)	0	1 (20.0)	0	5 (7.6)
Numbers (n) represent counts of subjects.								

QW, once weekly; Q2W, every 2 weeks.

Supplementary Table 3. Prior antineoplastic regimens at baseline by dose cohort

**Baseline**	**4 mg/kg Q2W N=5**	**8 mg/kg Q2W N=12**	**12 mg/kg Q2W N=13**	**16 mg/kg Q2W N=14**	**24 mg/kg Q2W N=11**	**12 mg/kg QW N=5**	**16 mg/kg QW N=6**	**All patients N=66**
Prior tebentafusp	3 (60.0)	3 (25.0)	3 (23.1)	5 (35.7)	4 (36.4)	1 (20.0)	3 (50.0)	22 (33.3)

therapy, n (%)								
Prior darovasertib therapy, n (%)	3 (60.0)	1 (8.3)	1 (7.7)	1 (7.1)	0	3 (60.0)	0	9 (13.6)
Prior darovasertib and crizotinib therapy, n (%)	0	0	1 (7.7)	1 (7.1)	0	3 (60.0)	0	5 (7.6)
Prior immune checkpoint inhibitor therapy, n (%)	4 (80.0)	9 (75.0)	11 (84.6)	9 (64.3)	9 (81.8)	1 (20.0)	5 (83.3)	48 (72.7)
Prior PD-1 blockade ±CTLA-4 blockade therapy, n (%)	4 (80.0)	9 (75.0)	10 (76.9)	9 (64.3)	9 (81.8)	1 (20.0)	4 (66.7)	46 (69.7)

CTLA-4, cytotoxic T-lymphocyte–associated protein 4; n, number; PD-1, programmed cell death protein 1; Q2W, every 2 weeks; QW, every week

Supplementary Table 4. Efficacy outcomes in uveal melanoma patients*

	4 mg/kg Q2W N=5 n (%)	8 mg/kg Q2W N=12 n (%)	12 mg/kg Q2W N=9 n (%)	16 mg/kg Q2W N=13 n (%)	24 mg/kg Q2W N=10 n (%)	12 mg/kg QW N=5 n (%)	16 mg/kg QW N=6 n (%)	All UM patients N= 60 n (%)
Number of evaluable patients	5	12	9	13	10	5	6	60
Patient with measurable disease at baseline	5 (100)	12 (100)	9 (100)	13 (100)	10 (100)	5 (100)	6 (100)	60 (100)
Patient with non-measurable disease only at baseline	0	0	0	0	0	0	0	0
Best overall response - n (%)								
Partial Response (PR)	0	2 (16.7)	2 (22.2)	5 (38.5)	0	2 (40.0)	1 (16.7)	12 (20.0)
Stable Disease (SD)	2 (40.0)	8 (66.7)	6 (66.7)	7 (53.8)	7 (70.0)	2 (40.0)	5 (83.3)	37 (61.7)
Progressive Disease (PD)	3 (60.0)	1 (8.3)	1 (11.1)	1 (7.7)	3 (30.0)	1 (20.0)	0	10 (16.7)
Not Evaluable (NE)	0	1 (8.3)	0	0	0	0	0	1 (1.7)
Overall Response Rate (ORR: CR+PR)	0	2 (16.7)	2 (22.2)	5 (38.5)	0	2 (40.0)	1 (16.7)	12 (20.0)
95% CI	(0.0,52.2)	(2.1,48.4)	(2.8,60.0)	(13.9,68.4)	(0.0,30.8)	(5.3,85.3)	(0.4,64.1)	(10.8,32.3)

	4 mg/kg Q2W N=5 n (%)	8 mg/kg Q2W N=12 n (%)	12 mg/kg Q2W N=9 n (%)	16 mg/kg Q2W N=13 n (%)	24 mg/kg Q2W N=10 n (%)	12 mg/kg QW N=5 n (%)	16 mg/kg QW N=6 n (%)	All UM patients N= 60 n (%)
Disease Control Rate (DCR: CR+PR+SD)	2 (40.0)	10 (83.3)	8 (88.9)	12 (92.3)	7 (70.0)	4 (80.0)	6 (100)	49 (81.7)
95% CI	(5.3,85.3)	(51.6,97.9)	(51.8,99.7)	(64.0,99.8)	(34.8,93.3)	(28.4,99.5)	(54.1,100.0)	(69.6,90.5)

*by local radiology review. CI, confidence interval; CR, complete response; DCR, disease control rate; NE, not evaluable; ORR, overall response rate; PD, progressive disease; PR, partial response; QW, once weekly; Q2W, every 2 weeks; SD, stable disease.

Supplementary Fig. 1. Treatment-related AEs of any grade reported in ≥5% of patients

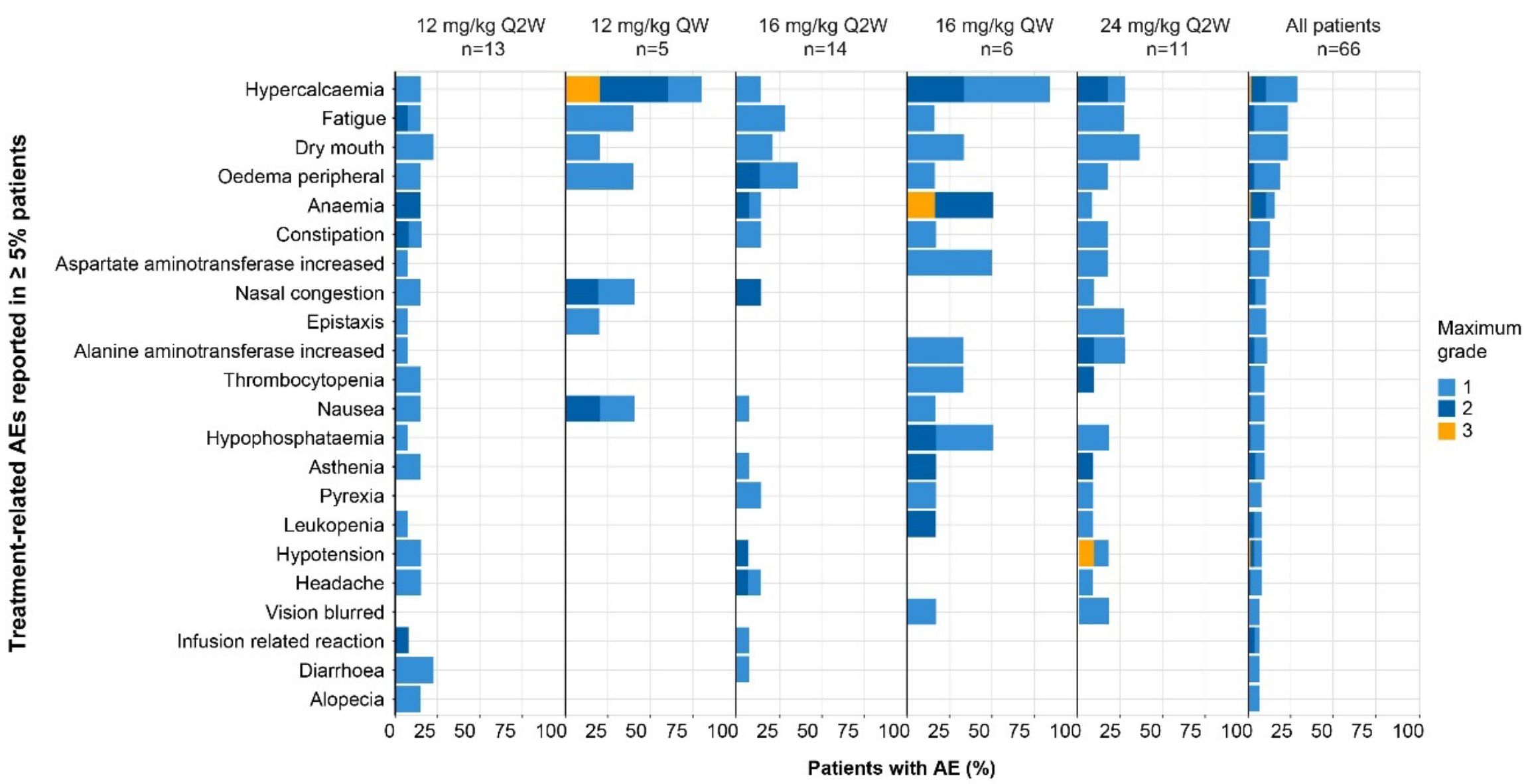

AE, adverse event; QW once weekly; Q2W, every 2 weeks.

Supplementary Fig. 2. Best percentage change in target lesion size by anatomical location

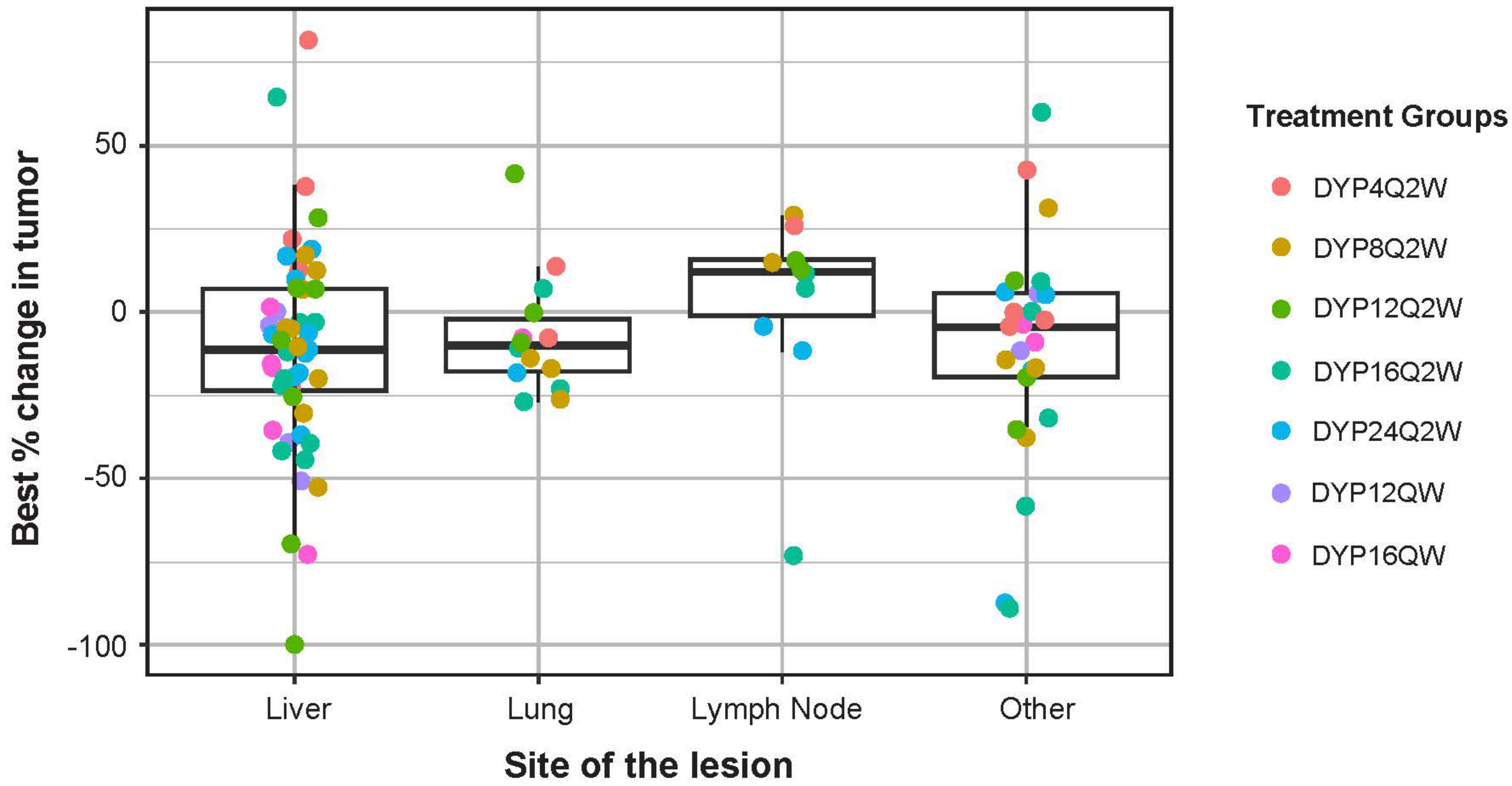

Best percent change in tumor size from baseline is shown for individual target lesions stratified by lesion location (liver, lung, lymph node, and other). Each point represents an individual lesion, colored by DYP688 dosing regimen, and boxplots summarize the distribution within each site. QW once weekly; Q2W, every 2 weeks

Supplementary References

1. Heng L. Aligning sequence reads, clone sequences and assembly contigs with BWA-MEM. https://arxiv.org/abs/1303.3997 (2013)
2. http://fulcrumgenomics.github.io/fgbio/. Accessed 10 Oct 2025.
3. McKenna, A. et al. The genome analysis toolkit: a MapReduce framework for analyzing next-generation DNA sequencing data. *Genome Res.* **20**, 1297–1303 (2010).
4. DePristo, M. A. et al. A framework for variation discovery and genotyping using next-generation DNA sequencing data. *Nat. Genet.* **43**, 491–498 (2011).
5. Cibulskis, K. et al. Sensitive detection of somatic point mutations in impure and heterogeneous cancer samples. *Nat. Biotechnol.* **31**, 213–219 (2013).

6. Ye, K., Schulz, M. H., Long, Q., Apweiler, R. & Ning, Z. Pindel: a pattern growth approach to detect break points of large deletions and medium sized insertions from paired-end short reads. *Bioinformatics*. **25**, 2865–2871 (2009).
7. Riester M, et al. PureCN: copy number calling and SNV classification using targeted short read sequencing. *Source Code Biol Med*. **11**, 13 (2016).
8. Jan Schröder J et al., Socrates: identification of genomic rearrangements in tumour genomes by re-aligning soft clipped reads, *Bioinformatics.* **30**, 1064–1072 (2014).
9. Dobin, A. et al. STAR: ultrafast universal RNA-seq aligner. *Bioinformatics*. **29**, 15-21 (2012).
10. Trapnell, C. et al. Differential gene and transcript expression analysis of RNA-seq experiments with TopHat and Cufflinks. *Nat. Protoc.* **7**, 562–578 (2012).
11. Anders, S., Pyl, P. T. & Huber, W. HTSeq--a Python framework to work with high-throughput sequencing data. *Bioinformatics*. **31**, 166–169 (2015).
12. Ritchie, M. E. et al. limma powers differential expression analyses for RNA-sequencing and microarray studies. *Nucleic Acids Res.* **43**, e47 (2015).
13. Phipson, B., Lee, S., Majewski, I. J., Alexander, W. S. & Smyth, G. K. Robust hyperparameter estimation protects against hypervariable genes and improves power to detect differential expression. *Ann. Appl. Stat.* **10**, 946–963 (2016).
14. Law, C. W., Chen, Y., Shi, W. & Smyth, G. K. voom: Precision weights unlock linear model analysis tools for RNA-seq read counts. *Genome Biol.* **15**, R29 (2014).
15. Hanzelmann, S., Castelo, R. & Guinney, J. GSVA: gene set variation analysis for microarray and RNA-seq data. *BMC Bioinformatics* **14**, 7 (2013).
16. Dolgalev, I. msigdbr: MSigDB gene sets for multiple organisms in a tidy data format. CRAN. https://doi.org/10.32614/CRAN.package.msigdbr
17. Emilsson, V. *et al.* Co-regulatory networks of human serum proteins link genetics to disease. *Science* **361**, 769–773 (2018).
18. SomaScan Data Standardization. SomaLogic, CO, USA (2017). https://somalogic.com/wp-content/uploads/2017/06/SSM-071-Rev-0-Technical-Note-SOMAscan-Data-Standardization.pdf
19. Bioconductor. http://www.bioconductor.org (2019).
20. Kauffmann, A., Gentleman, R. & Huber, W. arrayQualityMetrics – a Bioconductor package for quality assessment of microarray data. *Bioinformatics* **25**, 415–416 (2009).

NOVARTIS

Research and Development

DYP688

Clinical Trial Protocol CDYP688A12101

A Phase I/II, multi-center, open label study of DYP688 in patients with metastatic uveal melanoma (MUM) and other GNAQ/11 mutant melanomas

Clinical Trial Protocol Number: CDYP688A12101

Version Number: v07 (Clean)

Compound: DYP688

Brief Title: A study to investigate DYP688 in patients with metastatic uveal melanoma and other non-uveal melanomas

Study Phase: I/II

Sponsor Name and address: Novartis Pharma AG, Lichtstrasse 35, 4056 Basel town, Switzerland or its affiliates outside of the EEA (where applicable)

European Clinical Trials Database (EU CT) number: 2023-509451-14

Content final Date: 18-Apr-2025

Property of Novartis
Confidential
May not be used, divulged, published, or otherwise disclosed
without the consent of Novartis
Clinical Trial Protocol Template Version 4.0 dated 15-Feb-2021

Table of contents

Table of contents 2
List of tables 6
List of figures 8
List of abbreviations 9
Glossary of terms 11
Amendment 07 (18-Apr-2025) 14
Amendment 06 (18-Dec-2024) 15
Amendment 05 (13-Feb-2024) 17
Amendment 04 (27-Jun-2023) 21
Amendment 03 (13-Dec-2022) 22
Amendment 01-DE.01 (27-September-2022) 24
Amendment 02 (10-Aug-2022) 25
Amendment 01 (02-Jun-2022) 28
Protocol summary 30
1 Introduction 34
1.1 Background 34
1.1.1 Overview of DYP688 35
1.2 Purpose 41
2 Objectives and endpoints 42
2.1 Primary estimands 43
2.2 Secondary estimands 44
3 Study design 44
3.1 Phase I – Dose Escalation 45
3.2 Phase II 45
4 Rationale 46
4.1 Rationale for study design 46
4.2 Rationale for dose/regimen and duration of treatment 47
4.3 Purpose and timing of interim analyses/design adaptations 49
4.4 Risks and benefits 49
4.5 Rationale for public health emergency mitigation procedures 50
5 Study Population 50
5.1 Inclusion criteria 50
5.2 Exclusion criteria 51
6 Treatment 55
6.1 Description of study treatment 55

6.1.1 Investigational drug ... 55
6.1.2 Additional study treatments ... 56
6.1.3 Treatment duration ... 56
6.2 Other treatment(s) ... 57
6.2.1 Concomitant therapy ... 57
6.2.2 Prohibited medication ... 59
6.3 Preparation, handling, storage and accountability ... 59
6.3.1 Handling of study treatment ... 59
6.4 Patient numbering, treatment assignment, randomization ... 60
6.4.1 Patient numbering ... 60
6.4.2 Treatment assignment, randomization ... 60
6.5 Treatment blinding ... 61
6.6 Dose escalation and dose modification ... 61
6.6.1 Dose escalation guidelines ... 61
6.6.2 Guidelines for dose escalation and determination of MTD/RD ... 61
6.6.3 Definitions of dose limiting toxicities (DLTs) ... 64
6.6.4 Dose modifications ... 66
6.6.5 Follow-up for toxicities ... 73
6.7 Additional treatment guidance ... 74
6.7.1 Treatment compliance ... 74
7 Informed consent procedures ... 74
8 Visit schedule and assessments ... 76
8.1 Screening ... 86
8.1.1 Information to be collected on screening failures ... 86
8.2 Patient demographics/other baseline characteristics ... 86
8.3 Efficacy ... 87
8.3.1 Tumor Imaging ... 87
8.3.2 Overall survival ... 89
8.3.3 Appropriateness of efficacy assessments ... 89
8.4 Safety/Tolerability ... 89
8.4.1 Laboratory evaluations ... 91
8.4.2 Electrocardiogram (ECG) ... 92
8.4.3 Pregnancy and assessments of fertility ... 93
8.4.4 Ophthalmological assessments ... 94
8.4.5 Appropriateness of safety measurements ... 95
8.5 Additional assessments ... 95

8.5.1 Pharmacokinetics and Immunogenicity 95
8.5.2 Biomarkers 104
8.6 Optional additional research 106
9 Discontinuation and completion 106
9.1 Discontinuation 106
9.1.1 Discontinuation from study treatment 106
9.1.2 Discontinuation from study 107
9.1.3 Lost to follow-up 107
9.2 Withdrawal of informed consent/Opposition to use data/biological samples 108
9.3 Study completion and post-study treatment 108
9.3.1 End of Study 109
9.3.2 Post End of Treatment Safety Follow-up 109
9.3.3 Disease progression follow-up 109
9.3.4 Survival follow-up 109
9.4 Early study termination by the sponsor 110
9.5 Study stopping rules 110
10 Safety monitoring, reporting and committees 111
10.1 Definition of adverse events and reporting requirements 111
10.1.1 Adverse events 111
10.1.2 Serious adverse events 112
10.1.3 SAE reporting 113
10.1.4 Pregnancy reporting 115
10.1.5 Reporting of study treatment errors, misuse/abuse, and overdose 115
10.2 Additional Safety Monitoring 116
10.3 Committees 116
10.3.1 Data Monitoring Committee 116
11 Data Collection and Database management 116
11.1 Data collection 116
11.2 Database management and quality control 117
11.3 Site monitoring 117
11.4 Data protection 118
12 Data analysis and statistical methods 119
12.1 Analysis sets 120
12.1.1 Full Analysis Set (FAS) and Safety Set (SS) 120
12.1.2 Dose-determining analysis set 120
12.1.3 Pharmacokinetic Analysis Set (PAS) 120

12.1.4 Immunogenicity (IG) analysis Set ... 121
12.2 Patient demographics and other baseline characteristic ... 121
12.3 Treatments ... 121
12.4 Analysis supporting primary objectives ... 121
12.4.1 Safety endpoints ... 122
12.4.2 Efficacy endpoints ... 125
12.5 Analysis supporting secondary objectives ... 127
12.5.1 Efficacy and/or Pharmacodynamic endpoint(s) ... 127
12.5.2 Safety endpoints ... 128
12.5.3 Pharmacokinetics ... 128
12.6 Analysis of exploratory endpoints ... 129
12.6.1 [redacted] 129
12.6.2 [redacted] 129
12.7 Interim analyses ... 130
12.8 [redacted] 132
12.8.1. [redacted] 132
13 Ethical considerations and administrative ... 133
13.1 Regulatory and ethical compliance ... 133
13.2 Responsibilities of the investigator and IRB/IEC ... 134
13.3 Publication of study protocol and results ... 134
13.4 Quality Control and Quality Assurance ... 135
13.5 Data quality assurance ... 135
13.6 Patient Engagement ... 135
14 Protocol adherence ... 135
14.1 Protocol amendments ... 136
15 References (available upon request) ... 137
16 Appendices ... 140
16.1 Appendix 1: Guidelines for Response, Duration of Overall Response, TTF, TTP, Progression-Free Survival, and Overall Survival (based on RECIST 1.1) ... 140
16.1.1 Introduction ... 140
16.1.2 Efficacy assessments ... 140
16.1.3 Definitions ... 140
16.1.4 Methods of tumor measurement - general guidelines ... 141
16.1.5 Baseline documentation of target and non-target lesions ... 143
16.1.6 Follow-up evaluation of target and non-target lesions ... 144
16.1.7 Determination of target lesion response ... 145

16.1.8 Determination of non-target lesion response 147
16.1.9 New lesions 148
16.1.10 Evaluation of overall lesion response 149
16.1.11 Efficacy definitions 149
16.1.12 Time to event variables 152
16.1.13 Data handling and programming rules 160
16.1.14 References (available upon request) 164
16.2 Appendix 2: Statistical details of the Bayesian logistic regression model (BLRM): priors, design, operating characteristics and hypothetical data scenarios 165
16.2.1 Statistical considerations for DYP688 single agent 165
16.3 Creatinine clearance.. 170
16.4 Drugs that are to be used with caution.. 171

List of tables

Table 1-1 Epidemiology of G (alpha) subunit mutations in melanocytic neoplasms 35
Table 2-1 Objectives and related endpoints 42
Table 4-1 DYP688 tmAb and CCI [redacted] exposure multiples in cynomolgus monkey relative to the predicted human exposure at the starting dose (4 mg/kg) and predicted efficacious dose range (2-15 mg/kg) 48
Table 6-1 Investigational drugs 55
Table 6-2 Provisional dose levels for DYP688 single agent 61
Table 6-3 Criteria for defining dose-limiting toxicities in all patients 64
Table 6-4 Criteria for dose reduction / interruption and re-initiation of DYP688 treatment for adverse drug reactions 67
Table 8-1 Windows for Visit Assessments 77
Table 8-2 Assessment schedule (Q2W and Q4W dosing schedule) 78
Table 8-3 Assessment schedule (QW dosing schedule) 82
Table 8-4 Imaging Assessment Collection Plan 87
Table 8-5 Physical assessments 90
Table 8-6 ECOG Performance Status 90
Table 8-7 Karnofsky/Lansky Performance Status (Adolescent Population only) 91
Table 8-8 Laboratory assessments 92
Table 8-9 ECG collection plan for all patients enrolled in Dose escalation and Phase II 93
Table 8-10 Ophthalmologic monitoring 95

Table 8-11	Pharmacokinetic and Immunogenicity blood collection log for DYP688 single agent treatment in dose escalation (Q2W dosing schedule)[1,2]	96
Table 8-12	Pharmacokinetic and Immunogenicity blood collection log for DYP688 single agent treatment in Phase II (Q2W dosing schedule) [1,2]	97
Table 8-13	Pharmacokinetic and Immunogenicity blood collection log for DYP688 single agent treatment in dose escalation (Q4W dosing schedule) [1,2]	98
Table 8-14	Pharmacokinetic and Immunogenicity blood collection log for DYP688 single agent treatment in Phase II (Q4W dosing schedule) [1,2]	100
Table 8-15	Pharmacokinetic and Immunogenicity blood collection log for DYP688 single agent treatment in dose escalation (QW dosing schedule) [1,2]	101
Table 8-16	Pharmacokinetic and Immunogenicity blood collection log for DYP688 single agent treatment in Phase II (QW dosing schedule) [1,2]	102
Table 8-17	Biomarker sample collection plan	104
Table 12-1	Minimum exposure criteria for potential dosing regimen	120
Table 12-2	Non-compartmental pharmacokinetic parameters	129
Table 12-3	Predictive probability of study success under different IA results (N = 40 at primary analysis)	131
Table 12-4	Operating characteristics for one treatment group – Interim analysis (15 patients), primary analysis (40 patients)	132
Table 12-5	CCI [redacted]	133
Table 16-1	Response criteria for target lesions	145
Table 16-2	Response criteria for non-target lesions	147
Table 16-3	Overall lesion response at each assessment	149
Table 16-4	Overall lesion response at each assessment: patients with non-target disease only	157
Table 16-5	Options for event dates used in PFS, TTP, duration of response	158
Table 16-6	Prior distribution parameters-DYP688 SA (Q2W regimen)	166
Table 16-7	Summary of prior distribution of DLT rates- DYP688 SA (Q2W regimen)	166
Table 16-8	Hypothetical scenarios	166
Table 16-9	True probability of DLT for simulation scenarios	168
Table 16-10	Summary of simulation results	169
Table 16-11	List of medication that should be used with caution	171

List of figures

Figure 1-1 Efficacy of DYP688 single dose i.v. in the 92.1 xenograft mouse model....36
Figure 1-2 Mean exposure profiles of tmAb, tADCa and tADCi after single i.v. dose of 3 mg/kg in the cynomolgus monkey....38
Figure 3-1 Study design....46
Figure 3-2 Study Visit Flow....46

List of abbreviations

ADC	Antibody drug conjugate
AE	Adverse Event
ALP	Alkaline Phosphatase
ALT	Alanine Aminotransferase
AST	Aspartate Aminotransferase
AxMP	Auxiliary Medicinal Product
BLRM	Bayesian Logistic Regression Model
BHLRM	Bayesian Hierarchical Logistic Regression Model
BUN	Blood Urea Nitrogen
cfDNA	Circulating free DNA
CK	Creatine Kinase
CQA	Clinical Quality Assurance
CRF	Case Report/Record Form (paper or electronic)
CSR	Clinical study report
CSTDs	Closed system transfer device(s)
CTC	Common Terminology Criteria
CTIS	Clinical Trials Information System
DLT	Dose Limiting Toxicity
DMC	Data Monitoring Committee
ECG	Electrocardiogram
EDC	Electronic Data Capture
eSAE	Electronic Serious Adverse Event
eSource	Electronic Source
EU-CTR	European Union Clinical Trial Regulation
FAS	Full analysis set
FIH	First in Human
GCP	Good Clinical Practice
GCS	Global Clinical Supply
h	Hour
HBV	Hepatitis B Virus
HCV	Hepatitis C Virus
HNSTD	Highest Non-Severely Toxic Dose
i.v.	intravenous
IA	Interim analysis
IB	Investigator’s Brochure
ICF	Informed Consent Form
IEC	Independent Ethics Committee
IMP	Investigational Medicinal Product
IN	Investigator Notification
INR	International Normalized Ratio

IOP	Intraocular pressure
IRB	Institutional Review Board
IRR	Infusion-related reaction
IRT	Interactive Response Technology
LDH	lactate dehydrogenase
LLOQ	lower limit of quantification
MedDRA	Medical dictionary for regulatory activities
mg	milligram(s)
mL	milliliter(s)
mOS	Median overall survival
MTD	Maximum Tolerated Dose
MUM	Metastatic uveal melanoma
PD	Pharmacodynamic(s)
PK	Pharmacokinetic(s)
PT	prothrombin time
QTcF	QT interval corrected by Fridericia's formula
RD	Recommended Dose
RECIST	Response Evaluation Criteria In Solid Tumors
SAE	Serious Adverse Event
SAP	Statistical Analysis Plan
SD	standard deviation
SUSAR	Suspected Unexpected Serious Adverse Reaction
TrAEs	Treatment-related adverse events
WHO	World Health Organization
WoC	Withdrawal of Consent

Glossary of terms

Additional treatment	Medicinal products that may be used during the clinical trial as described in the protocol, but not as an investigational medicinal product (e.g. any background therapy)
Assessment	A procedure used to generate data required by the study
Auxiliary Medicinal Product	Medicinal product used for the needs of a clinical trial as described in the protocol, but not as an investigational medicinal product (e.g., rescue medication, challenge agents, background treatment or medicinal products used to assess endpoints in the clinical trial). Concomitant therapy is not considered as AxMP.
Biologic Samples	A biological specimen including, for example, blood (plasma, serum), saliva, tissue, urine, stool, etc. taken from a study patient
Coded Data	Personal Data which has been de-identified by the investigative center team by replacing personal identifiers with a code.
Cohort	A group of individuals who share a common exposure, experience or characteristic, or a group of individuals followed-up or traced over time
Cycles	Number and timing or recommended repetitions of therapy are usually expressed as number of days (e.g., 28 days)
Discontinuation from study	Point/time when the patient permanently stops receiving the study treatment and further protocol required assessments or follow-up, for any reason. No specific request is made to stop the use of their samples or data.
Discontinuation from study treatment	Point/time when the patient permanently stops receiving the study treatment for any reason (prior to the planned completion of study drug administration, if any). Patient agrees to the other protocol required assessments including follow-up. No specific request is made to stop the use of their samples or data.
Dosage	Dose of the study treatment given to the patient in a time unit (e.g. 100 mg once a day, 75 mg twice a day)
Electronic Data Capture (EDC)	Electronic data capture (EDC) is the electronic acquisition of clinical study data using data collection systems, such as Web-based applications, interactive voice response systems and clinical laboratory interfaces. EDC includes the use of Electronic Case Report Forms (eCRFs) which are used to capture data transcribed from source data/documents used at the point of care
End of the clinical trial/study	The end of the clinical trial is defined as the last visit of the last patient
Enrollment	Point/time of patient entry into the study at which informed consent must be obtained. The action of enrolling one or more patients
eSource (DDE)	eSource Direct Data Entry (DDE) refers to the capture of clinical study data electronically, at the point of care. eSource Platform/Applications combines source documents and case report forms (eCRFs) into one application, allowing for the real time collection of clinical trial information to sponsors and other oversight authorities, as appropriate
Estimand	As defined in the ICH E9 (R1) addendum, estimand is a precise description of the treatment effect reflecting the clinical question posed by the trial objective. It summarizes at a population-level what the outcomes would be in the same patients under different treatment conditions being compared. Attributes of an estimand include the population, variable (or endpoint) and treatment of interest, as well as

	the specification of how the remaining intercurrent events are addressed and a population-level summary for the variable.
Intercurrent events	Events occurring after treatment initiation that affect either the interpretation or the existence of the measurements associated with the clinical question of interest.
Investigational drug/ treatment	The drug whose properties are being tested in the study
Other treatment	Treatment that may be needed/allowed during the conduct of the study (i.e. concomitant or rescue therapy)
Part	A sub-division of a study used to evaluate specific objectives or contain different populations. For example, one study could contain a single dose part and a multiple dose part, or a part in patients with established disease and in those with newly-diagnosed disease
Patient	A trial patient (can be a healthy volunteer or a patient). "Patient" terminology is used in the protocol whereas term "Subject" is used in data collection
Patient number	A unique number assigned to each patient upon signing the informed consent. This number is the definitive, unique identifier for the patient and should be used to identify the patient throughout the study for all data collected, sample labels, etc.
Period	The subdivisions of the trial design (e.g. Screening, Treatment, Follow-up) which are described in the Protocol. Periods define the study phases and will be used in clinical trial database setup and eventually in analysis
Personal data	Patient information collected by the Investigator that is coded and transferred to Novartis for the purpose of the clinical trial. This data includes patient identifier information, study information and biological samples.
Randomization	The process of assigning trial patients to investigational drug or control/comparator drug using an element of chance to determine the assignments in order to reduce bias.
Re-screening	If a patient fails the initial screening and is considered as a Screen Failure, he/she can be invited once for a new Screening visit after medical judgment and as specified by the protocol
Remote	Describes any trial activities performed at a location that is not the investigative site where the investigator will conduct the trial, but is for example a home or another appropriate location
Screen Failure	A patient who did not meet one or more criteria that were required for participation in the study
Source Data/Document	Source data refers to the initial record, document, or primary location from where data comes. The data source can be a database, a dataset, a spreadsheet or even hard-coded data, such as paper or eSource
Start of the clinical trial	The start of the clinical trial is defined as the signature of the informed consent by the first patient<br>Per EU Regulation 536/2014 the start of a clinical trial means the first act of recruitment of a potential patient for a specific clinical trial, unless defined differently in the protocol.
Study treatment	Any drug or combination of drugs or intervention administered to the study patients as part of the required study procedures; includes investigational drug(s), control(s) or background therapy

Tele-visit	Procedures or communications conducted using technology such as telephone or video-conference, whereby the patient is not at the investigative site where the investigator will conduct the trial.
Treatment arm/group	A treatment arm/group defines the dose and regimen or the combination, and may consist of 1 or more cohorts.
Variable (or endpoint)	The variable (or endpoint) to be obtained for each patient that is required to address the clinical question. The specification of the variable might include whether the patient experiences an intercurrent event.
Withdrawal of study consent (WoC) / Opposition to use of data /biological samples	Withdrawal of consent from the study occurs when the patient explicitly requests to stop use of their data and biological samples (opposition to use data and biological samples) AND no longer wishes to receive study treatment, AND does not agree to further protocol required assessments. This request should be in writing (depending on local regulations) and recorded in the source documentation.<br>Opposition to use data/biological samples occurs in the countries where collection and processing of personal data is justified by a different legal reason than consent.

Amendment 07 (18-Apr-2025)

Amendment rationale

The purpose of this protocol amendment is to address [redacted] to update the exclusion criteria and the pregnancy and assessment of fertility section to further clarify the definition of women of childbearing potential (WOCBP) and postmenopausal women as well as to correct the methods of highly effective of contraception in the exclusion criteria.

Study Status

At the time of the release of this amendment, the study is ongoing in the dose escalation part. As of 18-Apr-2025, 66 patients have been treated with DYP688.

Changes to the protocol

[redacted]

IRBs/IECs

A copy of this amended protocol will be sent to the Institutional Review Board (IRBs)/Independent Ethics Committee (IECs) and Health Authorities.

The changes described in this amended protocol require IRB/IEC approval prior to implementation.

Amendment 06 (18-Dec-2024)

Amendment rationale

The purpose of this protocol amendment is to:

1. Align the protocol with the EU-CTR requirements.
2. Change the frequency of assessments to lessen the burden for long-term ongoing patients whilst maintaining sufficient safety and efficacy monitoring as per Institutional guidelines. As of 25-Oct-2024, DYP688 continues to be safe and tolerable with a median duration of treatment of 7 months with 95% CI (4.7, 8.8).
3. Allow patients to switch to a less frequent dosing schedule either due to toxicity on the more frequent dosing schedule, or due to patient convenience to reduce the burden of study visits after completing 4 cycles of study treatment. Based on the data generated to date which suggests more frequent dosing does not lead to greater clinical benefit, the QW schedule will not be pursued further, therefore, to reduce the burden of study visits, patients on the QW schedule will be allowed to switch to the Q2W schedule at a lower or the same dose level, after documented discussion with Novartis.

Study Status

At the time of the release of this amendment, the study is ongoing in the dose escalation part. As of 25-Oct-2024, 66 patients have been treated with DYP688.

Changes to the protocol

[redacted]

[redacted]

IRBs/IECs

A copy of this amended protocol will be sent to the Institutional Review Board (IRBs)/Independent Ethics Committee (IECs) and Health Authorities.

The changes described in this amended protocol require IRB/IEC approval prior to implementation.

The changes herein affect the Informed Consent. Sites are required to update and submit for approval a revised informed consent that takes into account the changes described in this protocol amendment.

Amendment 05 (13-Feb-2024)

Amendment rationale

The purpose of this protocol amendment is:

1. To update the contraception use, condom use, breastfeeding, and sperm donation requirements for patients treated with DYP688 to align with the current version of the DYP688 Investigator’s Brochure (Edition 2, dated 05-Jul-2023). Based on data from the completed GLP compliant Ames study and Novartis internal guidance on the prevention of pregnancy, the requirement for sexually active male patients being treated with DYP688 to use a condom during sexual intercourse, has been reduced from 7 months to 60 days (five half-lives of DYP688) after the last dose of DYP688. This update also applies to sperm donation, other contraception use, and breastfeeding. For more information, please refer to the [DYP688 Investigator’s Brochure].
2. [redacted]
3. To address a request from a health authority, the starting dose requirements to explore alternative dosing schedules to the Q2W schedule (QW and/or Q4W) have been added. Although the current protocol permits testing alternative dosing schedules, this clarification was added to address the health authority request.

Additional changes include:

[redacted]

- [redacted]

Study Status

At the time of the release of this amendment, the study is ongoing in the dose escalation part. As of 01-Jan-2024, 42 patients were treated with DYP688. The maximum tolerated dose (MTD) and/or recommended dose (RD) has not been determined.

Changes to the protocol

[redacted]

CCI

[redacted]

CCI

[redacted]

IRBs/IECs

A copy of this amended protocol will be sent to the Institutional Review Board (IRBs)/Independent Ethics Committee (IECs) and Health Authorities.

The changes described in this amended protocol require IRB/IEC approval prior to implementation.

The changes herein affect the Informed Consent. Sites are required to update and submit for approval a revised informed consent that takes into account the changes described in this protocol amendment.

Amendment 04 (27-Jun-2023)

Amendment rationale

The purpose of this protocol amendment is to address a Health Authority (HA) request regarding the provisional dose levels to be tested in this study. Although the current protocol permits additional and/or intermediate dose levels below the MTD to be investigated during the course of the study, Table 6-2 has been updated to include a higher provisional dose level as per the HA request. The addition of the provisional dose level of 24 mg/kg Q2W is supported by the safety and tolerability data observed in the clinic to date and recommendations of the BLRM. In total, 37 patients have been treated across doses ranging from 4 to 16 mg/kg Q2W. No DLTs have been observed in DLT-evaluable patients at any dose level tested. Based upon these data, the BLRM along with EWOC and dose increment limitations specified in the protocol, doses up to 32 mg/kg Q2W are allowed to be tested. However, due to limitations in the current study drug formulation, only doses up to 24 mg/kg Q2W may be explored.

Minor clarifications and typographical errors have also been updated throughout the document.

Study Status

This study is ongoing in the dose escalation part. As of the date of this amendment, 37 patients have been treated with DYP688. The recommended dose (RD) or the maximum tolerated dose (MTD) has not been declared.

Changes to the protocol

[redacted]

IRBs/IECs

A copy of this amended protocol will be sent to an Independent Ethics Committee (IEC) and Health Authority.

The changes described in this amended protocol require IEC approval prior to implementation.

Amendment 03 (13-Dec-2022)

Amendment rationale

The primary purpose of this protocol amendment is to address health authority requests to:

- Restrict enrollment to adult patients ≥18 years of age in countries where required by local Health Authority.
- Increase the safety monitoring interval for the first dose between patients treated within each dose escalation cohort. The interval will be increased from 24 hours for the first 3 patients of each dose escalation cohort, to 48 hours for all patients in each dose escalation cohort, where required by local health authority.

Other changes incorporated into this amendment are as follows:

[redacted]

Study Status

This study is open for enrollment and as of 09-Dec-2022, eleven patients have been treated in the dose escalation part of the study.

Changes to the protocol

[redacted]

[redacted]

IRBs/IECs

A copy of this amended protocol will be sent to the Institutional Review Board (IRBs)/Independent Ethics Committee (IECs) and Health Authorities.

The changes described in this amended protocol require IRB/IEC approval prior to implementation.

The changes herein affect the Informed Consent. Sites are required to update and submit for approval a revised Informed Consent that takes into account the changes described in this protocol amendment.

Amendment 01-DE.01 (27-September-2022)

Amendment rationale

The protocol has been amended locally to address feedback from the German Health Authority, Paul-Ehrlich-Institut (PEI), regarding the study objectives, exclusion criteria, patient monitoring post infusion, and end of study definition.

Study Status

At the time of release of this amendment, the study has not been opened for recruitment in Germany.

Changes to the protocol

[redacted]

IRBs/IECs

A copy of this amended protocol will be sent to an Independent Ethics Committee (IEC) and Health Authority.

The changes described in this amended protocol require IEC approval prior to implementation.

Amendment 02 (10-Aug-2022)

Amendment rationale

The purpose of this amendment is to address Health Authority requests to include study stopping rules, clarify eligibility criteria, clarify DLT criteria, include later ophthalmologic monitoring and limit blood collection in adolescent patients where restricted by local law.

Study Status

As of 05-Aug-2022, one patient has been treated in the Phase I, dose escalation part of the study.

Changes to the protocol

CCI [redacted]

CCI [redacted]

IRBs/IECs

A copy of this amended protocol will be sent to the Institutional Review Board (IRBs)/Independent Ethics Committee (IECs) and Health Authorities.

The changes described in this amended protocol require IRB/IEC approval prior to implementation.

The changes herein affect the Informed Consent. Sites are required to update and submit for approval a revised Informed Consent that takes into account the changes described in this protocol amendment.

Amendment 01 (02-Jun-2022)

Amendment rationale

The purpose of this protocol amendment is to address Health Authority requests regarding the inclusion/exclusion criteria, treatment beyond progression criteria, dose limiting toxicities and dose modification tables.

Study Status

At the time of release of this amendment, the study has not been opened for recruitment.

Changes to the protocol

[redacted]

IRBs/IECs

A copy of this amended protocol will be sent to the Institutional Review Board (IRBs)/Independent Ethics Committee (IECs) and Health Authorities.

The changes described in this amended protocol require IRB/IEC approval prior to implementation.

The changes herein affect the Informed Consent. Sites are required to update and submit for approval a revised Informed Consent that takes into account the changes described in this protocol amendment.

Protocol summary

**Brief title**	A Phase I/II study of DYP688 in patients with metastatic uveal melanoma (MUM) and other GNAQ/11 mutant melanomas
**Sponsor and Clinical Phase**	Novartis
**Investigation type**	Drug
**Study type**	Interventional
**Purpose**	The purpose of this Phase I/II, FIH study is to characterize the safety, tolerability, and anti-tumor activity of DYP688 as a single agent in patients with MUM and potentially other melanomas harboring GNAQ/11 mutations.
**Primary Objective(s)**	Phase I: To characterize the safety and tolerability and to identify the MTD and/or RD and regimen for future studies of DYP688 as a single agent<br>Phase II: To evaluate the anti-tumor activity of DYP688 as a single agent
**Secondary Objectives**	Phase I<br>• To characterize the pharmacokinetics (PK) of DYP688 as a single agent<br>• To assess the immunogenicity (IG) of DYP688 as a single agent<br>• To evaluate the preliminary anti-tumor activity of DYP688 as a single agent<br>Phase II<br>• To further evaluate the anti-tumor activity of DYP688 as a single agent<br>• To evaluate the effects of DYP688 as a single agent on overall survival<br>• To further characterize the safety and tolerability of DYP688 as a single agent<br>• To further characterize the PK of DYP688 as a single agent<br>• To further characterize the IG of DYP688 as a single agent
**Study design**	This is a FIH, phase I/II, open label, multi-center, single arm study. Phase I will characterize the safety and tolerability of DYP688 in adult patients with MUM and other GNAQ/11 mutant melanomas. After the determination of the maximum tolerated dose (MTD)/recommended dose (RD) whichever occurs first, for DYP688, phase II will access the anti-tumor activity and further access the safety, tolerability, and PK/PD at the MTD/RD.
**Rationale**	High unmet medical need remains for patients with uveal melanoma and non-uveal GNAQ/11 mutant melanomas. DYP688 is an ADC that first targets PMEL17, a melanocyte lineage protein that is highly expressed in primary and metastatic cutaneous and uveal melanoma. Upon ADC binding to PMEL17 on the target cells and internalization, the linker is cleaved to release the Gq/11 inhibitor SDZ475, which inhibits Gq/11 oncogenic signaling, resulting in dose-dependent apoptosis.

**Study population**	This study will be conducted in adult patients with MUM and other non-uveal, GNAQ/11 mutant melanomas. Adolescent patients may be eligible for enrollment in the Phase II part of the study, once the initial safety profile and pharmacologically active dose have been identified in adults. In countries where required by the local health authority, enrollment will be restricted to adult patients ≥ 18 years of age.
**Key Inclusion criteria**	1. Patients in the dose escalation part must be ≥ 18 years of age at the time of informed consent (ICF) signature. In the phase II part, patients ≥ 12 years of age at the time of informed consent may be eligible for enrollment (not applicable in countries where enrollment is restricted by the local health authority to patients ≥ 18 years of age). Patients must have a minimum weight of 40 kg. For the 32 mg/kg dose level a maximum weight limit of 115 kg may not be exceeded.<br>2. ECOG performance status ≤ 1 for patients ≥ 18 years of age; Karnofsky performance status ≥ 70 for patients ≥ 16 and < 18 years of age; Lansky performance status ≥ 70 for patients ≥ 12 and < 16 years of age<br>3. Patients must be suitable and willing to undergo study required biopsies according to the treating institution's own guidelines and requirements, if medically feasible.<br>**For all patients in Dose Escalation**<br>4. MUM: uveal melanoma with histologically or cytologically confirmed metastatic disease. Patient must be either treatment naive or have received any number of prior lines and progressed on most recent therapy<br>5. Non-MUM: advanced cutaneous or mucosal melanoma with histologically or cytologically confirmed metastatic disease that has progressed following all standard therapies or that has no satisfactory alternative therapies and has evidence of GNAQ/11 mutation based on local data<br>**For patients in Phase II**<br>6. Tebentafusp naïve group: Diagnosis of uveal melanoma with histologically or cytologically confirmed metastatic disease that has progressed following standard therapies or that has no satisfactory alternative therapies<br>7. Tebentafusp pre-treated group: Diagnosis of uveal melanoma with histologically or cytologically confirmed metastatic disease. Patients must be previously treated with tebentafusp and have progressed<br>8. Non-MUM: patients with diagnosis of cutaneous or mucosal melanomas harboring GNAQ/11 mutations based on local data, with histologically or cytologically confirmed metastatic disease that has progressed following all standard therapies or that has no satisfactory alternative therapies
**Key Exclusion criteria**	1. Malignant disease, other than that being treated in this study.<br>2. Active brain metastases, i.e. symptomatic brain metastases or known leptomeningeal disease.

	3. Evidence of active bleeding or bleeding diathesis or significant coagulopathy (including familial) or a medical condition requiring long term systemic anticoagulation that would interfere with biopsies.<br>4. History of anaphylactic or other severe hypersensitivity / infusion reactions to ADCs or monoclonal antibodies, which in the opinion of the investigator may pose an increased risk of serious infusion reaction.<br>5. Treatment with any of the following anti-cancer therapies prior to the first dose of study treatment within the stated timeframes:<br>• ≤ 2 weeks for fluoropyrimidine therapy<br>• ≤ 4 weeks for radiation therapy or limited field radiation for palliation within ≤ 2 weeks prior to the first dose of study treatment.<br>• ≤ 4 weeks or ≤ 5 half-lives (whichever is shorter) for chemotherapy or biological therapy (including monoclonal antibodies) or continuous or intermittent small molecule therapeutics or any other investigational agent.<br>• ≤ 6 weeks for cytotoxic agents with major delayed toxicities, such as nitrosoureas and mitomycin C.<br>• ≤ 4 weeks for immuno-oncologic therapy, such as CTLA-4, PD-1, or PD-L1 antagonists.<br>6. Clinically significant and / or uncontrolled heart disease such as congestive heart failure requiring treatment (NYHA grade ≥ 2) or clinically significant arrhythmia despite medical treatment.
**Study treatment**	DYP688 single agent
**Efficacy assessments**	Tumor assessments per RECIST 1.1
**Pharmacodynamic assessments**	Assessment in tumor of expression and localization of biomarkers including but not limited to PMEL17 & pERK, DUSP6 & RASGRP3 in mRNA, and sequencing of cancer related genes<br>Presence of emerging, existing, and resistance mutations, tumor mutational burden, and cfDNA with relationship to clinical response and development of tumor resistance
**Pharmacokinetic assessments**	Concentrations of DYP688 (as total antibody) in serum<br>Concentrations [redacted] in plasma<br>Concentrations of [redacted] SDZ475 [redacted] in blood<br>Anti-drug antibodies (ADA)
**Key safety assessments**	Physical examination, lab assessments, Electrocardiogram (ECG), monitoring of adverse events
**Other assessments**	Complete ophthalmologic exam

**Data analysis**	In the escalation part, a Bayesian Logistic Regression Model (BLRM) will be used to guide the dose escalation and determine the MTD/RD. In the phase II part, the study will assess the preliminary anti-tumor activity, as well as the safety, tolerability, PK, and PD of DYP688 single agent in the defined patient population at the MTD/RD.
**Key words**	Phase I/II, DYP688, metastatic uveal melanoma (MUM), GNAQ/11, PMEL17, tebentafusp

1 Introduction

1.1 Background

Uveal melanoma is a malignant neoplasm of the eye, biologically distinct from melanoma of the skin, and although it represents only 3 to 5% of all melanomas, it is the most common primary intraocular malignant tumor in adults. Arising in the pigmented portions of the eye, specifically in the iris, ciliary body or choroid, uveal melanoma has a population incidence of approximately five cases per million individuals globally (Aronow et al 2018). The disease incidence is roughly equivalent between genders, but varies significantly with latitude and skin pigmentation, and has an approximately 8-fold higher incidence in fair-skinned Caucasian populations than in Asian and dark-skinned populations (Virgili et al 2007).

Nearly 50% of patients with uveal melanoma develop metastatic disease within 15 years of their initial diagnosis (Kujala et al 2003). Frequent sites of metastasis are the liver (95%), lungs (24%), bone (16%), and skin (11%), and rarely the brain and lymph nodes. The median overall survival (mOS) for patients with metastatic disease ranges from ~5-19 months with a one-year survival of approximately 10 to 15% (Buder et al 2013; Woodman et al 2012). No standard of care exists and, due to the lack of available therapies, outcome for patients with metastatic disease remains extremely poor. Systemic chemotherapy, hepatic artery infusion, hepatic embolization and surgical resection have all been used, but with varying and limited success (Piperno-Neumann et al 2016; Carvajal et al 2017; Rodriguez-Vidal et al 2020). Despite showing significant survival benefit in cutaneous melanoma, immunotherapies have shown modest impact on modulating the course of disease in metastatic uveal melanoma (MUM) (Bender et al 2017). The anti-CTLA4 therapy ipilimumab provided only modest benefit with mOS of 10 months, and anti-PD-1 therapies nivolumab and pembrolizumab showed similar OS outcomes coupled with limited improvement in progression free survival (PFS) (Namikawa et al 2020; Rossi et al 2019; Algazi et al 2016). Combinations of anti-CTLA4 and anti-PD-1 immunotherapies appear to provide the best overall patient benefit with mOS of up to 19 months in some studies, although sample size in these studies was small (Pelster et al 2019). Most recently, clinical trial data with a bi-specific fusion protein (tebentafusp, Immunocore) demonstrate that immunotherapies that are designed to redirect T-cells to tumor cells can improve OS in patients with MUM (Middleton et al 2020; Piperno-Neumann et al 2021). In a randomized Phase 3 trial, overall survival for MUM patients at 1 year was 73% in the tebentafusp group versus 59% in the control group (investigators choice). Despite improvements in OS, tebentafusp did not improve overall response rate (ORR) or median PFS. (Nathan et al 2021). Moreover, based upon its mechanism of action, a clear limitation in the therapeutic approach of tebentafusp is the requirement for patients to be HLA-A*02:01 positive, thereby excluding at least 50% of patients who have no current treatment for metastatic disease (Marincola et al 1992).

Somatic mutations in either GNAQ or GNA11, genes that encode G protein alpha subunits of heterotrimeric G protein-coupled receptor (GPCR) complexes, are present in up to 90% of patients with MUM (van Raamsdonk et al 2009; van Raamsdonk et al 2010; Piperno-Neumann et al 2020). GPCRs are increasingly recognized as promoters of malignancy in diverse cell types, including melanocytes (Rozengurt 2007), and Table 1-1 summarizes the incidence of mutations affecting GNA11 or GNAQ in melanocytic neoplasms

(van Raamsdonk et al 2010; Sheng et al 2016). The incidence varies with clinical setting, suggesting that the products of the two genes contribute unequally to the biology of uveal melanoma. In addition to the prevalence of GNAQ/11 mutations in uveal melanoma, mucosal melanoma, and other dermal melanocytic tumors such as blue naevi, a subset of cutaneous melanomas (1-4%) can also harbor GNAQ/11 mutations (Livingstone et al 2020; Akbani et al 2015).

Table 1-1 Epidemiology of G (alpha) subunit mutations in melanocytic neoplasms

Melanocytic neoplasm	N	GNA11 (%)	GNAQ (%)	Either (%)
Blue nevus	139	7	56	63
Uveal melanoma primary	163	34	48	82
Uveal melanoma metastasis	23	62	28	90
Mucosal melanoma	284	4.9	4.6	9.5

As mutations in GNAQ/11 are the key drivers leading to the development of uveal melanoma, targeted and specific inhibition of GNAQ/11 should result in robust and durable anti-tumor activity. Melanomas that may be driven by GNAQ/11 mutations appear biologically distinct from melanomas that typically harbor BRAF or NRAS mutations and have low mutational burden. Although this biological representation may explain why MAPK inhibitors have been ineffective in treating this subset of melanoma patients, targeting the GNAQ/11 mutation may lead to robust anti-tumor responses (Carvajal et al 2017).

1.1.1 Overview of DYP688

DYP688, is a first-in-class PMEL17 targeting Antibody-drug Conjugate (ADC), composed of [redacted], a highly selective humanized cysteine engineered PMEL17 monoclonal antibody, conjugated [redacted] on its engineered sites to the specific Gq/11 inhibitor SDZ475 via the maleimide-based stable ValCit linker.

DYP688 is a “targeted-targeted” ADC that first “targets” PMEL17, a melanocyte lineage protein that is highly expressed in primary and metastatic cutaneous and uveal melanoma, and to some extent in normal tissues containing melanocytic cells (i.e. retina, skin, and substantia nigra) (Wagner et al 1997, Rothermel et al 2016). Upon ADC binding to PMEL17 on the target cells and internalization, the linker is cleaved to release the “targeted” Gq/11/14 inhibitor SDZ475, which inhibits GNAQ/GNA11 oncogenic signaling, resulting in dose-dependent apoptosis. Moreover, inhibition of Gq/11 can result in upregulation of PMEL17, potentially further sensitizing melanoma cells to the pharmacological activity of DYP688. DYP688 selectively binds to PMEL17 and has human and cyno cross-reactivity. DYP688 shows dose-dependent anti-tumor activity across a panel of MUM xenografts, including models featuring different levels of PMEL17 and patient-derived xenografts of MUM. Preclinical toxicology showed that DYP688 was well tolerated with minimal and reversible clinical and histopathological findings, supporting the notion that DYP688 offers the possibility to specifically target GNAQ/GNA11-mutant melanocytic tumors with a favorable safety profile.

1.1.1.1 Non-clinical experience

1.1.1.1.1 Non-clinical pharmacology (*in-vitro*)

In biochemical assays, DYP688 displays binding to PMEL17 at subnanomolar concentrations (Kinexa KD: Hu 230 pM; Cy 160 pM). Selective binding of CCI [redacted] was assessed in a plasma membrane protein array employing 5528 full-length membrane and membrane-tethered secreted protein expressed in HEK293 cells. No off-target binder was identified in this screen. In cell-based assays, DYP688 inhibited the formation of GNAQ/GNA11 downstream second messenger IP3 (IC50 values of 0.15 nM in 92.1 cell model), and potently and selectively impaired the proliferation of PMEL17-expressing GNAQ/GNA11-mutant uveal melanoma cells, but not PMEL17-non expressing or GNAQ/GNA11 WT cancer cell lines (IC50 value of 0.31 in 92.1 GNAQ Q209L cell model; IC50 > 150 nM in PMEL17 KO 92.1 or PMEL17-positive GNAQ/GNA11 WT SKMEL30 models). In PMEL17-positive and -negative *in vitro* cell mixing studies, DYP688 displayed significant bystander cell killing. Pharmacodynamic markers of pathway inhibition (RASGRP3, DUSP6, and pERK) all decreased in a dose-dependent manner following Gq/11 inhibition and DUSP6 mRNA levels were highly correlated to the percentage of inhibition of proliferation in uveal melanoma cell lines.

1.1.1.1.2 Non-clinical pharmacology (*in-vivo*)

The anti-tumor activity of DYP688 was evaluated in multiple *in vivo* pharmacology models aimed to represent a range of clinical disease settings. In 92.1-luc tumor bearing nude mice, DYP688 demonstrated dose-dependent efficacy in a well-tolerated manner. A single 7.5 mg/kg i.v. dose resulted in tumor regression at day 8 (26% to 48%), although tumors began to regrow between day 16-20 post treatment. When a single dose of DYP688 was increased to 20 mg/kg i.v., the treatment achieved a maximal and sustained tumor regression (90% to 92% at day 10-13) that lasted for at least 65 days with no body weight changes observed (Figure 1-1).

Figure 1-1 Efficacy of DYP688 single dose i.v. in the 92.1 xenograft mouse model

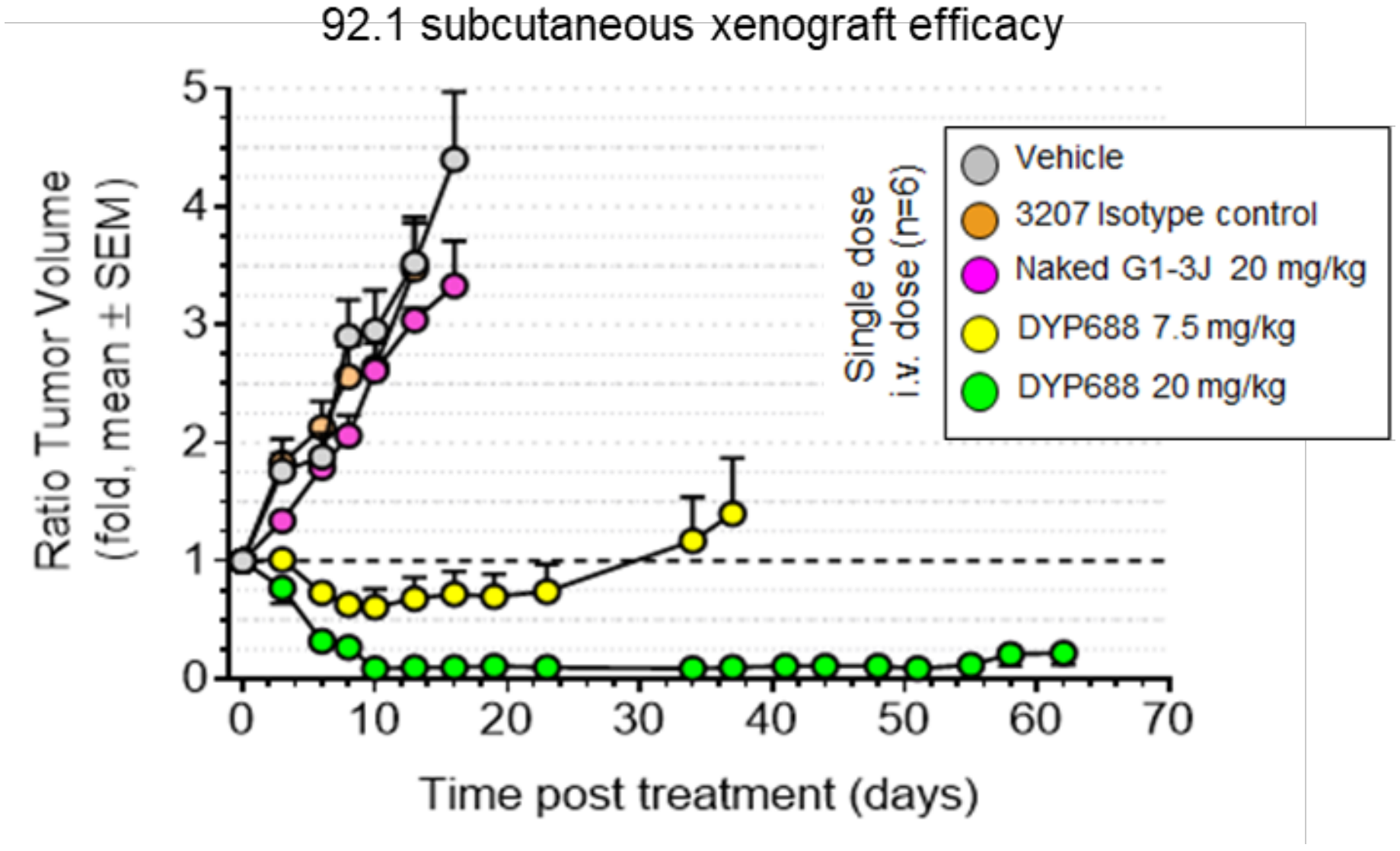

To assess the impact of PMEL17 expression on DYP688 anti-tumor activity, Mel270 tumor bearing nude mice were treated with DYP688. Despite the lower expression of PMEL17 in this model, a single dose of 12.5 mg/kg (i.v) still achieved maximal and sustained tumor regression (93% at day 14) lasting for at least 60 days with no body weight changes observed. Further evaluation of DYP688 in a difficult to treat preclinical model (30201-HX patient-derived MUM tumor bearing mice), also showed DYP688 dose-dependent efficacy in a well-tolerated manner. At the highest tested single dose (30 mg/kg, i.v) maximal tumor regression was achieved (54%) at day 11-12, with a clear regrowth phase at day 22 post treatment. The impact of repeat dosing (12.5 mg/kg i.v. administered Q2W regimen over 4 weeks or Q2W over 10 weeks) demonstrated similar tumor regression as the single dose studies with tumor regrowth occurring at ~day 20 post last treatment independent of treatment duration. Despite being well-tolerated, further increase of the DYP688 dose to 30 mg/kg i.v. administered Q2W over a 10 week period did not enhance efficacy nor time to regrowth in this model.

To model and assess the ability of DYP688 to treat patients with metastatic disease, DYP688 was evaluated in a 92.1-Gluc disseminated tumor NSG mouse model. DYP688 administered as a single 7.5 mg/kg i.v. dose induced maximal tumor regression at day 9 post single dose treatment (5.8%), and tumors began to grow again at day 13 post treatment. When DYP688 was administered at 12.5 mg/kg i.v. with a Q2W regimen over a 4 week period, complete regression (99%) was maintained over the 4 weeks of the treatment period and no significant body weight changes was observed. At day 12 post last treatment, we observed regrowth of the metastases as previously observed after single dose treatment.

Overall, the results of these *in vivo* studies demonstrate the robust anti-tumor activity of DYP688, and support the proposed Q2W dose regimen in this first in human (FIH) clinical trial.

1.1.1.1.3 Non-clinical pharmacokinetics and metabolism

The pharmacokinetics and metabolism of DYP688 was investigated *in vitro* assays using mouse, monkey or human matrices and *in vivo* in mouse and cynomolgus monkeys.

In vitro stability of DYP688 was assessed in plasma of mouse cynomolgus monkey and human. Evaluation of total antibody (tmAb) concentrations demonstrated that the antibody portion of the molecule was relatively stable over time, with [redacted]. In plasma from all three species, total active ADC (tADCa) levels [redacted] over time. It was determined that [redacted] SDZ475 has potential to be gradually metabolized by enzymatic ring-opening to the [redacted]. This leads to the presence of [redacted] The deactivation rate was similar between monkey and human plasma, whereas it was slower in mouse plasma. In the [redacted] form, only the [redacted] was observed [redacted] the LLOQ [redacted] in some samples.

Ligand binding assays were developed to quantify tADCa and tADCi, and, in addition, an assay was developed [redacted] by incubation with papain, followed by [redacted] an LC-MS method (papain release assay, PRA).

After a single i.v. dose of DYP688 to the mouse in the range of 0.03 to 0.3 mg/kg, concentration versus time profiles were observed for tmAb and tADCa, with half-lives in the range of [redacted] for tmAb, and [redacted] for tADCa.

After single or repeated i.v. administration of DYP688 in the cynomolgus monkey in the dose range of 3 to 75 mg/kg, tmAb exposures decreased over time with an elimination half-life of roughly [redacted] The exposure to tADCa [redacted] faster, with a half-life of approximately [redacted], whereas tADCi [redacted] after dosing, after which it [redacted] to tmAb (Figure 1-2). These exposure profiles of tADCa and tADCi are [redacted] measured by PRA.

DYP688 showed roughly dose proportional exposure between 3 and 75 mg/kg. Only minor accumulation was observed after three Q2W doses for tmAb and tADCi [redacted], whereas tADCa [redacted]. Plasma exposure of [redacted] SDZ475 was only detected at very low levels (<1 ng/mL) in all samples, even at the highest tested DYP688 dose of 75 mg/kg. Findings were consistent with the *in vitro* assessment of plasma stability.

No target mediated drug disposition was observed in monkey.

PK profiles in cynomolgus monkeys demonstrated a consistent rate of clearance, suggesting little to no formation and/or consequence of anti-drug antibody (ADA) complexes. Only one animal tested ADA positive in the monkey GLP toxicology study, with no significant effect on exposure.

Figure 1-2 Mean exposure profiles of tmAb, tADCa and tADCi after single i.v. dose of 3 mg/kg in the cynomolgus monkey

[redacted]

The pharmacokinetics and metabolism of the payload SDZ475 was investigated in *in vitro* assays using mouse, monkey or human matrices and *in vivo* in mouse.

The permeability of SDZ475 was found to be moderate. The plasma protein binding in mouse, monkey and human was determined to be 81, 64 and 90%, respectively, without any significant concentration dependency in the range of 1 to 1000 ng/mL. SDZ475 showed a concentration

dependent distribution into blood cells in fresh mouse, monkey and human blood. SDZ475 was predominantly associated with blood cells at the lowest concentration of 5 ng/mL (<15% in plasma), whereas it was mainly associated with plasma at the highest tested concentration of 1000 ng/mL (>70% in plasma). At concentrations [redacted] SDZ475 measured in toxicology studies, it is therefore likely to reside mainly in the cellular fraction of the blood.

The pharmacokinetics of SDZ475 was investigated in a GLP toxicology study in mice at doses of 0.03, 0.1 and 0.3 mg/kg, administered by intravenous bolus injection. SDZ475 was rapidly cleared from circulation, with an estimated half-life of <20 minutes. The increase in SDZ475 exposure was greater than dose proportional for both male and female mice, and no gender difference was observed.

As an IgG-based ADC, DYP688 is unlikely to cause or be affected by drug-drug interaction (DDI) itself, and SDZ475 is not expected to be a perpetrator of DDI due to its low CYP450 inhibition (Schlegel et al 2021) and very low exposures (<0.5 ng/mL after dosing DYP688 at 75 mg/kg in monkey, and a predicted Cmax of 0.04 ng/mL in human at the starting does of 4 mg/kg). In addition, SDZ475 is not expected to be a DDI victim since the rapid deactivation through ring-opening is mediated by enzymes present in plasma and are not likely to be inhibited by specific inhibitors. SDZ475 is readily metabolized by liver microsomal enzymes, likely cytochrome P450s. Hence, there is a possibility that strong CYP inhibitors may slow the hepatic metabolism of SDZ475. However, given the extremely low levels of SDZ475 detected in circulation in monkey, and the rapid deactivation by plasma enzymes before it can reach the liver, the possibility for a CYP based DDI to cause a significant increase in concentrations of SDZ475 is considered to be very low.

1.1.1.1.4 Non-clinical toxicology

The nonclinical safety profile of DYP688 has been evaluated in cynomolgus monkeys both in a non-GLP dose-range-finding (DRF) study of 6 weeks (4 doses) and a pivotal GLP-compliant 13-week toxicity study (7 doses) with Q2W dosing. All in-life observations as well as most clinical and anatomic pathology observations can be related to the pharmacology of SDZ475 (Gq/11 protein inhibition).

In the DRF study, transient inhibition of ex-vivo whole blood aggregometry (WBA) was observed at ≥30 mg/kg Q2W. The NOAEL in this study was set at 75 mg/kg Q2W.

In the pivotal 13-week GLP study with a 3-week recovery period, transient bilateral mydriasis was observed at ≥30 mg/kg Q2W. Mydriasis appeared within 1 to 4 days after dose administration and normalized within 10 days prior to next dosing. In mydriatic animals, the menace reflex was normal and the pupillary light reflex was normal except on single occasions (days)/single animals. There were no other observations upon ophthalmic examination including tonometry. Other clinical signs observed in the 13-week GLP study included a slightly higher incidence of alopecia and scabs compared to controls.

There were no effects on food consumption, body weight, auditory stimulus test (normal reaction), coagulation, and urinalysis.

A decrease in mature red blood cell mass was observed in all animals including controls; however, the decrease (likely related to intense blood sampling) was slightly and dose-dependently higher in treated animals. There was a concurrent increase in relative and

absolute reticulocyte numbers. Red blood cell mass had a partial return to baseline values and stabilization during treatment and reversed during the treatment-free period (3 weeks). The ex-vivo WBA showed a dose-dependent decrease in treated animals, evident when both TRAP6 (agonist) or collagen (agonist) were used to induce platelet aggregation. There was partial reversibility during the treatment cycle and full reversibility after the recovery period.

In clinical chemistry, there was a mild increase in serum calcium concentration and a mild dose-dependent increase in magnesium concentration at ≥30 mg/kg Q2W compared to controls.

In anatomic pathology, higher organ weights have been recorded for the testes after the recovery period (+47% relative to body weight at 75 mg/kg Q2W). Macroscopically, there were three treated animals with skin findings (scabs) on the tail which correlated microscopically in two animals with erosion/ulcer and were assessed to be related to trauma. Other microscopic findings were in the exocrine pancreas with dose-dependent minimal to moderate (single animal at 75 mg/kg Q2W) vacuolation of acinar cells and a higher incidence/severity (minimal to slight) of single cell necrosis compared to controls. In addition, minimal interstitial edema was recorded at 75 mg/kg Q2W. In the routine skin sample (thoracic skin including the area of the papilla mammaria), higher incidence/severity of minimal to slight epidermal vesicle, pustule, edema, degeneration/necrosis, and erosion/ulcer have been recorded compared to controls. In addition, treated animals had minimal to slight epidermal hyperplasia and dermal inflammation which were assessed to be secondary to the erosion/ulceration described above. Finally, minimal dermal hemorrhage has been observed in all females including controls.

In the inguinal skin sample (injection site), there were no microscopic differences between treated and control animals.

An equivocal finding was observed in the testes with higher severity (slight to moderate) of tubular dilatation at 75 mg/kg Q2W compared to controls (minimal only) as well as epidiymides (minimal cell debris) at 75 mg/kg Q2W.

All findings except epididymides have either fully (clinical signs, clinical pathology, skin, pancreas) or partially (testes) reversed after the 3-week recovery period. In the epidiymides, there was still presence of minimal cell debris which was assessed as secondary to the testicular finding.

All findings were minimal to mild in severity and showed full or partial reversibility within the 3-week recovery period all at dose levels tested; therefore, the HNSTD was determined to be 75 mg/kg Q2W, the highest tested dose.

According to ICH S9, no stand-alone safety pharmacology study has been conducted with DYP688. The functional effects of on major physiological systems (cardiovascular, respiratory, and central nervous systems) were adequately evaluated within the context of the DRF and pivotal GLP toxicity studies following repeat administration of DYP688 at doses up to 75 mg/kg Q2W in cynomolgus monkeys. There were no effects on cardiovascular, respiratory, or central nervous systems. Furthermore, SDZ475 IC50 values for hERG channel inhibition *in vitro* were >30µM, significantly higher than circulating concentrations anticipated in patients.

Genotoxicity of SDZ475 was assessed in an Ames assay both in non-GLP and GLP studies as well as by a combined micronucleus test/Comet assay in mice. There was no evidence of genotoxic potential in in vitro and in vivo studies.

For further information, please refer to the DYP688 Investigator Brochure.

1.1.1.2 Clinical experience

CDYP688A12101, is an ongoing first in human (FIH) study in patients with metastatic uveal melanoma and other GNAQ/11 mutant melanomas. As of protocol amendment 06 and the clinical data cutoff date of 25-Oct-2024, doses of 4, 8, 12, 16, and 24 mg/kg administered every two weeks (Q2W) and 12 and 16 mg/kg administered every week (QW) intravenously have been evaluated in the dose escalation part of the study. In total, 66 patients have been enrolled and treated with single-agent DYP688. One DLT of grade 3 hypotension was observed in one patient treated at 24 mg/kg Q2W and was reported as a serious adverse event (SAE). The most common treatment related AEs (all grades/doses) were fatigue, hypercalcemia, dry mouth, and peripheral edema. Thirteen non-serious ocular toxicities ≤ grade 2 in severity have been reported as related to study treatment. None of these events required dose reduction or study drug interruption or discontinuation. Preliminary anti-tumor activity was observed at doses ≥12 mg/kg Q2W. Among 43 patients who had at least one post-baseline disease evaluation per RECIST treated with DYP688 at 12 mg/kg Q2W or higher, a total of 10 confirmed partial responses (PR) and 1 confirmed complete response (CR) were observed.

For further details, please refer to the DYP688 Investigator's Brochure.

1.2 Purpose

The purpose of this Phase I/II, FIH study is to characterize the safety, tolerability, and anti-tumor activity of DYP688 as a single agent in patients with MUM and potentially other melanomas harboring GNAQ/11 mutations. The main purpose of Phase I/dose escalation part of the study is to explore the safety and tolerability and determine the maximum tolerated dose (MTD) and/or recommended dose(s) (RD) of DYP688. The purpose of the Phase II part of the study is to assess the anti-tumor activity of DYP688 and to further explore safety and tolerability of DYP688 at the RD. Additionally, the study will aim to characterize the pharmacokinetics (PK) of DYP688 and the PK/PD relationship.

2 Objectives and endpoints

Table 2-1 Objectives and related endpoints

Objective(s)	Endpoint(s)
**Primary Objective(s)**	**Endpoint(s) for primary objective(s)**
Phase I<br>• To characterize the safety and tolerability and to identify the MTD and/or RD and regimen for future studies of DYP688 as a single agent	Safety: Incidence and severity of dose limiting toxicities (DLTs) during the first 28 days of treatment. Incidence and severity of adverse events (AEs) and serious adverse events (SAEs), including changes in laboratory values, electrocardiograms (ECGs), and vital signs<br>Tolerability: Frequency of dose interruptions, reductions, and discontinuations
Phase II<br>• To evaluate the anti-tumor activity of DYP688 as a single agent	Overall Response rate (ORR) per RECIST 1.1*
**Secondary Objective(s)**	**Endpoint(s) for secondary objective(s)**
Phase I<br>• To characterize the pharmacokinetics of DYP688 as a single agent	PK parameters for total mAb, CCI [redacted] (e.g., AUC, Cmax, CL, half-life)
• To assess the immunogenicity (IG) of DYP688 as a single agent	Prevalence and incidence of anti-DYP688 antibodies
• To evaluate the preliminary anti-tumor activity of DYP688 as a single agent	Best Overall Response (BOR), Overall Response Rate (ORR) per RECIST v1.1
Phase II<br>• To further evaluate the anti-tumor activity of DYP688 as a single agent	Duration of response (DoR), progression free survival (PFS) and DCR per RECIST v1.1
• To evaluate the effects of DYP688 as a single agent on overall survival	Overall survival (OS)
• To further characterize the safety and tolerability of DYP688 as a single agent	Safety: Incidence and severity of adverse events (AEs) and serious adverse events (SAEs), including changes in laboratory values, electrocardiograms (ECGs), and vital signs<br>Tolerability: Frequency of dose interruptions, reductions, and discontinuations
• To further characterize the pharmacokinetics of DYP688 as a single agent	PK parameters for total mAb, CCI [redacted]

Objective(s)	Endpoint(s)
	[redacted] (e.g., AUC, Cmax, CL, half-life)
• To further assess the immunogenicity (IG) of DYP688 as a single agent	Prevalence and incidence of anti-DYP688 antibodies
**Exploratory Objective(s)**	**Endpoint(s) for exploratory objective(s)**
[redacted]	
*ORR in Phase I will be evaluated first by local review per RECIST 1.1 and may be followed by a retrospective central review. ORR in Phase II will be evaluated by central review per RECIST 1.1.	

2.1 Primary estimands

Phase I – Dose escalation

Not applicable.

Phase II

The primary clinical question of interest is: What is the treatment effect of DYP688 as a single agent in terms of ORR by central assessment per RECIST 1.1 in patients with MUM irrespective of prior systemic treatment history other than tebentafusp and regardless of study treatment discontinuation or use of concomitant medication, and before the start of any new anti-neoplastic therapy. The justification for the primary estimand is that it will capture both the effect of the study drug and the effect of additional medications, mirroring the conditions in clinical practice, but avoid potential confounding effects of any other new anti-neoplastic therapy. Further details can be found in Section 12.

The primary estimand is described by the following attributes:

1. Population: Group 1: Patients with MUM, previously treated but naive to tebentafusp or treatment naive in the metastatic setting; Group 2: Patients with MUM, previously treated with tebentafusp in addition to other treatments or naive to other treatments in the metastatic setting. Further details on the population are provided in Section 5.
2. Endpoint: confirmed Overall response rate (ORR) by central assessment as per RECIST 1.1. The ORR is defined as the proportion of patients with best overall response (BOR) of either confirmed complete response (CR) or confirmed partial response (PR), as per central assessment according to RECIST 1.1, recorded from starting study treatment until PD, death, start of new therapy, withdrawal of consent or end of study, whichever comes first.
3. Treatment of interest: single agent DYP688 regardless of discontinuation or use of any concomitant medication. Further details about the treatment are provided in Section 6.
4. Handling of intercurrent events (refer to Section 12.4.2.2):
    - Discontinuation of study treatment for any reason (per the treatment policy strategy)
    - Start of new anti-neoplastic therapy (per the "while on treatment" policy)
    - Usage of any concomitant medication (per the treatment policy strategy)
    - Any public health emergency as declared by local or regional authorities, e.g., pandemic, epidemic or natural disaster etc. (per the treatment policy strategy)
5. The summary measure: proportion of patients with BOR of a confirmed CR/PR by central assessment as per RECIST 1.1 with its corresponding two-sided exact binomial 95% CI evaluated for each group separately.

2.2 Secondary estimands

Not applicable.

3 Study design

This is a FIH, phase I/II, open label, multi-center, single arm study of DYP688 as a single agent. There will be two parts to this study: a phase I, dose escalation part followed by a phase II part. Dose escalation will be conducted in adult patients with MUM and other melanomas harboring GNAQ/11 mutations. Once the MTD and/or RD(s) is determined in the dose escalation part, the study may continue with a phase II part. The phase II part will be conducted in two groups

of patients with MUM, a prior tebentafusp-treated group and a tebentafusp-naïve group. In addition to MUM, a third group of patients with non-uveal GNAQ/11 mutant melanomas may also be explored. This cohort may be opened based on emerging data from the dose escalation part of the study. Study drug will be administered until a patient experiences unacceptable toxicity, progressive disease by RECIST 1.1 by investigator assessment, or the treatment is discontinued at the discretion of the investigator or the patient. Alternative dosing regimens (e.g. less or more frequent dosing) may be implemented during the study if supported by available clinical data including preliminary PK, PD, safety, and efficacy findings.

Additionally, adolescent patients may be eligible for enrollment in the Phase II part of the study, once the initial safety profile and a pharmacologically active dose have been identified in adults. In countries where required by the local health authority, enrollment will be restricted to adult patients ≥18 years of age.

3.1 Phase I – Dose Escalation

The escalation part of the study will be conducted in patients with MUM and other melanomas harboring GNAQ/11 mutations. All dose escalation decisions will be guided by an adaptive Bayesian logistic regression model (BLRM) following the Escalation with Overdose Control (EWOC) principle. A minimum of 14 patients may be required during dose escalation to define the MTD. The RD(s) refers to the recommended regimen, dose(s) and schedule(s) for further exploration. The safety (including the dose-DLT relationship) and tolerability of study treatment will be assessed, and regimen(s) including dose(s) and schedule(s) will be identified for use in the phase II part based on the review of all available data including PK, PD, and preliminary anti-tumor activity.

To begin, DYP688 single agent will be dosed every two weeks (Q2W) during a 28-day cycle. Based on emerging data, Novartis may, in agreement with the study investigators, implement alternative dosing regimens –less or more frequent dosing QW and /or Q4W—during the study if supported by available non-clinical and clinical data including preliminary PK, PD, safety and efficacy findings.

3.2 Phase II

Once an MTD/RD(s) has been identified the study will continue in the phase II part in order to assess the anti-tumor activity of DYP688 as single agent in defined patient populations and further characterize the safety, PK, and PD of the study treatment. Both the prior tebentafusp treated and tebentafusp naive groups will enroll approximately 40 patients each. The non-uveal GNAQ/11 mutant melanoma group may enroll approximately 10-20 patients, if explored. Enrollment in any of these groups may be halted early based on review of emerging data from the dose escalation part or the ongoing review of data from the phase II part of the study. Details of sample size calculation are provided in Section 12.8.

Figure 3-1 Study design

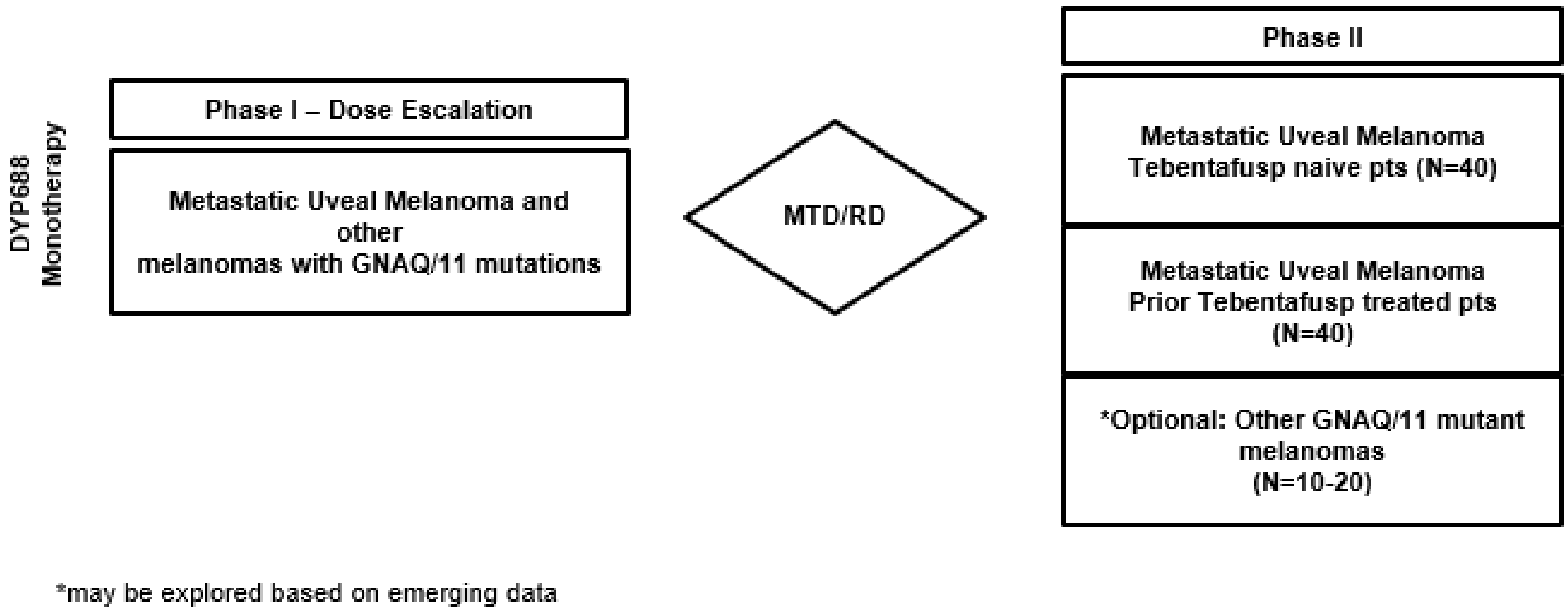

Figure 3-2 Study Visit Flow

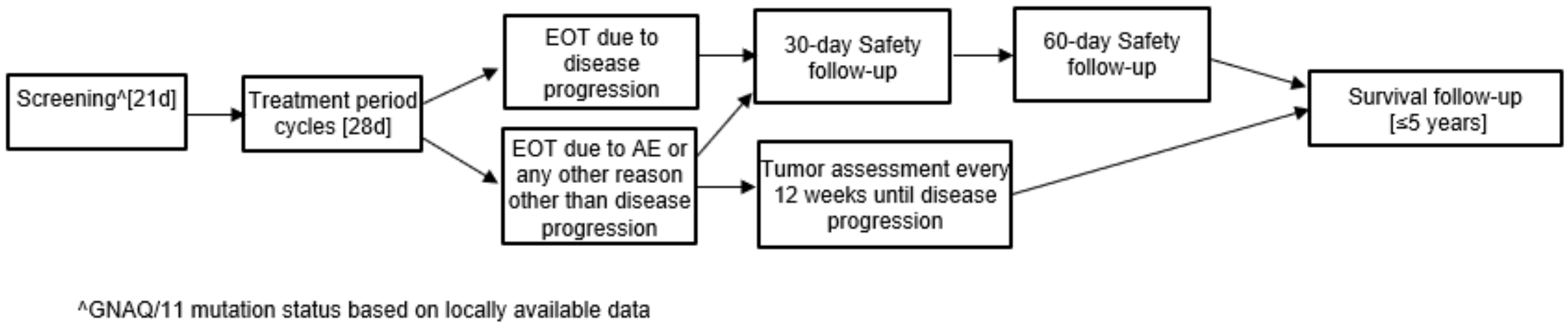

4 Rationale

4.1 Rationale for study design

The design of this phase I/II, open label study aims to characterize the safety and tolerability of DYP688 single agent in patients with MUM and other melanomas harboring GNAQ/11 mutations, and to determine a recommended regimen (including dose(s) and schedules(s)) for phase II and future studies. Where necessary, the dose escalation allows the MTD of DYP688 single agent to be established and will be guided by a BLRM along with EWOC principle.

A BLRM is a well-established method to estimate the MTD in patients with cancer. The adaptive BLRM will be guided by the EWOC principle to control the risk of DLT in future patients on study. The use of Bayesian response adaptive models for small datasets has been

accepted by EMA ("Guideline on clinical trials in small populations", February 1, 2007) and endorsed by numerous publications (Babb et al 1998; Neuenschwander et al 2008) and its development and appropriate use is one aspect of the FDA's Critical Path Initiative.

The phase II part of this study will contain two defined groups of patients with MUM: tebentafusp-treated and tebentafusp-naive. Post-treatment assessment of PMEL17 levels from tebentafusp-treated patients suggests some patients may have up to a 50% decrease in PMEL17 expression (Middleton et al 2020; Butler et al 2021). Recruitment to these specific groups in the phase II part of the study will ensure inclusion of patients with MUM regardless of prior therapy received, while also allowing for exploratory analysis of any difference in response observed between patients who received prior tebentafusp and those who did not. To assess the anti-tumor activity of DYP688, hypothesis testing based on point estimate of ORR and exact 95% confidence interval will be implemented for each MUM group separately. To minimize the risk of exposing patients to an inactive treatment and to provide a preliminary evaluation for efficacy, an interim analysis (IA) for futility will be conducted for each MUM group separately for the phase II part of the study. Non-uveal melanomas harboring GNAQ/11 mutations may be biologically more related to uveal melanoma than melanomas harboring other mutations (e.g. BRAF, NRAS) (Carvajal et al 2017), therefore a third group targeting patients with non-uveal melanomas harboring a GNAQ/11 mutation may be explored based on emerging data from the dose escalation part of the study.

4.2 Rationale for dose/regimen and duration of treatment

This is the first trial that will evaluate DYP688 in humans. The selection of the starting dose for DYP688 is consistent with the ICH S9 guidelines for choosing a starting dose for a first-in-human trial conducted in patients with cancer. Preclinical pharmacology and PK/PD data also informed the selection of the starting dose. The proposed starting dose of DYP688 is 4 mg/kg Q2W, administered via intravenous infusion. The predicted efficacious dose is likely to be in the range of 2-15 mg/kg every 2 weeks.

The nonclinical safety profile of DYP688 has been evaluated in cynomolgus monkeys both in a non-GLP dose-range-finding (DRF) study of 6 weeks (4 doses) and the pivotal GLP-compliant 13-week toxicity study (7 doses). All in-life observations as well as most clinical and anatomic pathology observations can be related to the pharmacology of SDZ475 (Gq/11 protein inhibition). All findings were minimal to mild in severity and showed full or partial reversibility within the 3-week recovery period at all doses levels tested; therefore, the HNSTD was set to the highest tested dose of 75 mg/kg Q2W.

Two retrospective analyses (Saber et al 2019; Saber, Leighton 2015) of multiple ADCs found that selecting a starting dose using 1/6th of the BSA-based converted HNSTD in cynomolgus monkeys) generally resulted in an acceptable balance of evaluation of safety with an effective dose-escalation in Phase 1 trials. This approach also avoids treating patients at sub-therapeutic dose levels and enables an efficient determination of the MTD (Saber, Leighton 2015). Based on the above, the starting dose for DYP688 was calculated by BSA conversion of 75 mg/kg (HNSTD) and applying a safety factor of 6, and yields a starting dose of 4 mg/kg.

Based on the predicted human PK at 4 mg/kg, the exposure margins achieved at HNSTD in cynomolgus monkey relative to the proposed starting dose (4 mg/kg Q2W) and the predicted efficacious dose range (2-15 mg/kg Q2W) are shown in Table 4-1.

Table 4-1 DYP688 tmAb [redacted]) exposure multiples in cynomolgus monkey relative to the predicted human exposure at the starting dose (4 mg/kg) and predicted efficacious dose range (2-15 mg/kg)

	HNSTD in cynomolgus monkey GLP study		Starting dose in patients		*Predicted efficacious dose range in patients	
Dose (mg/kg)	75		4		2-15	
Analyte	tmAb	[redacted]	tmAb	[redacted]	tmAb	[redacted]
Cmax (µg/mL)	3790	25.1	159	2.1	80-591	1.1-7.8
AUC336h (µg*h/mL)	328000	831	8900	54	4480-32900	27-198
Cmax exposure multiple in monkey compared to patients			24	12	47-6.4	24-3.2
AUC336h exposure multiple in monkey compared to patients			37	15	73-10	31-4.2

HNSTD = Highest non-severely toxic dose; tmAb = total monoclonal antibody; [redacted] SDZ475 (Section 1.1.1.1.3)

* This dose range represents the range of minimum efficacious doses (MED), whereas the lowest dose (2 mg/kg) reflects the predicted MED when applying the PK-efficacy model to the most sensitive xenograft tumor model 92.1, and the highest dose (15 mg/kg) the predicted MED when using the least sensitive preclinical tumor model 30201-HX.

Exposures represent steady state exposures in cynomolgus monkey (mean on Day 71) and predicted steady state exposures in human. The predicted half-life in human is 160 h for DYP688 total mAb, and approximately 44 h for the [redacted].

Based on the predicted human PK and computational models of tumor growth inhibition, exposures achieved between 2 and 15 mg/kg, administered Q2W, are predicted to achieve anti-tumor activity in patients. The dose escalation part of the study will start with the dosing schedule of DYP688 Q2W. Based on emerging clinical data, more or less frequent dosing regimens (QW, Q4W) may be explored in the interest of patient benefit and/or convenience. The starting dose for any alternative dosing schedule will be selected based on the totality of available safety, PK, PD, and efficacy data and will satisfy both of the following conditions:

- will not exceed the highest dose level satisfying the EWOC principle by the BHLRM model for the new dosing schedule
- the cumulative dose of DYP688 over the DLT period cannot exceed the cumulative Q2W dose (over the same period) already tested and deemed to be safe at the time of the switch.

A separate BHLRM will be set up to guide the selection of starting dose and dose escalation for the new dosing schedule.

4.3 Purpose and timing of interim analyses/design adaptations

There is no formal IA planned for the dose escalation part. However, the dose escalation design foresees that decisions on future dose levels and/or regimens based on available data are taken at the end of each cohort. These are described in Section 6.6.2 and Section 12.8.

In addition, in the phase II part, an IA for futility will be conducted after at least 15 patients of each group have been treated and have completed at least four cycles (at least two post-baseline tumor assessments), progressed, died, withdrawn consent, been lost to follow-up, started a new antineoplastic therapy or followed-up for at least 4 months, whichever occurs first (Section 12.7). The IA for futility will be conducted to assess the overall efficacy of DYP688 single agent in MUM patients based on ORR by central review per RECIST 1.1. Enrollment will continue until the futility boundary is met based on the pre-specified rule in (Section 12.7)

Safety data will be monitored throughout the phase II part of the study. At the time of planned IA or at least twice per year, if any of the stopping rule criteria in Section 9.5 are met for a Phase II group, then enrollment to that phase II group will be paused and all the safety of the RD will be re-evaluated in a meeting with the DMC.

4.4 Risks and benefits

As of protocol amendment 05, the FIH study of DYP688 is continuing to generate preliminary clinical data in dose escalation. Anticipated risks and benefits are therefore primarily based on preclinical toxicity and pharmacology data, as described in Section 1.1.1.1.1 to Section 1.1.1.1.4 and in the DYP688 Investigator's Brochure.

The trial will enroll patients with MUM and other malignant melanomas harboring GNAQ/11 mutations for whom there is currently no approved curative therapy or established standard of care. These patients have limited treatment options, all of which (including chemotherapy, immunotherapy, and other targeted therapies) are experimental. The doses of DYP688 tested in this study may be pharmacologically active and result in an anti-tumor response. While there may be no clinical benefit to participating patients, the trial offers the opportunity for patients to receive a novel investigative therapy for incurable disease.

Appropriate eligibility criteria and specific dose-limiting toxicity definitions, as well as specific dose modification and study treatment discontinuation rules, are included in this protocol. Based on the patterns of toxicities in preclinical toxicology studies patients will be monitored for potential ophthalmologic, dermatologic, hematologic, and pancreatic effects. For further details please refer to the DYP688 Investigator's Brochure.

The risk to patients in this trial may be minimized by compliance with the eligibility criteria and study procedures, as well as close clinical monitoring, dose modification and stopping rules, and, in the Phase II portions, periodic review of safety data by an independent data monitoring committee (DMC) as outlined in the clinical trial protocol.

Women of child-bearing potential and sexually active males must be informed that taking the study treatment may involve unknown risks to the fetus if pregnancy were to occur during the study, and agree that in order to participate in the study they must adhere to the contraception requirements outlined in the exclusion criteria. If there is any question that the patient will not reliably comply, they should not be entered or continue in the study.

Melanoma is a disease that may occur in adolescents and therefore this study is open to patients ≥12 years of age in the phase II part of the study once sufficient safety and tolerability data have been generated in adult patients and following a documented discussion with the Novartis medical monitor. Investigative sites are not required to enroll adolescent patients but this study allows enrollment of adolescents who fulfil eligibility criteria, in countries and sites where this is feasible. The same risks and benefits apply to adolescents and adults enrolled in this study. In countries where required by the local health authority, enrollment will be restricted to adult patients ≥18 years of age.

4.5 Rationale for public health emergency mitigation procedures

During a public health emergency as declared by local or regional authorities, e.g., pandemic, epidemic or natural disaster, mitigation procedures required to ensure patient safety and trial integrity may be implemented. Notification of the public health emergency as declared by local or regional authorities should be discussed among investigators and Novartis. All procedures adapted to the situation must be submitted, if required as per local regulations, through a protocol amendment for approval by local or regional Health Authorities and Ethics Committees prior to implementation of mitigation procedures.

5 Study Population

In this study, DYP688 will be administered to patients with MUM and other, non-uveal GNAQ/11 mutant melanomas. The investigator or designee must ensure that only patients who meet all the following inclusion and none of the exclusion criteria are offered treatment in the study. All data for the inclusion and exclusion criteria must be verifiable in the patient's source documents.

A written Informed Consent Form (ICF) will be obtained from each patient before any study procedures not considered part of standard of care are initiated. For adolescent patients**,** consent will be obtained from parent(s) or legal guardian(s) and the signature of at least one parent or guardian will be required. Investigators will also obtain assent of patients according to local, regional, or national guidelines.

5.1 Inclusion criteria

Patients eligible for inclusion in this study must meet **all** of the following criteria:

1. Signed informed consent must be obtained prior to participation in the study

   For adolescent patients: consent will be obtained from parent(s) or legal guardian(s) and the signature of at least one parent or guardian will be required. Investigators will also obtain assent of patients according to local, regional or national guidelines.
2. Patients in the dose escalation part must be ≥ 18 years of age at the time of informed consent (ICF) signature. In the phase II part, patients ≥ 12 years of age at the time of informed consent may be eligible for enrollment (not applicable in countries where enrollment is restricted by the local health authority to patients ≥ 18 years of age). Patients must have a minimum weight of 40 kg. For the 32 mg/kg dose level a maximum weight limit of 115 kg may not be exceeded.

3. ECOG performance status ≤ 1 for patients ≥ 18 years of age; Karnofsky performance status ≥ 70 for patients ≥ 16 and < 18 years of age; Lansky performance status ≥ 70 for patients ≥ 12 and < 16 years of age
4. Measurable disease at the time of enrollment, one lesion that can be accurately measured in at least one dimension (longest diameter to be recorded) as > 20 mm with conventional techniques (refer to Section 16.1) or as >10 mm with CT scan.
5. Patients must be suitable and willing to undergo study required biopsies according to the treating institution's own guidelines and requirements. A new tumor biopsy will be obtained at screening and during therapy on the study, if medically feasible. If a biopsy is not medically feasible, exceptions may be considered after documented discussion with Novartis.

Note: An archival biopsy may be accepted at screening if collected within 6 months of treatment start and no intervening treatment has been received since.

For all patients in Dose Escalation

6. MUM: uveal melanoma with histologically or cytologically confirmed metastatic disease. Patient must be either treatment naive or have received any number of prior lines and progressed on most recent therapy.
7. Non-MUM: advanced cutaneous or mucosal melanoma with histologically or cytologically confirmed metastatic disease that has progressed following all standard therapies or that has no satisfactory alternative therapies and has evidence of GNAQ/11 mutation based on local data

For patients in Phase II

8. Tebentafusp naïve group: Diagnosis of uveal melanoma with histologically or cytologically confirmed metastatic disease that has progressed following standard therapies or that has no satisfactory alternative therapies
9. Tebentafusp pre-treated group: Diagnosis of uveal melanoma with histologically or cytologically confirmed metastatic disease. Patients must be previously treated with tebentafusp and have progressed or discontinued due to an adverse event. If tebentafusp was discontinued due to an AE, the disease must have progressed on the most recent standard therapy prior to study entry
10. Non-MUM: patients with diagnosis of cutaneous or mucosal melanomas harboring GNAQ/11 mutations based on local data, with histologically or cytologically confirmed metastatic disease that has progressed following all standard therapies or that has no satisfactory alternative therapies

5.2 Exclusion criteria

Patients meeting any of the following criteria are not eligible for inclusion in this study.

1. Malignant disease, other than that being treated in this study. Exceptions to this criterion include the following: malignancies that were treated curatively and have not recurred within two years prior to study treatment; completely resected basal cell and squamous cell skin cancers; any malignancy considered to be indolent and that has never required therapy; and completely resected carcinoma in situ of any type.

2. Active brain metastases, i.e. symptomatic brain metastases or known leptomeningeal disease. Asymptomatic brain metastases are allowed if they meet the following criteria:
   - Have been treated and have been stable for greater than or equal to 4 weeks as documented by radiologic imaging.
   - Have not required increasing doses of corticosteroids within 2 weeks prior to study treatment.
   - Patients who have had complete surgical resection or received stereotactic radiosurgery will be permitted to enroll in the study provided that they completed treatment >2 weeks prior to study treatment, have recovered from treatment, are clinically/neurologically stable and do not require escalating corticosteroid therapy as noted above. If treatment was completed >4 weeks prior to study entry, patients must not have new findings on central nervous system (CNS) imaging.
3. Evidence of active bleeding or bleeding diathesis or significant coagulopathy (including familial) or a medical condition requiring long term systemic anticoagulation that would interfere with biopsies. Low-dose aspirin treatment (up to 200 mg/day) or anticoagulation therapy that can be interrupted to allow biopsies are allowed.
4. History of anaphylactic or other severe hypersensitivity / infusion reactions to monoclonal antibodies and excipients, which in the opinion of the investigator may pose an increased risk of serious infusion reaction.
5. History of allogeneic bone marrow or solid organ transplant.
6. Use of any live vaccines against infectious diseases within 4 weeks of initiation of study treatment
7. Treatment with any of the following anti-cancer therapies prior to the first dose of study treatment within the stated timeframes:
   - ≤ 2 weeks for fluoropyrimidine therapy
   - ≤ 4 weeks for radiation therapy or limited field radiation for palliation within ≤ 2 weeks prior to the first dose of study treatment.
   - ≤ 4 weeks or ≤ 5 half-lives (whichever is shorter) for chemotherapy or biological therapy (including monoclonal antibodies) or continuous or intermittent small molecule therapeutics or any other investigational agent.
   - ≤ 6 weeks for cytotoxic agents with major delayed toxicities, such as nitrosoureas and mitomycin C.
   - ≤ 4 weeks for immuno-oncologic therapy, such as CTLA-4, PD-1, or PD-L1 antagonists.
8. Patients who have undergone major surgery ≤ 4 weeks prior to first dose of study treatment or who have not recovered from the surgical procedure.
9. Clinically significant cardiac disease or risk factors at screening including any of the following:
   - Clinically significant and / or uncontrolled heart disease such as congestive heart failure requiring treatment (NYHA grade ≥ 2) or clinically significant arrhythmia despite medical treatment.

- Uncontrolled hypertension, defined by blood pressure ≥140/90 mm Hg at rest (average of 3 consecutive readings); treatment is permitted to control the hypertension

10. QTcF > 470 msec (as a mean value of triplicates) on screening ECG or congenital long QT syndrome
11. Acute myocardial infarction or unstable angina pectoris < 3 months prior to study entry.
12. Insufficient bone marrow function at screening:
    - Absolute Neutrophil Count (ANC) < 1.0 x 10^9/L
    - Hemoglobin (Hgb) < 9.0 g/dL (transfusion support is not permitted within 7 days prior to the first dose of study drug)
    - Platelets < 100 x 10^9/L (transfusion support is not permitted within 7 days prior to the first dose of study drug)
13. Insufficient hepatic or renal function at screening:
    - Total bilirubin > 1.5 x ULN, except for patients with Gilbert's syndrome who are excluded if total bilirubin > 3.0 x ULN and direct bilirubin > 1.5 x ULN
    - Aspartate aminotransferase (AST) or alanine aminotransferase (ALT) > 3 x ULN or > 5 x ULN if liver metastases are present
    - Creatinine clearance for adults < 60 mL/min (calculated using Cockcroft-Gault equation) **For adolescent patients**: Calculated creatinine clearance ≤ 70 mL/min/1.73m^2 (calculated using the Schwartz method).
14. Patients with acute or chronic pancreatitis prior to enrollment; patients with asymptomatic elevated lipase/amylase without radiological evidence of pancreatitis may be included.
15. Presence of Grade ≥ 2 toxicity due to prior cancer therapy according to National Cancer Institute (NCI) Common Terminology Criteria for Adverse Events (CTCAE), with the exception of neuropathy (inclusion of patients with neuropathy of Grade 2 or less is permitted) and alopecia. Patients adequately treated with replacement therapy for endocrinopathies should not be excluded.
16. Any serious uncontrolled acute or chronic infections including a COVID-19 related diagnosis, such as but not limited to those caused by bacteria, viruses, or fungi, confirmed by clinical evidence, imaging, and/or relevant positive laboratory tests (e.g., blood cultures, PCR for DNA/RNA, etc.).
17. Active Hepatitis B (HBV) and / or Hepatitis C (HCV). Patients whose disease is controlled under antiviral therapy are eligible. Testing for HBV or HCV status is not necessary unless clinically indicated or the patient has a history of HBV or HCV infection
18. Any medical condition that would, in the investigator's judgment, prevent the patient's participation in the clinical study due to safety concerns, compliance with clinical study procedures or interpretation of study results.
19. Pregnant or nursing (lactating) women. Women patients should not breast feed while taking study treatment and for 60 days after stopping study treatment.
20. Women of child-bearing potential, defined as all women physiologically capable of becoming pregnant from menarche until becoming post-menopausal, **unless** they are using highly effective methods of contraception (failure rate < 1% per year) while taking study

treatment and for 60 days after stopping study treatment or have had surgical bilateral oophorectomy (with or without hysterectomy), total hysterectomy or bilateral salpingectomy at least six weeks before taking study treatment. In the case of oophorectomy alone, the reproductives status of the woman needs to have been confirmed by follow-up hormone level assessment. Women are considered post-menopausal if they have had 12 months of natural (spontaneous) amenorrhea with an appropriate clinical profile (e.g., hormonal profile confirming menopause and/or age-appropriate history of vasomotor symptoms).

Highly effective contraception methods include:

- Total abstinence (when this is in line with the preferred and usual lifestyle of the patient). Note that periodic abstinence (e.g. calendar, ovulation, symptothermal, post-ovulation methods) and withdrawal are not acceptable methods of contraception.
- Bilateral oophorectomy with or without hysterectomy, total hysterectomy, or bilateral salpingectomy at least six weeks before taking study treatment. In case of oophorectomy alone, only when the reproductive status of the woman has been confirmed by follow up hormone level assessment are they not considered to be of childbearing potential.
- Bilateral tubal occlusion, Bilateral tubal ligation (at least six weeks before taking study treatment).
- Sterilization (vasectomy) of male partner(s) of the female participant at least 6 months prior to screening provided partner(s) has(have) received medical assessment of the surgical success.
- Use of hormonal contraception methods:
    - Combined (estrogen and progestogen containing) hormonal contraception associated with inhibition of ovulation; oral, intravaginal or transdermal.
    - Progestogen-only hormonal contraception (where inhibition of ovulation is not the primary or only mode of action): oral, injectable or implantable.
    - Intrauterine device (IUD) or intrauterine hormone releasing system (IUS)

In case of use of hormonal contraception women should have been stable on the same method for a minimum of 3 months before taking study treatment.

If local regulations are more stringent than the contraception methods listed above, local regulations apply and will be described in the ICF.

21. Sexually active males unwilling to use a condom during intercourse while taking study treatment and for 60 days after stopping study treatment. A condom is required for all sexually active male patients to prevent them from fathering a child AND to prevent delivery of study treatment via seminal fluid to their partner. In addition, male patients must not donate sperm for the time period specified above.

If local regulations deviate from the contraception methods listed above to prevent pregnancy, local regulations apply and will be described in the ICF.

6 Treatment

6.1 Description of study treatment

6.1.1 Investigational drug

For this study, "investigational drug" and "study treatment" refer to single agent DYP688. The investigational drug to be used in this study is described in Table 6-1.

Table 6-1 Investigational drugs

**Treatment Title**	**DYP688**
**Treatment Description**	Every 2 weeks (Q2W)*;
**Type**	Drug
**Dose Formulation**	Lyophilisate in vial<br>(Powder for solution for infusion)
**Unit Dose Strength(s)**	100 mg
**Dosage Level(s)**	4 mg/kg starting dose for Q2W
**Route of Administration**	IV infusion
**Use**	Experimental
**IMP**	Yes
**Sourcing**	Provided centrally by the sponsor
**Packaging and Labeling**	Study treatment will be provided in a carton containing 5 vials. Each carton will be labeled as required per country requirement.

* Based on emerging clinical data, more or less frequent dosing regimens (QW and/or Q4W) may be explored.

The administered dose will be prepared as indicated in the CDYP688A12101 Pharmacy Manual. For dose levels ≤ 24 mg/kg DYP688, the intravenous infusion must be administered over a minimum of 60 minutes. For dose levels > 24 mg/kg DYP688, the intravenous infusion must be administered over a minimum of 2 hours.

In exceptional circumstances when a dosing day must be rescheduled, the next dose of DYP688 may be administered +/- 4 days (for Q2W schedule), +/-2 days (for QW schedule) and +/-7 days (for Q4W schedule), if needed. If the next dose cannot be administered within the forementioned window, then the dose should be skipped. Dosing will be resumed at the next scheduled dose and the visit assessment schedule will be shifted accordingly. For visits when extensive PK sampling is collected (C1D15 and C3D15 for Q2W, C1D8 and C3D8 for QW and C2D1 and C4D1 for Q4W), every effort should be made to complete visits on scheduled days.

6.1.2 Additional study treatments

No auxiliary medicinal products (AxMPs) or other treatment beyond the investigational drug described in Table 6-1 is included in this trial.

In order to determine if premedication is necessary for repeat dosing, patients must not receive premedication to prevent an IRR before the first infusion of DYP688. If a patient experiences a grade 2 IRR, he/she may receive pre-medication on subsequent dosing days after a documented discussion with the Novartis medical monitor. The pre-medication should be chosen per institutional practice at the discretion of the treating physician and may include, but is not limited to acetaminophen/paracetamol (650 mg PO) and diphenhydramine hydrochloride or an equivalent antihistamine (25 to 50 mg PO or IV).

Should patients experience significant infusion reactions in the initial escalation cohorts, based on the frequency and severity of those events, premedication may be considered for all new subsequent patients. Premedication administered should be as per institutional guidelines and may include, but is not limited to acetaminophen/paracetamol (650 mg PO) and diphenhydramine hydrochloride or an equivalent antihistamine (25 to 50 mg PO or IV) and dexamethasone (10 mg PO or IV) or an equivalent corticosteroid before infusion of DYP688.

Acute allergic reactions should be treated as needed per institutional standard of care. In the event of anaphylactic/anaphylactoid reactions, this includes any therapy necessary to restore normal cardiopulmonary status. If a patient experiences a severe anaphylactic/anaphylactoid reaction, the infusion should be discontinued immediately. The patient may only resume study treatment following discussion with Novartis. If a subject experiences a ≥ Grade 3 anaphylactic/anaphylactoid reaction, the subject will be permanently discontinued from the study.

Patients must be treated in a facility equipped for cardiopulmonary resuscitation. Appropriate resuscitation equipment should be available and a physician readily available.

Guidelines on management of DYP688 infusion reactions are provided in Section 6.6.4.

The CTCAE v5.0 category of “Infusion related reaction” must be used to describe DYP688 infusion reactions, unless the investigator considers another category, such as “Allergic reaction”.

6.1.3 Treatment duration

Patients will be treated until they experience unacceptable toxicity, disease progression per RECIST 1.1 as determined by the investigator, withdrawal of consent/opposition to use data/biological samples and/or treatment is discontinued at the discretion of the investigator or the patient, as described in Section 9.2. A complete list of the circumstances requiring study treatment discontinuation is provided in Section 9.1.1.

Patients may continue treatment beyond disease progression after documented discussion with Novartis if, in the judgment of the investigator, there is evidence of clinical benefit and the patient wishes to continue on the study treatment. Criteria for treatment beyond progression are described below.

6.1.3.1 Treatment beyond disease progression

Patients treated with DYP688 may be permitted to continue study treatment beyond initial disease progression per RECIST 1.1 criteria following a documented discussion with the Novartis medical monitor and provided they meet all the following criteria:

- Clinical benefit per investigator's judgement
- Patient exhibits adequate tolerance to study treatment
- Patient performance status is stable
- Absence of symptoms and signs (including worsening of clinically relevant laboratory values) indicating disease progression
- Absence of symptomatic rapid disease progression requiring urgent medical intervention (e.g., symptomatic pleural effusion, spinal cord compression)

Patients who meet the above criteria and continue study treatment beyond initial disease progression per RECIST 1.1 will continue all study procedures as outlined in Table 8-2 and Table 8-3. The reasons for the patients continuing treatment beyond progression will be documented in the electronic case report form (eCRF). In case of clinical deterioration or suspicion of disease progression, a follow-up imaging assessment should be performed promptly rather than waiting for the next scheduled assessment. Patients with evidence of further disease progression on an imaging assessment or who are no longer deriving clinical benefit will be discontinued. Patients are to be informed by the investigator of the option to continue study treatment as well as of other therapeutic options and their benefits, should they exist.

6.2 Other treatment(s)

6.2.1 Concomitant therapy

In general, concomitant medications and therapies deemed necessary for the supportive care (e.g. anti-emetics, anti-diarrheal agents) and safety of the patient are allowed except when specifically prohibited (see Section 6.2.2).

The patient must be told to notify the investigational site about any new medications he/she takes after the start of the study treatment. Medications include not only physician prescribed medications, but also all over-the counter medications, vaccines, herbal medications, food supplements and vitamins.

All medications, procedures, and significant non-drug therapies (including physical therapy and blood transfusions) administered after the patient was enrolled into the study must be recorded on the appropriate eCRFs.

Each concomitant drug must be individually assessed against all exclusion criteria/prohibited medication. If in doubt, the investigator should contact the Novartis medical monitor before enrolling a patient or allowing a new medication to be started. If the patient is already enrolled, contact Novartis to determine if the patient should continue participation in the study.

There is no contraindication for the use of an inactivated, viral-vector-, or mRNA based Sars-CoV-2 vaccine in patients treated with DYP688.

For patients enrolled in this trial who receive a COVID-19 vaccine, which has been permitted (includes full approval, conditional approval, emergency use authorization, provisional/temporary/interim authorization by Health Authority i.e., outside of a COVID-19 clinical trial to evaluate the effectiveness/safety of a COVID-19 vaccine) for use by a local Health Authority, the vaccine will be captured as a concomitant medication and IRB/EC approval and inclusion in Informed Consent are not required. Exceptions may apply if a Health Authority considers the vaccine as "investigational".

Although there is no requirement for patients in this clinical trial to be vaccinated against Sars-CoV-2 prior to study entry, patients are advised to complete vaccination against Sars-CoV-2 at least 2 weeks prior to enrollment. Additionally, patients must avoid receiving the vaccine during the first cycle of treatment (DLT evaluation period).

6.2.1.1 Permitted concomitant therapy requiring caution and/or action

Anticoagulation therapy is permitted if subjects are already at stable doses of oral anticoagulants or stable doses of low molecular weight heparin (LMWH) for > 2 weeks at time of first study treatment dose and in the absence of any clinical evidence of bleeding. Appropriate laboratory parameters, such as the International Normalized Ratio (INR) should be monitored as clinically indicated. Subjects who develop a new requirement for anticoagulant therapy during the conduct of the study may remain on study after documented discussion with the Novartis medical monitor. If medically safe to do so, ongoing anticoagulant therapy should be temporarily discontinued to allow tumor sampling according to the institutional guidelines. If it is not safe to temporarily stop anticoagulant therapy the tumor biopsy sampling should be omitted.

If a patient is using a thrombopoietin mimetic or an erythropoiesis-stimulating agent (ESA) prior to enrollment (at least 2 weeks prior to the first dose of study treatment), they may continue at the same dose.

Treatment with hematopoietic colony-stimulating growth factors (e.g., G-CSF, GM-CSF, M-CSF) and erythropoiesis- or platelet-stimulating agents may not be initiated during the DLT observation window in the dose-escalation part of the study, unless the patient has already experienced a DLT.

Blood/platelet transfusions, the use of bisphosphonates and local steroid therapies (e.g. ophthalmic or inhaled medications), and daily systemic corticosteroid use of ≤ 10 mg/day prednisone (or equivalent) are permitted at the discretion of the treating physician.

The use of systemic steroid therapy and other immunosuppressive drugs is not allowed, with the exception of:

- Prophylactic use for subjects with imaging contrast dye allergy.
- Replacement-dose corticosteroids (defined as 10 mg/day (or lower dose) of prednisone or equivalent dose of other corticosteroids) in the setting of adrenal insufficiency.
- Transient exacerbations of chronic inflammatory conditions such as chronic obstructive pulmonary disease (COPD). Corticosteroids must be reduced to 10 mg/day (or lower dose) of prednisone or equivalent dose of other corticosteroids prior to the next treatment with DYP688.

- Upon treatment of DYP688 infusion reactions or DYP688-related AEs, corticosteroids must be reduced to 10 mg/day (or lower dose) of prednisone or equivalent dose of other corticosteroids prior to the next treatment with DYP688.

Topical, inhaled, nasal and ophthalmic corticosteroids are allowed.

Anti-hypertensives are allowed as concomitant medications; however, because transient hypotension has occurred during infusions of monoclonal antibodies, consideration should be given to withholding anti-hypertensive medications for 12 hours prior to infusion with DYP688.

6.2.2 Prohibited medication

Although QTc prolongation is not expected with DYP688, for this FIH study which includes ECG assessments to assess any potential effect of DYP688 on cardiac function, the use of medications with a known risk of Torsades de Pointes (Section 16.4) should be used with caution and alternative medications should be considered.

The use of systemic steroid therapy (at doses greater than 10 mg/day prednisone or equivalent) and other immunosuppressive drugs is not allowed, see Section 6.2.1.1 for more details regarding exceptions.

During study treatment, subjects must not receive other additional investigational drugs, devices, chemotherapy, or any other therapies that may be active against cancer or that are intended to modulate an immune response. However, limited-field palliative radiotherapy to non-target lesion(s) may be allowed as concomitant therapy after documented discussion with the Novartis medical monitor. Such local therapies administered during the study treatment must be listed on the appropriate CRF. Study treatment must be interrupted during radiotherapy.

The use of live vaccines within 4 weeks of study initiation and throughout the duration of the study is not permitted.

6.3 Preparation, handling, storage and accountability

Each study site will be supplied with study drug in packaging as described Table 6-1 investigational drugs.

Further instructions for the preparation of DYP688 is described in the Pharmacy Manual.

As per the treatment assigned to the patient, investigator staff will select the study treatment to dispense to the patient.

6.3.1 Handling of study treatment

Study treatment must be received by a designated person at the study site, handled and stored safely and properly and kept in a secured location to which only the investigator and designated site personnel have access. Upon receipt, all study treatment must be stored according to the instructions specified on the labels and in the Investigator's Brochure.

Clinical supplies are to be dispensed only in accordance with the protocol. Technical complaints are to be reported to the respective Novartis Country Organization Quality Assurance.

Study treatment labels will be in the local language and comply with the legal requirements of each country. They will include storage conditions for the study treatment but no information about the patient except for the study treatment number.

The investigator or designated site staff must maintain an accurate record of the shipment and dispensing of study treatment in a drug accountability log. Monitoring of drug accountability will be performed by field monitors during site or remote monitoring visits, and at the completion of the trial. The Investigator must also provide accountability for locally sourced materials used for administration (e.g. IV bags).

The site may destroy and document destruction of unused study treatment, drug labels and packaging as appropriate in compliance with site processes, monitoring processes, and per local regulation/guidelines. Otherwise, the investigator will return all unused study treatment, packaging, drug labels, and a copy of the completed drug accountability log to the Novartis monitor or to the Novartis address provided in the investigator folder at each site.

6.4 Patient numbering, treatment assignment, randomization

6.4.1 Patient numbering

Each patient is identified in the study by a patient number (Patient No.), that is assigned when the patient is enrolled for screening and is retained for the patient throughout his/her participation in the trial. A new patient no. will be assigned at every subsequent enrollment if the patient is re-screened. The patient no. consists of the site number (Site No.) (as assigned by Novartis to the investigative site) with a sequential patient number suffixed to it, so that each patient's participation is numbered uniquely across the entire database. Upon signing the informed consent form, the patient is assigned to the next sequential Patient No. available.

6.4.2 Treatment assignment, randomization

No randomization will be performed in this study. The assignment of a patient to a particular cohort will be coordinated by the sponsor.

6.4.2.1 Replacement policy

Phase I – Dose Escalation part

Patients will not be replaced on study. However, if a patient is considered as non-evaluable for the escalation decisions, enrollment of a new patient to the current cohort will be considered if there is less than the required number of evaluable patients. Enrollment of new patients may be considered until at least the minimum number (2 or 3) or at most the maximum number (3 or 6) of evaluable patients is achieved within the cohort. Minimum and maximum numbers of evaluable patients per cohort are defined in the guidelines for dose escalation and determination section.

Phase II part

During the Phase II part, no replacements will be allowed.

6.5 Treatment blinding

Not applicable.

6.6 Dose escalation and dose modification

6.6.1 Dose escalation guidelines

6.6.1.1 Starting dose

The starting dose of DYP688 is 4 mg/kg administered intravenously once every 2 weeks (Q2W).

6.6.1.2 Provisional dose levels

Table 6-2 describes the starting dose and the provisional dose levels that may be evaluated during this trial on the Q2W dosing schedule

Actual dose levels of DYP688 and the starting dose for any alternate dosing schedules such as weekly or monthly (QW or Q4W) dosing will be determined based on available data, including toxicity and as applicable, PK and PD information, guided by the BLRM (or BHLRM) along with EWOC, and following a discussion with participating investigators during dose escalation meetings (refer to Section 4.2). It is possible for additional higher and/or intermediate dose levels to be explored during the course of the study. Cohorts may be added at any dose level below the MTD in order to better understand safety, PK, or PD as shown in Table 6-2.

Table 6-2 Provisional dose levels for DYP688 single agent

Dose level	Proposed daily dose*	Increment from previous dose
-2**	1 mg/kg	-50%
-1**	2 mg/kg	-50%
1	4 mg/kg	(starting dose)
2	8 mg/kg	100%
3	12 mg/kg	50%
4	16 mg/kg	33%
5	24 mg/kg	50%
6	32 mg/kg	33%

* Dose levels represent total daily doses administered at the Q2W dosing schedule

**If dose de-escalation from starting dose level is required, dose level -1 and -2 represent treatment dose level at which a new cohort may be enrolled. Dose reduction below dose level -2 is not permitted for this study.

6.6.2 Guidelines for dose escalation and determination of MTD/RD

Dose escalation is conducted in order to establish the recommended dose and regimen for the single agent treatments that will be used in the phase II part of the study. Specifically, the RD represents the dose and schedule for a particular study treatment that the Investigators and Novartis believe has the most appropriate benefit-risk ratio as assessed by the review of the safety, tolerability, PK, preliminary efficacy, and PD effect, taking into consideration the MTD.

The MTD is the highest dose estimated to have less than 25% risk of causing a DLT during the DLT evaluation period in more than 33% of treated patients. The dose(s) selected for the phase II part can be any dose equal to or less than the MTD, and may be declared without identifying the MTD.

For the purposes of dose escalation decisions, the first cohort (dose level one) of DYP688 will consist of 2 to 6 enrolled patients. If any subject experiences a DLT during the DLT evaluation period for the first cohort (dose level one), the minimum cohort size will be increased to at least three. All other dose escalation cohorts will consist of 3 to 6 enrolled patients. Patients must have adequate exposure and follow-up to be considered evaluable for dose escalation decisions (Section 12.1) for criteria in the definition of Dose Determining Set). If one or more patients discontinue and fail to meet the evaluability criteria, the replacement policy (Section 6.4.2.1) may be used to enroll additional patients to the same cohort, in order to support the benefit-risk assessment.

For the first three patients in each cohort, where a previously untested, higher dose level is administered, during the dose escalation part, a staggered approach will be utilized for dosing and will occur as follows:

- 1st patient dosed, wait at least 24 hours
- 2nd patient dosed, wait at least 24 hours
- 3rd patient dosed

In countries where required by the health authority, the safety monitoring interval between each patient dosed will be 48 hours for all patients in each dose escalation cohort. Please refer to Section 12.8 for further details on cohort size during dose escalation.

Dose escalation decisions will be made when all patients in a cohort have completed the DLT evaluation period or discontinued. Decisions will be made by Investigators and Novartis study personnel in a dose-escalation meeting and will be based on a synthesis of all relevant data available from all dose levels evaluated in the ongoing study, including safety information, PK, available PD and preliminary efficacy.

Any dose escalation decisions made by investigators and Novartis personnel will not exceed the highest dose level satisfying EWOC principle by BLRM. In all cases, the dose for the next cohort will not exceed a 100% increase from a previously tested dose level that was determined to be safe. In addition, if more than one dosing regimen is evaluated for DYP688, the total cycle dose of the starting dose on the new regimen cannot exceed that of the highest dose previously tested and shown to be safe and tolerable in a prior regimen and meet the EWOC criteria. Smaller increases in dose and/or lower starting doses for new regimens may be recommended by the investigators and Novartis upon consideration of all of the available clinical data.

To better understand the safety, tolerability, PK, PD, or preliminary anti-tumor activity of DYP688 before or while proceeding with further escalation, additional cohorts of 1 to 6 patients may be enrolled at any dose level at or below the highest dose previously tested and shown to be safe.

To reduce the risk of exposing patients to an overly toxic dose, in the event that 2 patients experience a DLT in a new cohort, the BLRM will be updated with the most up-to-date new

information from all cohorts, without waiting for all patients from the current cohort to complete the evaluation period

- If the two DLTs occur in an escalation cohort, enrollment to that cohort will stop, and the next cohort will be opened at a lower dose that satisfy the EWOC criteria.
- If the two DLTs occur in an additional cohort, then upon re-evaluation of all relevant data, additional patients may be enrolled into the open cohorts only if the EWOC criteria is still satisfied. Alternatively, if recruitment to the same dose cannot continue, a new cohort of patients may be recruited to a lower dose that satisfies the EWOC principle.

In the event that a dose is not deemed acceptable for a newly enrolled patient, ongoing patients may continue treatment at that dose level at the discretion of the investigator and Novartis if it is in the best interest of the patient and is well tolerated.

Besides the scenario of 2 DLTs, the current dose being tested may be de-escalated based on new safety findings, including but not limited to observing a DLT, before a cohort is completed. Subsequent to a decision to de-escalate, re-escalation may occur if data in subsequent cohorts supports this (EWOC criteria are satisfied).

All dose decisions must be agreed by Investigators and Novartis study personnel.

6.6.2.1 Implementation of dose escalation decisions

To implement dose escalation decisions, the available toxicity information (including adverse events and laboratory abnormalities that are not DLTs), the assessment of risk to future patients from the BLRM (or BHLRM) along with the EWOC principle, and the available PK and PD information will all be evaluated by the Investigators and Novartis study personnel (including the study physician and statistician) during a dose decision meeting. Drug administration at the next higher dose level may not proceed until the investigator receives written confirmation from Novartis indicating that the results of the previous dose level were evaluated and that it is permissible to proceed to a higher dose level.

6.6.2.2 Intra-patient dose escalation

Intra-patient dose escalation is not permitted at any time within the first four cycles of treatment. After the fourth cycle is completed, individual patients may be considered for treatment at a dose of DYP688 higher than the dose to which they were initially assigned. In order for a patient to be treated at a higher dose of DYP688, he or she must have tolerated the lower dose for at least four cycles of therapy (i.e. he or she must not have experienced any DYP688-related toxicity CTCAE grade ≥ 2 at the lower dose originally assigned). Moreover, the new, higher dose with which the patient is to be treated must be a dose that has completed evaluation and shown to satisfy the EWOC principle. There is no limit to the number of times a patient may have his or her dose of DYP688 increased. For any further increases after the initial intra-patient dose escalation, the following rules apply: The patient must not have experienced any CTCAE grade ≥ 2, DYP688-related toxicity over at least two cycles of therapy at the lower dose, and the higher dose being considered must have been fully evaluated and shown to satisfy the EWOC principle. Consultation and agreement with Novartis must occur prior to any intra-patient dose escalation occurring. Data from the first cycle of treatment at the new dose level will not be formally included into the statistical model describing the relationship between dose

and occurrence of DLT. However, the data will be incorporated into the clinical assessment of safety within a dose escalation meeting.

6.6.3 Definitions of dose limiting toxicities (DLTs)

A dose-limiting toxicity (DLT) is defined as an adverse event or abnormal laboratory value that occurs within the DLT evaluation period with DYP688 and that is not primarily related to disease, disease progression, intercurrent illness, or concomitant medications and meets any of the criteria included in Table 6-3. The DLT evaluation period is defined as the first cycle of treatment (28 days). The National Cancer Institute Common Terminology Criteria for Adverse events (NCI CTCAE) version 5.0 will be used for all grading. For the purpose of dose-escalation decisions, DLTs will be considered and included in the BLRM.

The investigator must notify the Sponsor immediately of any unexpected CTCAE grade ≥ 3 AEs, laboratory abnormalities, or any AEs meeting the DLT criteria. Prior to enrolling patients into a higher dose level, CTCAE grade ≥ 2 AEs will be reviewed for all patients at the current dose level.

Table 6-3 Criteria for defining dose-limiting toxicities in all patients

**Any Grade 3 or Grade 4 AEs (CTCAE v5.0) related to study treatment occurring during Cycle 1 are DLTs, EXCEPT:**	
General disorders	Grade 3 fatigue that resolves to ≤ Grade 1 within 7 days.
Vascular disorders	Grade 3 hypertension (not to exceed a systolic blood pressure of 180 mmHg) that can be controlled (to BP <140/90) within 7 days after starting or optimizing anti-hypertensive therapy.
Skin and subcutaneous tissue disorders	Grade 3 non bullous rash without epidermal detachment that resolves to ≤ Grade 1 within 7 days of starting optimal skin toxicity treatment.
Gastrointestinal disorders	Grade 3 nausea and vomiting that resolves to ≤ Grade 1 within 48 hours of starting optimal anti-emetic therapy.
	Grade 3 diarrhea that resolves within 72 hours after starting optimal anti-diarrhea treatment, where colitis is not suspected.
	Asymptomatic Gr 3 amylase or lipase increase in the absence of radiological evidence of pancreatitis that resolves within 14 days.
Blood and lymphatic system disorders	Grade 3 anemia that resolves within 7 days in the absence of transfusion.
	Grade 3 neutropenia without fever or other clinical symptoms that resolves to ≤ Grade 1 within 7 days. Grade 3 leukopenia without fever or other clinical symptoms that resolves to ≤ Grade 1 within 7 days.
	Grade 3 thrombocytopenia without clinically significant bleeding.
	Lymphopenia of any grade unless clinically significant.
Immune-related disorders[1] (unless otherwise specified in this table)	Grade 3 immune-related AE that resolves to ≤ Grade 1 within 7 days of starting appropriate treatment.

Metabolic and nutrition disorders	Grade 3 electrolyte abnormalities that resolve to ≤ Grade 1 within 7 days after starting corrective treatment.
Events not described above	Grade 3 asymptomatic increase in creatine kinase that resolves within 14 days in the absence of evidence of cardiac involvement.
	Grade 3 laboratory investigations that are not considered clinically significant by the investigator
**The following additional AEs related to study treatment <u>are considered DLTs</u>:**	
Eye disorders	Grade 2 eye pain or reduction of visual acuity if it does not improve to Grade 1 within 14 days of the initiation of topical therapy, OR if it requires systemic treatment.
Respiratory, thoracic and mediastinal disorders	Grade 2 pneumonitis if it does not resolve to ≤ Grade 1 within 7 days of starting corticosteroids.
Cardiac disorders	Grade 2 myocarditis.
Gastrointestinal disorders	Grade 2 colitis if it does not resolve to ≤ Grade 1 within 7 days despite treatment with corticosteroids.
Hepatic	Grade 2 isolated total bilirubin[2] (> 1.5 – 3.0 x ULN) for > 14 days if baseline was normal<br>Grade 2 isolated bilirubin (>1.5 – 3.0 x baseline) for > 14 days if baseline was abnormal<br>For patients with normal baseline ALT and AST and total bilirubin value: Grade 2 AST or ALT (> 3.0 x ULN) combined[3] with total bilirubin > 2.0 x ULN without evidence of cholestasis[4]<br>OR<br>For patients with abnormal baseline AST or ALT or total bilirubin value:<br>AST or ALT > 2 x baseline AND > 3.0 x ULN<br>OR<br>AST or ALT > 8.0 x ULN, whichever is lower, combined with total bilirubin > 2 x baseline AND > 2.0 x ULN
Skin and subcutaneous tissue disorders	Grade 2 bullous disease that does not resolve to ≤ Grade 1 within 7 days of starting corticosteroids.<br>Stevens Johnson Syndrome is always considered a DLT
Other adverse events	Other clinically significant toxicities, including a single event or multiple occurrences of the same event that lead to a dosing delay of > 7 days in cycle 1 or a dose reduction, may be considered to be DLTs by the investigators and Novartis,

[1] Depending on the nature of the AE, there may be cases where immune-related Grade 2-3 AEs of any duration warrant declaration of a DLT and permanent study discontinuation. DLT determination not already outlined in this table will be made on a case-by case basis after investigator discussion with the Novartis Medical Monitor.

[2] Isolated total bilirubin" increase defined as total bilirubin increase without ALT or AST increase; "isolated AST or ALT" increase defined as AST or ALT increase without total bilirubin increase

[3] Combined defined as total bilirubin increase to the defined threshold concurrently with ALT/AST increase to the defined threshold

[4] Cholestasis" defined as ALP elevation [>2.0 x ULN and R value < 2] in patients without bone metastasis, or elevation of ALP liver fraction in patients with bone metastasis

Note: The R value is calculated by dividing the ALT by the ALP, using multiples of the ULN for both values. It denotes whether the relative pattern of ALT and/or ALP elevation is due to cholestatic (R ≤ 2), hepatocellular (R ≥ 5), or mixed (R >2 and < 5) liver injury

6.6.4 Dose modifications

For subjects who do not tolerate the protocol-specified dosing regimen, dose adjustments are permitted in order to allow the subject to continue the study treatment. All dose modifications should be based on the worst preceding toxicity (CTCAE version 5.0). All interruptions or changes to study drug administration must be recorded on the Study treatment Administration eCRF.

For toxicities considered to be at least possibly related to DYP688 treatment, the criteria for dose modification of DYP688 are listed in Table 6-4. If more than one cycle of DYP688 has to be skipped due to DYP688-related toxicity, the patient should be discontinued from study treatment. If the patient is experiencing clinical benefit, and in the opinion of the investigator it is in the patient's best interest to remain on study, then the patient may continue treatment on a case by case basis after discussion with the Novartis medical monitor (Section 6.1.3).

If the investigator considers it to be in the patient's best interest to resume therapy prior to resolution to grade 1 or to resume without dose modification, this may be permitted following discussion with the Novartis medical monitor. If treatment is resumed at the same dose, and the toxicity recurs (or continues) with the same or increased severity, the dose must be reduced. If study treatment with DYP688 is resumed at a lower dose, and the same toxicity re-occurs (or continues) with the same or increased severity, study treatment should be discontinued for this patient. However, if the patient is experiencing clinical benefit, a dose reduction may be permitted following discussion with the Novartis medical monitor.

In the absence of meeting dose modification criteria, patients may be allowed to switch to a less frequent dosing schedule either due to toxicity on the more frequent dosing schedule, or due to patient convenience to reduce the burden of study visits after completing 4 cycles of study treatment. Patients on the QW schedule will be allowed to switch to the Q2W schedule at a lower or the same dose level, after documented discussion with Novartis.

For each patient, once a dose level reduction has occurred due to intolerability at the higher dose level, the dose level may not be re-escalated during subsequent treatment cycles with DYP688. Up to 2 dose reductions are permitted per event. If a patient requires more than 2 dose reductions, then study treatment should be discontinued for this patient. No dose reduction below 1 mg/kg is permitted for this study.

Adverse events not included in the study protocol or the reference guidance documents should be managed per institutional preference.

Table 6-4 Criteria for dose reduction / interruption and re-initiation of DYP688 treatment for adverse drug reactions

Worst toxicity CTCAE[1] grade	Recommended Dose Modification
**Infusion reaction or hypersensitivity reaction**	
Grade 1	Decrease infusion rate until recovery from the symptoms.
Grade 2	Stop infusion immediately, and keep IV line open.<br>Follow institutional guidelines for the management and follow-up of infusion reaction.<br>Restart infusion at 50% of previous rate under continuous observation. Ensure that there is a minimum observation period of 1 hour prior to restarting the infusion.<br>If the AE recurs at the reinitiated slow rate of infusion, and despite oral pre-medication, then permanently discontinue study treatment.
Grade 3 and Grade 4	Discontinue infusion immediately and discontinue study treatment.<br>Provide supplemental oxygen, fluids, and other resuscitative measures as needed. Monitor vital signs (e.g., blood pressure, pulse, respiration, and temperature) every 15 ± 5 minutes until resolution.
**Cytokine Release Syndrome (CRS)**	
Grade 2	See instructions for Grade 2 Infusion Reaction above.
Grade 3 or Grade 4	Discontinue study treatment.<br>Follow-up CRS as per institutional guidelines.
**Ocular (uveitis, eye pain)**	
Grade 1	Continue study treatment without dose modification.<br>Ophthalmology consultation.
Grade 2	Hold study treatment.<br>Urgent ophthalmology consultation.<br>Upon resolution to ≤ Grade 1 may consider resuming study treatment without dose reduction after discussion with the Novartis Medical Monitor and in consultation with ophthalmology.
Grade 3 or Grade 4	Discontinue study treatment.<br>Urgent ophthalmology consultation.
**Blurred vision**	
Grade 1	Continue study treatment without dose modification.<br>Ophthalmology consultation.
Grade 2	Hold study treatment.<br>Urgent ophthalmology consultation<br>If resolves to ≤ Grade 1 in ≤ 14 days resume study treatment without dose reduction,<br>If remains Grade 2 for > 14 days resume study treatment with reduced dose

Worst toxicity CTCAE[1] grade	Recommended Dose Modification
Grade 3	Hold study treatment.<br>Urgent ophthalmology consultation<br>If resolves to ≤ Grade 1 in ≤ 14 days resume study treatment with reduced dose<br>If remains Grade 3 for > 14 days discontinue study treatment
Grade 4	Discontinue study treatment<br>Urgent ophthalmology consultation
**Pulmonary (pneumonitis)**	
Grade 1	Consider study treatment hold.<br>Manage per institutional practice.<br>Consider resuming study treatment upon radiographic evidence of improvement.
Grade 2	Hold study treatment.<br>Pulmonary and infection workup.<br>If resolved to Grade 1 ≤ 7 days resume study treatment without dose reduction, if remains Grade 2 for > 7 days discuss with Novartis Medical Monitor prior to resuming study treatment
Grade 3 or Grade 4	Discontinue study treatment.
**Cardiovascular**	
**ECG QTc-Interval prolonged[5]**	
Grade 3 (average QTcF of triplicate ECGs)	Hold study treatment.<br>Urgent consultation with cardiologist of qualified specialist.<br>Repeat ECGs as clinically indicated until QTcF returns to <500msec.<br>Upon resolution to Grade ≤ 1 or baseline, QTc or < 30 msec difference from baseline QTcF within ≤ 7 days, may resume study treatment without dose modification after discussion with the Novartis Medical Monitor. If remains Grade 2 for > 7 days discuss with Novartis Medical Monitor prior to resuming study treatment. Baseline ECG refers to the ECG(s) collected at screening.
Grade 4	Discontinue study treatment.
**Hypertension**	
Grade 3	Hold study treatment<br>Upon resolution to Grade ≤ 1 or baseline within ≤ 7 days, may resume study treatment without dose modification after discussion with the Novartis Medical Monitor
Grade 4	Discontinue study treatment
**Other cardiovascular disorders**	
Grade 2 (except myocarditis)	Hold study treatment.<br>Upon resolution to Grade ≤ 1 or baseline, may resume study treatment without dose modification after discussion with the Novartis Medical Monitor.

Worst toxicity CTCAE[1] grade	Recommended Dose Modification
Grade 2 myocarditis, or Grade ≥ 3 other cardiac disorders related to study treatment	Discontinue study treatment.
**Hypotension**	
Grade 3 Hypotension or Grade 2 hypotension with symptoms:	Hold study treatment and suspend any concomitant medications that may exacerbate hypotension until it resolves to ≤ Grade 1. Manage as per standard of care at the local institution, which may include supplementation with intravenous fluids<br>Monitor the patient with serial vital sign measurements and ECGs until hypotension resolves to ≤ Grade 1 then restart DYP688 at one lower dose level. If findings recur on lower dose, omit dose of DYP688 until resolves to ≤ Grade 1 or baseline and restart DYP688 at next lower dose level. If findings recur again discontinue
**Gastrointestinal (colitis[2])**	
Grade 1	May continue study treatment without dose modification. Manage per institutional standard guidelines which should include anti-diarrheal treatment, consideration of corticosteroid therapy, and hydration.
Grade 2	Hold study treatment.<br>GI consultation.<br>Upon resolution to ≤ Grade 1 and tapering of steroid requirement to ≤ 10 mg prednisone per day, may resume study treatment without dose modification
Grade 3	Hold study treatment.<br>GI consultation.<br>Upon resolution to ≤ Grade 1 and tapering of steroid requirement to ≤ 10 mg prednisone per day, may resume study treatment after discussion with the Novartis Medical Monitor.
Grade 4	Discontinue study treatment.
**Nausea, Vomiting, and/or Diarrhea**	
Grade 2	Grade 2 ≥72 hours despite optimal anti-emetic and/or anti-diarrheal treatment:<br>Anti-diarrheal medication is recommended at the first sign of abdominal cramping, loose stools or overt diarrhea<br>Hold study treatment until resolved to ≤ grade 1, then maintain dose level.<br>If findings recur, omit study treatment until resolved to ≤ grade 1, and then restart one dose level lower
Grade 3	Hold study treatment<br>For ≥ grade 3 vomiting or grade 3 nausea only if the vomiting or nausea cannot be controlled with optimal antiemetic (as per local practice)<br>Hold study treatment until resolved to ≤ grade 1, then restart one dose level lower
Grade 4	Discontinue study treatment.

Worst toxicity CTCAE[1] grade	Recommended Dose Modification
**AST and/or ALT elevation**	
Grade 2 AST and/or ALT	Hold study treatment.<br>Manage per institutional practice.<br>Upon resolution to ≤ Grade 1 or baseline, consider resuming study treatment without dose modification.
Grade 2 transaminitis with bilirubin >2 x ULN	Discontinue study treatment.
Grade 3 AST and/or ALT	Hold study treatment.<br>Manage per institutional practices.<br>Upon resolution to ≤ Grade 1 or baseline within 72 hours, consider resuming study treatment without dose modification after discussion with the Novartis Medical Monitor.<br>Otherwise, discontinue study treatment.
Grade 4 AST and/or ALT	Discontinue study treatment.
**Isolated total bilirubin elevation[3]**	
Grade 2	Hold study treatment.<br>Upon resolution to ≤ Grade 1, may continue study treatment without dose modification.
Grade 3	Hold study treatment.<br>Upon resolution to ≤ Grade 1, may consider resuming study treatment at a reduced dose after discussion with the Novartis Medical Monitor.
Grade 4	See footnote[3]. Otherwise, discontinue study treatment.
**Amylase and/or lipase elevation[4]**	
Grade 3 (> 5 xULN), not associated with symptoms or clinical manifestations of pancreatitis[4]<br><br>For Grade 3 (>2 – 5 x ULN) with symptoms	Continue study treatment.<br>If levels do not resolve to ≤ Grade 2 within ≤ 14 days, hold study treatment.<br>Upon resolution to ≤ Grade 2, may resume study treatment without dose modification, after discussion with the Novartis Medical Monitor.<br><br>Hold study treatment.<br>Upon resolution to ≤Grade 2 within ≤21 days, may resume study treatment at a reduced dose, after discussion with the Novartis Medical Monitor.<br>If remains Grade 2 for >21 days, discontinue study treatment
**Pancreatitis**	
Grade 2/radiologic evidence	Hold study treatment.<br>Manage per institutional practice.<br>Upon resolution to ≤ Grade 1, may resume study treatment without dose modification, if no clinical evidence of pancreatitis and after discussion with the Novartis Medical Monitor.
Grade 3 or Grade 4	Discontinue study treatment.

Worst toxicity CTCAE[1] grade	Recommended Dose Modification
**Renal**	
**Serum creatinine**	
Grade 2	Hold study treatment.<br>Manage per institutional practice.<br>Upon resolution to ≤ Grade 1, may resume study treatment without dose modification after discussion with the Novartis Medical Monitor.
Grade 3 or 4	Discontinue study treatment.
**Musculoskeletal**	
Grade 2 or Grade 3	Hold study treatment.<br>Consider resuming study treatment without dose modification upon resolution to ≤ Grade 1 with appropriate management.
Grade 4	Discontinue study treatment.<br>In some cases, resuming study treatment may be considered after documented discussion with the Novartis Medical Monitor and consultation with a rheumatologist.
**Endocrine**	
**Hypothyroidism or hyperthyroidism**	
Grade 2	May continue study treatment without dose modification.<br>Management according to institutional practice.
Grade 3	Hold study treatment.<br>Upon resolution to Grade ≤ 1 with appropriate management, may resume study treatment without dose modification.
Grade 4	May resume study treatment following resolution to Grade ≤ 1 after control with physiologic hormone replacement.
**Other endocrine disorders**	
Grade 2 and Grade 3	Hold study treatment.<br>Upon resolution to Grade ≤ 1 with appropriate management, may resume study treatment without dose modification.
Grade 4	Hold study treatment.<br>Grade 4 treatment-related endocrinopathies, such as adrenal insufficiency, adrenocorticotropic hormone<br>(ACTH) deficiency, or glucose intolerance, which resolve or are adequately controlled with physiologic hormone replacement (corticosteroids) or glucose-controlling agents, respectively, may not require discontinuation after discussion with and approval from the Novartis Medical Monitor.
**Neurology**	
Grade 1	Consider study treatment hold, particularly for clinical suspicion of Guillain-Barre syndrome, encephalitis, aseptic meningitis, transverse myelitis, or peripheral neuropathy.
Grade 2	Hold study treatment.<br>In some cases, resuming study treatment may be<br>Considered after discussion with the Novartis Medical Monitor.
Grade 3 or Grade 4	Discontinue study treatment.

Worst toxicity CTCAE[1] grade	Recommended Dose Modification
**Dermatology (rash)**	
Grade 1	Continue study treatment without dose modification. Topical steroids, antihistamines, topical emollients
Grade 2	Consider holding study treatment.<br>Topical or oral corticosteroids, antihistamines.<br>If study treatment is held and upon resolution to ≤ Grade 1, resume study treatment without dose modification.
Grade 3 or Grade 4	Hold study treatment.<br>Manage per institutional practice.<br>Upon resolution to ≤ Grade 1, consider resuming study treatment after discussion with the Novartis Medical Monitor.
Bullous dermatitis	Hold study treatment.<br>Grade 1-2 bullous dermatitis: consider resuming study treatment after discussion with the Novartis Medical Monitor<br>Grade 3 bullous dermatitis: consider resuming therapy after expert consultation and documented discussion with the Novartis medical monitor.<br>Grade 4 bullous dermatitis: discontinue study treatment
Stevens-Johnson syndrome (SJS), or Lyell syndrome/toxic epidermal necrolysis (TEN)	Suspected – Hold study treatment<br>Confirmed – Permanently discontinue study treatment
**Hematology**	
**Neutropenia (ANC)**	
Grade 3 or Grade 4	Hold study treatment.<br>Upon resolution to ≤ Grade 2 or baseline within ≤ 7 days, may resume study treatment without dose modification, after discussion with the Novartis Medical Monitor. If resolves to Grade 2 in > 7 days, discuss with Novartis Medical Monitor prior to resuming study treatment.
**Febrile neutropenia**	
Grade 3 or Grade 4	Hold study treatment.<br>Upon resolution of fever and improvement of neutropenia to ≤ Grade 2 or baseline, may resume study treatment without dose modification, after discussion with the Novartis Medical Monitor.
**Thrombocytopenia**	
Grade 3	Hold study treatment.<br>Upon resolution to ≤ Grade 2 or baseline, resume study treatment without dose modification.<br>For Grade 3 associated with major bleeding, discontinue study treatment.
Grade 4	Discontinue study treatment.

Worst toxicity CTCAE[1] grade	Recommended Dose Modification
**Anemia**	
Grade 3 or Grade 4	Hold study treatment.<br>Upon resolution to ≤ Grade 2 or baseline within ≤ 7 days, may resume study treatment without dose modification, after discussion with the Novartis Medical Monitor. If resolves to Grade 2 in > 7 days, discuss with Novartis Medical Monitor prior to resuming study treatment.
**Lymphopenia**	
Any grade	Treatment-related lymphopenia does not require study treatment hold or discontinuation.
**Other laboratory adverse events, not specified elsewhere in table**	
Grade 3 or Grade 4	Hold study treatment.<br>Upon resolution to ≤ Grade 1, resume study treatment without dose modification.<br>Isolated Grade 4 electrolyte abnormalities not associated with clinical sequelae and corrected after appropriate management within 72 hours of their onset do not require discontinuation.<br>In the case of Grade 4 electrolyte abnormalities associated with clinical sequelae, or not resolved to ≤ Grade 1 within 72 hours despite appropriate management, discontinue study treatment.
**Other non-laboratory adverse events, not specified elsewhere in table**	
Grade 2	Consider study treatment hold, at investigator discretion.<br>Upon resolution to ≤ Grade 1, resume study treatment without dose modification.
Grade 3	Hold study treatment.<br>Upon resolution to ≤ Grade 1, resuming study treatment must be discussed with the Novartis Medical Monitor.
Grade 4	Discontinue study treatment.

All dose modifications should be based on the worst preceding toxicity.

[1] Common Toxicity Criteria for Adverse Events (CTCAE)

[2] Anti-diarrheal medication is recommended at the first sign of abdominal cramping, loose stools or overt diarrhea.

[3] If Grade 3 or 4 hyper-bilirubinemia is due to the indirect (non-conjugated) component only, and hemolysis as the etiology has been ruled out as per institutional guidelines (e.g., review of peripheral blood smear and haptoglobin determination), then delay study treatment until resolved ≤ Grade 1, and resume study treatment at the discretion of the investigator.

[4] A CT scan or other imaging study to assess the pancreas, liver, and gallbladder must be performed within one week of the first occurrence of any ≥ Grade 3 of amylase and/or lipase.

[5] For all prolonged QTcF grades check quality of ECGs, check serum electrolytes, correct with supplementation as appropriate and repeat until within normal range.

6.6.5 Follow-up for toxicities

Patients whose treatment is interrupted or permanently discontinued due to an adverse event or clinically significant laboratory value, must be followed up at least once a week (or more frequently if required by institutional practices, or if clinically indicated) for 4 weeks, and

subsequently at approximately 4-week intervals or as clinically indicated, until resolution or stabilization of the event, whichever comes first. Appropriate clinical experts such as ophthalmologist, endocrinologist, dermatologist, cardiologists, etc., should be consulted as deemed necessary. Further guidelines and recommendations for the management of specific study treatment-related toxicities (e.g., ophthalmological toxicity) are provided in the DYP688 Investigator Brochure. All patients must be followed up for adverse events and serious adverse events for 60 days (+7 days) from the last dose of DYP688.

6.7 Additional treatment guidance

6.7.1 Treatment compliance

All treatment infusions will be administered in the clinic. This information must be captured in the source document, the appropriate CRF/s and in the Drug Accountability Log. Pharmacokinetic parameters (measures of treatment exposure) will be determined in all patients treated with DYP688, as detailed in Section 8.5.1.

7 Informed consent procedures

The Investigator or his/her representative will explain the nature of the study, including the risks and benefits, to the patient or their legally authorized representative (and answer all questions regarding the study).

Patients must be informed that their participation is voluntary. Patients or their legally authorized representatives will be required to sign a statement of informed consent that meets the requirements of 21 CFR 50, local regulations, ICH guidelines, privacy, and data protection, where applicable, and the IRB/IEC or study center.

Eligible patients may only be included in the study after providing (witnessed, where required by law or regulation), IRB/IEC-approved informed consent.

Informed consent must be obtained before conducting any study-specific procedures (e.g. all of the procedures described in the protocol). The process of obtaining informed consent must be documented in the patient source documents.

The medical record must include a statement that written informed consent was obtained before the patient was enrolled in the study and the date the written consent was obtained. The authorized person obtaining the informed consent must also sign the ICF.

A copy of the ICF(s) must be provided to the patient or their legally authorized representative.

If applicable, in cases where the patient's representative(s) gives consent (if allowed according to local requirements), the patient must be informed about the study to the extent possible given his/her level of understanding. If the patient is capable of doing so, he/she must indicate agreement by personally signing and dating the written informed consent document. Patients who are rescreened are required to sign a new ICF. Minor patients must be re-consented if they reach the age of majority during the course of the study, in order to continue participating.

Novartis will provide to investigators in a separate document a proposed informed consent form that complies with the ICH E6 GCP guidelines and regulatory requirements and is considered

appropriate for this study. Any changes to the proposed consent form suggested by the investigator must be agreed by Novartis before submission to the IRB/IEC.

Information about common side effects already known about the investigational treatment can be found in the Investigator's Brochure (IB). This information will be included in the patient informed consent and should be discussed with the patient during the study as needed. Any new information regarding the safety profile of the investigational drug that is identified between IB updates will be communicated as appropriate, for example, via an investigator notification or an aggregate safety finding. New information might require an update to the informed consent and then must be discussed with the patient. Information on any change in risk and/or change in scope must be provided to patients already actively participating in the study, and they must read, understand, and sign each revised ICF confirming their willingness to remain in the study.

As per Section 4.5, during a Public Health emergency as declared by Local or Regional authorities i.e. pandemic, epidemic or natural disaster, that may challenge the ability to obtain a standard written informed consent during the study due to limits that prevent an on-site visit, Investigator may conduct the informed consent discussion remotely (e.g. telephone, videoconference) if allowable by a local Heath Authority. Guidance issued by local regulatory bodies on this aspect prevail and must be implemented and appropriately documented (e.g. the presence of an impartial witness, sign/dating separate ICFs by trial patient and person obtaining informed consent, etc.).

The following informed consents are included in this study:

- Main study consent, which also included:
  - A subsection that requires a separate signature for the 'Optional Consent for Additional Research' to allow future research on data/samples collected during this study
  - Optional consent for activities that may be done outside of the study site
- As applicable, Pregnancy Outcomes Reporting Consent for female patients or the female partners of any male patients who took study treatment
- As applicable, Main study Adolescent assent, which also included:
  - Patient information specific to the pediatric population to be enrolled in the study
- As applicable, Main study parent/legal guardian consent, which also included:
  - A subsection that requires a separate signature for the 'Optional Consent for Additional Research' to allow future research on data/samples collected during this study
  - Optional consent for activities that may be done outside of the study site

Women of child bearing potential must be informed that taking the study treatment may involve unknown risks to the fetus if pregnancy were to occur during the study and agree that in order to participate in the study they must adhere to the contraception requirements.

Male patients must be informed that if a female partner becomes pregnant while he is enrolled in the study, contact with the female partner will be attempted to request her consent to collect pregnancy outcome information.

The study includes the option for the patient to have certain study procedures performed off-site instead of at the study site during a declared public health emergency for which a separate signature is required if the patient agrees. It is required as part of this protocol that the Investigator presents this option to the patient, as permitted by national and local governing regulations. The process for obtaining consent should be exactly the same as described above for the main informed consent.

A copy of the approved version of all consent forms must be provided to Novartis after IRB/IEC approval.

8 Visit schedule and assessments

The Assessment Schedule (Table 8-2 and Table 8-3) lists all of the assessments when they are performed. All data obtained from these assessments must be supported in the patient's source documentation.

Patients should be seen for all visits/assessments as outlined in the assessment schedule (Table 8-2 and Table 8-3) or as close to the designated day/time as possible. Screening evaluations must be performed ≤ 21 days before Cycle 1 Day 1, with the exception of tumor assessments, that must be performed ≤ 28 days before Cycle 1 Day 1. Laboratory assessments performed as part of the screening evaluations and within 72 hours of the first dose of study treatment (or within 72 hours of the dose of study treatment), are not required to be repeated on the first dosing day. Refer to Table 8-1 for all allowable visit windows.

Missed or rescheduled visits should not lead to automatic discontinuation. Patients who prematurely discontinue the study for any reason should be scheduled for a visit as soon as possible, at which time all of the assessments listed for the final visit will be performed. At this final visit, all dispensed investigational product should be reconciled, and the adverse event and concomitant medications recorded on the CRF.

As per Section 4.5, during a Public Health emergency as declared by Local or Regional authorities i.e. pandemic, epidemic or natural disaster that limits or prevents on-site study visits, alternative methods of providing continuing care may be implemented by the investigator as the situation dictates. If allowed by local Health Authority and depending on operational capabilities, phone calls, virtual contacts (e.g. tele consult) or visits by site staff/ home nursing staff to the patient´s home, can replace on-site study visits, for the duration of the disruption until it is safe for the patient to visit the site again.

The "X" in the table denotes the assessments to be recorded in the clinical database or received electronically from a vendor. The "S" in the table denotes the assessments that are only in the patient's source of documentation and do not need to be recorded in the clinical database.

No CRF will be used as a source document.

Table 8-1 Windows for Visit Assessments

Visit Name	Assessments	Window Allowed
Screening	Physical examination (complete), Performance status, vital signs, hematology, chemistry, coagulation, ECG<br><br>Serum pregnancy test<br><br>Other screening assessments	≤3 days before C1D1 do not need to be repeated at C1D1<br><br>≤ 3 days before C1D1<br><br>≤ 21 days before C1D1, with the exception of tumor assessments which must be performed ≤ 28 days before Cycle 1 Day 1.
C1D1	All visit assessments	N/A<br>(if screening laboratory assessments and physical examination were performed ≤ 3 days before first dose of study treatment, they do not need to be repeated on C1D1)
Day 1 visit of all subsequent cycles	All visit assessments	+/- 3 days
During Treatment and Efficacy follow-up periods	Ophthalmological assessments<br><br>Imaging assessments (regardless of dosing schedule or possible dose delays)	+/- 3 days<br><br>+/- 7 days
End of Treatment	All assessments	within 14 days of last dose
30 day and 60 day Safety Follow-Up Visit from last dose of study treatment	All assessments	+7 days

Table 8-2 Assessment schedule (Q2W and Q4W dosing schedule)

Period	Screening	Treatment															End of Treatment/ Study	Follow-up			
**Visit Name**		**Cycle 1**						**Cycle 2**					**Cycle 3**		**Cycle 4 & Subsequent Cycles**			**30-day safety**	**60-day safety**	**Disease Progression**	**Survival**
**Days**	**-21 to -1**	**1**	**2**	**4**	**8**	**15**	**16-18**	**1**	**2**	**4**	**8**	**15**	**1**	**15**	**1**	**15**					
Obtain informed consent	X																				
Demography	X																				
Inclusion/exclusion criteria	X																				
Relevant medical history/current medical history	X																				
Diagnosis & extent of cancer	X																				
Prior antineoplastic therapy	X																				
Prior/concomitant medications	X	**Continuous collections until 60-day safety follow-up**																			
Baseline demographics and tumor characteristics	X																				
Physical Exam	S	S				S		S				S	S		S		S				

Period	Screening	Treatment															End of Treatment/ Study	Follow-up			
**Visit Name**		**Cycle 1**						**Cycle 2**					**Cycle 3**		**Cycle 4 & Subsequent Cycles**			**30-day safety**	**60-day safety**	**Disease Progression**	**Survival**
**Days**	**-21 to -1**	**1**	**2**	**4**	**8**	**15**	**16-18**	**1**	**2**	**4**	**8**	**15**	**1**	**15**	**1**	**15**					
ECOG Performance status	X	X						X					X		X		X				
Tanner Scale (adolescents only)	X																X				
Karnofsky/Lansky performance scale (adolescents only)	X	X						X					X		X		X				
Height[1]	X																				
Weight	X	X				X[2]		X				X[2]	X	X[2]	X	X[2]	X				
Vital Signs[3]	X	X	X	X	X	X		X			X	X	X	X	X	X	X				
Hematology	X	X			X	X		X			X	X	X	X	X	X	X				
Chemistry	X	X			X	X		X			X	X	X	X	X	X	X				
Coagulation	X	Prior to taking the on-treatment biopsy															X				
Thyroid panel	X	X						X					X		X		X				
Hepatitis markers (HBV, HCV)	X	As clinically indicated																			
Serum Pregnancy Test	S	S						S					S		S		S	S	S		
Urine Pregnancy Test																		S	S		
Urinalysis (macroscopic only, if abnormal then microscopic)	X	As clinically indicated															X				

Period	Screening	Treatment															End of Treatment/ Study	Follow-up			
Visit Name		Cycle 1						Cycle 2					Cycle 3		Cycle 4 & Subsequent Cycles			30-day safety	60-day safety	Disease Progression	Survival
**Days**	**-21 to -1**	**1**	**2**	**4**	**8**	**15**	**16-18**	**1**	**2**	**4**	**8**	**15**	**1**	**15**	**1**	**15**					
Disease assessment per RECIST 1.1[4]	X	During treatment: Every 8 weeks (+/- 7 days) from C1D1 while on study until Cycle 13 day 1, then every 12 weeks (+/- 15 days) thereafter until progression of disease, death, lost to follow up or withdrawal of consent.															X if no scan within 30 days prior to EOT			X	
Electrocardiogram (ECG)	Refer to Table 8-9 for details																X				
Ophthalmological assessments	Refer to Table 8-10 for details																				
Adverse events	X	**Continuously report throughout the trial until the end of the 60-day safety follow-up**																			
Blood sample for PK (all analytes) and IG analysis		Refer to Table 8-11, Table 8-12, Table 8-13, and Table 8-14 for specific time points																			
Newly obtained tumor sample	X[8]						X[5]		X[5]								X[6]				
Blood sample for cfDNA		X			X			X					X		X[7]		X				
DYP688 dosing (Q2W and Q4W)		X				X[2]		X				X[2]	X	X[2]	X	X[2]					
Antineoplastic therapies since discontinuation																		X	X		
Survival contact																					Every 12 weeks post

Period	Screening	Treatment															End of Treatment/ Study	Follow-up			
**Visit Name**		**Cycle 1**						**Cycle 2**					**Cycle 3**		**Cycle 4 & Subsequent Cycles**			**30-day safety**	**60-day safety**	**Disease Progression**	**Survival**
**Days**	**-21 to -1**	**1**	**2**	**4**	**8**	**15**	**16-18**	**1**	**2**	**4**	**8**	**15**	**1**	**15**	**1**	**15**					
																					safety follow-up

X = assessment to be recorded in the clinical database or received electronically from a vendor
S = assessment to be recorded in the source documentation only

[1] For adolescent patients, height will be assessed prior to each cycle

[2] Q2W dosing schedule only

[3] During the infusion, vital sign monitoring should be performed every 15 min. Post infusion, patients should be monitored hourly, or more frequently if clinically indicated for approximately 4 hours, or if required by a local HA, for a minimum of 4 hours. Post-infusion observational periods can be reduced to a minimum of one (1) hour per PI discretion after completing 3 cycles. For cases where a patient intra-patient dose escalates, post-infusion observational periods can be reduced to one (1) hour per PI discretion after completing one (1) cycle at the new dose level/regimen.

[4] Imaging will be assessed locally and centrally for all patients.

[5] On treatment biopsy should be taken anytime between C1D16-18 for the Q2W dosing schedule and anytime between C2D2-4 for the Q4W dosing schedule.

[6] Optional at the time if disease progression, +14 days window for collection and prior to starting new treatment

[7] Cycle 5 only

[8] Archival tumor and a copy of a de-identified pathology report, may be permitted if it meets the inclusion criteria as outlined in Section 5.1.

If required by a local Health Authority, hospitalization up to 48 hours is possible at Cycle 1 Day 1 in the dose escalation part of the study.

Table 8-3 Assessment schedule (QW dosing schedule)

Period	Screening	Treatment																End of Treatment/ Study	Follow-up			
Visit Name		Cycle 1							Cycle 2					Cycle 3 & Subsequent Cycles					30-day safety	60-day safety	Disease Progression	Survival
**Days**	**-21 to -1**	**1**	**2**	**4**	**8**	**15**	**16-18**	**22**	**1**	**2**	**8**	**15**	**22**	**1**	**8**	**15**	**22**					
Obtain informed consent	X																					
Demography	X																					
Inclusion/exclusion criteria	X																					
Relevant medical history/current medical history	X																					
Diagnosis & extent of cancer	X																					
Prior antineoplastic therapy	X																					
Prior/concomitant medications	X	**Continuous collections until 60-day safety follow-up**																				
Baseline demographics and tumor characteristics	X																					
Physical Exam	S	S			S	S		S	S		S	S	S	S				S				
ECOG Performance status	X	X							X					X				X				
Tanner Scale (adolescents only)	X																	X				

Period	Screening	Treatment																End of Treatment/ Study	Follow-up			
**Visit Name**		**Cycle 1**							**Cycle 2**					**Cycle 3 & Subsequent Cycles**					**30-day safety**	**60-day safety**	**Disease Progression**	**Survival**
**Days**	**-21 to -1**	**1**	**2**	**4**	**8**	**15**	**16-18**	**22**	**1**	**2**	**8**	**15**	**22**	**1**	**8**	**15**	**22**					
Karnofsky/Lansky performance scale (adolescents only)	X	X							X					X				X				
Height[1]	X																					
Weight	X	X			X	X		X	X		X	X	X	X	X	X	X	X				
Vital Signs[2]	X	X	X	X	X	X		X	X		X	X	X	X	X	X		X				
Hematology	X	X			X	X		X	X		X	X	X	X		X		X				
Chemistry	X	X			X	X		X	X		X	X	X	X		X		X				
Coagulation	X	Prior to taking the on-treatment biopsy																X				
Thyroid panel	X	X							X					X				X				
Hepatitis markers (HBV,HCV)	X	As clinically indicated																				
Serum Pregnancy Test	S	S							S					S				S	S	S		
Urine Pregnancy Test																			S	S		
Urinalysis (macroscopic only, if abnormal then microscopic)	X	As clinically indicated																X				
Disease assessment per RECIST 1.1[3]	X	During treatment: Every 8 weeks (+/- 7 days) from C1D1 until Cycle 13 day 1 while on study, then every 12 weeks (+/- 15 days) thereafter until progression of disease, death, lost to follow up or withdrawal of consent.																X if no scan within 30 days prior to EOT			X	

Period	Screening	Treatment																End of Treatment/ Study	Follow-up			
**Visit Name**		**Cycle 1**							**Cycle 2**					**Cycle 3 & Subsequent Cycles**					**30-day safety**	**60-day safety**	**Disease Progression**	**Survival**
**Days**	**-21 to -1**	**1**	**2**	**4**	**8**	**15**	**16-18**	**22**	**1**	**2**	**8**	**15**	**22**	**1**	**8**	**15**	**22**					
Electrocardiogram (ECG)	Refer to Table 8-9 for details																	X				
Ophthalmological assessments	Refer to Table 8-10 for details																					
Adverse events	X	**Continuously report throughout the trial and until the end of the 60-day safety follow-up period**																				
Blood sample for PK (all analytes) and IG analysis			Refer to Table 8-15 and Table 8-16 for specific time points																			
Newly obtained tumor sample	X[7]						X[4]											X[5]				
Blood sample for cfDNA		X			X				X					X[6]				X				
DYP688 dosing (QW)		X			X	X		X	X		X	X	X	X	X	X	X					
Antineoplastic therapies since discontinuation																			X	X		
Survival contact																						Every 12 weeks post safety follow-up
	X = assessment to be recorded in the clinical database or received electronically from a vendor<br>S = assessment to be recorded in the source documentation only																					

Period	Screening	Treatment																End of Treatment/ Study	Follow-up			
Visit Name		Cycle 1							Cycle 2					Cycle 3 & Subsequent Cycles					30-day safety	60-day safety	Disease Progression	Survival
Days	-21 to -1	1	2	4	8	15	16-18	22	1	2	8	15	22	1	8	15	22					

[1] For adolescent patients, height will be assessed prior to each cycle

[2] During the infusion, vital sign monitoring should be performed every 15 min. Post infusion, patients should be monitored hourly, or more frequently if clinically indicated for approximately 4 hours, or if required by a local HA, for a minimum of 4 hours. Post-infusion observational periods can be reduced to a minimum of one (1) hour per PI discretion after completing 3 cycles. For cases where a patient intra-patient dose escalates, post-infusion observational periods can be reduced to one (1) hour per PI discretion after completing one (1) cycle at the new dose level/regimen.

[3] Imaging will be assessed locally and centrally for all patients.

[4] On treatment biopsy should be taken anytime between C1D16-18

[5] Optional at the time if disease progression, + 14 days window for collection and prior to starting new treatment

[6] Cycle 3 and 5 only

[7] Archival tumor and a copy of a de-identified pathology report, may be permitted if it meets the inclusion criteria as outlined in Section 5.1.

If required by a local Health Authority, hospitalization up to 48 hours is possible at Cycle 1 Day 1 in the dose escalation part of the study.

8.1 Screening

The study IRB/IEC approved informed consent form must be signed and dated before any screening procedures are performed, except for laboratory and radiological evaluations that were performed as part of the patient's clinical standard of care within the acceptable screening window.

Patients will be evaluated against study inclusion and exclusion criteria and safety assessments (Table 8-2 and Table 8-3). Screening assessments must be repeated if performed outside of the specified screening window. Patients must meet all inclusion and none of the exclusion criteria at screening in order to be eligible for the study.

Laboratory test result(s) or symptoms that do not satisfy the eligibility criteria may be repeated or treated during the screening visit window. In the event that the repeated laboratory test(s) cannot be performed within the screening window or the patient continues to not meet eligibility criteria, the patient is considered a screening failure.

Re-screening of a subject who has failed screening may be allowed. In such case a new ICF must be signed and a new patient ID will be assigned. All required screening assessments must be repeated if they do not meet the allowed time window for screening when the subject is re-screened for participation in the study. An individual patient can only be re-screened once for the study.

8.1.1 Information to be collected on screening failures

Patients who are found ineligible after signing the main ICF will be considered as screening failures and data will be handled in accordance with screening failures.

In this situation, the investigator must provide a minimal set of screen failure information. Minimal information includes reason for screen failure, demography, medical history, screen failure details, mutational status (local data), inclusion and exclusion criteria, and any serious adverse event (SAE). Data, tumor and blood samples collected from patients prior to screen failure may still be analyzed. The reason for screening failure will be entered on the disposition page. No other data will be entered into the clinical database for patients who are screening failures, unless the patient experienced a Serious Adverse Event during the Screening period (see Section 10 for SAE reporting details). For patients who fail screening, SAEs will be reported in the database up to the screening failure date.

Tumor and blood samples provided by patients who fail screening will be retained by Novartis and may be used for companion diagnostic development.

Patients who sign an informed consent and are considered eligible but fail to be started on treatment for any reason will be considered an early terminator. The reason for early termination should be captured on the appropriate disposition eCRF.

8.2 Patient demographics/other baseline characteristics

Demographic and baseline characteristic data are to be collected on all patients. This information will include general patient demographics: full date (only if required and permitted) or year of birth, sex, race/predominant ethnicity (if permitted); relevant medical history/current

medical conditions (until date of signature of informed consent) will be recorded in the CRF; concomitant medications; diagnosis and extent of cancer; baseline tumor molecular characteristics; HLA status (if available); and details of prior antineoplastic treatments.

Investigators will have the discretion to record abnormal test findings on the appropriate eCRF whenever in their judgment, the test abnormality occurred prior to the informed consent signature.

Country-specific regulations should be considered for the collection of demographic and baseline characteristics in alignment with eCRF.

Patient race and ethnicity are collected and analyzed to assess the diversity of the study population as required by certain Health Authorities and may be used to identify variations in safety or efficacy due to these factors.

8.3 Efficacy

8.3.1 Tumor Imaging

For all patients enrolled on the study tumor response will be assessed locally and centrally according to the Novartis Guideline for Response (Section 16.1) based on RECIST 1.1. The imaging assessment collection plan is presented in Table 8-2 and Table 8-3. The central review of the scans will be carried out in a blinded fashion. Further details regarding the central review process will be described in the Blinded Independent Review Committee (BIRC) charter.

Imaging data will be centrally collected and checked for quality by an imaging Contract Research Organization designated by Novartis. The results of the central evaluations will be used for primary analysis purposes. The local investigator’s assessment will be used for making treatment decisions and for the secondary endpoint analysis.

Table 8-4 Imaging Assessment Collection Plan

Procedure	Screening	During Treatment/Follow-up
Chest, abdomen and pelvis CT or MRI (with intravenous contrast enhancement)	Mandated	Mandated, every 8 weeks (+/- 7 days) until Cycle 13 day 1 while on study and every 12 weeks (+/- 15 days) thereafter
Brain CT or MRI	If clinically indicated	If lesions were documented at baseline, follow same schedule as CT/MRI of chest, abdomen, and pelvis
Whole body bone scan	If clinically indicated	If clinically indicated
Localized bone CT, MRI or x-ray	For any lesions identified on the whole body bone scan that are not visible on the chest, abdomen and pelvis CT or MRI	If lesions were documented at baseline, follow same schedule as CT/MRI of chest, abdomen, and pelvis
Color photography (with scale/ruler)	For any skin lesions present	If lesions were documented at baseline, follow same schedule as CT/MRI of chest, abdomen, and pelvis

Procedure	Screening	During Treatment/Follow-up
CT or MRI of other metastatic sites (e.g., neck)	If clinically indicated	If lesions were documented at baseline, follow same schedule as CT/MRI of chest, abdomen, and pelvis

8.3.1.1 Baseline imaging assessments

Imaging assessments should be performed at screening/baseline within 28 days of start of treatment (Day -28 to Day -1 prior to Cycle 1 Day 1) per Table 8-2. Any imaging assessments already completed during the regular work-up of the patient within 28 days prior to start of treatment including before signing the main study ICF, can be considered as the baseline images for this study. Any imaging assessments obtained after the first dose of treatment cannot be considered baseline images.

Any potentially measurable lesion that has been previously treated with radiotherapy should be considered as a non-measurable lesion. However, if a lesion previously treated with radiotherapy has clearly progressed since radiotherapy, it can be considered as a measurable lesion.

If a patient is known to have a contraindication to CT i.v. contrast media or develops a contraindication during the trial, a non-contrast CT of the chest (MRI is not recommended due to respiratory artifacts, however if CT is not feasible per local regulations, MRI can be performed instead) plus a contrast-enhanced MRI (if possible) of the abdomen and pelvis should be performed.

8.3.1.2 Post-baseline imaging assessments

Imaging assessments as described in Table 8-2 and Table 8-3 should be performed at the time points specified using the same imaging modality used at baseline, irrespective of study treatment interruption or actual dosing (see Table 8-2 and Table 8-3). Imaging assessments for response evaluation will be performed every 8 weeks (+/- 7 days) until disease progression, death, lost to follow-up or withdrawal of consent, and at EOT if no scan was performed within 30 days prior to EOT. For all patients in dose escalation, the imaging assessments should continue until disease progression by local review by the investigator. For patients in the Phase II part of the study, the imaging assessments should continue until disease progression by central review even if progression is assessed earlier by the local investigator.

For patients continuing treatment beyond disease progression (if receiving clinical benefit) the imaging assessments will continue till the discontinuation of treatment.

Note: Imaging assessments should be scheduled using the first dose of study treatment and should be respected regardless of whether treatment with study treatment is temporarily withheld or unscheduled assessments are performed.

Additional imaging assessments may be performed at any time during the study at the investigator's discretion to support the efficacy evaluations for a patient, as necessary.

Clinical suspicion of disease progression at any time requires a physical examination and imaging assessments to be performed promptly rather than waiting for the next scheduled imaging assessment.

Each lesion that is measured at baseline must be measured by the same method (either same imaging method or by photography, including a metric ruler) and when possible, by the same local radiologist/physician throughout the study so that the comparison is consistent. If an off-schedule imaging assessment is performed because progression is suspected, subsequent imaging assessments should be performed in accordance with the original imaging schedule.

Combined PET/CT may be used only if the CT is of similar diagnostic quality as a CT performed without PET, including the utilization of i.v. contrast media. At the discretion of the Investigators, FDG-PET scans may be performed to document disease progression per RECIST 1.1 (Section 16.1).

All complete responses (CRs) and partial responses (PRs) must be confirmed by a second assessment. If the second assessment is performed outside the normal window of scans, it should not be performed earlier than 4 weeks after the scan where the criteria for response were first met.

8.3.1.3 Post-Treatment efficacy follow-up

For patients who discontinue treatment for reasons other than documented disease progression, death, lost to follow-up, or withdrawal of consent, tumor assessments should continue to be collected according to Table 8-2 and Table 8-3 until documented disease progression, death, lost to follow-up, or withdrawal of consent occurs.

8.3.2 Overall survival

All patients enrolled will enter the survival follow-up period after completion of the safety follow-up. Survival status will be collected every 12 weeks (+/- 14 days) for up to 5 years regardless of treatment discontinuation reason (except if consent is withdrawn or patient is lost to follow-up) until death, lost to follow-up, or withdrawal of consent for survival follow-up. Survival information can be obtained via phone, and information will be documented in the source documents and relevant eCRFs.

Additional survival assessments may be performed outside the 12 weeks follow-up schedule if a survival update is required for an interim assessment to meet safety or regulatory needs.

8.3.3 Appropriateness of efficacy assessments

RECIST 1.1 is a standard approach to assessing treatment response in patients with solid tumors. Additional information can be found in Section 16.1.

8.4 Safety/Tolerability

Safety assessments are specified below with the assessment schedule detailing when each assessment is to be performed.

For details on AE collection and reporting, refer to Section 10.

Table 8-5 Physical assessments

Assessment	Specification
Physical examination	A complete physical examination will be performed at screening and Cycle 1 Day 1 and include the examination of the following: general appearance, skin, neck (including thyroid), eyes, ears, nose, throat, lungs, heart, abdomen, back, lymph nodes, extremities, and neurological examination<br>After Cycle 1 Day 1, a short physical examination will be performed (including the examination of general appearance).<br>Clinically relevant findings that were present prior to signing informed consent will be considered as part of the patient's medical history.<br>Significant new findings that begin or worsen after informed consent which meet the definition of an adverse event must be recorded as an adverse event.
Vital signs	Vital signs include blood pressure (supine position preferred when ECG is collected), pulse measurement, and body temperature.<br>During the infusion, monitor vital signs every 15 min. Post infusion, patients should be monitored hourly, or more frequently, if clinically indicated, for approximately 4 hours, or if required by a local HA, for a minimum of 4 hours. Post-infusion observational periods can be reduced to a minimum of one (1) hour per PI discretion after the patient has completed at least 3 cycles. For cases where a patient intra-patient dose escalates, post-infusion observational periods can be reduced to one (1) hour per PI discretion after completing at least one (1) cycle at a new dose level.
Height and weight	Height and body weight (in indoor clothing, but without shoes) will be measured at screening and at subsequent time points as specified in Table 8-2 and Table 8-3.

Performance status:

ECOG Performance status scale will be used as described in Table 8-6.

Table 8-6 ECOG Performance Status

Grade	ECOG status
0	Fully active, able to carry on all pre-disease performance without restriction
1	Restricted in physically strenuous activity but ambulatory and able to carry out work of a light or sedentary nature e.g., light housework, office work
2	Ambulatory and capable of all self-care but unable to carry out any work activities. Up and about more than 50% of waking hours
3	Capable of only limited self-care, confined to bed or chair more than 50% of waking hours
4	Completely disabled. Cannot carry on any self-care. Totally confined to bed or chair
5	Dead

Table 8-7 Karnofsky/Lansky Performance Status (Adolescent Population only)

**Karnofsky Scale (recipient age ≥ 16 years)**		**Lansky Scale (recipient age <16 years)**	
**Able to carry on normal activity; no special care is needed**		**Able to carry on normal activity; no special care is needed**	
100	Normal, no complaints, no evidence of disease	100	Fully active
90	Able to carry on normal activity	90	Minor restriction in physically strenuous play
80	Normal activity with effort	80	Restricted in strenuous play, tires more easily, otherwise active
**Unable to work, able to live at home cares for most personal needs, a varying amount of assistance is needed**		**Mild to moderate restriction**	
70	Cares for self, unable to carry on normal activity or to do active work	70	Both greater restrictions of, and less time spent in active play
60	Requires occasional assistance but is able to care for most needs	60	Ambulatory up to 50% of time, limited active play with assistance/supervision
50	Requires considerable assistance and frequent medical care	50	Considerable assistance required for any active play, fully able to engage in quiet play
**Unable to care for self, requires equivalent of institutional or hospital care, disease may be progressing rapidly**		**Moderate to severe restriction**	
40	Disabled, requires special care and assistance	40	Able to initiate quite activities
30	Severely disabled, hospitalization indicated, although death not imminent	30	Needs considerable assistance for quiet activity
20	Very sick, hospitalization necessary	20	Limited to very passive activity initiated by others (e.g., TV)
10	Moribund, fatal process progressing rapidly	10	Completed disabled, not even passive play

8.4.1 Laboratory evaluations

All laboratory parameters assessed for safety purposes will be evaluated locally. Refer to Table 8-8 for a summary of the parameters to be evaluated according to the visit evaluation scheduled (Table 8-2 and Table 8-3). On days of dosing, samples for these parameters should be collected prior to the administration of study drugs.

More frequent evaluations may be performed at the investigator's discretion if medically indicated; results should be recorded as unscheduled laboratory assessments.

Novartis will be provided with a copy of the laboratory certification and tabulation of the normal ranges for each parameter required. In addition, if at any time a patient has laboratory parameters obtained from a different outside laboratory, Novartis must be provided with a copy of the certification and a tabulation of the normal ranges for that laboratory. Urinalysis will be performed locally.

As per Section 4.5, during a Public Health emergency as declared by Local or Regional authorities, e.g., pandemic, epidemic or natural disaster, that limits or prevents on-site study visits, if patients cannot visit the site for protocol specified safety lab assessments, an alternative lab (local) collection site may be used.

Table 8-8 Laboratory assessments

Test Category	Test Name
Hematology	Hemoglobin, Red blood cells, Platelets, White blood cells, Differential (Basophils, Eosinophils, Lymphocytes, Monocytes, Neutrophils - absolute value preferred)
Chemistry	Albumin, Alkaline phosphatase (ALP), ALT (SGPT), AST (SGOT), Corrected Calcium, Creatinine, Gamma-glutamyl-transferase (GGT), Blood Urea Nitrogen (BUN) or Urea,<br>Amylase, Lipase, Lactate dehydrogenase (LDH), Glucose (non-fasting), Sodium, Potassium, Magnesium, Phosphorus, Total Bilirubin.<br>If Total Bilirubin is ≥ 1.5 ULN then also provide direct or indirect Bilirubin.
Urinalysis	Macroscopic Panel (Dipstick) (Color, Bilirubin, Blood, Glucose, Ketones, Leukocytes esterase, Nitrite, pH, Protein, Specific Gravity, Urobilinogen)<br>Microscopic Panel (Red Blood Cells, White Blood Cells, Casts, Crystals, Bacteria, Epithelial cells)[1]
Coagulation	Activated partial thromboplastin time (aPTT), International normalized ratio (INR)
Hormone	T4 [free], TSH,
Hepatitis markers	Hepatitis B Virus DNA, Hepatitis B Virus Surface Antigen, Hepatitis B Virus Surface Antibody, Hepatitis B Virus Core Antibody, Hepatitis C Virus RNA
Pregnancy Test (For WOCBP only)	Serum / Urine pregnancy test (see Section 8.4.3 for additional information)

[1] Microscopic Panel only to be performed if macroscopic is abnormal

8.4.2 Electrocardiogram (ECG)

A standard 12 lead ECG will be performed for all patients in dose escalation and the Phase 2 part of the study as per the assessment schedule in Table 8-9. Blood samples scheduled at the same time point should be taken after the ECGs are completed. All ECGs must be performed in triplicate.

All ECGs will be independently reviewed by a central laboratory. Instructions for the collection and transmission of ECGs to the central ECG laboratory will be provided in the ECG Manual.

Clinically significant abnormalities present at screening should be reported on the Medical History CRF page. New or worsened clinically significant findings occurring after informed consent must be recorded on the Adverse Events CRF page. All eligibility and patient management decisions should be made based on the local reading of the ECG.

For all patients treated in the study experiencing a grade 3 QTc interval prolongation, potassium and magnesium levels should be checked and any electrolyte abnormalities corrected with supplements appropriately; unscheduled ECG assessments should be performed as necessary until resolution to baseline or Grade 1.

Additional, unscheduled, safety ECGs may be repeated at the discretion of the investigator at any time during the study as clinically indicated. Unscheduled ECGs with clinically significant findings should be collected in triplicate. Local cardiologist ECG assessment may also be performed at any time during the study at the discretion of the investigator.

Table 8-9 ECG collection plan for all patients enrolled in Dose escalation and Phase II

Cycle	Day	Time	ECG Type	Number of ECGs
Screening		Anytime	12 Lead	3
1	1	Pre-dose<br>Post-dose 1 h (within 15 min prior to PK)<br>Post-dose 4 h (within 15 min prior to PK)*	12 Lead	3
1	2	Within 15 min prior to PK	12 Lead	3
1	4	Within 15 min prior to PK	12 Lead	3
3	1	Pre-dose<br>Post-dose 1 h (within 15 min prior to PK)<br>Post-dose 4 h (within 15 min prior to PK)*	12 Lead	3
3	2	Within 15 min prior to PK	12 Lead	3
3	4	Within 15 min prior to PK	12 Lead	3
EOT		Anytime	12 Lead	3
Unscheduled ECG		Anytime if clinically indicated	12 Lead	3
*To be performed in Phase I only				

8.4.3 Pregnancy and assessments of fertility

All women of childbearing potential will have pregnancy testing per the assessment schedule in Table 8-2 and Table 8-3. Women of childbearing potential are defined as women physiologically capable of becoming pregnant from menarche until becoming post-menopausal unless they are using highly effective methods of contraception (failure rate < 1% per year) while taking study treatment and for 60 days after stopping study treatment or they have had surgical bilateral oophorectomy (with or without hysterectomy), total hysterectomy or bilateral salpingectomy. In the case of oophorectomy alone, the reproductive status of the woman needs to have been confirmed by follow-up hormone level assessment. Women are considered post-menopausal if they have had 12 months of natural (spontaneous) amenorrhea with an appropriate clinical profile (e.g., hormonal profile confirming menopause and/or age-appropriate history of vasomotor symptoms).

Medical documentation of bilateral oophorectomy, hysterectomy or bilateral salpingectomy must be retained as source documents.

In absence of the medical documentation of these procedures, or if the post-menopausal status is not clear, the investigator should use medical judgment to appropriately evaluate the fertility state of the woman and document it in the source document.

All pre-menopausal women who are not surgically sterile will have pregnancy testing, as described below. Additional pregnancy testing might be performed if requested by local requirements.

At screening, a serum pregnancy test must be performed within 72 hours before the first dose. During the study, a serum pregnancy test must be performed on day 1 of every cycle starting with cycle 1 and at end of treatment.

A serum or a urine pregnancy testing will be performed at the 30-day and 60-day follow-up visits after discontinuing study treatment. If the subject is not coming to the clinical site for the safety follow-up visit, it can be performed at home or at a local doctor's office, and the results will be communicated to the site staff. The follow-up pregnancy test will be documented only in source. In case of a positive urine pregnancy test, additional tests must be performed to confirm pregnancy and if confirmed the reporting requirements as described in Section 10.1.4 must be followed.

If a patient becomes pregnant, study treatment must be stopped immediately. If a pregnancy test (urine or serum) is positive, but the subject is thought not to be pregnant, study drug should be stopped until it is determined that the test was falsely positive, and pregnancy is excluded.

If patients cannot visit the site to have serum pregnancy tests, urine pregnancy test kits may be used. Relevant patients can perform the urine pregnancy test at home and report the result to the site. A communication process should be established with the patient so that the Site is informed and can verify the pregnancy test results (e.g. following Country specific measures).

8.4.4 Ophthalmological assessments

Complete ophthalmologic exams will be performed by an ophthalmologist or qualified specialist in all patients at screening and on-treatment, end of treatment, and if there are any new ocular symptoms such as blurred vision, in a given patient. Ocular symptom assessment will be collected as indicated in Table 8-10.

If abnormal ocular findings were observed at EOT compared with baseline (screening) assessments, a complete ophthalmologic exam should be performed during safety follow-up. For slit lamp examination, it is recommended to use the same technique at screening and post screening.

Clinically significant abnormalities present at screening should be reported on the Medical History CRF page. New or worsened clinically significant findings occurring after informed consent must be recorded on the Adverse Events CRF page.

Table 8-10 Ophthalmologic monitoring

Assessment	Screening	C1D4	C1D15	C2D1	C3D1	C5D1[3]	End of Treatment
Prior ophthalmologic history	X						
Complete ophthalmologic exam including[2]: Visual acuity<br>Pupillary reflexes Slit lamp examination Intraocular pressure measurement Dilated Fundoscopy	X[1]	X	X	X	X	X	X

[1] Within 14 days prior to Cycle 1, Day 1
[2] Complete ophthalmologic exam should also be performed as clinically indicated
[3] As per investigator judgment or for patients who have experienced ocular AEs during prior cycles.

8.4.5 Appropriateness of safety measurements

The safety assessments selected are standard for this indication/patient population.

8.5 Additional assessments

8.5.1 Pharmacokinetics and Immunogenicity

Serial blood samples will be collected from all patients in dose escalation and the Phase II part of the study in order to characterize the single dose and steady-state PK of DYP688 in serum, plasma, and blood. In addition, remaining plasma/serum/blood samples might be used for exploratory PK and/or biomarker analysis. This could include using remaining serum, plasma, and blood samples for protein binding analysis, metabolite profiling, exploratory biomarker analyses, protein binding, or other assays if there is sufficient sample remaining. Collection of PK and/or IG samples may be stopped upon decision of the Sponsor, if/when sufficient data are collected, after the primary CSR cut-off date is reached or upon early termination of the study.

8.5.1.1 Pharmacokinetic and Immunogenicity blood collection and handling

PK and IG blood samples will be taken by either direct venipuncture or via an indwelling cannula inserted in a forearm vein at the visits defined in the pharmacokinetic log (Table 8-11, Table 8-12, Table 8-13, Table 8-14, Table 8-15, and Table 8-16). Blood samples are to be collected from the arm opposite from infusion site. Refer to the central laboratory manual for detailed instructions regarding sample collection, numbering, processing and shipment.

Complete dosing and blood sampling information, including the date/time of actual blood draw and date/time of the last study drug dose prior to the sampling, should be obtained on all sampling days and recorded on the PK/IG eCRF.

An unscheduled PK/IG blood sample will be collected, 1) if a patient experiences an AE suspected to be related to study treatment that results in an unscheduled visit or fits the criteria of an SAE, 2) whenever a post-baseline tumor sample is collected during the treatment.

The date and time of the unscheduled blood sample collection, and the date, time and dose of the investigational drug(s) taken prior to the unscheduled blood sample collection should be entered in the appropriate eCRF

On days and time points where blood PD samples are to be drawn, the PK and IG samples should be drawn first. On days and time points where time-matched PK and ECG are collected, take ECG prior to PK and IG blood draws.

In order to allow for flexibility to explore other dosing regimens, the blood collection logs are given for the following dosing regimen: Q2W (initial frequency of dosing every two weeks, Table 8-11 and Table 8-12), Q4W (dosing every four weeks, Table 8-13 and Table 8-14) and QW (weekly dosing, Table 8-15 and Table 8-16).

Table 8-11 Pharmacokinetic and Immunogenicity blood collection log for DYP688 single agent treatment in dose escalation (Q2W dosing schedule)[1,2]

Cycle	Day	Schedule time (hours)	Analytes
1	1	Pre-dose (0 h)	DYP688, SDZ475, [redacted], [redacted], IG
1	1	1 h post dose (± 5 min)	DYP688, SDZ475, [redacted], [redacted]
1	1	4 h post dose (± 1 h)	DYP688, SDZ475, [redacted], [redacted]
1	2	24 h post dose (± 2h)	DYP688, SDZ475, [redacted], [redacted]
1	4	72 h post dose (± 4 h)	DYP688, SDZ475, [redacted], [redacted]
1	8	168 h post dose (± 6 h)	DYP688, SDZ475, [redacted], [redacted]
1	15	Pre-dose (0 h)	DYP688, SDZ475, [redacted], [redacted], IG
1	15	1 h post dose (± 5 min)	DYP688, SDZ475, [redacted], [redacted]
1	16-18	At time of biopsy	DYP688, SDZ475, [redacted], [redacted]
2	1	Pre-dose (0 h)	DYP688, SDZ475, [redacted], [redacted], IG
2	1	1 h post dose (± 5 min)	DYP688, SDZ475, [redacted], [redacted]
2	15	Pre-dose (0 h)	DYP688, SDZ475, [redacted], [redacted], IG
2	15	1 h post dose (± 5 min)	DYP688, SDZ475, [redacted], [redacted]
3	1	Pre-dose (0 h)	DYP688, SDZ475, [redacted], [redacted], IG

Cycle	Day	Schedule time (hours)	Analytes
3	1	1 h post dose (± 5 min)	DYP688, SDZ475, [redacted], [redacted]
3	1	4 h post dose (± 1 h)	DYP688, SDZ475, [redacted], [redacted]
3	2	24 h post dose (± 2 h)	DYP688, SDZ475, [redacted], [redacted]
3	4	72 h post dose (± 4 h)	DYP688, SDZ475, [redacted], [redacted]
3	8	168 h post dose (± 6 h)	DYP688, SDZ475, [redacted], [redacted]
3	15	Pre-dose (0 h)	DYP688, SDZ475, [redacted], [redacted], IG
3	15	1 h post dose (± 5 min)	DYP688, SDZ475, [redacted], [redacted]
4	1	Pre-dose (0 h)	DYP688, SDZ475, [redacted], [redacted], IG
5	1	Pre-dose (0 h)	DYP688, SDZ475, [redacted], [redacted], IG
6	1	Pre-dose (0 h)	DYP688, SDZ475, [redacted], [redacted], IG
EOT			DYP688, SDZ475, [redacted], [redacted], IG
Unscheduled			DYP688, SDZ475, [redacted], [redacted], IG

Table 8-12 Pharmacokinetic and Immunogenicity blood collection log for DYP688 single agent treatment in Phase II (Q2W dosing schedule) [1,2]

Cycle	Day	Schedule time (hours)	Analytes
1	1	Pre-dose (0 h)	DYP688, SDZ475, [redacted], [redacted], IG
1	1	1 h post dose (± 5 min)	DYP688, SDZ475, [redacted], [redacted]
1	2	24 h post dose (± 2 h)	DYP688, SDZ475, [redacted], [redacted]
1	4	72 h post dose (± 4 h)	DYP688, SDZ475, [redacted], [redacted]
1	15	Pre-dose (0 h)	DYP688, SDZ475, [redacted], [redacted], IG
1	15	1 h post dose (± 5 min)	DYP688, SDZ475, [redacted], [redacted]
1	16-18	At time of biopsy	DYP688, SDZ475, [redacted], [redacted]
2	1	Pre-dose (0 h)	DYP688, SDZ475, [redacted], [redacted], IG

Cycle	Day	Schedule time (hours)	Analytes
2	1	1 h post dose (± 5 min)	DYP688, SDZ475, [redacted], [redacted]
2	15	Pre-dose (0 h)	DYP688, SDZ475, [redacted], [redacted], IG
2	15	1 h post dose (± 5 min)	DYP688, SDZ475, [redacted], [redacted]
3	1	Pre-dose (0 h)	DYP688, SDZ475, [redacted], [redacted], IG
3	1	1 h post dose (± 5 min)	DYP688, SDZ475, [redacted], [redacted]
3	2	24 h post dose (± 2 h)	DYP688, SDZ475, [redacted], [redacted]
3	4	72 h post dose (± 4 h)	DYP688, SDZ475, [redacted], [redacted]
3	15	Pre-dose (0 h)	DYP688, SDZ475, [redacted], [redacted], IG
3	15	1 h post dose (± 5 min)	DYP688, SDZ475, [redacted], [redacted]
4	1	Pre-dose (0 h)	DYP688, SDZ475, [redacted], [redacted], IG
5	1	Pre-dose (0 h)	DYP688, SDZ475, [redacted], [redacted], IG
6	1	Pre-dose (0 h)	DYP688, SDZ475, [redacted], [redacted], IG
EOT			DYP688, SDZ475, [redacted], [redacted], IG
Unscheduled			DYP688, SDZ475, [redacted], [redacted], IG

Table 8-13 Pharmacokinetic and Immunogenicity blood collection log for DYP688 single agent treatment in dose escalation (Q4W dosing schedule) [1,2]

Cycle	Day	Schedule time (hours)	Analytes
1	1	Pre-dose (0 h)	DYP688, SDZ475, [redacted], [redacted], IG
1	1	1 h post dose (± 5 min)	DYP688, SDZ475, [redacted], [redacted]
1	1	4 h post dose (± 1 h)	DYP688, SDZ475, [redacted], [redacted]
1	2	24 h post dose (± 2 h)	DYP688, SDZ475, [redacted], [redacted]
1	4	72 h post dose (± 4 h)	DYP688, SDZ475, [redacted], [redacted]
1	8	168 h post dose (± 6 h)	DYP688, SDZ475, [redacted], [redacted]

Cycle	Day	Schedule time (hours)	Analytes
1	15	336 h post dose (± 8 h)	DYP688, SDZ475, [redacted], [redacted]
2	1	Pre-dose (0 h)	DYP688, SDZ475, [redacted], [redacted], IG
2	1	1 h post dose (± 5 min)	DYP688, SDZ475, [redacted], [redacted]
2	2-4	At time of biopsy	DYP688, SDZ475, [redacted], [redacted]
3	1	Pre-dose (0 h)	DYP688, SDZ475, [redacted], [redacted], IG
3	1	1 h post dose (± 5 min)	DYP688, SDZ475, [redacted], [redacted]
3	1	4 h post dose (± 1 h)	DYP688, SDZ475, [redacted], [redacted]
3	2	24 h post dose (± 2h)	DYP688, SDZ475, [redacted], [redacted]
3	4	72 h post dose (± 4 h)	DYP688, SDZ475, [redacted], [redacted]
3	8	168 h post dose (± 6 h)	DYP688, SDZ475, [redacted], [redacted]
3	15	336 h post dose (± 8 h)	DYP688, SDZ475, [redacted], [redacted]
4	1	Pre-dose (0 h)	DYP688, SDZ475, [redacted], [redacted], IG
4	1	1 h post dose (± 5 min)	DYP688, SDZ475, [redacted], [redacted]
5	1	Pre-dose (0 h)	DYP688, SDZ475, [redacted], [redacted], IG
6	1	Pre-dose (0 h)	DYP688, SDZ475, [redacted], [redacted], IG
EOT			DYP688, SDZ475, [redacted], [redacted], IG
Unscheduled			DYP688, SDZ475, [redacted], [redacted], IG

Table 8-14 Pharmacokinetic and Immunogenicity blood collection log for DYP688 single agent treatment in Phase II (Q4W dosing schedule) [1,2]

Cycle	Day	Schedule time (hours)	Analytes
1	1	Pre-dose (0 h)	DYP688, SDZ475, [redacted], [redacted], IG
1	1	1 h post dose (± 5 min)	DYP688, SDZ475, [redacted], [redacted]
1	2	24 h post dose (± 2 h)	DYP688, SDZ475, [redacted], [redacted]
1	4	72 h post dose (± 4 h)	DYP688, SDZ475, [redacted], [redacted]
1	8	168 h post dose (± 6 h)	DYP688, SDZ475, [redacted], [redacted]
2	1	Pre-dose (0 h)	DYP688, SDZ475, [redacted], [redacted], IG
2	1	1 h post dose (± 5 min)	DYP688, SDZ475, [redacted], [redacted]
2	2-4	At time of biopsy	DYP688, SDZ475, [redacted], [redacted]
3	1	Pre-dose (0 h)	DYP688, SDZ475, [redacted], [redacted], IG
3	1	1 h post dose (± 5 min)	DYP688, SDZ475, [redacted], [redacted]
3	2	24 h post dose (± 2 h)	DYP688, SDZ475, [redacted], [redacted]
3	4	72 h post dose (± 4 h)	DYP688, SDZ475, [redacted], [redacted]
3	8	168 h post dose (± 6 h)	DYP688, SDZ475, [redacted], [redacted]
4	1	Pre-dose (0 h)	DYP688, SDZ475, [redacted], [redacted], IG
4	1	1 h post dose (± 5 min)	DYP688, SDZ475, [redacted], [redacted]
5	1	Pre-dose (0 h)	DYP688, SDZ475, [redacted], [redacted], IG
6	1	Pre-dose (0 h)	DYP688, SDZ475, [redacted], [redacted], IG
EOT			DYP688, SDZ475, [redacted], [redacted], IG
Unscheduled			DYP688, SDZ475, [redacted], [redacted], IG

Table 8-15 Pharmacokinetic and Immunogenicity blood collection log for DYP688 single agent treatment in dose escalation (QW dosing schedule) [1,2]

Cycle	Day	Schedule time (hours)	Analytes
1	1	Pre-dose (0 h)	DYP688, SDZ475, [redacted], [redacted], IG
1	1	1 h post dose (± 5 min)	DYP688, SDZ475, [redacted], [redacted]
1	1	4 h post dose (± 1 h)	DYP688, SDZ475, [redacted], [redacted]
1	2	24 h post dose (± 2 h)	DYP688, SDZ475, [redacted], [redacted]
1	4	72 h post dose (± 4 h)	DYP688, SDZ475, [redacted], [redacted]
1	8	Pre-dose (0 h)	DYP688, SDZ475, [redacted], [redacted]
1	8	1 h post dose (± 5 min)	DYP688, SDZ475, [redacted], [redacted]
1	15	Pre-dose (0 h)	DYP688, SDZ475, [redacted], [redacted], IG
1	15	1 h post dose (± 5 min)	DYP688, SDZ475, [redacted], [redacted]
1	16-18	At time of biopsy	DYP688, SDZ475, [redacted], [redacted]
1	22	Pre-dose (0 h)	DYP688, SDZ475, [redacted], [redacted]
1	22	1 h post dose (± 5 min)	DYP688, SDZ475, [redacted], [redacted]
2	1	Pre-dose (0 h)	DYP688, SDZ475, [redacted], [redacted], IG
2	1	1 h post dose (± 5 min)	DYP688, SDZ475, [redacted], [redacted]
2	15	Pre-dose (0 h)	DYP688, SDZ475, [redacted], [redacted], IG
2	15	1 h post dose (± 5 min)	DYP688, SDZ475, [redacted], [redacted]
3	1	Pre-dose (0 h)	DYP688, SDZ475, [redacted], [redacted], IG
3	1	1 h post dose (± 5 min)	DYP688, SDZ475, [redacted], [redacted]
3	1	4 h post dose (± 1 h)	DYP688, SDZ475, [redacted], [redacted]
3	2	24 h post dose (± 2 h)	DYP688, SDZ475, [redacted], [redacted]
3	4	72 h post dose (± 4 h)	DYP688, SDZ475, [redacted], [redacted]

Cycle	Day	Schedule time (hours)	Analytes
3	8	Pre-dose (0 h)	DYP688, SDZ475, [redacted], [redacted]
3	8	1 h post dose (± 5 min)	DYP688, SDZ475, [redacted], [redacted]
4	1	Pre-dose (0 h)	DYP688, SDZ475, [redacted], [redacted], IG
5	1	Pre-dose (0 h)	DYP688, SDZ475, [redacted], [redacted], IG
6	1	Pre-dose (0 h)	DYP688, SDZ475, [redacted], [redacted], IG
EOT			DYP688, SDZ475, [redacted], [redacted], IG
Unscheduled			DYP688, SDZ475, [redacted], [redacted], IG

Table 8-16 Pharmacokinetic and Immunogenicity blood collection log for DYP688 single agent treatment in Phase II (QW dosing schedule) [1,2]

Cycle	Day	Schedule time (hours)	Analytes
1	1	Pre-dose (0 h)	DYP688, SDZ475, [redacted], [redacted], IG
1	1	1 h post dose (± 5 min)	DYP688, SDZ475, [redacted], [redacted]
1	2	24 h post dose (± 2 h)	DYP688, SDZ475, [redacted], [redacted]
1	4	72 h post dose (± 4 h)	DYP688, SDZ475, [redacted], [redacted]
1	8	Pre-dose (0 h)	DYP688, SDZ475, [redacted], [redacted]
1	8	1 h post dose (± 5 min)	DYP688, SDZ475, [redacted], [redacted]
1	15	Pre-dose (0 h)	DYP688, SDZ475, [redacted], [redacted], IG
1	15	1 h post dose (± 5 min)	DYP688, SDZ475, [redacted], [redacted]
1	16-18	At time of biopsy	DYP688, SDZ475, [redacted], [redacted]
1	22	Pre-dose (0 h)	DYP688, SDZ475, [redacted], [redacted]
1	22	1 h post dose (± 5 min)	DYP688, SDZ475, [redacted], [redacted]
2	1	Pre-dose (0 h)	DYP688, SDZ475, [redacted], [redacted], IG
2	1	1 h post dose (± 5 min)	DYP688, SDZ475, [redacted], [redacted]

Cycle	Day	Schedule time (hours)	Analytes
2	15	Pre-dose (0 h)	DYP688, SDZ475, [redacted], [redacted], IG
2	15	1 h post dose (± 5 min)	DYP688, SDZ475, [redacted], [redacted]
3	1	Pre-dose (0 h)	DYP688, SDZ475, [redacted], [redacted], IG
3	1	1 h post dose (± 5 min)	DYP688, SDZ475, [redacted], [redacted]
3	2	24 h post dose (± 2 h)	DYP688, SDZ475, [redacted], [redacted]
3	4	72 h post dose (± 4 h)	DYP688, SDZ475, [redacted], [redacted]
3	8	Pre-dose (0 h)	DYP688, SDZ475, [redacted], [redacted]
3	8	1 h post dose (± 5 min)	DYP688, SDZ475, [redacted], [redacted]
4	1	Pre-dose (0 h)	DYP688, SDZ475, [redacted], [redacted], IG
5	1	Pre-dose (0 h)	DYP688, SDZ475, [redacted], [redacted], IG
6	1	Pre-dose (0 h)	DYP688, SDZ475, [redacted], [redacted], IG
EOT			DYP688, SDZ475, [redacted], [redacted], IG
Unscheduled			DYP688, SDZ475, [redacted], [redacted], IG

[1] Post dose = post end of infusion; Pre-dose = before start of infusion

[2] Pharmacokinetics and Immunogenicity blood samples will not be collected for adolescent patients if total required blood volumes would exceed maximum volumes restricted by local law.

8.5.1.2 Analytical methods

Concentrations of DYP688 (as total antibody) in human serum will be determined with a validated ligand binding assay. Concentrations [redacted] will be analyzed in plasma with a validated assay using an incubation with papain followed by liquid chromatography-tandem mass spectrometry (LC-MS/MS) analysis. Concentrations of [redacted] SDZ475 [redacted] in blood will be determined with a validated LC-MS/MS assay. Anti-drug antibodies will be analyzed with a validated ligand binding assay in a tiered approach (screening, confirmation, titer assay).

Concentrations below the LLOQ will be reported as 0.00 ng/mL and missing samples will be labeled accordingly.

8.5.2 Biomarkers

Biomarker analyses will be used to investigate the effect of study therapies at the molecular and cellular level as well as to determine how changes in the markers may relate to exposure and clinical outcomes. In addition, potential predictive markers of efficacy, as well as mechanisms of resistance may be explored.

While the goal of the biomarker assessments is to provide supportive data for the clinical study, there may be circumstances when a decision is made to stop a collection, or not perform or discontinue an analysis due to either practical or strategic reasons (e.g., inadequate sample number, issues related to the quality of the sample or issues related to the assay that preclude analysis, impossibility to perform correlative analyses, etc.). Therefore, depending on the results obtained during the study, sample collection analysis may be omitted at the discretion of Novartis. If a particular assay is not available for samples collected in a particular region/country, sample collection and/or analysis may be omitted at the discretion of Novartis. In such cases, the sample collections are not required. Additionally, other related biomarkers may also be analyzed from the samples listed in Table 8-17, depending on sample availability, resources, patient's outcomes and as new scientific evidence or alternative methods become available.

The sample collection information must be entered on the appropriate sample collection eCRF page(s) and requisition form(s). Detailed instructions for the collection, processing, and shipment of biomarker samples are outlined in the laboratory manual for the study. Sample(s) should be collected at the visit/time point(s) defined in the biomarker table; Table 8-17.

Table 8-17 Biomarker sample collection plan

Sample Type	Volume	Visit	Marker	Purpose
**Tumor Samples**				
Archival tumor<br><br>Newly obtained tumor sample	**Archival tumor**: Block (preferred) or a minimum of 20 newly cut sections (4um thickness)<br><br>**Newly obtained tumor**: 3-6 passes of core needle biopsy	Screening[1]<br><br>Anytime between C1D16-C1D18 (Q2W and QW dosing regimen)<br><br>Anytime between C2D2-C2D4<br>(Q4W dosing regimen)<br><br>Disease Progression +14 days and prior to starting new	Expression of markers such as:<br>PMEL17<br>PERK<br>CD8<br>PDL1<br><br>Gene expression of DUSP6 &/or RASGRP3<br><br>Cancer-related genes by next generation DNA sequencing	Presence of ADC target confirmation<br><br>Spatial determination & extent of target inhibition<br><br>Assess expression status of potential predictors of efficacy.<br>Pharmacodynamic markers

Sample Type	Volume	Visit	Marker	Purpose
		treatment (optional)	Whole transcriptome analysis; expression of immune and cancer related genes	Assess potential predictive markers of response & mechanisms of resistance
[1] Archival tumor and a copy of a de-identified pathology report, may be permitted if it meets the inclusion criteria as outlined in Section 5.1				
**Blood samples**				
Blood sample for cfDNA[2]	10 mL per visit	C1D1 pre-infusion<br>C1D8<br>C2D1<br>C3D1<br>C5D1<br>End of Treatment	DNA sequencing in cell free DNA	Assessing cfDNA as surrogate for<br>determination of driver mutation status, predictive pharmacodynamics, for mutational burden and resistance markers in tumor biopsy DNA
[2] Blood samples for cfDNA will not be collected for adolescent patients if total required blood volumes would exceed maximum volumes restricted by local law.				

8.5.2.1 Tissue Collection

Newly obtained pre- and on-treatment paired tumor samples are required and are collected if medically feasible, at screening, on-treatment and disease progression (optional) as indicated in Table 8-17. An archival tumor and a copy of the corresponding de-identified pathology report may be submitted at the screening visit in place of a newly obtained biopsy provided the biopsy meets the criteria outlined in Section 5.1. Otherwise, a newly obtained biopsy at screening is required, if medically feasible. The timing for the on-treatment biopsy may be adjusted based on cases by case basis for logistical reasons, following a discussion with Novartis.

The status of several immune checkpoint targets, target modulation and cell populations may be analyzed in tumor tissue. Expression and localization of biomarkers including but not limited to PMEL17 and pERK may be measured by IHC or using additional techniques deemed suitable. Tumor specimens will be examined for expression of DUSP6 & RASGRP3 in mRNA. Sequencing of cancer related genes may be performed in tumor. Tumor may be sequenced at screening, on-treatment, and end of treatment.

8.5.2.2 Blood Collection

Peripheral blood will be collected pre-treatment and during treatment as indicated in Table 8-17.

Blood will be collected on treatment and at end of treatment to allow for sequence analysis of cfDNA. This analysis will explore the presence of emerging, existing, and resistance mutations

and potentially the tumor mutation burden in tumor and cfDNA at different time points and investigate their relationship with clinical response and the development of tumor resistance.

8.5.2.3 Additional biomarker assessments

During the study, in addition to the biomarkers specified above, exploratory biomarker research may be conducted on any remaining biomarker and/or PK samples. These studies would extend the search for other potential relevant biomarkers for the study drug effect and/or safety. This may also include the development of ways to detect, monitor or treat cancer. These additional investigations would be dependent upon clinical outcome, reagent and sample availability.

8.6 Optional additional research

If the participant agrees, by signing the optional consent for Additional Research, data and/or biological samples that remain after analysis is completed may be used for additional research to help develop ways to detect, monitor or treat human diseases and/or learn more about related human diseases. A decision to perform such exploratory research studies would be based on outcome data from this study or from new scientific findings related to the drug class or disease, as well as assay availability.

9 Discontinuation and completion

9.1 Discontinuation

9.1.1 Discontinuation from study treatment

Discontinuation of study treatment for a patient can be initiated by either the patient or the investigator.

The investigator must discontinue study treatment for a given patient if, he/she believes that continuation would negatively impact the patient's well-being.

Study treatment must be discontinued under the following circumstances:

- Patient/guardian decision
- Any situation in which study participation might result in a safety risk to the patient (see Table 6-4 for criteria for permanent discontinuation for adverse drugs reactions).
- Pregnancy
- Death

If discontinuation of study treatment occurs by patients or guardian decision, the investigator should make a reasonable effort to understand the primary reason for the patient's premature discontinuation of study treatment and record this information.

Patients who discontinue study treatment or who decide they do not wish to participate in the study further should not be considered withdrawn from the study unless they withdraw their consent (see Section 9.1.2). Where possible, they should return for the assessments indicated in the assessment schedule. If they fail to return for these assessments for unknown reasons, every

effort (e.g. telephone, e-mail, letter) should be made to contact the patient/pre-designated contact as specified in the lost to follow-up section (Section 9.1.3). This contact should preferably be done according to the study visit schedule.

If the patient cannot or is unwilling to attend any visit(s), the site staff should maintain regular telephone contact with the patient, or with a person pre-designated by the patient. This telephone contact should preferably be done according to the study visit schedule.

After study treatment discontinuation, at a minimum, in abbreviated visits, the following data should be collected at clinic visits or via telephone/email contact:

- new / concomitant treatments
- adverse events/Serious Adverse Events

For patients who discontinue treatment for reasons other than documented disease progression, death, lost to follow-up, or withdrawal of consent, tumor assessments must continue to be performed every 12 weeks until documented disease progression, death, lost to follow-up, start of new anti-neoplastic therapy or withdrawal of consent.

9.1.2 Discontinuation from study

Discontinuation from study is when the patient permanently stops receiving the study treatment, and further protocol-required assessments or follow-up, for any reason.

Patients who transfer into another study or use an alternative means to continue provision of study treatment, will perform the end of treatment procedures. If the patient agrees, a final evaluation at the time of the patient's study discontinuation should be made as detailed in the assessment table (refer to Section 8).

Novartis will make every effort to supply DYP688 to patients who may benefit from continued treatment as per the Investigator's opinion; safety will be monitored and reported to Health Authorities per regulatory requirements.

9.1.3 Lost to follow-up

For patients whose status is unclear because they fail to appear for study visits without stating an intention to discontinue from study treatment or discontinue from study or withdraw consent/oppose the use of their data/biological samples, the investigator must show "due diligence" by documenting in the source documents steps taken to contact the patient, e.g. dates of telephone calls, registered letters, etc. A patient should not be considered as lost to follow-up until due diligence has been completed or until the end of the study.

9.2 Withdrawal of informed consent/Opposition to use data/biological samples

Withdrawal of consent/opposition to use data/biological samples occurs when a patient:

- Explicitly requests to stop use of their biological samples and/or data (opposition to use patient's data and biological samples)

and

- No longer wishes to receive study treatment

and

- Does not want any further visits or assessments (including further study-related contacts)

This request should be in writing (depending on local regulations) and recorded in the source documentation.

In this situation, the investigator should make a reasonable effort (e.g. telephone, e-mail, letter) to understand the primary reason for the patient's decision to withdraw their consent/opposition to use data/biological samples and record this information.

Where consent to the use of Personal and Coded Data is not required in a certain country's legal framework, the patient therefore cannot withdraw consent. However, they still retain the right to object to the further collection or use of their Personal Data.

Study treatment must be discontinued and no further assessments conducted, and the data that would have been collected at subsequent visits will be considered missing.

Further attempts to contact the patient are not allowed unless safety findings require communicating or follow-up.

If the patient agrees, a final evaluation at the time of the patient's withdrawal of consent/opposition to use data/biological samples should be made as detailed in the assessment table (refer to Section 8).

Further details on withdrawal of consent or the exercise of patients' data privacy rights are included in the corresponding informed consent form.

9.3 Study completion and post-study treatment

Study completion is defined as when the last patient finishes their study completion visit and any repeat assessment associated with this visit and supporting the collection of data for the primary outcome measure, have been documented and followed up appropriately by the Investigator or, in the event of an early study termination decision, the date of that decision.

9.3.1 End of Study

The end of study is defined when the last patient has the last trial related visit/contact. This could happen at the earliest occurrence of one of the following:

- All patients have discontinued study treatment and completed the 60-day safety follow-up and at least 80% of the patients per group have died, withdrawn consent, or are lost to follow-up or completed the follow up.

or

- The study is terminated early (Section 9.4)

or

- Another clinical study becomes available that can continue to provide the study treatment and all patients ongoing are transferred to that clinical study. Note: For patients who transfer to another clinical study or an alternative treatment option to continue provision of study treatment, the follow-up for safety, disease progression, and survival will not be performed.

The follow-up for disease progression and survival might not be performed or pursued if Novartis decides to stop enrollment prematurely. In such cases, end of study will be when the treatment period and the safety follow-up have been completed for all patients.

9.3.2 Post End of Treatment Safety Follow-up

As of protocol amendment 5, the safety follow-up period was increased from 30 to 60 days based on DYP688 preliminary PK which is approximately five half-lives of DYP688. All patients must have safety evaluations at 30 days (+ 7 day window) and 60 days (+ 7 day window) after the last dose administration of DYP688. Evaluations can be done either by telephone call or visit for the 30-day and 60-day safety follow-up visits. Concomitant medications will be collected until the 60-day safety follow-up visit has been completed or the start of a new antineoplastic therapy, whichever occurs first. For female patients of child-bearing potential, a pregnancy test will be performed as outlined in Section 8.4.3.

9.3.3 Disease progression follow-up

Patients who discontinue study treatment for any reasons other than documented disease progression, death, lost to follow-up, or withdrawal of consent, will be followed up for disease progression according to the criteria described in Section 8.3.1.3.

If patients refuse to return for these visits or are unable to do so, every effort should be made to contact them or a knowledgeable informant by telephone to determine if the patient had disease progression.

Once the Follow up for Disease progression period ended, the End of Post treatment Phase disposition eCRF should be completed.

9.3.4 Survival follow-up

After completion of the safety follow-up and/or disease progression follow up, patients will enter the survival follow-up period. Patients will be contacted by telephone every 12 weeks to

follow-up on their survival status. The survival follow up will continue until all patients have discontinued study treatment and either completed the 60-day safety follow up period from the last patient first visit (LPFV), or at least 80% of the patients have died, withdrawn consent, or been lost to follow up. Any new anti-neoplastic therapies that have been started since the last contact date will also be collected during these phone calls. Disease progression on new antineoplastic therapies will also be collected.

9.4 Early study termination by the sponsor

The study can be terminated by Novartis at any time.

Reasons for early termination:

- Unexpected, significant, or unacceptable safety risk to subjects enrolled in the study, including excessive fatal AEs related to DYP688
- Decision based on recommendations from applicable board(s) after review of safety and efficacy data
- Discontinuation of study drug development

In taking the decision to terminate, Novartis will always consider subject welfare and safety. Should early termination be necessary, subjects must be seen as soon as possible and treated as a prematurely withdrawn subject. The investigator may be informed of additional procedures to be followed in order to ensure that adequate consideration is given to the protection of the subject's interests. The investigator or sponsor depending on local regulation will be responsible for informing IRBs/IECs of the early termination of the trial.

9.5 Study stopping rules

During the phase II part of the study, safety review of all available data will be performed by the Sponsor at the time of planned interim analyses (as per Section 12.7) or at least twice per year. If any of the following criteria are met for a Phase II group, then enrollment to that Phase II group will be paused and no new patients will be started on treatment in that group pending review of data by the DMC and a decision from the DMC for how to proceed:

- More than 50% of patients require dose reduction of DYP688 due to treatment-related AEs
- More than 25% of patients require permanent dose discontinuation of DYP688 due to treatment-related AEs
- The Sponsor considers that the number and/or severity of AEs, abnormal safety monitoring tests, or abnormal laboratory findings justify pausing the Phase II group

Upon evaluation of all relevant data, if the DMC and Novartis agree that the treatment is safe, enrollment can resume at the same dose. In the event that a dose is not deemed acceptable for newly enrolled patients, a new lower dose and/or regimen (i.e. a new RD) that satisfies the EWOC criteria might be explored. In this scenario, ongoing patients may continue treatment at the same dose level at the discretion of the investigator and Novartis if it is in the best interest of the patient.

10 Safety monitoring, reporting and committees

10.1 Definition of adverse events and reporting requirements

10.1.1 Adverse events

An adverse event (AE) is any untoward medical occurrence (e.g. any unfavorable and unintended sign [including abnormal laboratory findings], symptom or disease) in a clinical investigation patient after providing written informed consent for participation in the study. Therefore, an AE may or may not be temporally or causally associated with the use of a medicinal (investigational) product.

The investigator has the responsibility for managing the safety of individual patient and identifying adverse events.

Novartis qualified medical personnel will be readily available to advise on trial related medical questions or problems.

The occurrence of adverse events must be sought by non-directive questioning of the patient at each visit during the study. Adverse events also may be detected when they are volunteered by the patient during or between visits or through physical examination findings, laboratory test findings, or other assessments.

Adverse events must be recorded under the signs, symptoms, or diagnosis associated with them, accompanied by the following information (as far as possible) (if the event is serious refer to Section 10.1.2):

1. Adverse events will be assessed and graded according to the Common Terminology Criteria for Adverse Events (CTCAE) version 5.0.
2. Causality: its relationship to the study treatment. If the event is due to lack of efficacy or progression of underlying illness (i.e. progression of the study indication) the assessment of causality will usually be ‘Not suspected.’ The rationale for this guidance is that the symptoms of a lack of efficacy or progression of underlying illness are not caused by the trial drug, they happen in spite of its administration and/or both lack of efficacy and progression of underlying disease can only be evaluated meaningfully by an analysis of cohorts, not on a single patient. The causality assessment is one of the criteria used when determining regulatory reporting requirements.
3. Its duration (start and end dates or ongoing) and the outcome must be reported
4. Whether it constitutes a SAE (see Section 10.1.2 for definition of SAE) and which seriousness criteria have been met
5. Action taken regarding with study treatment.

All adverse events must be treated appropriately. Treatment may include one or more of the following:

- Dose not changed
- Dose reduced/increased
- Drug interrupted/permanently discontinued

6. Its outcome (i.e. recovery status or whether it was fatal)

If the event worsens the event should be reported a second time in the CRF noting the start date when the event worsens in toxicity. For grade 3 and 4 adverse events only, if improvement to a lower grade is determined a new entry for this event should be reported in the CRF noting the start date when the event improved from having been Grade 3 or Grade 4.

Conditions that were already present at the time of informed consent should be recorded in medical history of the patient.

Adverse events (including lab abnormalities that constitute AEs) should be described using a diagnosis whenever possible, rather than individual underlying signs and symptoms.

Adverse event monitoring should be continued for at least 60 days following the last dose of study treatment.

Once an adverse event is detected, it must be followed until its resolution or until it is judged to be permanent (e.g. continuing at the end of the study), and assessment must be made at each visit (or more frequently, if necessary) of any changes in severity, the suspected relationship to the interventions required to treat it, and the outcome.

Progression of malignancy (including fatal outcomes), if documented by use of appropriate method (for example, as per RECIST 1.1 criteria for solid tumors), should not be reported as a serious adverse event, except if the investigator considers that progression of malignancy is related to study treatment.

Adverse events separate from the progression of malignancy (for example deep vein thrombosis at the time of progression or hemoptysis concurrent with finding of disease progression) will be reported as per usual guidelines used for such events with proper attribution regarding relatedness to the drug.

Information about adverse drug reactions for the investigational drug can be found in the Investigator's Brochure (IB).

Abnormal laboratory values or test results constitute adverse events only if they fulfill at least one of the following criteria:

- they induce clinical signs or symptoms
- they are considered clinically significant
- they require therapy

Clinically significant abnormal laboratory values or test results must be identified through a review of values outside of normal ranges/clinically notable ranges, significant changes from baseline or the previous visit, or values which are considered to be non-typical in patient with the underlying disease.

10.1.2 Serious adverse events

An SAE is defined as any adverse event [appearance of (or worsening of any pre-existing)] undesirable sign(s), symptom(s), or medical conditions(s) which meets any one of the following criteria:

- fatal

- Life-threatening: life-threatening in the context of a SAE refers to a reaction in which the patient was at risk of death at the time of the reaction; it does not refer to a reaction that hypothetically might have caused death if it were more severe (please refer to the ICH-E2D Guidelines).
- results in persistent or significant disability/incapacity
- constitutes a congenital anomaly/birth defect or fetal death
- requires inpatient hospitalization or prolongation of existing hospitalization, unless hospitalization is for:
  - routine treatment or monitoring of the studied indication, not associated with any deterioration in condition
  - elective or pre-planned treatment for a pre-existing condition that is unrelated to the indication under study and has not worsened since signing the informed consent
  - social reasons and respite care in the absence of any deterioration in the patient's general condition
  - treatment on an emergency outpatient basis for an event not fulfilling any of the definitions of a SAE given above and not resulting in hospital admission
- is medically significant, e.g. defined as an event that jeopardizes the patient or may require medical or surgical intervention to prevent one of the outcomes listed above

Medical and scientific judgment should be exercised in deciding whether other situations should be considered serious reactions, such as important medical events that might not be immediately life threatening or result in death or hospitalization but might jeopardize the patient or might require intervention to prevent one of the other outcomes listed above. Such events should be considered as "medically significant." Examples of such events are intensive treatment in an emergency room or at home for allergic bronchospasm, blood dyscrasias, or convulsions that do not result in hospitalization or development of dependency or abuse (please refer to the ICH-E2D Guidelines).

All new malignant neoplasms will be assessed as serious under "medically significant" if other seriousness criteria are not met and the malignant neoplasm is not a disease progression of the study indication.

Any suspected transmission via a medicinal product of an infectious agent is also considered a serious adverse reaction.

All reports of intentional misuse and abuse of the product are also considered serious adverse event irrespective if a clinical event has occurred.

10.1.3 SAE reporting

To ensure patient safety, every SAE, regardless of causality, occurring after the patient has provided informed consent and until 60 days after the last dose of DYP688 must be reported to Novartis safety immediately, without undue delay, but under no circumstances later than within 24 hours of obtaining knowledge of the events (Note: If more stringent, local regulations regarding reporting timelines prevail). Detailed instructions regarding the submission process and requirements are to be found in the investigator folder provided to each site. Information

about all SAEs is collected and recorded on the electronic Serious Adverse Event Report Form with paper back-up; all applicable sections of the form must be completed in order to provide a clinically thorough report.

Screen Failures: SAEs occurring after the patient has provided informed consent until the time the patient is deemed a Screen Failure must be reported to Novartis.

Enrolled patients: SAEs collected between time patient signs ICF until 60 days after the patient has discontinued from study treatment.

The investigator must review and provide an assessment of causality for each SAE. There may be situations in which an SAE has occurred, and the investigator has minimal information to include in the initial report to Novartis. However, it is very important that the investigator always makes an assessment of causality for every event before the initial transmission of the SAE data to Novartis. The investigator may change their opinion of causality in light of follow-up information and send an SAE follow-up report with the updated causality assessment.

All follow-up information for the SAE including information on complications, progression of the initial SAE and recurrent episodes must be reported as follow-up to the original episode immediately, without undue delay, but under no circumstances later than 24 hours of the investigator receiving the follow-up information (Note: If more stringent, local regulations regarding reporting timelines prevail). An SAE occurring at a different time interval or otherwise considered completely unrelated to a previously reported one must be reported separately as a new event.

If the SAE is not previously documented in the Investigator's Brochure or Package Insert (new occurrence) and is thought to be related to the study treatment, Novartis may urgently require further information from the investigator for health authority reporting. Novartis may need to issue an Investigator Notification (IN) to inform all investigators involved in any study with the same study treatment that this SAE has been reported.Suspected Unexpected Serious Adverse Reactions (SUSARs) will be collected and reported to the competent authorities and relevant ethics committees in accordance with national regulatory requirements in participating countries, including EU Clinical Trial Regulation 536/2014.

Treatment-emergent elevations of a certain magnitude in AST or ALT in combination with total bilirubin or jaundice in the absence of cholestasis or other causes of hyperbilirubinemia can be an indicator of severe drug induced liver injury (Hy's Law).

For patients with normal AST and ALT values at baseline, these thresholds are defined as

- AST or ALT >3x ULN, combined with
- total bilirubin >2x ULN or jaundice, in the absence of cholestasis or other causes of hyperbilirubinemia. Absence of cholestasis is defined as ALP <2x ULN.

For patients with abnormal AST and ALT values at baseline, these thresholds are modified to

- ALT or AST >3x baseline OR ALT or AST >8x ULN, whichever is lower combined with
- total bilirubin >2x baseline AND >2x ULN or jaundice, in the absence of cholestasis or other causes of hyperbilirubinemia. Absence of cholestasis is defined as ALP <2x ULN.

For this reason, a potential Hy's Law case requires expedited reporting, and will be handled as a serious unexpected adverse event (assessing it as medically significant in the absence of any other seriousness criteria). It must be reported as an SAE to the sponsor promptly (i.e., even before all other possible causes of liver injury have been excluded). Reporting should include all available information, especially that needed for evaluating the diagnosis, severity and likelihood that the study treatment caused the reaction. For patient monitoring and to better understand potential etiologies, the investigator must initiate a close follow-up until complete resolution of the problem and completion of all attempts to obtain supplementary data.

Any SAEs experienced after the 60-day period after the last dose of DYP688 should only be reported to Novartis Safety if the investigator suspects a causal relationship to study treatment, unless otherwise specified by local law/regulations.

10.1.4 Pregnancy reporting

Pregnancies

If a female trial patient becomes pregnant, the study treatment should be stopped, and the pregnancy consent form should be presented to the trial patient. The patient must be given adequate time to read, review and sign the pregnancy consent form. This consent form is necessary to allow the investigator to collect and report information regarding the pregnancy. To ensure patient safety, each pregnancy occurring after signing the informed consent must be reported to Novartis within 24 hours of learning of its occurrence. The pregnancy should be followed up to determine outcome, including spontaneous or voluntary termination, details of the birth, and the presence or absence of any birth defects, congenital abnormalities, or maternal and/or newborn complications.

Pregnancy should be recorded and reported by the investigator to Novartis. Pregnancy follow-up should be recorded on the same form and should include an assessment of the possible relationship to the study treatment any pregnancy outcome. Any SAE experienced during pregnancy must be reported.

If a female partner of a male trial patient who took study treatment in this study becomes pregnant, pregnancy outcomes should be collected. Consent to report information regarding these pregnancy outcomes should be obtained from the mother.

After consent is provided, the pregnancy reporting will occur up to one year after the estimated date of delivery.

10.1.5 Reporting of study treatment errors, misuse/abuse, and overdose

Study treatment errors are unintentional errors in the prescribing, dispensing, and administration of study treatment.

Study treatment misuse refers to situations where the study treatment is intentionally and inappropriately used not in accordance with the protocol.

Study treatment abuse corresponds to the persistent or sporadic, intentional excessive use of a study treatment, which is accompanied by harmful physical or psychological effects.

Study treatment errors and uses outside of what is foreseen in the protocol, including the misuse or abuse will be recorded on the AE (or SAE, if the event meets the definition of SAE) CRF.

For more information on AE and SAE definition and reporting requirements, please see the respective sections (Section 10.1.1, Section 10.1.2 and Section 10.1.3).

10.2 Additional Safety Monitoring

Not applicable.

10.3 Committees

10.3.1 Data Monitoring Committee

A data monitoring committee will not be used for the dose escalation part of this study. Novartis will host investigator meetings on a regular basis during the study. Further, Novartis and the investigators will meet at the end of each treatment cohort in phase I to discuss and evaluate all of the gathered safety data. At the dose escalation meeting the clinical course (safety information including both DLTs and all CTCAE Grade 2 or higher toxicity data during the DLT evaluation period (Cycle 1), and available PK data) for each patient in the current dose cohort will be described in detail. Updated safety data on other ongoing patients, including data in later cycles, may be discussed as well.

Dose escalation decisions will be based on a clinical synthesis of all relevant available data and not solely on DLT information. Selection of the actual dose for the next cohort of patients will be guided by recommendations from the BLRM model (with EWOC), and a medical review of relevant clinical, PK and laboratory data. Novartis and the investigators must reach a consensus on whether to declare MTD, escalate the dose, de-escalate the dose and/or recruit an additional cohort of patients at the current dose level.

A data monitoring committee (DMC), which will function independently of all other individuals associated with the conduct of this clinical trial (including the site investigators participating in the study), will be instituted for the phase II part of the study. The DMC will assess at defined intervals the progress of the clinical trial, safety data, and critical efficacy variables as specified in the DMC Charter and recommend to the sponsor whether to continue, modify, or terminate the trial.

The DMC will be constituted after the decision to initiate phase II is taken and prior to the enrollment of the first patient into the Phase II part of the study.

Specific details regarding composition, responsibilities, data monitoring, and meeting frequency, and documentation of DMC reports, minutes, and recommendations will be described in a separate DMC charter that is established between the sponsor and the DMC.

11 Data Collection and Database management

11.1 Data collection

Data not requiring a separate written record will be defined in the protocol and the Assessment Schedule (Table 8-2 and Table 8-3) and can be recorded directly on the CRFs. All other data

captured for this study will have an external originating source (either written or electronic) with the CRF not being considered as source.

All data should be recorded, handled, and stored in a way that allows its accurate reporting, interpretation, and verification.

Designated investigator staff will enter the data required by the protocol into the Electronic Case Report Forms (eCRF). The eCRFs have been built using fully validated secure web-enabled software that conforms to 21 CFR Part 11 requirements, Investigator site staff will not be given access to the EDC system until they have been trained. Automatic validation programs check for data discrepancies in the eCRFs, allow modification and/or verification of the entered data by the investigator staff.

The investigator/designee is responsible for assuring that the data (recorded on CRFs) (entered into eCRF) is complete, accurate, and that entry and updates are performed in a timely manner. The Investigator must certify that the data entered are complete and accurate.

After final database lock, the investigator will receive copies of the patient data for archiving at the investigational site.

All data should be recorded, handled, and stored in a way that allows its accurate reporting, interpretation, and verification.

11.2 Database management and quality control

Novartis personnel (or designated CRO) will review the data entered by investigational staff for completeness and accuracy. Electronic data queries stating the nature of the problem and requesting clarification will be created for discrepancies and missing values and sent to the investigational site via the EDC system. Designated investigator site staff are required to respond promptly to queries and to make any necessary changes to the data.

Concomitant treatments and prior medications entered into the database will be coded using the World Health Organization (WHO) Drug Reference List, which employs the Anatomical Therapeutic Chemical classification system. Medical history/current medical conditions and adverse events will be coded using the Medical Dictionary for Regulatory Activities (MedDRA) terminology.

Once all the necessary actions have been completed and the database has been declared to be complete and accurate, it will be locked and made available for data analysis/moved to restricted area to be accessed by independent programmer and statistician. Any changes to the database after that time can only be made after written agreement by Novartis/Sponsor development management.

11.3 Site monitoring

Before study initiation, at a site initiation visit or at an investigator's meeting, a Novartis representative will review the protocol and data capture requirements (i.e. eSource DDE or eCRFs) with the investigators and their staff. During the study, Novartis employs several methods of ensuring protocol and GCP compliance and the quality/integrity of the sites' data. The field monitor will visit the site to check the completeness of patient records, the accuracy of data capture / data entry, the adherence to the protocol and to Good Clinical Practice, the

progress of enrollment, and to ensure that study treatment is being stored, dispensed, and accounted for according to specifications.

Source documents provide evidence for the existence of the patient and substantiate the integrity of the data collected. Source documents are filed at the Investigator's site.

Data reported on the CRF or entered in the CRF that are transcribed from source documents must be consistent with the source documents or the discrepancies must be explained. The Investigator may need to request previous medical records or transfer records.

The Investigator must give the monitor access to all relevant source documents to confirm their consistency with the data capture and/or data entry.

The Investigator must maintain source documents for each patient in the study, consisting of case and visit notes (hospital or clinic medical records) containing demographic and medical information, laboratory data, electrocardiograms, and the results of any other tests or assessments. All information on CRFs must be traceable to these source documents in the patient's file. The Investigator must also keep the original informed consent form signed by the patient (a signed copy is given to the patient).

Study monitors will perform source data verification to confirm that data entered into the CRF by authorized site personnel are accurate, complete, and verifiable from source documents; that the safety and rights of patients are being protected; and that the study is being conducted in accordance with the currently approved protocol and any other study agreements, ICH GCP, and all applicable regulatory requirements.

Key study personnel must be available to assist the field monitor during these visits. Continuous remote monitoring of each site's data may be performed by a centralized Novartis organization. Additionally, a central analytics organization may analyze data & identify risks & trends for site operational parameters, and provide reports to Novartis clinical teams to assist with trial oversight.

11.4 Data protection

Participants will be assigned a unique identifier. Any participant records or datasets that are transferred to Novartis will contain the identifier only; participant names or any information which would make the participant identifiable will not be transferred.

The participant must be informed that his/her personal study-related data will be used by Novartis in accordance with local data protection law. The level of disclosure must also be explained to the participant who will be required to give consent for their data to be used as described in the informed consent

The participant must be informed that his/her medical records may be examined by Clinical Quality Assurance auditors or other authorized personnel appointed by Novartis, by appropriate IRB/IEC members, and by inspectors from regulatory authorities.

Novartis has appropriate processes and policies in place to handle personal data breaches according to applicable privacy laws.

Any data security breach that may impact the confidentiality and protection of study patients' data is recorded and investigated by Novartis as required by applicable laws or regulations and as adequate to the nature of the breach. Upon identification of any potential data security breach, including the categories of data impacted, the data security breach management process may include investigation of the root cause of the incident, and, on a basis of its outcome, determination of the adequate mitigation actions to address any immediate impact, and corrective and preventive measures to prevent recurrence and mitigate any adverse effects, as applicable. The data security breach will be reported to the regulatory authorities and Ethic Committees or IRBs as required by the applicable laws or regulations.

12 Data analysis and statistical methods

Data from participating centers in this protocol will be combined, so that an adequate number of patients will be available for analysis. Data will be summarized using descriptive statistics (continuous data) and/or contingency tables (categorical data) for demographic and baseline characteristics, and efficacy, safety, pharmacokinetic, and pharmacodynamics measurements.

Study data may be analyzed and reported in a primary CSR(s) based on all patients' data up to the time when all patients in the corresponding phase II group have either progressed, or died, or withdrawn consent, or been lost to follow-up, or started a new antineoplastic therapy or have been followed-up for at least 6 months after treatment start, whichever occurs earlier. Any additional data for patients continuing to receive study treatment past the data cut-off date for the primary CSR, as allowed by the protocol, will be reported at completion of the study in a final CSR. The primary CSR for the phase II groups may be separated, if considered appropriate, depending upon the differential enrollment rate and difference in sample size for the different Phase II groups.

The following rules will be followed for reporting results unless stated otherwise:

- For the dose escalation part, cohorts treated with the same dosing regimen (dose and dosing schedule) of DYP688 single agent will be pooled into one single treatment group. All summaries, listings, figures and analyses will be performed by treatment group.
- For the phase II part, all summaries, listings, figures for primary efficacy analysis and safety analysis will be presented by patient group.

Patients from dose escalation and the Phase II part will not be pooled in any analyses unless otherwise specified.

Screen failure patients, as described in Section 8.1.1, and the reasons for not starting the study treatment will be reported in a listing, but will not be included in any analyses.

Details of the statistical analysis and data reporting will be provided in the Statistical Analysis Plan (SAP).

Any data analysis carried out independently by the investigator should be submitted to Novartis before publication or presentation.

12.1 Analysis sets

12.1.1 Full Analysis Set (FAS) and Safety Set (SS)

The Full Analysis Set (FAS) and Safety Set (SS) comprise all patients who received at least one dose of study treatment. Patients will be analyzed according to the study treatment received, defined as the assigned treatment if the patient took at least one dose of that treatment or the first treatment received if the assigned treatment was never received.

12.1.2 Dose-determining analysis set

The Dose-Determining Set (DDS) includes all FAS patients who met the minimum exposure criterion and have sufficient safety evaluations (as determined by Novartis and Investigator), or experienced a dose limiting toxicity (DLT) during the DLT-evaluation period (Cycle 1, i.e. the first 28 days).

A patient has met the minimum exposure criterion if she/he received the minimum number of doses as per the assigned regimen described in Table 12-1 during the DLT evaluation period.

A patient is considered evaluable at the assigned dose level if they have received the minimum number of planned doses as described in Table 12-1 during the DLT evaluation period.

Patients in the Dose-Determining Set (DDS) will be summarized according to the treatment most frequently taken between Study Day 1 and the end of the DLT evaluation period (Cycle 1, i.e. the first 28 days), the onset of a DLT or discontinuation from study treatment, whichever occurs first.

Table 12-1 Minimum exposure criteria for potential dosing regimen

Study drug	Dosing regimen	Minimum number of doses in DLT evaluation period*
DYP688	Every 2 weeks (Q2W)	2
DYP688	Every 4 weeks (Q4W)	1
DYP688	Every 1 week (QW)	3

* A dose is considered to be taken as planned dose if the dose administered is greater than or equal to the original prescribed dose.

Patients who do not experience a DLT during cycle 1 (i.e. the first 28 days) are considered to have sufficient safety evaluations if they have been observed for ≥ 28 days, as applicable, following the first dose, and are considered by both the sponsor and investigators to have enough safety data to conclude that a DLT did not occur.

12.1.3 Pharmacokinetic Analysis Set (PAS)

The pharmacokinetic analysis set (PAS) includes all patients who provide at least one evaluable PK concentration. For a concentration to be evaluable, a patient must:

- Receive at least one of the planned treatments
- Provide at least one primary PK parameter

12.1.4 Immunogenicity (IG) analysis Set

The Immunogenicity prevalence set includes all patients in the Full analysis set with a determinant baseline IG sample or at least one determinant post-baseline IG sample.

The Immunogenicity incidence set includes all patients in the Immunogenicity prevalence set with a determinant baseline IG sample and at least one determinant post-baseline IG sample.

12.2 Patient demographics and other baseline characteristic

Demographic and other baseline data including disease characteristics will be summarized descriptively for the FAS.

Categorical data will be presented as frequencies and percentages. For continuous data, mean, standard deviation, median, minimum, and maximum will be presented.

Relevant medical histories and current medical conditions at baseline will be summarized by system organ class and preferred term.

12.3 Treatments

The Safety Set will be used for the analyses below. Categorical data will be summarized as frequencies and percentages. For continuous data, mean, standard deviation, median, 25th and 75th percentiles, minimum, and maximum will be presented.

The duration of exposure to DYP688 single agent in months as well as the dose intensity (computed as the ratio of actual cumulative dose received and actual duration of exposure) and the relative dose intensity (computed as the ratio of dose intensity and planned dose intensity) will be summarized by means of descriptive statistics using the safety set.

The number of patients with dose adjustments (reductions, interruption, or permanent discontinuation) and the reasons will be summarized by treatment group and all dosing data will be listed.

Concomitant medications and significant non-drug therapies prior to and after the start of the study treatment will be listed by treatment group.

12.4 Analysis supporting primary objectives

Dose escalation part

The primary objective of the dose escalation part is to characterize the safety and tolerability of DYP688 as a single agent, and to identify a recommended regimen(s) (dose(s) and schedule(s)) for phase II part. All safety analysis will be based on the safety set, with the exception of the DLT analysis, which is based on the DDS.

Phase II part

The primary objective of the phase II part is to assess the anti-tumor activity of DYP688 as a single agent. All efficacy analysis will be based on FAS.

12.4.1 Safety endpoints

The primary safety endpoints for the escalation part are listed in Table 2-1.

12.4.1.1 Definition of primary endpoint(s)

The primary endpoints are described in Table 2-1.

12.4.1.2 Statistical hypothesis, model, and method of analysis

For all safety analyses, except the DLT analyses, the safety set will be used. All listings and tables will be presented by treatment group.

The overall observation period will be divided into three mutually exclusive segments:

Pre-treatment period

- From day of patient's first informed consent to the day before first administration of study treatment

On-treatment period

- From date of first administration of study treatment to 30 days after date of last actual administration of study treatment (including start and stop date).

Post-treatment period

- Starting at day 31 after last administration of study treatment

Safety summaries will primarily be based on all data from the on-treatment period. Following last administration of study treatment, AEs (including SAEs), and new antineoplastic therapies are collected for a period of 60 days after the last dose of DYP688.

Dose-limiting toxicities

Estimation of the MTD of the treatment will be based upon the estimation of the probability of DLT in Cycle 1 (first 28 days of treatment) for patients in the DDS. A recommended dose(s) below the MTD may be identified based on other safety, clinical, PK, and PD data.

Bayesian adaptive approach

The dose escalation part of this study will be guided by a Bayesian analysis of Cycle 1 dose limiting toxicity (DLT) data for DYP688. The relationship between dose and the probability of DLT is modelled using logistic regression. Details of the model are given in (Section 16.2).

Additional dosing regimens

In case any additional dosing regimen is to be tested during the study, separate BLRM or BHLRM will be implemented to guide the dose escalation by the EWOC principle for the new dosing regimen. A full description including parameter specification, operating characteristics and hypothetical dose scenarios of the BLRM/BHLRM will be given in a stand-alone

document(s), prior to the first patient is treated on the cohort with new dosing regimen. The additional model specification will also be presented in the primary study report.

Assessment of patient risk

After each cohort of patients, the posterior distribution for the risk of DLT for new patients at doses of interest will be evaluated. The posterior distributions will be summarized to provide the posterior probability that the risk of DLT lies within the following intervals:

Under-dosing:	[0, 0.16)
Targeted toxicity:	[0.16, 0.33)
Excessive toxicity:	[0.33, 1]

The Escalation with Overdose Control (EWOC) Principle

Dosing decisions are guided by the escalation with overdose control principle (Rogatko 2007). A dose may only be used for newly enrolled patients if the risk of excessive toxicity at that dose is less than 25%.

Prior distributions

Weakly informative prior distributions based on pre-clinical information were chosen for the model parameters, see (Section 16.2).

Starting dose

The starting dose is 4 mg/kg DYP688, (Section 6.6.1). A full assessment of the prior risk to patients is given in (Section 16.2).

Reporting

Bayesian analyses will be based on the DDS. DLTs will be listed, and their incidence summarized by primary system organ class and worst grade (CTCAE version 5.0), using the DDS.

Adverse events

All information obtained on adverse events will be displayed by treatment group and patient.

The number (and percentage) of patients with treatment emergent adverse events (events that started after the first dose of study medication or events present prior to start of study treatment but increased in severity based on preferred term) will be summarized in the following ways:

- by treatment, primary system organ class and preferred term.
- by treatment, primary system organ class, preferred term and maximum severity.

Separate summaries will be provided for study treatment related adverse events, death, serious adverse events, other significant adverse events leading to discontinuation from study and adverse events leading to dose adjustment.

A patient with multiple adverse events within a primary system organ class is only counted once towards the total of the primary system organ class.

Summary tables for adverse events (AEs) will include only AEs that started or worsened during the on-treatment period, the **treatment-emergent** AEs.

The incidence of treatment-emergent adverse events (new or worsening from baseline) will be summarized by system organ class and/or preferred term, severity (based on CTCAE grades), type of adverse event, and relationship to study treatment.

Serious adverse events, non-serious adverse events during the on-treatment period will be tabulated.

All deaths (on-treatment and post-treatment) will be summarized.

All AEs, deaths and serious adverse events (including those from the pre and post-treatment periods) will be listed and those collected during the pre-treatment and post-treatment period will be flagged.

Vital signs

All vital signs data will be listed by treatment group, subject and visit/time, abnormalities will be flagged. Summary of notable vital sign values will be provided by treatment.

12-lead ECG

12-lead ECGs including PR, QRS, QT, QTcF, and HR intervals will be obtained for each patient during the study. ECG data will be read and interpreted centrally.

- Summary of notable ECG values.
- Listing of ECG evaluations for all patients with at least one abnormality.

All ECG data will be listed by treatment group, patient and visit/time, and abnormalities will be flagged. Summaries of notable ECG value will be provided by treatment group.

Clinical Laboratory Evaluations

Grading of laboratory values will be assigned programmatically as per NCI Common Terminology Criteria for Adverse Events (CTCAE) version 5.0. The calculation of CTCAE grades will be based on the observed laboratory values only, clinical assessments will not be taken into account.

CTCAE Grade 0 will be assigned for all non-missing values not graded as 1 or higher. Grade 5 will not be used.

For laboratory tests where grades are not defined by CTCAE v5.0, results will be categorized as low/normal/high based on laboratory normal ranges.

The following listings/summaries will be generated separately for hematology, and biochemistry tests:

- Listing of all laboratory data with values flagged to show the corresponding CTCAE version 5.0 grades if applicable and the classifications relative to the laboratory normal ranges

For laboratory tests where grades are defined by CTCAE version 5.0

- Worst post-baseline CTCAE grade (regardless of the baseline status). Each patient will be counted only once for the worst grade observed post-baseline.
- Shift tables using CTCAE version 5.0 grades to compare baseline to the worst on-treatment value

For laboratory tests where grades are not defined by CTCAE version 5.0

- Shift tables using the low/normal/high/ (low and high) classification to compare baseline to the worst on-treatment value.

Tolerability

Tolerability of study drug will be assessed by summarizing the number of and reasons for dose interruption, dose reductions and dose discontinuation by treatment group. Dose intensity will also be tabulated by treatment group. Refer to Section 12.3.

12.4.1.3 Handling of missing values/censoring/discontinuations

Patient safety data will be reported up to the cut-off date, end of post-treatment (if discontinued), death, lost to follow up, or withdrawal of consent/opposition to use data/biological samples, whichever happened first. Missing values will not be imputed and will be reported as such.

12.4.1.4 Sensitivity and Supportive analyses

No formal sensitivity analysis is planned.

12.4.2 Efficacy endpoints

The primary objective of the Phase II part of this study is to assess the anti-tumor activity of DYP688 based on overall response rate (ORR).

12.4.2.1 Definition of primary endpoint(s)

The primary efficacy endpoint of the phase II part of the study is the overall response rate (ORR), defined as the proportion of patients with best overall response (BOR) of complete response (CR) or partial response (PR), as per central assessment according to RECIST 1.1 (see Section 16.1 for details). Best overall response is the best post baseline confirmed overall response observed in a given patient, among the confirmed response categories, excluding "unknown" and "not assessed". Best overall response is determined based on overall responses observed at all post-baseline response assessments, recorded from start of study treatment until PD, death, start of new therapy, withdrawal of consent or end of study, whatever comes first.

12.4.2.2 Statistical hypothesis, model, and method of analysis

FAS will be used for the analysis of primary efficacy endpoint for the phase II part.

The point estimate of ORR along with corresponding exact 95% two-sided confidence intervals according to Clopper-Pearson will be presented by patient group.

The following statistical hypothesis will be tested with each patient group separately:

- H_0: ORR ≤ 10%
- H_1: ORR >10%.

The null hypothesis will be formally rejected if the lower bound of exact 95% two-sided CI for ORR is above 10%. The type I error rate is controlled at 2.5% one-sided individually for each treatment group.

No formal statistical comparison between patient groups is planned in this study. No hypothesis testing will be conducted for the optional third group targeting other GNAQ/11 mutant melanoma.

Detailed statistical considerations, including sample size and power consideration of this study design, are presented in Section 12.8.

12.4.2.3 Handling of the intercurrent events of primary estimand

The primary estimand will account for different key intercurrent events as follows:

- **Discontinuation of the study treatment for any reason other than disease progression by RECIST 1.1 as per local assessment:** Per treatment policy strategy, tumor assessment data collected after discontinuation of study treatment for any reason other than disease progression will be used to derive BOR. Tumor assessments collected after the start of new therapy will not be considered for evaluation of BOR.
- **Start of new anti-neoplastic therapy:** Per while on treatment strategy, tumor assessments collected before start of new anti-neoplastic therapy will be used to derive BOR. Tumor assessments collected on/after the start of new therapy will not be considered for evaluation of BOR. However, a tumor assessment on or after start of new anti-neoplastic therapy can confirm the response observed prior to start of new therapy.
- **Usage of any concomitant medications:** Per treatment policy strategy**,** BOR will be obtained irrespective of any concomitant medications.
- **Any Public health emergency as declared by local or regional authorities i.e. pandemic, epidemic or natural disaster etc**.: Per treatment policy strategy, BOR will take into account all tumor assessments irrespective of any public health emergency.

12.4.2.4 Handling of missing values/censoring/discontinuations

Patients in the FAS with unknown or missing best overall response (BOR) will be noted as such in the appropriate tables/listings and counted as failures in the ORR. If there is no baseline tumor assessment, all post-baseline overall lesion responses are expected to be 'Unknown.' If no valid post-baseline tumor assessments are available, the best overall response must be "Unknown" unless progression is reported.

More details will be provided in the Statistical Analysis Plan (SAP).

12.5 Analysis supporting secondary objectives

The secondary safety endpoints for phase II part used to assess safety and tolerability are described in Table 2-1.

The secondary efficacy endpoints used to assess the preliminary anti-tumor activity of DYP688, for dose escalation part, are BOR and ORR as defined in Section 12.4.2 and for phase II part, are DCR, DOR, PFS, and OS defined below as per RECIST 1.1. All relative listings, tables and figures will be presented by treatment group for dose escalation part and by patient group for phase II part, unless otherwise specified.

12.5.1 Efficacy and/or Pharmacodynamic endpoint(s)

Progression free survival (PFS)

PFS is defined as the time from the date of start of treatment to the date of the first documented progression as per central review and according to RECIST 1.1, or death due to any cause.

In case a patient does not have progression or death prior to data cutoff, PFS will be censored at the date of the last adequate assessment on or prior to the earliest censoring event. The censoring reason could be:

- Ongoing without event
- Lost to follow-up
- Withdrawal consent
- New anticancer therapy
- Event documented after at least two missing tumor assessments
- Adequate assessments no longer available

PFS will be summarized at the RD and for all other treatment groups with at least 10 pts treated, using the Kaplan-Meier method. Median PFS and PFS probability at pre-specified time-points (defined in the SAP) will be presented along with 95% CI using FAS.

Duration of Response (DOR)

DOR applies only to patients with a BOR of CR or PR as assessed by central review as per RECIST 1.1. It is defined as the time from the date of first documented disease response (CR or PR) to the date of first documented progression as assessed by RECIST 1.1 or death due to indication. If a patient has not had an event, duration of overall response is censored at the date of the last adequate assessment.

In case a patient does not have progression or death due to indication prior to data cutoff, DOR follow the same censoring rules as for PFS. Median DOR, with corresponding 95% CI, and 25th and 75th percentiles will be presented. KM estimates for DOR proportions at specific time points, along with 95% CI (Greenwood's formula, Kalbfleisch and Prentice 2002) will also be provided.

Disease control rate (DCR)

DCR is defined as the proportion of patients with a confirmed BOR of CR or PR or SD by central review as per RECIST v1.1. DCR calculated based on the data from the FAS and the corresponding 95% exact Clopper-Pearson confidence interval (Clopper and Pearson 1934) will be presented.

Overall Survival (OS)

OS is defined as the time form date of start of treatment to date of death due to any cause. If a patient is not known to have died, survival will be censored at the date of last contact prior to/on cut-off date.

OS will be summarized using the Kaplan-Meier method. Median OS, with corresponding 95% CI, and 25th and 75th percentiles (Brookmeyer and Crowley 1982, Klein and Moeschberger 1997) will be presented. Kaplan-Meier estimates for OS proportions at specific time points along with 95% CI (Greenwood's formula, Kalbfleisch and Prentice 2002) will also be provided. OS will only be summarized if there are more than 10 patients treated in a treatment group.

12.5.2 Safety endpoints

Ophthalmologic assessments

Full ophthalmic examination includes slit lamp examination, best recorded visual acuity for distance testing, intraocular pressure (IOP), and dilated fundoscopy.

IOP and visual acuity at baseline, worst post-baseline value and changes from baseline of visual acuity scores and IOP will be tabulated. Clinically significant changes in visual acuity will also be tabulated.

New abnormalities identified in indirect (dilated) fundoscopy (ophthalmoscopy) and slit lamp clinical evaluations will be tabulated.

Immunogenicity

All immunogenicity results will be listed by treatment group, patient, and visit/time. Immunogenicity of DYP688 will also be characterized descriptively tabulating antidrug antibody (ADA) prevalence at baseline and ADA incidence on-treatment.

12.5.3 Pharmacokinetics

Descriptive summary statistics of DYP688 plasma concentration data will be provided by treatment group, and visit/sampling time point, including the frequency (n, %) of concentrations below the LLOQ and reported as zero.

Descriptive summary statistics will include mean (arithmetic and geometric), SD, and CV (arithmetic and geometric), median, minimum, and maximum. An exception to this is *Tmax* where median, minimum, and maximum will be presented. Concentration values below LLOQ will be handled as zero in summary statistics, and reported as it in data listings.

Descriptive graphical plots for individual and mean concentration versus time profiles will be generated when applicable.

Pharmacokinetic parameters will be determined by non-compartmental method(s) using pharmacokinetic profile of DYP688. PK parameters listed in Table 12-2 will be derived and reported, when feasible. Additional PK parameters may be estimated if feasible.

Table 12-2 Non-compartmental pharmacokinetic parameters

AUClast	The AUC from time zero to the last measurable concentration sampling time (tlast) (mass x time x volume^{-1})
AUCinf	The AUC from time zero to infinity (mass x time x volume^{-1})
AUCtau	The AUC calculated to the end of a dosing interval (tau) at steady-state (amount x time x volume^{-1})
Cmax	The maximum (peak) observed plasma, blood, serum, or other body fluid drug concentration after single dose administration (mass x volume^{-1})
Ctrough	The predose observed serum drug concentration collected before dose administration (mass × volume^{-1})
Tmax	The time to reach maximum (peak) plasma, blood, serum, or other body fluid drug concentration after single dose administration (time)
T1/2	The elimination half-life associated with the terminal slope (λz) of a semi logarithmic concentration-time curve (time).
CL	The total body clearance of drug from the plasma (volume x time^{-1})
Vz	The apparent volume of distribution during terminal phase (associated with λz) (volume)
Vss	The volume of distribution at steady state (volume)
AR	Accumulation Ratio=AUCtau (multiple Dose)/AUCtau (single dose)

12.6 Analysis of exploratory endpoints

Analysis of exploratory endpoints intended for the Clinical Study Report (CSR) will be detailed in the Statistical Analysis Plan (SAP).

12.6.1 [redacted]

[redacted]

[redacted]

12.6.2 [redacted]

[redacted]

See Section 2 for exploratory objectives and endpoints.

12.7 Interim analyses

Dose escalation part:

No formal interim analyses are planned. However, the dose-escalation design allows that decisions based on the current data are taken before the end of the study. More precisely, after each cohort in the dose escalation, the next dose of study treatment will be selected based on review by Novartis study personnel and Investigators of available safety and tolerability information (including the DLT risk assessment from the BLRM using EWOC) along with PK, PD and efficacy data. Details of this procedure and the process for communication with Investigators are provided in Section 6.6.

Phase II part

IA will only be conducted for MUM patient group. One IA for futility is planned after approximately 15 patients have been treated and have completed at least 2 post-baseline tumor assessments, progressed, died, withdrawn consent, been lost to follow-up, started a new antineoplastic therapy or have been followed up for at least 4 months , whichever comes first. The primary assessment of efficacy at the IA will be based on ORR according to RECIST 1.1 criteria as determined by central assessment. The IA futility decision rule for both groups will be based on Bayesian predictive probability of success (PPoS):

Based on the PPoS that the null hypothesis is formally rejected at the primary analysis, i.e. the lower bound of 95% exact CI of the observed ORR > 10%, given the observed interim data (x) among n patients and the expected additional m patients after the IA (a total of n + m patients of the phase II part of the study). Thus,

$$\text{PPoS} = \text{Prob [Study success at primary analysis} \mid x, n, m]$$
$$= \text{Prob [Lower bound of 95\% exact CI} > 10\% \mid x, n, m]$$

Thus with a total of 40 patients of the phase II part of the study, it translates to

$$= \text{Prob [Observed ORR} \geq 22.5\% \mid x,n].$$

Where the minimum required response observed at primary analysis is 9 (ORR = 22.5%), with the lower bound of 95% exact CI of the observed ORR excluding 10%, i.e., the null hypothesis is formally rejected with a total of 40 patients. PPoS is selected to be 10%, i.e if PPoS is > 10%, the treatment group will continue to enroll as planned. The choice and justification of IA futility threshold is also based on operating characteristics presented in Table 12-4.

The efficacy outcome (ORR) is binary, namely, response (CR or PR) vs. non-response (SD, PD or UNK), and the number of responders on each arm is assumed to follow a binomial distribution. Suppose the prior belief regarding the probability of response (π) is that it has a beta distribution with parameters a_0 and b_0. If there are x responders out of n patients at the IA, then the posterior distribution for π is beta with parameters $a_1=a_0+x$ and $b_1=b_0+n-x$. Now let Y represent the number of successes (i.e. responders) from future observations at the primary

analysis, then the (posterior) predictive distribution of Y conditioned on the observed data at the IA (x responders out of n patients) is a beta-binomial distribution described by

$$p(Y = y \mid x, n) = \binom{m}{y} \frac{B(a_0 + x + y, b_0 + n - x + m - y)}{B(a_0 + x, b_0 + n - x)}$$

where B (a, b) is the beta function.

A minimally informative unimodal Beta (0.11, 1) prior distribution for ORR is used for each treatment arm. This prior distribution has mean 10% and 95% probability interval from 0.00% to 79.6%.

Table 12-3 shows the PPoS with different IA sample size and various number of responders at IA with a total of 40 patients at primary analysis, it demonstrates that with at least 2 responders out of 15 patients at IA per patient group, there will be ≥ 11.9% predictive probability of success (reject null).

Table 12-3 Predictive probability of study success under different IA results (N = 40 at primary analysis)

IA sample size	# of responders observed at IA	observed ORR at IA	PPoS P(study success at primary analysis | IA data)
15	1	6.7%	0.017
	**2**	**13.3%**	**0.119**
	3	20.0%	0.367
	4	26.7%	0.679
	5	33.3%	0.895
18	1	5.6%	0.005
	2	11.1%	0.049
	3	16.7%	0.205
	4	22.2%	0.487
	5	27.8%	0.5014
	6	33.3%	0.938
20	1	5.0%	0.002
	2	10.0%	0.025
	3	15.0%	0.126
	4	20.0%	0.361
	5	25.0%	0.665
	6	30.0%	0.888

Table 12-4 below shows the probability of stopping trial at IA, positive study or negative study at primary analysis for various true ORRs and total number of patients enrolled in the phase II part. For both patient groups, if the true ORR is 30% or more, the probability of success at the primary analysis is ≥ 87% (study power in the presence of IA). While if the true ORR is equal or less than 10%, there will be ≥ 55% probability of stopping trial at IA and the likelihood of rejecting the null hypothesis is very low (under 1% and thus alpha is controlled below 2.5%). Therefore, the proposed trial design is deemed to have desired operating characteristics.

Table 12-4 Operating characteristics for one treatment group – Interim analysis (15 patients), primary analysis (40 patients)

True ORR	Operating characteristics (N = 40)	
	Probability of stopping at IA	Probability of study success at primary analysis
0.05	0.83	< 0.001
0.10	0.55	0.01
0.15	0.32	0.13
0.20	0.17	0.39
0.25	0.08	0.68
0.30	0.04	0.87
0.35	0.01	0.96
0.40	0.01	0.99

The final decision on whether to continue enrollment will primarily be based on the observed ORR using decision rule described above but other efficacy (in particular duration of response), safety, and available biomarker data will be considered as well. Detailed analysis plan for IA will be specified in the SAP.

12.8 [redacted]

12.8.1. [redacted]

[redacted]

CCI [redacted]

Table 12-5 CCI [redacted]

CCI [redacted]

13 Ethical considerations and administrative

13.1 Regulatory and ethical compliance

This clinical study was designed and shall be implemented, executed and reported in accordance with the ICH Harmonized Tripartite Guidelines for Good Clinical Practice, with applicable local regulations (including European Clinical Trial Regulation 536/2014, US CFR 21), and with the ethical principles laid down in the Declaration of Helsinki and Council for International Organizations of Medical Sciences (CIOMS) international ethical guidelines.

13.2 Responsibilities of the investigator and IRB/IEC

Before initiating a trial, the investigator/institution must obtain approval/favorable opinion from the Institutional Review Board/Independent Ethics Committee (IRB/IEC) for the trial protocol, protocol amendments, written informed consent form (ICF), consent form updates, Investigator's Brochure, patient recruitment procedures (e.g., advertisements) and any other written information or relevant documentation to be provided to patients. Any amendments to the protocol will require IRB/IEC and Health Authority approval before implementation of changes made to the study design, except for changes necessary to eliminate an immediate hazard to study patients.

The Investigator will be responsible for the following:

- Signing a protocol signature page confirming his/her agreement to conduct the study in accordance with these documents and all of the instructions and procedures found in this protocol and to give access to all relevant data and records to Novartis/Sponsor monitors, auditors, Novartis/Sponsor Quality Assurance representatives, designated agents of Novartis/Sponsor, IRBs/IECs, and regulatory authorities as required
- Providing written summaries of the status of the study to the IRB/IEC annually or more frequently in accordance with the requirements, policies, and procedures established by the IRB/IEC
- Notifying the IRB/IEC of SAEs or other significant safety findings as required by IRB/IEC procedures
- Taking any immediate action required for the safety of any patient included in this study, even if this action represents a deviation from the protocol. In such cases, Novartis/Sponsor must be notified of this action and the IRB/IEC at the study site must be informed according to local regulations.
- Providing oversight of the conduct of the study at the site and adherence to requirements of 21 CFR, ICH guidelines, the IRB/IEC, European regulation 536/2014 for clinical studies (if applicable), European Medical Device Regulation 2017/745 for clinical device research (if applicable), and all other applicable local regulations
- Informing Novartis/Sponsor immediately if an inspection of the clinical site is requested by a regulatory authority

13.3 Publication of study protocol and results

The protocol will be registered in a publicly accessible database such as clinicaltrials.gov and as required in CTIS public website. In addition, after study completion (defined as last patient last visit) and finalization of the study report the results of this trial will be submitted for publication and posted in a publicly accessible database of clinical trial results, such as the Novartis clinical trial results website and all required Health Authority websites (e.g. Clinicaltrials.gov, CTIS public website etc.).

Summary results of primary and secondary endpoints will be disclosed based upon the global Last Participant Last Visit (LPLV) date, since multinational studies are locked and reported based upon the global LPLV.

For details on the Novartis publication policy including authorship criteria, please refer to the Novartis publication policy training materials that were provided.

Any data analysis carried out independently by the Investigator must be submitted to Novartis before publication or presentation.

13.4 Quality Control and Quality Assurance

Novartis maintains a robust Quality Management System (QMS) that includes all activities involved in quality assurance and quality control, to ensure compliance with written Standard Operating Procedures as well as applicable global/local GCP regulations and ICH Guidelines.

Audits of investigator sites, vendors, and Novartis systems are performed by auditors, independent from those involved in conducting, monitoring or performing quality control of the clinical trial. The clinical audit process uses a knowledge/risk-based approach.

Audits are conducted to assess GCP compliance with global and local regulatory requirements, protocols and internal SOPs, and are performed according to written Novartis processes.

13.5 Data quality assurance

Monitoring strategy, methods, responsibilities, and requirements are provided in the monitoring plan, contracts. Details may include definition of study critical data items and processes (e.g. risk-based initiatives in operations and quality such as risk management and mitigation strategies and analytical risk-based monitoring), including handling of noncompliance issues and monitoring techniques (central, remote, or on-site monitoring)

Records and documents, including signed ICFs, pertaining to the conduct of this study must be retained by the Investigator for 15 years after study completion unless local regulations or institutional policies require a longer retention period. No records may be destroyed during the retention period without the written approval of Novartis. No records may be transferred to another location or party without written notification to Novartis.

13.6 Patient Engagement

The following patient engagement initiatives are included in this study and will be provided, as available, for distribution to study patients at the time points indicated. If compliance is impacted by cultural norms or local laws and regulations, sites may discuss modifications to these requirements with Novartis.

- Thank You letter
- Plain language trial summary - after CSR publication

14 Protocol adherence

This protocol defines the study objectives, the study procedures and the data to be collected on study patients. Additional assessments required to ensure safety of patients should be administered as deemed necessary on a case-by-case basis. Under no circumstances including incidental collection is an investigator allowed to collect additional data or conduct any additional procedures for any purpose involving any investigational drugs under the protocol,

other than the purpose of the study. If despite this interdiction prohibition, data, information, observation would be incidentally collected, the investigator shall immediately disclose it to Novartis and not use it for any purpose other than the study, except for the appropriate monitoring on study patients.

Investigators ascertain they will apply due diligence to avoid protocol deviations. If an investigator feels a protocol deviation would improve the conduct of the study this must be considered a protocol amendment, and unless such an amendment is agreed upon by Novartis and approved by the IRB/IEC and Health Authorities, where required, it cannot be implemented.

14.1 Protocol amendments

Any change or addition to the protocol can only be made in a written protocol amendment that must be approved by Novartis, health authorities where required, and the IRB/IEC prior to implementation.

Only amendments that are required for patient safety may be implemented immediately provided the health authorities are subsequently notified by protocol amendment and the reviewing IRB/IEC is notified.

Notwithstanding the need for approval of formal protocol amendments, the investigator is expected to take any immediate action required for the safety of any patient included in this study, even if this action represents a deviation from the protocol. In such cases, Novartis should be notified of this action and the IRB/IEC at the study site should be informed according to local regulations.

15 References (available upon request)

Aronow ME, Topham AK, Singh AD, et al (2018) Uveal Melanoma: 5- Year Update on Incidence, Treatment, and Survival. Ocul Oncol Pathol.; 4:145–151

Akbani R, Kadir C, Akdemir B, et al (2015) Genomic Classification of Cutaneous Melanoma. Cell; 161(7):1681–1696.

Algazi AP, Tsai KK, Shoushtari AN, et al (2016) Clinical Outcomes in metastatic uveal melanoma treated with PD-1 and PD-L-1 antibodies. Cancer; 122: 3344-3353.

Babb J, Rogatko A, Shelemyahu Z et al, (1998) Cancer Phase I Clinical Trials: Efficient Dose Escalation with Overdose Control. Statist. Med; 17: 1103-1120.

Bender C, Enk A, Gutzmer R, et al (2017) Anti-PD-1 antibodies in metastatic uveal melanoma: a treatment option? Cancer Med; 6: 1581-1586

Buder K, Gesierch A, Gelbrich G, et al (2013) Systemic treatment of metastatic uveal melanoma: review of literature and future perspectives. Cancer Med; 2(5): 674-686.

Butler M, Sato T, Carvajal RD, et al (2021) Kinetics of radiographic response for tebentafusp (tebe) in previously treated metastatic uveal melanoma (MUM) patients (pts) achieving prolonged survival. AACR Annual meeting.

Carvajal R, Schwartz GK, Tezel T, et al (2017) Metatstatic disease from uveal melanoma: treatment options and future prospects. Ophthalmol; 101:38-44.

Kujala E, Makitie T, Kivela T, et al (2003) Very long-term prognosis of patients with malignant uveal melanoma. Invest Opthalmol Vis Sci; 44(11):4651-9

Livingstone E, Zaremba A, Horn S, et al (2020) GNAQ and GNA11 mutant non-uveal melanoma: a subtype distinct from both cutaneous and uveal melanoma. Br J Dermatol; 183 (5):928-939

Marincola FM, Venzon D, White D, et al (1992) HLA Association with Response and Toxicity in Melanoma Patients Treated with Interleukin 2-based Immunotherapy. Cancer Res; 52(23):6561-6.

Middleton MR, McAlpine C, Woodcock VK, et al (2020) Tebentafusp, A TCR/Anti-CD3 Bispecific Fusion Protein Targeting gp100, Potently Activated Antitumor Immune Responses in Patients with Metastatic Melanoma. Clin Cancer Res; 26(22):5869-5878

Namikawa K, Takahashi A, Mori T, et al (2020) Nivolumab for patients with metatstatic uveal melanoma previously untreated with ipilimumab: a single-institution retrospective study. Melanoma Res; 30: 76-84.

Nathan P, Hassel JC, Rutkowski P, et al (2021) Overall survival benefit with Tebentafusp in patients with Metastatic Uveal Melanoma. N Engl J Med; 385(13):1196-1206

Neuenschwander B, Branson M, Gsponer T, et al (2008) Critical aspects of the Bayesian approach to phase I cancer trials. Stat Med; 27: 2420-39.

Pelster M, Gruschkus SK, Bassett R, et al (2019) Phase II study if ipilimumab and nivolumab (ipi/nivo) in metastatic uveal melanoma (UM). J. Clin. Oncol; 37: 9522

Piperno-Neumann S, Diallo A, Etienne-Grimaldi MC, et al (2016) Phase II trial of Bevacizumab in Combination with Temozolide as First-Line Treatment in patient with Metastatic Uveal Melanoma. Oncologist; 21, 281–282

Piperno-Neumann S, Larkin J, Carvajal RD, et al (2020) Genomic Profiling if Metastatic Uveal Melanoma and Clinical Results of a Phase I study of the Protein Kinase C Inhibitor AEB017. Mol Cancer Ther; 19(4):1031-1039.

Piperno-Neumann S, Hassel J, Rutkowski P, et al (2021) Phase 3 randomized trial comparing tebentafusp with investigator's choice in first line metastatic uveal melanoma. Cancer Res; 81(13)

Rodriguez-Vidal C, Fernandez-Diaz D, Fernandez-Marta B, et al (2020) Treatment of Metastatic Uveal Melanoma: Systemic Review. Cancers (Basel); 12(9): 2557

Rogatko A (2007) Statistical Concepts and Applications in Clinical Medicine. John Aitchison, Jim W. Kay and Ian J. Lauder, Chapman Hall/CRC, Boca Raton, 2005. ISBN 1-58488-208-5." Statistics in Medicine 26.4. 943-44.

Rossi E, Pagliara MM, Orteschi D, et al (2019) Pembrolizumab as first-line treatment for metastatic uveal melanoma Cancer Immunol. Immunother; CII 2019, 68, 1179–1185

Rothermel LD, Sabesan AC, Stephens DJ, et al (2016) Identification of an Immunogenic Subset of Metastatic Uveal Melanoma. Clin Cancer Res; 22(9):2237-49

Rozengurt E (2007) Mitogenic signaling pathways induced by G protein-coupled receptors. Journal of Cellular Physiology; 10.1002/jcp.21246

Saber H, Leighton JK (2015) An FDA oncology analysis of antibody-drug conjugates. Regul Toxicol Pharmacol; 71(3):444-52.

Saber H, Simpson N, Ricks TK, et al (2019) An FDA oncology analysis of toxicities associated with PBD-containing antibody-drug conjugates. Regul Toxicol Pharmacol; 107:104429.

Schlegel JG, Tahoun M, Seidinger A, et al (2021) Macrocyclic Gq protein inhibitors FR900359 and/or YM-254890−fit for translation? ACS Pharmacol Transl Sci; 4:888-97.

Sheng X, Kong Y, Li Y, et al (2016) GNAQ and GNA11 mutations occurs in 9.5% of mucosal melanoma and are associated with poor prognosis. European Journal of Cancer; 65:156-163

Van Raamsdonk CD, Bezrookove V, Green G, et al (2009) Frequent somatic mutations of GNAQ in uveal melanoma and blue naevi. Nature; 457(7229):599-602.

Van Raamsdonk CD, Griewank KG, Crosby MB, et al (2010) Mutations in GNA11 in uveal melanoma. N Engl J Med; 363(23):2191-9.

Virgili G, Gatta G, Ciccolallo L, et al (2007) Incidence of uveal melanoma in Europe. Ophthalmology; 114:2309–15.

Wagner SN, Wagner C, Schultewolter T, et al (1997) Analysis of Pmel17/gp100 expression in primary human tissue specimens: implications for melanoma immuno- and gene-therapy. Cancer Immunol Immunother; 44(4):239-47.

Woodman SE (2012) Metastatic uveal melanoma biology and emergence treatments. Cancer J.; 18(2):148-152.

16 Appendices

16.1 Appendix 1: Guidelines for Response, Duration of Overall Response, TTF, TTP, Progression-Free Survival, and Overall Survival (based on RECIST 1.1)

16.1.1 Introduction

The purpose of this document is to provide the working definitions and rules necessary for a consistent and efficient analysis of efficacy for oncology studies in solid tumors. This document is based on the RECIST criteria for tumor responses (Therasse et al 2000) and the revised RECIST 1.1 guidelines (Eisenhauer et al 2009).

The efficacy assessments described in Section 16.1.2 and the definition of best response in Section 3.1 are based on the RECIST 1.1 criteria but also give more detailed instructions and rules for determination of best response. Section 16.1.12 is summarizing the "time to event" variables and rules which are mainly derived from internal discussions and regulatory consultations, as the RECIST criteria do not define these variables in detail. Section 16.1.14 of this guideline describes data handling and programming rules. This section is to be referred to in the SAP (Statistical Analysis Plan) to provide further details needed for programming.

16.1.2 Efficacy assessments

Tumor evaluations are made based on RECIST criteria (Therasse et al 2000), New Guidelines to Evaluate the Response to Treatment in Solid Tumors, Journal of National Cancer Institute, Vol. 92; 205-16 and revised RECIST guidelines (version 1.1) (Eisenhauer et al 2009) European Journal of Cancer; 45:228-247.

16.1.3 Definitions

16.1.3.1 Disease measurability

In order to evaluate tumors throughout a study, definitions of measurability are required in order to classify lesions appropriately at baseline. In defining measurability, a distinction also needs to be made between nodal lesions (pathological lymph nodes) and non-nodal lesions.

- **Measurable disease** - the presence of at least one measurable nodal or non-nodal lesion. If the measurable disease is restricted to a solitary lesion, its neoplastic nature should be confirmed by cytology/histology.

For patients without measurable disease see Section 16.1.12.8

Measurable lesions (both nodal and non-nodal)

- Measurable non-nodal - As a rule of thumb, the minimum size of a measurable non-nodal target lesion at baseline should be no less than double the slice thickness or 10mm whichever is greater - e.g. the minimum non-nodal lesion size for CT/MRI with 5mm cuts will be 10 mm, for 8 mm contiguous cuts the minimum size will be 16 mm.

- Lytic bone lesions or mixed lytic-blastic lesions with identifiable soft tissue components, that can be evaluated by CT/MRI, can be considered as measurable lesions, if the soft tissue component meets the definition of measurability.
- Measurable nodal lesions (i.e. lymph nodes) - Lymph nodes ≥15 mm in short axis can be considered for selection as target lesions. Lymph nodes measuring ≥10 mm and <15 mm are considered non-measurable. Lymph nodes smaller than 10 mm in short axis at baseline, regardless of the slice thickness, are normal and not considered indicative of disease.
- Cystic lesions:
  - Lesions that meet the criteria for radiographically defined simple cysts (i.e., spherical structure with a thin, non-irregular, non-nodular and non-enhancing wall, no septations, and low CT density [water-like] content) should not be considered as malignant lesions (neither measurable nor non-measurable) since they are, by definition, simple cysts.
  - ‘Cystic lesions’ thought to represent cystic metastases can be considered as measurable lesions, if they meet the definition of measurability described above. However, if noncystic lesions are present in the same patient, these are preferred for selection as target lesions.
- Non-measurable lesions - all other lesions are considered non-measurable, including small lesions (e.g. longest diameter <10 mm with CT/MRI or pathological lymph nodes with ≥ 10 to < 15 mm short axis), as well as truly non-measurable lesions e.g., blastic bone lesions, leptomeningeal disease, ascites, pleural/pericardial effusion, inflammatory breast disease, lymphangitis cutis/pulmonis, abdominal masses/abdominal organomegaly identified by physical exam that is not measurable by reproducible imaging techniques.

16.1.3.2 Eligibility based on measurable disease

If no measurable lesions are identified at baseline, the patient may be allowed to enter the study in some situations (e.g. in Phase III studies where PFS is the primary endpoint). However, it is recommended that patients be excluded from trials where the main focus is on the Overall Response Rate (ORR). Guidance on how patients with just non-measurable disease at baseline will be evaluated for response and also handled in the statistical analyses is given in Section 16.1.12.8.

16.1.4 Methods of tumor measurement - general guidelines

In this document, the term “contrast” refers to intravenous (i.v.) contrast.

The following considerations are to be made when evaluating the tumor:

- All measurements should be taken and recorded in metric notation (mm), using a ruler or calipers. All baseline evaluations should be performed as closely as possible to the beginning of treatment and never more than 4 weeks before the beginning of the treatment.
- Imaging-based evaluation is preferred to evaluation by clinical examination when both methods have been used to assess the antitumor effect of a treatment.

- For optimal evaluation of patients, the same methods of assessment and technique should be used to characterize each identified and reported lesion at baseline and during follow-up. Contrast-enhanced CT of chest, abdomen and pelvis should preferably be performed using a 5 mm slice thickness with a contiguous reconstruction algorithm. CT/MRI scan slice thickness should not exceed 8 mm cuts using a contiguous reconstruction algorithm. If, at baseline, a patient is known to have a medical contraindication to CT contrast or develops a contraindication during the trial, the following change in imaging modality will be accepted for follow up: a non-contrast CT of chest (MRI not recommended due to respiratory artifacts) plus contrast-enhanced MRI of abdomen and pelvis.
- A change in methodology can be defined as either a change in contrast use (e.g. keeping the same technique, like CT, but switching from with to without contrast use or vice-versa, regardless of the justification for the change) or a major change in technique (e.g. from CT to MRI, or vice-versa), or a change in any other imaging modality. A change from conventional to spiral CT or vice versa will not constitute a major “change in method” for the purposes of response assessment. A change in methodology will result by default in a UNK overall lesion response assessment as per Novartis calculated response. However, another response assessment than the Novartis calculated UNK response may be accepted from the investigator or the central blinded reviewer if a definitive response assessment can be justified, based on the available information.
- FDG-PET: can complement CT scans in assessing progression (particularly possible for ‘new’ disease). New lesions on the basis of FDG-PET imaging can be identified according to the following algorithm:
  - Negative FDG-PET at baseline, with a positive FDG-PET at follow-up is a sign of PD based on a new lesion.
  - No FDG-PET at baseline with a positive FDG-PET at follow-up:
- If new disease is indicated by a positive PET scan but is not confirmed by CT (or some other conventional technique such as MRI) at the same assessment, then follow-up assessments by CT will be needed to determine if there is truly progression occurring at that site. In all cases PD will be the date of confirmation of new disease by CT (or some other conventional technique such as MRI) rather than the date of the positive PET scan. If there is a positive PET scan without any confirmed progression at that site by CT, then a PD cannot be assigned.
  - If the positive FDG-PET at follow-up corresponds to a pre-existing site of disease on CT that is not progressing on the basis of the anatomic images, this is not PD.
  - Chest x-ray: Lesions on chest x-ray are acceptable as measurable lesions when they are clearly defined and surrounded by aerated lung. However, CT is preferable.
  - Physical exams: Evaluation of lesions by physical examination is accepted when lesions are superficial, with at least 10mm size, and can be assessed using calipers.
  - Ultrasound: When the primary endpoint of the study is objective response evaluation, ultrasound (US) should not be used to measure tumor lesions, unless pre-specified by the protocol. It is, however, a possible alternative to clinical measurements of superficial palpable lymph nodes, subcutaneous lesions and thyroid nodules. US

might also be useful to confirm the complete disappearance of superficial lesions usually assessed by clinical examination.

- Endoscopy and laparoscopy: The utilization of endoscopy and laparoscopy for objective tumor evaluation has not yet been fully and widely validated. Their uses in this specific context require sophisticated equipment and a high level of expertise that may only be available in some centers. Therefore, the utilization of such techniques for objective tumor response should be restricted to validation purposes in specialized centers. However, such techniques can be useful in confirming complete pathological response when biopsies are obtained.
- Tumor markers: Tumor markers alone cannot be used to assess response. However, some disease specific and more validated tumor markers (e.g. CA-125 for ovarian cancer, PSA for prostate cancer, alpha-FP, LDH and Beta-hCG for testicular cancer) can be integrated as non-target disease. If markers are initially above the upper normal limit they must normalize for a patient to be considered in complete clinical response when all lesions have disappeared.

- **Cytology and histology**: Cytology and histology can be used to differentiate between PR and CR in rare cases (i.e., after treatment to differentiate between residual benign lesions and residual malignant lesions in tumor types such as germ cell tumors). Cytologic confirmation of neoplastic nature of any effusion that appears or worsens during treatment is required when the measurable tumor has met the criteria for response or stable disease. Under such circumstances, the cytologic examination of the fluid collected will permit differentiation between response and stable disease (an effusion may be a side effect of the treatment) or progressive disease (if the neoplastic origin of the fluid is confirmed).
- **Clinical examination**: Clinical lesions will only be considered measurable when they are superficial (i.e., skin nodules and palpable lymph nodes). For the case of skin lesions, documentation by color photography, including a ruler to estimate the size of the lesion, is recommended.

16.1.5 Baseline documentation of target and non-target lesions

For the evaluation of lesions at baseline and throughout the study, the lesions are classified at baseline as either target or non-target lesions:

- Target lesions: All measurable lesions (nodal and non-nodal) up to a maximum of five lesions in total (and a maximum of two lesions per organ), representative of all involved organs should be identified as target lesions and recorded and measured at baseline. Target lesions should be selected on the basis of their size (lesions with the longest diameter) and their suitability for accurate repeated measurements (either by imaging techniques or clinically). Each target lesion must be uniquely and sequentially numbered on the CRF (even if it resides in the same organ).

Minimum target lesion size at baseline

- **Non-nodal target**: Non-nodal target lesions identified by methods for which slice thickness is not applicable (e.g. clinical examination, photography) should be at least 10 mm in longest diameter. See Section 16.1.3.1.

- **Nodal target**: See Section 16.1.3.1.

A sum of diameters (long axis for non-nodal lesions, short axis for nodal) for all target lesions will be calculated and reported as the baseline sum of diameters (SOD). The baseline sum of diameters will be used as reference by which to characterize the objective tumor response. Each target lesion identified at baseline must be followed at each subsequent evaluation and documented on eCRF.

- **Non-target lesions**: All other lesions are considered non-target lesions, i.e. lesions not fulfilling the criteria for target lesions at baseline. Presence or absence or worsening of non-target lesions should be assessed throughout the study; measurements of these lesions are not required. Multiple non-target lesions involved in the same organ can be assessed as a group and recorded as a single item (i.e. multiple liver metastases). Each non-target lesion identified at baseline must be followed at each subsequent evaluation and documented on eCRF.

16.1.6 Follow-up evaluation of target and non-target lesions

To assess tumor response, the sum of diameters for all target lesions will be calculated (at baseline and throughout the study). At each assessment response is evaluated first separately for the target (Table 16-1) and non-target lesions (Table 16-2) identified at baseline. These evaluations are then used to calculate the overall lesion response considering both the target and non-target lesions together (Table 16-3) as well as the presence or absence of new lesions.

16.1.6.1 Follow-up and recording of lesions

At each visit and for each lesion the actual date of the scan or procedure which was used for the evaluation of each specific lesion should be recorded. This applies to target and non-target lesions as well as new lesions that are detected. At the assessment visit all of the separate lesion evaluation data are examined by the investigator in order to derive the overall visit response. Therefore all such data applicable to a particular visit should be associated with the same assessment number.

16.1.6.2 Non-nodal lesions

Following treatment, lesions may have longest diameter measurements smaller than the image reconstruction interval. Lesions smaller than twice the reconstruction interval are subject to substantial “partial volume” effects (i.e., size may be underestimated because of the distance of the cut from the longest diameter; such lesions may appear to have responded or progressed on subsequent examinations, when, in fact, they remain the same size).

If the lesion has completely disappeared, the lesion size should be reported as 0 mm.

Measurements of non-nodal target lesions that become 5 mm or less in longest diameter are likely to be non-reproducible. Therefore, it is recommended to report a default value of 5 mm, instead of the actual measurement. This default value is derived from the 5 mm CT slice thickness (but should not be changed with varying CT slice thickness). Actual measurement should be given for all lesions larger than 5 mm in longest diameter irrespective of slice thickness/reconstruction interval.

In other cases where the lesion cannot be reliably measured for reasons other than its size (e.g., borders of the lesion are confounded by neighboring anatomical structures), no measurement should be entered and the lesion cannot be evaluated.

16.1.6.3 Nodal lesions

A nodal lesion less than 10 mm in size by short axis is considered normal. Lymph nodes are not expected to disappear completely, so a "non-zero size" will always persist.

Measurements of nodal target lesions that become 5 mm or less in short axis are likely to be non-reproducible. Therefore, it is recommended to report a default value of 5 mm, instead of the actual measurement. This default value is derived from the 5 mm CT slice thickness (but should not be changed with varying CT slice thickness).Actual measurement should be given for all lesions larger than 5 mm in short axis irrespective of slice thickness/reconstruction interval.

However, once a target nodal lesion shrinks to less than 10 mm in its short axis, it will be considered normal for response purpose determination. The lymph node measurements will continue to be recorded to allow the values to be included in the sum of diameters for target lesions, which may be required subsequently for response determination.

16.1.7 Determination of target lesion response

Table 16-1 Response criteria for target lesions

Response Criteria	Evaluation of target lesions
Complete Response (CR):	Disappearance of all non-nodal target lesions. In addition, any pathological lymph nodes assigned as target lesions must have a reduction in short axis to < 10 mm [1]
Partial Response (PR):	At least a 30% decrease in the sum of diameter of all target lesions, taking as reference the baseline sum of diameters.
Progressive Disease (PD):	At least a 20% increase in the sum of diameter of all measured target lesions, taking as reference the smallest sum of diameter of all target lesions recorded at or after baseline. In addition to the relative increase of 20%, the sum must also demonstrate an absolute increase of at least 5 mm [2].
Stable Disease (SD):	Neither sufficient shrinkage to qualify for PR or CR nor an increase in lesions which would qualify for PD.
Unknown (UNK)	Progression has not been documented and one or more target lesions have not been assessed or have been assessed using a different method than baseline.[3]

1. SOD for CR may not be zero when nodal lesions are part of target lesions
2. Following an initial CR, a PD cannot be assigned if all non-nodal target lesions are still not present and all nodal lesions are <10 mm in size. In this case, the target lesion response is CR
3. In exceptional circumstances an UNK response due to change in method could be over-ruled by the investigator or central reviewer using expert judgment based on the available information (see Notes on target lesion response and methodology change in Section 16.1.4).

Notes on target lesion response

Reappearance of lesions: If the lesion appears at the same anatomical location where a target lesion had previously disappeared, it is advised that the time point of lesion disappearance (i.e., the "0 mm" recording) be re-evaluated to make sure that the lesion was not actually present and/or not visualized for technical reasons in this previous assessment. If it is not possible to change the 0 value, then the investigator/radiologist has to decide between the following possibilities:

- The lesion is a new lesion, in which case the overall tumor assessment will be considered as progressive disease
- The lesion is clearly a reappearance of a previously disappeared lesion, in which case the size of the lesion has to be entered in the CRF and the tumor assessment will remain based on the sum of tumor measurements as presented in Table 16-1 above (i.e., a PD will be determined if there is at least 20% increase in the sum of diameters of **all** measured target lesions, taking as reference the smallest sum of diameters of all target lesions recorded at or after baseline with at least 5 mm increase in the absolute sum of the diameters). Proper documentation should be available to support this decision. This applies to patients who have not achieved target response of CR. For patients who have achieved CR, please refer to last bullet in this section.
- For those patients who have only one target lesion at baseline, the reappearance of the target lesion which disappeared previously, even if still small, is considered a PD.
- Missing measurements: In cases where measurements are missing for one or more target lesions it is sometimes still possible to assign PD based on the measurements of the remaining lesions. For example, if the sum of diameters for 5 target lesions at baseline is 100 mm at baseline and the sum of diameters for 3 of those lesions at a post-baseline visit is 140 mm (with data for 2 other lesions missing) then a PD should be assigned. However, in other cases where a PD cannot definitely be attributed, the target lesion response would be UNK.
- Nodal lesion decrease to normal size: When nodal disease is included in the sum of target lesions and the nodes decrease to "normal" size they should still have a measurement recorded on scans. This measurement should be reported even when the nodes are normal in order not to overstate progression should it be based on increase in the size of nodes.
- Lesions split: In some circumstances, disease that is measurable as a target lesion at baseline and appears to be one mass can split to become two or more smaller sub-lesions. When this occurs, the diameters (long axis - non-nodal lesion, short axis - nodal lesions) of the two split lesions should be added together and the sum recorded in the diameter field on the case report form under the original lesion number. This value will be included in the sum of diameters when deriving target lesion response. The individual split lesions will not be considered as new lesions, and will not automatically trigger a PD designation.
- Lesions coalesced: Conversely, it is also possible that two or more lesions which were distinctly separate at baseline become confluent at subsequent visits. When this occurs a plane between the original lesions may be maintained that would aid in obtaining diameter measurements of each individual lesion. If the lesions have truly coalesced such that they are no longer separable, the maximal diameters (long axis - non-nodal lesion, short axis -

nodal lesions) of the “merged lesion” should be used when calculating the sum of diameters for target lesions. On the case report form, the diameter of the “merged lesion” should be recorded for the size of one of the original lesions while a size of “0”mm should be entered for the remaining lesion numbers which have coalesced.

- The **measurements for nodal lesions**, even if less than 10 mm in size, will contribute to the calculation of target lesion response in the usual way with slight modifications.
- Since lesions less than 10 mm are considered normal, a CR for target lesion response should be assigned when all nodal target lesions shrink to less than 10 mm and all non-nodal target lesions have disappeared.
- Once a CR target lesion response has been assigned a CR will continue to be appropriate (in the absence of missing data) until progression of target lesions.
- Following a CR, a PD can subsequently only be assigned for target lesion response if either a non-nodal target lesion “reappears” or if any single nodal lesion is at least 10 mm and there is at least 20% increase in sum of the diameters of all nodal target lesions relative to nadir with at least 5 mm increase in the absolute sum of the diameters.
- A change in method for the evaluation of one or more lesions will usually lead to an UNK target lesion response unless there is progression indicated by the remaining lesions which have been evaluated by the same method. In exceptional circumstances an investigator or central reviewer might over-rule this assignment to put a non-UNK response using expert judgment based on the available information. E.g. a change to a more sensitive method might indicate some tumor shrinkage of target lesions and definitely rule out progression in which case the investigator might assign an SD target lesion response; however, this should be done with caution and conservatively as the response categories have well defined criteria.

16.1.8 Determination of non-target lesion response

Table 16-2 Response criteria for non-target lesions

Response Criteria	Evaluation of non-target lesions
Complete Response (CR):	Disappearance of all non-target lesions. In addition, all lymph nodes assigned a non-target lesions must be non-pathological in size (< 10 mm short axis)
Progressive Disease (PD):	Unequivocal progression of existing non-target lesions.[1]
Non-CR/Non-PD:	Neither CR nor PD
Unknown (UNK)	Progression has not been documented and one or more non-target lesions have not been assessed or have been assessed using a different method than baseline[2.]

1. The assignment of PD solely based on change in non-target lesions in light of target lesion response of CR, PR or SD should be exceptional. In such circumstances, the opinion of the investigator or central reviewer does prevail..
2. It is recommended that the investigator and/or central reviewer should use expert judgment to assign a Non-UNK response wherever possible (see notes section for more details)

Notes on non-target lesion response

- The investigator and/or central reviewer can use expert judgment to assign a non-UNK response wherever possible, even where lesions have not been fully assessed or a different method has been used. In many of these situations it may still be possible to identify equivocal progression (PD) or definitively rule this out (non-CR/Non-PD) based on the available information. In the specific case where a more sensitive method has been used indicating the absence of any non-target lesions, a CR response can also be assigned.
- The response for non-target lesions is **CR** only if all non-target non-nodal lesions which were evaluated at baseline are now all absent and with all non-target nodal lesions returned to normal size (i.e. < 10 mm). If any of the non-target lesions are still present, or there are any abnormal nodal lesions (i.e. ≥ 10 mm) the response can only be '**Non-CR/Non-PD**' unless there is unequivocal progression of the non-target lesions (in which case response is **PD**) or it is not possible to determine whether there is unequivocal progression (in which case response is UNK).
- Unequivocal progression: To achieve "unequivocal progression" on the basis of non-target disease there must be an overall level of substantial worsening in non-target disease such that, even in presence of CR, PR or SD in target disease, the overall tumor burden has increased sufficiently to merit discontinuation of therapy. A modest "increase" in the size of one or more non-target lesions is usually not sufficient to qualify for unequivocal progression status. The designation of overall progression solely on the basis of change in non-target disease in the face of CR, PR or SD of target disease is therefore expected to be rare. In order for a PD to be assigned on the basis of non-target lesions, the increase in the extent of the disease must be substantial even in cases where there is no measurable disease at baseline. If there is unequivocal progression of non-target lesion(s), then at least one of the non-target lesions must be assigned a status of "Worsened". Where possible, similar rules to those described in Section 16.1.7 for assigning PD following a CR for the non-target lesion response in the presence of non-target lesions nodal lesions should be applied.

16.1.9 New lesions

The appearance of a new lesion is always associated with Progressive Disease (PD) and has to be recorded as a new lesion in the New Lesion CRF page.

- If a new lesion is **equivocal**, for example because of its small size, continued therapy and follow-up evaluation will clarify if it represents truly new disease. If repeat scans confirm there is definitely a new lesion, then progression should be declared using the date of the first observation of the lesion
- If new disease is observed in a region which was **not scanned at baseline** or where the particular baseline scan is not available for some reason, then this should be considered as a PD. The one exception to this is when there are no baseline scans at all available for a patient in which case the response should be UNK, as for any of this patient's assessment (see Section 16.1.10).
- A **lymph node is considered as a "new lesion"** and, therefore, indicative of progressive disease if the short axis increases in size to ≥ 10 mm for the first time in the study plus

5 mm absolute increase.

FDG-PET: can complement CT scans in assessing progression (particularly possible for 'new' disease). See Section 16.1.4.

16.1.10 Evaluation of overall lesion response

The evaluation of overall lesion response at each assessment is a composite of the target lesion response, non-target lesion response and presence of new lesions as shown below in Table 16- 3.

Table 16-3 Overall lesion response at each assessment

Target lesions	Non-target lesions	New Lesions	Overall lesion response
CR	CR	No	CR[1]
CR	Non-CR/Non-PD[3]	No	PR
CR, PR, SD	UNK	No	UNK
PR	Non-PD and not UNK	No	PR[1]
SD	Non-PD and not UNK	No	SD[1, 2]
UNK	Non-PD or UNK	No	UNK[1]
PD	Any	Yes or No	PD
Any	PD	Yes or No	PD
Any	Any	Yes	PD

1. This overall lesion response also applies when there are no non-target lesions identified at baseline.
2. Once confirmed PR was achieved, all these assessments are considered PR.
3. As defined in Section 16.1.6.

If there are no baseline scans available at all, then the overall lesion response at each assessment should be considered Unknown (UNK).

In some circumstances it may be difficult to distinguish residual disease from normal tissue. When the evaluation of complete response depends on this determination, it is recommended that the residual lesion be investigated (fine needle aspirate/biopsy) to confirm the CR.

16.1.11 Efficacy definitions

The following definitions primarily relate to patients who have measurable disease at baseline. Section 16.1.12.8 outlines the special considerations that need to be given to patients with no measurable disease at baseline in order to apply the same concepts.

16.1.11.1 Best overall response

The best overall response is the best response recorded from the start of the treatment until disease progression/recurrence (taking as reference for PD the smallest measurements recorded since the treatment started). In general, the patient's best response assignment will depend on the achievement of both measurement and confirmation criteria.

The best overall response will usually be determined from response assessments undertaken while on treatment. However, if any assessments occur after treatment withdrawal the protocol

should specifically describe if these will be included in the determination of best overall response and/or whether these additional assessments will be required for sensitivity or supportive analyses. As a default, any assessments taken more than 30 days after the last dose of study treatment will not be included in the best overall response derivation. If any alternative cancer therapy is taken while on study any subsequent assessments would ordinarily be excluded from the best overall response determination. If response assessments taken after withdrawal from study treatment and/or alternative therapy are to be included in the main endpoint determination, then this should be described and justified in the protocol.

Where a study requires confirmation of response (PR or CR), changes in tumor measurements must be confirmed by repeat assessments that should be performed not less than 4 weeks after the criteria for response are first met.

Longer intervals may also be appropriate. However, this must be clearly stated in the protocol. The main goal of confirmation of objective response is to avoid overestimating the response rate observed. In cases where confirmation of response is not feasible, it should be made clear when reporting the outcome of such studies that the responses are not confirmed.

- For non-randomized trials where response is the primary endpoint, confirmation is needed.
- For trials intended to support accelerated approval, confirmation is needed
- For all other trials, confirmation of response may be considered optional.

The best overall response for each patient is determined from the sequence of overall (lesion) responses according to the following rules:

- CR = at least two determinations of CR at least 4 weeks apart before progression where confirmation required or one determination of CR prior to progression where confirmation not required
- PR = at least two determinations of PR or better at least 4 weeks apart before progression (and not qualifying for a CR) where confirmation required or one determination of PR prior to progression where confirmation not required
- SD = at least one SD assessment (or better) > 6 weeks after randomization/start of treatment (and not qualifying for CR or PR).
- PD = progression ≤ 12 weeks after randomization/ start of treatment (and not qualifying for CR, PR or SD).
- UNK = all other cases (i.e. not qualifying for confirmed CR or PR and without SD after more than 6 weeks or early progression within the first 12 weeks)

The time durations specified in the SD/PD/UNK definitions above are defaults based on a 6 week tumor assessment frequency. However these may be modified for specific indications which are more or less aggressive. In addition, it is envisaged that the time duration may also take into account assessment windows. E.g. if the assessment occurs every 6 weeks with a time window of +/- 7 days, a BOR of SD would require a SD or better response longer than 5 weeks after randomization/start of treatment.

Overall lesion responses of CR must stay the same until progression sets in, with the exception of a UNK status. A patient who had a CR cannot subsequently have a lower status other than a PD, e.g. PR or SD, as this would imply a progression based on one or more lesions reappearing, in which case the status would become a PD.

Once an overall lesion response of PR is observed (which may have to be a confirmed PR depending on the study) this assignment must stay the same or improve over time until progression sets in, with the exception of an UNK status. However, in studies where confirmation of response is required, if a patient has a single PR (≥30% reduction of tumor burden compared to baseline) at one assessment, followed by a <30% reduction from baseline at the next assessment (but not ≥20% increase from previous smallest sum), the objective status at that assessment should be SD. Once a confirmed PR was seen, the overall lesion response should be considered PR (or UNK) until progression is documented or the lesions totally disappear in which case a CR assignment is applicable. In studies where confirmation of response is not required after a single PR the overall lesion response should still be considered PR (or UNK) until progression is documented or the lesion totally disappears in which case a CR assignment is applicable.

Example: In a case where confirmation of response is required the sum of lesion diameters is 200 mm at baseline and then 140 mm - 150 mm - 140 mm - 160 mm - 160 mm at the subsequent visits. Assuming that non-target lesions did not progress, the overall lesion response would be PR - SD - PR - PR - PR. The second assessment with 140 mm confirms the PR for this patient. All subsequent assessments are considered PR even if tumor measurements decrease only by 20% compared to baseline (200 mm to 160 mm) at the following assessments.

If the patient progressed but continues study treatment, further assessments are not considered for the determination of best overall response.

Note: these cases may be described as a separate finding in the CSR but not included in the overall response or disease control rates.

The best overall response for a patient is always calculated, based on the sequence of overall lesion responses. However, the overall lesion response at a given assessment may be provided from different sources:

- Investigator overall lesion response
- Central Blinded Review overall lesion response
- Novartis calculated overall lesion response (based on measurements from either Investigator or Central Review)

The primary analysis of the best overall response will be based on the sequence of investigator/central blinded review/calculated (investigator)/calculated (central) overall lesion responses.

Based on the patients' best overall response during the study, the following rates are then calculated:

Overall response rate (ORR) is the proportion of patients with a best overall response of CR or PR. This is also referred to as 'Objective response rate' in some protocols or publications.

Disease control rate (DCR) is the proportion of patients with a best overall response of CR or PR or SD. The objective of this endpoint is to summarize patients with signs of "activity" defined as either shrinkage of tumor (regardless of duration) or slowing down of tumor growth.

Clinical benefit rate (CBR) is the proportion of patients with a best overall response of CR or PR , or an overall lesion response of SD or Non-CR/Non-PD which lasts for a minimum time duration (with a default of at least 24 weeks in breast cancer studies). This endpoint measures signs of activity taking into account duration of disease stabilization.

Another approach is to summarize the progression rate at a certain time point after baseline. In this case, the following definition is used:

Early progression rate (EPR) is the proportion of patients with progressive disease within 8 weeks of the start of treatment.

The protocol should define populations for which these will be calculated. The time point for EPR is study specific. EPR is used for the multinomial designs of (Dent et al 2001) and counts all patients who at the specified assessment (in this example the assessment would be at 8 weeks ± window) do not have an overall lesion response of SD, PR or CR. Patients with an unknown (UNK) assessment at that time point and no PD before, will not be counted as early progressors in the analysis but may be included in the denominator of the EPR rate, depending on the analysis population used. Similarly when examining overall response and disease control, patients with a best overall response assessment of unknown (UNK) will not be regarded as "responders" but may be included in the denominator for ORR and DCR calculation depending on the analysis population (e.g. populations based on an ITT approach).

16.1.12 Time to event variables

The protocol should state which of the following variables is used in that study.

16.1.12.1 Progression-free survival

Usually in all Oncology studies, patients are followed for tumor progression after discontinuation of study medication for reasons other than progression or death. If this is not used, e.g. in Phase I or II studies, this should be clearly stated in the protocol. Note that randomized trials (preferably blinded) are recommended where PFS is to be the primary endpoint.

Progression-free survival (PFS) is the time from date of randomization/start of treatment to the date of event defined as the first documented progression or death due to any cause. If a patient has not had an event, progression-free survival is censored at the date of last adequate tumor assessment.

PFS rate at x weeks is an additional measure used to quantify PFS endpoint. It is recommended that a Kaplan Meier estimate is used to assess this endpoint.

16.1.12.2 Overall survival

All patients should be followed until death or until patient has had adequate follow-up time as specified in the protocol whichever comes first. The follow-up data should contain the date the patient was last seen alive / last known date patient alive, the date of death and the reason of death ("Study indication" or "Other").

Overall survival (OS) is defined as the time from date of randomization/start of treatment to date of death due to any cause. If a patient is not known to have died, survival will be censored at the date of last known date patient alive.

16.1.12.3 Time to progression

Some studies might consider only death related to underlying cancer as an event which indicates progression. In this case the variable "Time to progression" might be used. TTP is defined as PFS except for death unrelated to underlying cancer.

Time to progression (TTP) is the time from date of randomization/start of treatment to the date of event defined as the first documented progression or death due to underlying cancer. If a patient has not had an event, time to progression is censored at the date of last adequate tumor assessment.

16.1.12.4 PFS2

A recent EMA guidance (EMA 2017) recommends a substitute end point intermediate to PFS and OS called PFS2, a surrogate for OS when OS cannot be measured reliably, which assesses the impact of the experimental therapy on next-line treatment. The main purpose of this endpoint is to assess long term maintenance strategies, particularly of resensitizing agents and where it is necessary to examine the overall "field of influence".

PFS2, which could be termed PFS deferred, PFS delayed, tandem PFS, or PFS version 2.0, is the time from date of randomization/start of treatment to the date of event defined as the first documented progression on next-line treatment or death from any cause. The censoring rules for this endpoint will incorporate the same principles as those considered for PFS in this document, and in addition may involve other considerations which will need to be detailed in the protocol.

Please note that data collection for the PFS2 is limited to the date of progression and not specific read of the tumor assessments.

It is strongly recommended that the teams consult regulatory agencies for scientific advice given the limited experience with the use of this endpoint in regulatory setting in light of methodological issues w.r.t. censoring foreseen.

16.1.12.5 Time to treatment failure

This endpoint is often appropriate in studies of advanced disease where early discontinuation is typically related to intolerance of the study drug. In some protocols, time to treatment failure may be considered as a sensitivity analysis for time to progression. The list of discontinuation reasons to be considered or not as treatment failure may be adapted according to the specificities of the study or the disease.

Time to treatment failure (TTF) is the time from date of randomization/start of treatment to the earliest of date of progression, date of death due to any cause, or date of discontinuation due to reasons other than ‘Protocol violation’ or ‘Administrative problems’. The time to treatment failure for patients who did not experience treatment failure will be censored at last adequate tumor assessment.

16.1.12.6 Duration of response

The analysis of the following variables should be performed with much caution when restricted to responders since treatment bias could have been introduced. There have been reports where a treatment with a significantly higher response rate had a significantly shorter duration of response but where this probably primarily reflected selection bias which is explained as follows: It is postulated that there are two groups of patients: a good risk group and a poor risk group. Good risk patients tend to get into response readily (and relatively quickly) and tend to remain in response after they have a response. Poor risk patients tend to be difficult to achieve a response, may have a longer time to respond, and tend to relapse quickly when they do respond. Potent agents induce a response in both good risk and poor risk patients. Less potent agents induce a response mainly in good risk patients only. This is described in more detail by (Morgan 1988)

It is recommended that an analysis of all patients (both responders and non-responders) be performed whether or not a “responders only” descriptive analysis is presented. An analysis of responders should only be performed to provide descriptive statistics and even then interpreted with caution by evaluating the results in the context of the observed response rates... If an inferential comparison between treatments is required this should only be performed on all patients (i.e. not restricting to “responders” only) using appropriate statistical methods such as the techniques described in (Ellis et al 2008). It should also be stated in the protocol if duration of response is to be calculated in addition for unconfirmed response.

For summary statistics on “responders” only the following definitions are appropriate. (Specific definitions for an all-patient analysis of these endpoints are not appropriate since the status of patients throughout the study is usually taken into account in the analysis).

Duration of overall response (CR or PR): For patients with a CR or PR (which may have to be confirmed the start date is the date of first documented response (CR or PR) and the end date and censoring is defined the same as that for time to progression.

The following two durations might be calculated in addition for a large Phase III study in which a reasonable number of responders is seen.

Duration of overall complete response (CR): For patients with a CR (which may have to be confirmed) the start date is the date of first documented CR and the end date and censoring is defined the same as that for time to progression.

Duration of stable disease (CR/PR/SD): For patients with a CR or PR (which may have to be confirmed) or SD the start and end date as well as censoring is defined the same as that for time to progression.

16.1.12.7 Time to response

Time to overall response (CR or PR) is the time between date of randomization/start of treatment until first documented response (CR or PR). The response may need to be confirmed depending on the type of study and its importance. Where the response needs to be confirmed then time to response is the time to the first CR or PR observed.

Although an analysis on the full population is preferred a descriptive analysis may be performed on the "responders" subset only, in which case the results should be interpreted with caution and in the context of the overall response rates, since the same kind of selection bias may be introduced as described for duration of response in Section 16.1.12.5. It is recommended that an analysis of all patients (both responders and non-responders) be performed whether or not a "responders only" descriptive analysis is presented. Where an inferential statistical comparison is required, then all patients should definitely be included in the analysis to ensure the statistical test is valid. For analysis including all patients, patients who did not achieve a response (which may have to be a confirmed response) will be censored using one of the following options.

- at maximum follow-up (i.e. FPFV to LPLV used for the analysis) for patients who had a PFS event (i.e. progressed or died due to any cause). In this case the PFS event is the worst possible outcome as it means the patient cannot subsequently respond. Since the statistical analysis usually makes use of the ranking of times to response it is sufficient to assign the worst possible censoring time which could be observed in the study which is equal to the maximum follow-up time (i.e. time from FPFV to LPLV)
- at last adequate tumor assessment date otherwise. In this case patients have not yet progressed so they theoretically still have a chance of responding

Time to overall complete response (CR) is the time between dates of randomization/start of treatment until first documented CR. Similar analysis considerations including (if appropriate) censoring rules apply for this endpoint described for the time to overall response endpoint.

16.1.12.8 Definition of start and end dates for time to event variables

Assessment date

For each assessment (i.e. evaluation number), the **assessment date** is calculated as the latest of all measurement dates (e.g. X-ray, CT-scan) if the overall lesion response at that assessment is CR/PR/SD/UNK. Otherwise - if overall lesion response is progression - the assessment date is calculated as the earliest date of all measurement dates at that evaluation number.

In the calculation of the assessment date for time to event variables, any unscheduled assessment should be treated similarly to other evaluations.

Start dates

For all "time to event" variables, other than duration of response, the randomization/ date of treatment start will be used as the start date.

For the calculation of duration of response the following start date should be used:

- Date of first documented response is the assessment date of the first overall lesion response of CR (for duration of overall complete response) or CR / PR (for duration of overall response) respectively, when this status is later confirmed.

End dates

The end dates which are used to calculate 'time to event' variables are defined as follows:

- Date of death (during treatment as recorded on the treatment completion page or during follow-up as recorded on the study evaluation completion page or the survival follow-up page).
- Date of progression is the first assessment date at which the overall lesion response was recorded as progressive disease.
- Date of last adequate tumor assessment is the date the last tumor assessment with overall lesion response of CR, PR or SD which was made before an event or a censoring reason occurred. In this case the last tumor evaluation date at that assessment is used. If no post-baseline assessments are available (before an event or a censoring reason occurred) the date of randomization/start of treatment is used.
- Date of next scheduled assessment is the date of the last adequate tumor assessment plus the protocol specified time interval for assessments. This date may be used if back-dating is considered when the event occurred beyond the acceptable time window for the next tumor assessment as per protocol (see Section 16.1.12.8).

Example (if protocol defined schedule of assessments is 3 months): tumor assessments at baseline - 3 months - 6 months - missing - missing - PD. Date of next scheduled assessment would then correspond to 9 months.

- Date of discontinuation is the date of the end of treatment visit.
- Date of last contact is defined as the last date the patient was known to be alive. This corresponds to the latest date for either the visit date, lab sample date or tumor assessment date. If available, the last known date patient alive from the survival follow-up page is used. If no survival follow-up is available, the date of discontinuation is used as last contact date.
- Date of secondary anti-cancer therapy is defined as the start date of any additional (secondary) antineoplastic therapy or surgery.

16.1.12.9 Handling of patients with non-measurable disease only at baseline

It is possible that patients with only non-measurable disease present at baseline are entered into the study, either because of a protocol violation or by design (e.g. in Phase III studies with PFS as the primary endpoint). In such cases the handling of the response data requires special consideration with respect to inclusion in any analysis of endpoints based on the overall response evaluations.

It is recommended that any patients with only non-measurable disease at baseline should be included in the main (ITT) analysis of each of these endpoints.

Although the text of the definitions described in the previous sections primarily relates to patients with measurable disease at baseline, patients without measurable disease should also be incorporated in an appropriate manner. The overall response for patients with non-measurable disease is derived slightly differently according to Table 16-4.

Table 16-4 Overall lesion response at each assessment: patients with non-target disease only

Non-target lesions	New Lesions	Overall lesion response
CR	No	CR
Non-CR/Non-PD[1]	No	Non-CR/non-PD
UNK	No	UNK
PD	Yes or No	PD
Any	Yes	PD

[1] As defined in Section 16.1.6.

In general, the **non-CR/non-PD response** for these patients is considered equivalent to an SD response in endpoint determination. In summary tables for best overall response patients with only non-measurable disease may be highlighted in an appropriate fashion e.g. in particular by displaying the specific numbers with the non-CR/non-PD category.

In considering how to incorporate data from these patients into the analysis the importance to each endpoint of being able to identify a PR and/or to determine the occurrence and timing of progression needs to be taken into account.

For ORR it is recommended that the main (ITT) analysis includes data from patients with only non-measurable disease at baseline, handling patients with a best response of CR as "responders" with respect to ORR and all other patients as "non-responders".

For PFS, it is again recommended that the main ITT analyses on these endpoints include all patients with only non-measurable disease at baseline, with possible sensitivity analyses which exclude these particular patients. Endpoints such as PFS which are reliant on the determination and/or timing of progression can incorporate data from patients with only non-measurable disease.

16.1.12.10 Sensitivity analyses

This section outlines the possible event and censoring dates for progression, as well as addresses the issues of missing tumor assessments during the study. For instance, if one or more assessment visits are missed prior to the progression event, to what date should the progression event be assigned? And should progression event be ignored if it occurred after a long period of a patient being lost to follow-up? It is important that the protocol and RAP specify the primary analysis in detail with respect to the definition of event and censoring dates and also include a description of one or more sensitivity analyses to be performed.

Based on definitions outlined in Section 16.1.12.7, and using the draft FDA guidelines on endpoints (FDA 2005, FDA 2007) as a reference, the following analyses can be considered:

Table 16-5 Options for event dates used in PFS, TTP, duration of response

**Situation**		**Options for end-date (progression or censoring)**[1]<br>**(1) = default unless specified differently in the protocol or RAP**	**Outcome**
A	No baseline assessment	(1) Date of randomization/start of treatment[3]	Censored
B	Progression at or before next scheduled assessment	(1) Date of progression<br>(2) Date of next scheduled assessment[2]	Progressed<br>Progressed
C1	Progression or death after **exactly one** missing assessment	(1) Date of progression (or death)<br>(2) Date of next scheduled assessment[2]	Progressed<br>Progressed
C2	Progression or death after **two or more** missing assessments	(1) Date of last adequate assessment[2]<br>(2) Date of next scheduled assessment[2]<br>(3) Date of progression (or death)	Censored<br>Progressed<br>Progressed
D	No progression	(1) Date of last adequate assessment	Censored
E	Treatment discontinuation due to 'Disease progression' without documented progression, i.e. clinical progression based on investigator claim	(1) Ignore clinical progression and follow situations above<br>(2) Date of discontinuation (visit date at which clinical progression was determined)	As per above situations<br>Progressed
F	New anticancer therapy given	Ignore the new anticancer therapy and follow situations above (ITT approach)<br>Date of last adequate assessment prior to new anticancer therapy<br>(3) Date of secondary anti-cancer therapy<br>(4) Date of secondary anti-cancer therapy	As per above situations<br>Censored<br>Censored<br>Event
G	Deaths due to reason other than deterioration of 'Study indication'	(1) Date of last adequate assessment	Censored (only TTP and duration of response)

1. =Definitions can be found in Section 16.1.12.7.
2. =After the last adequate tumor assessment. "Date of next scheduled assessment" is defined in Section 16.1.12.7.
3. =The rare exception to this is if the patient dies no later than the time of the second scheduled assessment as defined in the protocol in which case this is a PFS event at the date of death.

The primary analysis and the sensitivity analyses must be specified in the protocol. Clearly define if and why options (1) are not used for situations C, E and (if applicable) F.

Situations C (C1 and C2): Progression or death after one or more missing assessments: The primary analysis is usually using options (1) for situations C1 and C2, i.e.

- (C1) taking the actual progression or death date, in the case of only one missing assessment.
- (C2) censoring at the date of the last adequate assessment, in the case of two or more consecutive missing assessments.

In the case of two or missing assessments (situation C2), option (3) may be considered jointly with option (1) in situation C1 as sensitivity analysis. A variant of this sensitivity analysis consists of backdating the date of event to the next scheduled assessment as proposed with option (2) in situations C1 and C2.

Situation E: Treatment discontinuation due to 'Disease progression' without documented progression: By default, option (1) is used for situation E as patients without documented PD should be followed for progression after discontinuation of treatment. However, option (2) may be used as sensitivity analysis. If progression is claimed based on clinical deterioration instead of tumor assessment by e.g. CT-scan, option (2) may be used for indications with high early progression rate or difficulties to assess the tumor due to clinical deterioration.

Situation F: New cancer therapy given: the handling of this situation must be specified in detail in the protocol. However, option (1) (ITT) is the recommended approach; events documented after the initiation of new cancer therapy will be considered for the primary analysis i.e. progressions and deaths documented after the initiation of new cancer therapy would be included as events. This will require continued follow-up for progression after the start of the new cancer therapy. In such cases, it is recommended that an additional sensitivity analysis be performed by censoring at last adequate assessment prior to initiation of new cancer therapy.

Option (2), i.e. censoring at last adequate assessment may be used as a sensitivity analysis. If a high censoring rate due to start of new cancer therapy is expected, a window of approximately 8 weeks performed after the start of new cancer therapy can be used to calculate the date of the event or censoring. This should be clearly specified in the analysis plan.

In some specific settings, local treatments (e.g. radiation/surgery) may not be considered as cancer therapies for assessment of event/censoring in PFS/TTP/DoR analysis. For example, palliative radiotherapy given in the trial for analgesic purposes or for lytic lesions at risk of fracture will not be considered as cancer therapy for the assessment of BOR and PFS analyses. The protocol should clearly state the local treatments which are not considered as antineoplastic therapies in the PFS/TTP/DoR analysis.

The protocol should state that tumor assessments will be performed every x weeks until radiological progression irrespective of initiation of new antineoplastic therapy. It is strongly recommended that a tumor assessment is performed before the patient is switched to a new cancer therapy.

Additional suggestions for sensitivity analyses

Other suggestions for additional sensitivity analyses may include analyses to check for potential bias in follow-up schedules for tumor assessments, e.g. by assigning the dates for censoring and events only at scheduled visit dates. The latter could be handled by replacing in Table 16-2 the "Date of last adequate assessment" by the "Date of previous scheduled assessment (from baseline)", with the following definition:

- **Date of previous scheduled assessment (from baseline)** is the date when a tumor assessment would have taken place, if the protocol assessment scheme was strictly followed from baseline, immediately before or on the date of the last adequate tumor assessment.

In addition, analyses could be repeated using the Investigators' assessments of response rather than the calculated response. The need for these types of sensitivity analyses will depend on the individual requirements for the specific study and disease area and have to be specified in the protocol or RAP documentation.

16.1.13 Data handling and programming rules

The following section should be used as guidance for development of the protocol, data handling procedures or programming requirements (e.g. on incomplete dates).

16.1.13.1 Study/project specific decisions

For each study (or project) various issues need to be addressed and specified in the protocol or RAP documentation. Any deviations from protocol must be discussed and defined at the latest in the RAP documentation.

The proposed primary analysis and potential sensitivity analyses should be discussed and agreed with the health authorities and documented in the protocol (or at the latest in the RAP documentation before database lock).

16.1.13.2 End of treatment phase completion

Patients **may** voluntarily withdraw from the study treatment or may be taken off the study treatment at the discretion of the investigator at any time. For patients who are lost to follow-up, the investigator or designee should show "due diligence" by documenting in the source documents steps taken to contact the patient, e.g., dates of telephone calls, registered letters, etc.

The end of treatment visit and its associated assessments should occur within 7 days of the last study treatment.

Patients may discontinue study treatment for any of the following reasons:

- Adverse event(s)
- Lost to follow-up
- Physician decision
- Pregnancy
- Protocol deviation
- Technical problems
- Subject/guardian decision
- Progressive disease
- Study terminated by the sponsor
- Non-compliant with study treatment
- No longer requires treatment
- Treatment duration completed as per protocol (optional, to be used if only a fixed number of cycles is given)

Death is a reason which "*must*" lead to discontinuation of patient from trial.

16.1.13.3 End of post-treatment follow-up (study phase completion)

End of post-treatment follow-up visit will be completed after discontinuation of study treatment and post-treatment evaluations but prior to collecting survival follow-up.

Patients may provide study phase completion information for one of the following reasons:

- Adverse event
- Lost to follow-up
- Physician decision
- Pregnancy
- Protocol deviation
- Technical problems
- Subject/guardian decision
- Death
- Progressive disease
- Study terminated by the sponsor

16.1.13.4 Medical validation of programmed overall lesion response

In order to be as objective as possible the RECIST programmed calculated response assessment is very strict regarding measurement methods (i.e. any assessment with more or less sensitive method than the one used to assess the lesion at baseline is considered UNK) and not available evaluations (i.e. if any target or non-target lesion was not evaluated the whole overall lesion response is UNK unless remaining lesions qualified for PD). This contrasts with the slightly more flexible guidance given to local investigators (and to the central reviewers) to use expert judgment in determining response in these type of situations, and therefore as a consequence discrepancies between the different sources of response assessment often arise. To ensure the quality of response assessments from the local site and/or the central reviewer, the responses may be re-evaluated by clinicians (based on local investigator data recorded in eCRF or based on central reviewer data entered in the database) at Novartis or external experts. In addition, data review reports will be available to identify assessments for which the investigators' or central reader's opinion does not match the programmed calculated response based on RECIST criteria. This may be queried for clarification. However, the investigator or central reader's response assessment will never be overruled.

If Novartis elect to invalidate an overall lesion response as evaluated by the investigator or central reader upon internal or external review of the data, the calculated overall lesion response at that specific assessment is to be kept in a dataset. This must be clearly documented in the RAP documentation and agreed before database lock. This dataset should be created and stored as part of the 'raw' data.

Any discontinuation due to 'Disease progression' without documentation of progression by RECIST criteria should be carefully reviewed. Only patients with documented deterioration of symptoms indicative of progression of disease should have this reason for discontinuation of treatment or study evaluation.

16.1.13.5 Programming rules

The following should be used for programming of efficacy results:

16.1.13.5.1 Calculation of 'time to event' variables

Time to event = end date - start date + 1 (in days)

When no post-baseline tumor assessments are available, the date of randomization/start of treatment will be used as end date (duration = 1 day) when time is to be censored at last tumor assessment, i.e. time to event variables can never be negative.

16.1.13.5.2 Incomplete assessment dates

All investigation dates (e.g. X-ray, CT scan) must be completed with day, month and year.

If one or more investigation dates are incomplete but other investigation dates are available, this/these incomplete date(s) are not considered for calculation of the assessment date (and assessment date is calculated as outlined in Section 16.1.12.7). If all measurement dates have no day recorded, the 1st of the month is used.

If the month is not completed, for any of the investigations, the respective assessment will be considered to be at the date which is exactly between previous and following assessment. If a previous and following assessment is not available, this assessment will not be used for any calculation.

16.1.13.5.3 Incomplete dates for last known date patient alive or death

All dates must be completed with day, month and year. If the day is missing, the 15th of the month will be used for incomplete death dates or dates of last contact.

16.1.13.5.4 Non-target lesion response

If no non-target lesions are identified at baseline (and therefore not followed throughout the study), the non-target lesion response at each assessment will be considered 'not applicable (NA)'.

16.1.13.5.5 Study/project specific programming

The standard analysis programs need to be adapted for each study/project.

16.1.13.5.6 Censoring reason

In order to summarize the various reasons for censoring, the following categories will be calculated for each time to event variable based on the treatment completion page, the study evaluation completion page and the survival page.

For survival the following censoring reasons are possible:

- Alive
- Lost to follow-up

For PFS and TTP (and therefore duration of responses) the following censoring reasons are possible:

- Ongoing without event
- Lost to follow-up
- Withdrew consent
- Adequate assessment no longer available*
- Event documented after two or more missing tumor assessments (optional, see Table 16-2)
- Death due to reason other than underlying cancer *(only used for TTP and duration of response)*
- Initiation of new anti-cancer therapy

* Adequate assessment is defined in Section 16.1.12.7. This reason is applicable when adequate evaluations are missing for a specified period prior to data cut-off (or prior to any other censoring reason) corresponding to the unavailability of two or more planned tumor assessments prior to the cut-off date. The following clarifications concerning this reason should also be noted:

- This may be when there has been a definite decision to stop evaluation (e.g. reason="Sponsor decision" on study evaluation completion page), when patients are not followed for progression after treatment completion or when only UNK assessments are available just prior to data cut-off).
- The reason "Adequate assessment no longer available" also prevails in situations when another censoring reason (e.g. withdrawal of consent, loss to follow-up or alternative anti-cancer therapy) has occurred more than the specified period following the last adequate assessment.
- This reason will also be used to censor in case of no baseline assessment.

16.1.14 References (available upon request)

Dent S, Zee B, Dancey J, et al (2001) Application of a new multinomial phase II stopping rule using response and early progression. J Clin Oncol; 19: 785-791.

Eisenhauer EA, Therasse P, Bogaerts J, et al (2009) New response evaluation criteria in solid tumors: revised RECIST guideline (version 1.1). Eur J Cancer; 45(2):228-47.

Ellis S, Carroll KJ, Pemberton K, et al (2008) Analysis of duration of response in oncology trials. Contemp Clin Trials; 29: 456-465.

European Medicines Agency (2017) Guideline on the evaluation of anticancer medicinal products in man. EMA/CHMP/205/95 Rev.5. 22-Sep-2017. London, United Kingdom.

Food and Drug Administration (2005) Clinical Trial Endpoints for the Approval of Cancer Drugs and Biologics. Guidance for Industry. April 2005. Oncology Center of Excellence, Center for Drug Evaluation and Research (CDER), and Center for Biologics Evaluation and Research (CBER). Silver Spring, MD.

Food and Drug Administration (2007) Clinical Trial Endpoints for the Approval of Cancer Drugs and Biologics. Guidance for Industry.May 2007. Revision 1. Oncology Center of Excellence, Center for Drug Evaluation and Research (CDER), and Center for Biologics Evaluation and Research (CBER). Silver Spring, MD.

Morgan TM (1988) Analysis of duration of response: a problem of oncology trials. Cont Clin Trials; 9: 11-18.

Therasse P, Arbuck S, Eisenhauer E, et al (2000) New Guidelines to Evaluate the Response to Treatment in Solid Tumors. J Natl Cancer Inst; 92(3):205-16.

16.2 Appendix 2: Statistical details of the Bayesian logistic regression model (BLRM): priors, design, operating characteristics and hypothetical data scenarios

This appendix provides details of the statistical model, the derivation of prior distributions from pre-clinical data, the results of the Bayesian analyses and respective dosing decisions for some hypothetical data scenarios, and a simulation study of the operating characteristics of the model.

16.2.1 Statistical considerations for DYP688 single agent

16.2.1.1 Statistical model

Let $\pi(d)$ be the risk of DLT for DYP688 given at dose d, the dose-DLT model is logistic:

$$\text{logit}\big(\pi(d)\big) = \log(\alpha) + \beta \log(d/d^{*})$$

where $d^{*} = 12$ mg/kg is used as a reference to scale the doses of DYP688. Hence, α (>0) is the odds of a DLT at d^{*}mg; and β (>0) is the increase in the log-odds of a DLT by a unit increase in log-dose.

16.2.1.2 Prior specifications

The Bayesian approach requires the specification of prior distributions for the model parameters $\log(\alpha)$ and $\log(\beta)$. Based on pre-clinical toxicology data, weakly informative priors were derived for these parameters.

16.2.1.2.1 Prior distribution for the logistic parameters

Pre-clinical toxicology data showed no definitive DYP688 related toxicity, thereby making it impossible to predict the MTD in human. For this reason the priors of model parameter distributions are defined based on a weakly informative prior. A vague bivariate normal prior distribution for the model parameters, (log(α), log(β)), was elicited based on a plausible mean values but allowing for considerable uncertainty, essentially making sure that the a-priori range for the parameters cover a wide range of plausible values.

Assuming a median prior probability of DLT equal to 0.10 at the dose reference results in a mean of log(α) equal to logit (0.10) = -2.20.

Assuming the odds is doubled when doubling the dose results in a mean of log (β) equal to 0.

The standard deviations of log (α) and log (β) are set respectively to 1.4 and 1, and the correlation is assumed to be 0.

The prior distribution parameters of the DYP688 for single agent are provided in Table 16-6 and summary of prior distribution of DLT rates in Table 16-7. The doses not meeting the overdose control criterion are bolded in the table, i.e., doses not eligible at the start of the study (under the prior). As noted below, doses of 16 mg/kg and higher cannot be used as starting dose.

Table 16-6 Prior distribution parameters-DYP688 SA (Q2W regimen)

	(log(α), log(β))	
**Means**	**SDs**	**Correlation**
(-2.20, 0)	(1.4, 1)	0

Table 16-7 Summary of prior distribution of DLT rates- DYP688 SA (Q2W regimen)

Dose level (mg/kg)	Prior probabilities that P(DLT) is in interval:					Quantiles		
	0-0.16	0.16-0.33	0.33-1	Mean	SD	0.025	Median	0.975
1	0.946	0.039	0.015	0.036	0.077	0	0.007	0.264
2	0.921	0.056	0.023	0.048	0.09	0	0.014	0.317
4*	0.875	0.087	0.038	0.069	0.108	0	0.027	0.394
8	0.775	0.148	0.077	0.11	0.136	0.002	0.058	0.512
12	0.651	0.21	0.139	0.159	0.165	0.007	0.098	0.628
16	0.534	0.234	0.232	0.219	0.211	0.01	0.142	0.793

* Starting dose

16.2.1.3 Hypothetical dose escalation scenarios

In order to show how the proposed approach works, hypothetical dose escalation scenarios are investigated. The model will be used to assess risk to future patients during the clinical trial based on the observed DLTs. This assessment of risk is then used to guide future dosing decisions. In choosing a dose to explore, the clinical team will also consider all available clinical, safety, PK and PD, but will not consider a dose for use unless it satisfies the EWOC criterion.

Table 16-8 illustrates a selection of possible dose escalation scenarios.

Table 16-8 Hypothetical scenarios

Scenario	Cohort	Dose level (mg)	Ntox / Npat	Next dose level (NDL) (mg)	P(target) at NDL	P(over) at NDL	Median at NDL
1	1	4	0/2	8	0.119	0.041	0.046
2	1	4	1/2	4	0.279	0.179	0.144
3	1	4	1/4	8	0.338	0.183	0.168
4	1	4	2/3	1	0.296	0.218	0.166
5	1	4	1/3	8	0.33	0.242	0.191
6	1	4	1/3	4	0.348	0.135	0.155
	2	8	1/3				
7	1	4	1/3	8	0.449	0.151	0.188
	2	8	1/5				
8	1	4	1/3	12	0.441	0.237	0.219
	2	8	1/6				
9	1	4	1/5	8	0.438	0.193	0.2
	2	8	1/3				
10	1	4	1/5	12	0.435	0.234	0.215
	2	8	1/5				

Scenario	Cohort	Dose level (mg)	Ntox / Npat	Next dose level (NDL) (mg)	P(target) at NDL	P(over) at NDL	Median at NDL
11	c	4	0/2	12	0.345	0.23	0.188
	2	8	1/3				
12	1	4	0/2	8	0.387	0.107	0.159
	2	8	1/3				
	3	12	1/3				
13	1	4	0/2	12	0.449	0.15	0.188
	2	8	1/3				
	3	12	1/5				
14	1	4	0/2	12	0.452	0.163	0.193
	2	8	1/3				
	3	12	1/3				
	4	8	0/3				
15	1	4	0/2	16	0.418	0.209	0.201
	2	8	1/5				
	3	12	1/6				
16	1	4	1/4	12	0.448	0.212	0.211
	2	8	1/6				
17	1	4	1/4	12	0.517	0.245	0.236
	2	8	1/6				
	3	12	1/3				
18	1	4	1/6	12	0.438	0.213	0.207
	2	8	1/5				
19	1	4	1/5	8	0.438	0.193	0.2
	2	8	1/3				
20	1	4	1/5	8	0.505	0.115	0.19
	2	8	1/5				
	3	12	1/3				
21	1	4	1/5	16	0.505	0.226	0.228
	2	8	1/5				
	3	12	1/6				
22	1	4	1/5	12	0.518	0.172	0.211
	2	8	1/6				
	3	12	1/4				

16.2.1.4 Operating characteristics

In order to show how the proposed design performs under different true dose- DLT profiles, various hypothetical scenarios were investigated.

16.2.1.5 Simulation setup

Table 16-9 shows 5 dose-DLT scenarios, taking scenario 1 (true DLT rates equal to median prior DLT rates) as the basis. Scenario 2 has decreased DLT rates compared to scenario 1. Scenarios 3, 4 and 5 have increased DLT rates compared to scenario 1:

The following table presents the true underlying probabilities of DLT for each scenario.

Table 16-9 True probability of DLT for simulation scenarios

DYP688	1 mg/kg	2 mg/kg	4 mg/kg	8 mg/kg	12 mg/kg	16 mg/kg
Scenario 1	0.007	0.014	0.027	0.058	0.098	0.142
Scenario 2	0.005	0.010	0.019	0.041	0.071	0.104
Scenario 3	0.014	0.028	0.053	0.110	0.179	0.249
Scenario 4	0.015	0.025	0.10	0.20	0.30	0.40
Scenario 5	0.45	0.55	0.65	0.75	0.85	0.9

16.2.1.6 Operating characteristics

A simulation study is used to illustrate the long run performance of the Bayesian dose escalation model. Several example scenarios were investigated (Table 16-9), and in each case 1000 trials were simulated, with results summarized in Table 16-10.

Scenarios

In order to show how the design performs, 5 hypothetical scenarios were investigated:

1. Scenario 1 represents a scenario which is in line with the prior, i.e. the true underlying toxicity is set to the median DLT rate estimated by BLRM with only prior information.
2. Scenario 2 represents a scenario assuming lower toxicity, i.e. the true underlying toxicity is lower than what would be predicted from the prior;
3. Scenario 3 represents a scenario assuming higher toxicity, i.e. the true underlying toxicity is higher than what would be predicted from the prior.
4. Senario 4 represents a scenario assuming high toxicity at the highest dose level.
5. Scenario 5 represents a scenario assuming much higher toxicity, i.e. the true underlying toxicity is higher than 45% for all dose levels.

The true probabilities used in the simulation are presented in Table 16-9 for each scenario above.

Simulation details

1000 trials were used to simulate each scenario. The starting dose was chosen as 4 mg/kg every 2 weeks. The provisional doses to jump to follows the protocol specifications and are 8, 12, 16 mg/kg (single dose Q2W). The number of patients to enroll in each cohort and stopping rule used to declare MTD were defined as:

- Cohort size: 2 patients for the first cohort and 3-6 patients for the following cohorts
- Minimum number of patients to be treated in the trial: 14
- Minimum number of patients treated at a given dose in order to declare MTD: 6

Metrics

Operating characteristics were reviewed for the simulations to compare the relative performance under each true probability scenario. The metrics reviewed were:

1. Average proportion of patients receiving a dose with true P(DLT) < 16% on study (I)

2. Average proportion of patients receiving a dose with a target dose on study (II)
3. Average proportion of patients receiving a dose with true P(DLT) ≥ 33% on study (III)
4. Probability of recommending a dose with true P(DLT) < 16% as MTD (IV)
5. Probability of recommending a target dose as MTD (V)
6. Probability of recommending a dose with true P(DLT) ≥ 33% as MTD (VI)
7. Percentage of trials stops without declaring MTD
8. Average number of patients treated on a trial

Simulation results

Table 16-5 below summarizes the simulated operating characteristics of the model for the 4 different scenarios studied, additionally showing the percentage of trials stopped before declaring MTD when all doses were considered too toxic.

Table 16-10 Summary of simulation results

Scenario	Metric							
	I	II	III	IV	V	VI	Stopped	AveN
1	100	0	0	99.7	0	0	0.3	17.7
2	100	0	0	99.9	0	0	0.1	17.1
3	45.25	54.75	0	13.4	83.7	0	2.9	19.5
4	24.46	57.41	18.14	12.9	65.8	15.8	5.5	20.7
5	0	0	100	0	0	3	97	6.5

The simulated operating characteristics presented show that the model performs well under the hypothetical scenarios investigated.

In scenario 1 and 2, since the true DLT rates at all provisional dose levels are less than 16%, all patients received under toxic doses (metric I). All identified MTDs fall within under-dose toxicity range (metric IV).

In scenario 3, the probability that the identified MTD falls within the target interval (metric V) is 83.7%, showing good targeting of MTD even if the true DLT rates are greater than what would be predicted from the prior. Since none of the provisional dose levels have DLT rates ≥ 0.33, the probability of identifying a dose with P(DLT) ≥ 0.33 as MTD (metric VI) is 0.

In scenario 4, because the true DLT rate of provisional dose (16 mg/kg) is greater than 33%, the probability that the identified MTD being overly toxic (metric VI) is 15.8%, which is expected and acceptable.

In scenario 5, because the true DLT rates for all dose levels are greater than 45%, none of the trials identify the MTD falling within the target interval. There is 3% (VI) of the chance that an over-dose level would be declared as MTD. Otherwise the trial will stops for being overly toxic without declaring a MTD (97%).

In scenario 1, 2the percentage of trials that were stopped without declaring MTD is relatively very low, under 1%.

On average, trials enroll 18-21 patients in scenario 1, 2, 3 and 4. For scenario 5, an average of 6.5 patients are enrolled.

In conclusion, the simulations performed illustrate that the model has reasonable operating characteristics. The probability of identifying a safe dose as MTD is high (except for scenario 5). On the contrary, the probability to identify an MTD with P(DLT) ≥ 0.33 (metric V) is low across all scenarios.

16.3 Creatinine clearance

Note: The estimated GFR for pediatric patients should be calculated using the formula in Schwartz and Work, 2009:

- eGFR (mL/min/1.73m2)= k x Length/Scr,
- L is height in centimeters (cm), Scr is serum creatinine in milligrams per deciliter (mg/dL), and k is 0.55 for children and adolescent females and 0.7 for adolescent males.

Schwartz GJ, Work DF. Measurement and estimation of GFR in children and adolescents. Clin J Am Soc Nephrol. 2009 Nov;4(11):1832-43.

For adult patients Creatinine clearance will be calculated using the following formulas:

- Male GFR = (140 - age) x (weight) / (sCr x 72)
- Female GFR = (140 - age) x (weight) x 0.85 / (sCr x 72)

GFR is Glomerular Filtration Rate in ml/min; Age is in years; Weight is Lean Body Mass in kilograms; sCr is Serum Creatinine in mg/dl

The formulas above should be altered in the following cases:

1. Overweight (BMI >25 kg/m2)
    a. Calculate based on adjusted body weight
    b. Adjusted body weight = wtKgIdeal + 0.4 * (wtKgActual - wtKgIdeal)
2. Underweight
    a. Use actual weight
    b. Do not round up to Serum Creatinine (significantly underestimates GFR)
3. Elderly
    a. Do not round up to Serum Creatinine (significantly underestimates GFR)
4. Amputation
    a. Measure 24 hour Creatinine Clearance

Wallach, J. Interpretation of Diagnostic Tests. Ed. 8 (2007) Lippincott Williams and Wilkins.

16.4 Drugs that are to be used with caution

Drugs with a known risk of Torsades de Pointes (TdP) should be used with caution and alternative medications should be considered. The QT drugs listed below (Table 16-11) is obtained from the website crediblemeds.org/healthcare-providers/drug-list, which is maintained by the Arizona Center for Education and Research on Therapeutics. Please contact the medical monitor with any questions.

Table 16-11 List of medication that should be used with caution

Class of medication	Drug Name
With Known risk of Torsades des Pointes*	aclarubicin, amiodarone, anagrelide, arsenic trioxide, astemizole1, azithromycin, bepridil, cesium chloride, chloroquine, chlorpromazine, chlorprothixene, cilostazol, ciprofloxacin, cisapride, citalopram, clarithromycin, cocaine, disopyramide, dofetilide, domperidone, donepezil, dronedarone, droperidol, erythromycin, escitalopram, flecainide, fluconazole, gatifloxacin, grepafloxacin, halofantrine, haloperidol, hydroquinidine (dihydroquinidine), hydroxychloroquine, ibogaine, ibutilide, levofloxacin, levomepromazine (methotrimeprazine), levomethadyl acetate, levosulpiride, meglumine antimoniate, mesoridazine, methadone, moxifloxacin, nifekalant, ondansetron, oxaliplatin, papaverine HCl (intra-coronary), pentamidine, pimozide, probucol, procainamide, propofol, quinidine, roxithromycin, sertindole, sevoflurane, sotalol, sparfloxacin, sulpiride, sultopride, terfenadine, terlipressin, terodiline, thioridazine, vandetanib
*Check crediblemeds.org/healthcare-providers/drug-list for the most updated list.	

Clinical Development

DYP688

Manuscript 2025

Statistical Analysis Plan

Author(s):	Trial Statistician
Document type:	RAP Documentation
Document status:	Final
Release date:	29-May-2025
Number of pages:	34

Property of Novartis
Confidential
May not be used, divulged, published or otherwise disclosed
without the consent of Novartis

Document History – Changes compared to previous final version of SAP

Date	Reason for update	Outcome for update	Section and title impacted (Current)
29-May-2025	Creation of final version	N/A - First version	NA

Table of contents

Table of contents 3
List of abbreviations 4
1 General guidance 5
1.1 Studies covered by this document 5
1.2 Population definitions 5
1.3 Treatment group labels and ordering 6
1.4 Document headers and footers 6
1.5 List of outputs 6
1.6 General specification 7
1.7 Output shells 7
1.8 Data panels to be cleaned (RAVE) 32

List of abbreviations

AE	Adverse Event
ASCO	American Society of Clinical Oncology
CSR	Clinical Study Report
PK	Pharmacokinetic
SAP	Statistical Analysis Plan
SOC	System Organ Class

1 General guidance

This document serves as the reference for tables, listing and figures required for the manuscript preparation of DYP688 study. The required tables, listings, and figures will be delivered per the timeline in Table 1-1. It is adapted to meet specific needs for DYP688 program, based upon the Oncology Standard Tables, Listings and Figures for CSR documentation.

Table 1-1 Tasks and timelines

Deliverable	Activities	Completion date
	Clinical Data cut-off	Thursday, May 1, 2025
	All data entered	Tuesday, May 6, 2025
	Data clean	Friday, May 9, 2025
	All queries issued/resolved	Wednesday, May 14, 2025
	DM snapshot for IB	Thursday, May 15, 2025
	Final clinical data transfer/upload	Thursday, May 15, 2025
	Data available in LSH for extraction to GPS	Friday, May 16, 2025
	Programming of Clinical Outputs	Friday, May 30, 2025
	Review of outputs by team	Wednesday, June 4, 2025

1.1 Studies covered by this document

- CDYP688A12101

1.2 Population definitions

Refer to the study protocol and SAP for CSR of CDYP688A12101.

1.3 Treatment group labels and ordering

- Treatment group label 1: DYP688 single agent dose escalation part:

4 mg/kg Q2W	8 mg/kg Q2W	12 mg/kg Q2W	16 mg/kg Q2W	24 mg/kg Q2W	12 mg/kg QW	16 mg/kg QW	All Patients

1.4 Document headers and footers

- The following header will be used for all tables, listings and figures outlined in this document:

DYP688 Manuscript 2025 – <*study ID, e.g. CDYP688A12101*> (Clinical cut-off date: 01-May-2025)

Clinical cut-off is 01-May-2025.

- The following footer will be used for all tables, listings and figures outlined in this document:

report/pgm_saf/<pgm_name.sas> DDMMMYYYY: HH:MM Draft/final Version Outputs

1.5 List of outputs

Table 1-2 shows the outputs to be produced for the Manuscript for DYP688 study. The shells for the outputs are provided in Section 1.7 and the panels to be cleaned are detailed in Section 1.8.

Table 1-2 List of tables, figures and listings to be produced by study

	Output	Short description	Required for abstract (Y/N)
Outputs to support Manuscript	Table 1-1	Subject disposition by treatment groups in dose escalation (Full Analysis Set)	Y
	Table 1-2	Subject demographics and baseline characteristics (Full analysis set)	Y
	Table 1-3	Disease characteristics (Full analysis set)	Y
	Table 1-4	Prior antineoplastic therapy – Medication (Full analysis set)	Y
	Table 1-5	Duration of exposure to DYP688 (Safety set)	Y
	Table 1-6	Dose of DYP688 received (Safety set)	N
	Table 1-7	Overview of adverse events (Safety set)	Y
	Table 1-8	Adverse events by preferred term and treatment group in dose escalation (Safety set)	Y
	Table 1-9	Adverse events, suspected to be study treatment related, by preferred term and treatment group in dose escalation (Safety set)	Y
	Table 1-10	Adverse events, suspected to be study treatment related, by preferred term, maximum grade and treatment group in dose escalation (Safety set)	N
	Table 1-11	Serious adverse events, by system organ class, preferred term, maximum grade and treatment in dose escalation (Safety set)	Y
	Table 1-12	Suspected serious adverse events, by system organ class, preferred term, maximum grade and treatment (Safety set)	N

	Output	Short description	Required for abstract (Y/N)
	Table 1-13	Best overall response summary (Full analysis set)	Y
	Figure 1-1	Waterfall plot for best percentage change from baseline in sum of longest diameters based on local radiology review (Full analysis set)	Y
	Figure 1-2	Overall responses and percentage changes from baseline of sum of longest diameters of targeted lesions at each timepoint (Full analysis set)	Y
	Figure 1-3	Time on study drug and duration of response (Full analysis set)	Y
	Figure 1-4	Tornado plot (Full analysis set)	Y

Note: all tables and listings will be summarized by treatment group.

Tables and figures required for abstract submission using data from Clinical cut-off snapshot. The data will be refreshed and cleaned after abstract submission. The full list of outputs from table 1-2 will be produced for the presentation.

1.6 General specification

The outputs will be produced under in-text layout.

For AE summaries, the following AE preferred terms which are equivalent will be grouped and reported once for any patient who has either of equivalent preferred terms:

A patient with either ‘Platelet count decreased’ or ‘Thrombocytopenia’ will be reported once under ‘Thrombocytopenia’ and SOC ‘Blood and lymphatic system disorders’

A patient with either ‘Neutrophil count decreased’ or ‘Neutropenia’ will be reported once under ‘Neutropenia’ and SOC ‘Blood and lymphatic system disorders’

A patient with either ‘Abdominal pain’ or ‘Abdominal pain lower’ or ‘Abdominal pain upper’ will be reported once under ‘Abdominal pain’ and SOC ‘Gastrointestinal disorders’

A patient with either ‘White blood cell count decreased’ or ‘Leukopenia’ will be reported once under ‘Leukopenia’ and SOC ‘Blood and lymphatic system disorders’

A patient with either 'Dyspnoea exertional' or 'Dyspnoea' will be reported once under ‘'Dyspnoea ’ and SOC ‘Respiratory, thoracic and mediastinal disorders’

1.7 Output shells

Table 1-1 Subject disposition by treatment groups in dose escalation (Full analysis set)

	4 mg/kg Q2W N=xxx n (%)	8 mg/kg Q2W N=xxx n (%)	12 mg/kg Q2W N=xxx n (%)	16 mg/kg Q2W N=xxx n (%)	24 mg/kg Q2W N=xxx n (%)	12 mg/kg QW N=xxx n (%)	16 mg/kg QW N=xxx n (%)	All subjects N=xxx n (%)
Subjects treated								
Treated	xx (xx.x)	xx (xx.x)	xx (xx.x)	xx (xx.x)	xx (xx.x)	xx (xx.x)	xx (xx.x)	xx (xx.x)
Treatment ongoing *	xx (xx.x)	xx (xx.x)	xx (xx.x)	xx (xx.x)	xx (xx.x)	xx (xx.x)	xx (xx.x)	xx (xx.x)
Discontinued <from treatment/from study>	xx (xx.x)	xx (xx.x)	xx (xx.x)	xx (xx.x)	xx (xx.x)	xx (xx.x)	xx (xx.x)	xx (xx.x)
Reason for discontinuation								
<Reason 1>	xx (xx.x)	xx (xx.x)	xx (xx.x)	xx (xx.x)	xx (xx.x)	xx (xx.x)	xx (xx.x)	xx (xx.x)
<Reason 2>	xx (xx.x)	xx (xx.x)	xx (xx.x)	xx (xx.x)	xx (xx.x)	xx (xx.x)	xx (xx.x)	xx (xx.x)
etc.								
**Post-treatment follow-up for subjects who discontinued <treatment/epoch>**								
Did not enter post-treatment follow-up	xx (xx.x)	xx (xx.x)	xx (xx.x)	xx (xx.x)	xx (xx.x)	xx (xx.x)	xx (xx.x)	xx (xx.x)
Entered post-treatment follow-up, ongoing *	xx (xx.x)	xx (xx.x)	xx (xx.x)	xx (xx.x)	xx (xx.x)	xx (xx.x)	xx (xx.x)	xx (xx.x)
Entered post-treatment follow-up, discontinued	xx (xx.x)	xx (xx.x)	xx (xx.x)	xx (xx.x)	xx (xx.x)	xx (xx.x)	xx (xx.x)	xx (xx.x)
Reason for discontinuation	xx (xx.x)	xx (xx.x)	xx (xx.x)	xx (xx.x)	xx (xx.x)	xx (xx.x)	xx (xx.x)	xx (xx.x)
<Reason 1>	xx (xx.x)	xx (xx.x)	xx (xx.x)	xx (xx.x)	xx (xx.x)	xx (xx.x)	xx (xx.x)	xx (xx.x)
<Reason 2>	xx (xx.x)	xx (xx.x)	xx (xx.x)	xx (xx.x)	xx (xx.x)	xx (xx.x)	xx (xx.x)	xx (xx.x)
etc.								

Programming Note: OSO 3.0 T_DS_301_IN

Table 1-2 Subject demographics and baseline characteristics (Full analysis set)

Characteristic<br>Categories/Statistics	4 mg/kg Q2W N=xxx n (%)	8 mg/kg Q2W N=xxx n (%)	12 mg/kg Q2W N=xxx n (%)	16 mg/kg Q2W N=xxx n (%)	24 mg/kg Q2W N=xxx n (%)	12 mg/kg QW N=xxx n (%)	16 mg/kg QW N=xxx n (%)	All Subjects N=xxx n (%)
Age group – n (%)								
12-<18 years	xx (xx.x)	xx (xx.x)	xx (xx.x)	xx (xx.x)	xx (xx.x)	xx (xx.x)	xx (xx.x)	xx (xx.x)
18-< 65 years	xx (xx.x)	xx (xx.x)	xx (xx.x)	xx (xx.x)	xx (xx.x)	xx (xx.x)	xx (xx.x)	xx (xx.x)
>= 65 years	xx (xx.x)	xx (xx.x)	xx (xx.x)	xx (xx.x)	xx (xx.x)	xx (xx.x)	xx (xx.x)	xx (xx.x)
Age (years)								
n	xx	xx	xx	xx	xx	xx	xx	xx
Mean (SD)	xx.x (xx.xx)	xx.x (xx.xx)	xx.x (xx.xx)	xx.x (xx.xx)	xx.x (xx.xx)	xx.x (xx.xx)	xx.x (xx.xx)	xx.x (xx.xx)
Median	xx.x	xx.x	xx.x	xx.x	xx.x	xx.x	xx.x	xx.x
Q1 - Q3	xx.x - xx.x	xx.x - xx.x	xx.x - xx.x	xx.x - xx.x	xx.x - xx.x	xx.x - xx.x	xx.x - xx.x	xx.x - xx.x
Min - Max	xx - xx	xx - xx	xx - xx	xx - xx	xx - xx	xx - xx	xx - xx	xx - xx
Sex – n (%)								
Male	xx (xx.x)	xx (xx.x)	xx (xx.x)	xx (xx.x)	xx (xx.x)	xx (xx.x)	xx (xx.x)	xx (xx.x)
Female	xx (xx.x)	xx (xx.x)	xx (xx.x)	xx (xx.x)	xx (xx.x)	xx (xx.x)	xx (xx.x)	xx (xx.x)
Race – n (%)								
Caucasian	xx (xx.x)	xx (xx.x)	xx (xx.x)	xx (xx.x)	xx (xx.x)	xx (xx.x)	xx (xx.x)	xx (xx.x)
Black	xx (xx.x)	xx (xx.x)	xx (xx.x)	xx (xx.x)	xx (xx.x)	xx (xx.x)	xx (xx.x)	xx (xx.x)
Asian	xx (xx.x)	xx (xx.x)	xx (xx.x)	xx (xx.x)	xx (xx.x)	xx (xx.x)	xx (xx.x)	xx (xx.x)
Native American	xx (xx.x)	xx (xx.x)	xx (xx.x)	xx (xx.x)	xx (xx.x)	xx (xx.x)	xx (xx.x)	xx (xx.x)
Pacific Islander	xx (xx.x)	xx (xx.x)	xx (xx.x)	xx (xx.x)	xx (xx.x)	xx (xx.x)	xx (xx.x)	xx (xx.x)
Unknown	xx (xx.x)	xx (xx.x)	xx (xx.x)	xx (xx.x)	xx (xx.x)	xx (xx.x)	xx (xx.x)	xx (xx.x)
Other	xx (xx.x)	xx (xx.x)	xx (xx.x)	xx (xx.x)	xx (xx.x)	xx (xx.x)	xx (xx.x)	xx (xx.x)

Ethnicity – n (%)								
Hispanic/Latino	xx (xx.x)	xx (xx.x)	xx (xx.x)	xx (xx.x)	xx (xx.x)	xx (xx.x)	xx (xx.x)	xx (xx.x)
East Asian	xx (xx.x)	xx (xx.x)	xx (xx.x)	xx (xx.x)	xx (xx.x)	xx (xx.x)	xx (xx.x)	xx (xx.x)
Southeast Asian	xx (xx.x)	xx (xx.x)	xx (xx.x)	xx (xx.x)	xx (xx.x)	xx (xx.x)	xx (xx.x)	xx (xx.x)
South Asian	xx (xx.x)	xx (xx.x)	xx (xx.x)	xx (xx.x)	xx (xx.x)	xx (xx.x)	xx (xx.x)	xx (xx.x)
West Asian	xx (xx.x)	xx (xx.x)	xx (xx.x)	xx (xx.x)	xx (xx.x)	xx (xx.x)	xx (xx.x)	xx (xx.x)
Russian	xx (xx.x)	xx (xx.x)	xx (xx.x)	xx (xx.x)	xx (xx.x)	xx (xx.x)	xx (xx.x)	xx (xx.x)
Japanese	xx (xx.x)	xx (xx.x)	xx (xx.x)	xx (xx.x)	xx (xx.x)	xx (xx.x)	xx (xx.x)	xx (xx.x)
Chinese	xx (xx.x)	xx (xx.x)	xx (xx.x)	xx (xx.x)	xx (xx.x)	xx (xx.x)	xx (xx.x)	xx (xx.x)
Indian	xx (xx.x)	xx (xx.x)	xx (xx.x)	xx (xx.x)	xx (xx.x)	xx (xx.x)	xx (xx.x)	xx (xx.x)
Mixed Ethnicity	xx (xx.x)	xx (xx.x)	xx (xx.x)	xx (xx.x)	xx (xx.x)	xx (xx.x)	xx (xx.x)	xx (xx.x)
Unknown	xx (xx.x)	xx (xx.x)	xx (xx.x)	xx (xx.x)	xx (xx.x)	xx (xx.x)	xx (xx.x)	xx (xx.x)
Other	xx (xx.x)	xx (xx.x)	xx (xx.x)	xx (xx.x)	xx (xx.x)	xx (xx.x)	xx (xx.x)	xx (xx.x)
Weight (kg)								
n	xx	xx	xx	xx	xx	xx	xx	xx
Mean (SD)	xx.x (xx.xx)	xx.x (xx.xx)	xx.x (xx.xx)	xx.x (xx.xx)	xx.x (xx.xx)	xx.x (xx.xx)	xx.x (xx.xx)	xx.x (xx.xx)
Median	xx.x	xx.x	xx.x	xx.x	xx.x	xx.x	xx.x	xx.x
Q1 - Q3	xx.x - xx.x	xx.x - xx.x	xx.x - xx.x	xx.x - xx.x	xx.x - xx.x	xx.x - xx.x	xx.x - xx.x	xx.x - xx.x
Min - Max	xx - xx	xx - xx	xx - xx	xx - xx	xx - xx	xx - xx	xx - xx	xx - xx
ECOG PS – n (%)								
0	xx (xx.x)	xx (xx.x)	xx (xx.x)	xx (xx.x)	xx (xx.x)	xx (xx.x)	xx (xx.x)	xx (xx.x)
1	xx (xx.x)	xx (xx.x)	xx (xx.x)	xx (xx.x)	xx (xx.x)	xx (xx.x)	xx (xx.x)	xx (xx.x)
Lactate dehydrogenase- n (%)								
>ULN	xx (xx.x)	xx (xx.x)	xx (xx.x)	xx (xx.x)	xx (xx.x)	xx (xx.x)	xx (xx.x)	xx (xx.x)
≤ULN	xx (xx.x)	xx (xx.x)	xx (xx.x)	xx (xx.x)	xx (xx.x)	xx (xx.x)	xx (xx.x)	xx (xx.x)

Table 1-3 Disease characteristics (Full analysis set)

Characteristic<br>Categories/Statistics	4 mg/kg<br>Q2W<br>N=xxx<br>n (%)	8 mg/kg<br>Q2W<br>N=xxx<br>n (%)	12 mg/kg<br>Q2W<br>N=xxx<br>n (%)	16 mg/kg<br>Q2W<br>N=xxx<br>n (%)	24 mg/kg<br>Q2W<br>N=xxx<br>n (%)	12 mg/kg<br>QW<br>N=xxx<br>n (%)	16 mg/kg<br>QW<br>N=xxx<br>n (%)	All<br>Subjects<br>N=xxx<br>n (%)
Primary site of cancer-n (%)								
Site 1	xx (xx.x)	xx (xx.x)	xx (xx.x)	xx (xx.x)	xx (xx.x)	xx (xx.x)	xx (xx.x)	xx (xx.x)
Site 2 etc.	xx (xx.x)	xx (xx.x)	xx (xx.x)	xx (xx.x)	xx (xx.x)	xx (xx.x)	xx (xx.x)	xx (xx.x)
Predominant Histology/Cytology								
Melanoma	xx (xx.x)	xx (xx.x)	xx (xx.x)	xx (xx.x)	xx (xx.x)	xx (xx.x)	xx (xx.x)	xx (xx.x)
Other	xx (xx.x)	xx (xx.x)	xx (xx.x)	xx (xx.x)	xx (xx.x)	xx (xx.x)	xx (xx.x)	xx (xx.x)
Diagnosis of Disease								
Uveal Melanoma	xx (xx.x)	xx (xx.x)	xx (xx.x)	xx (xx.x)	xx (xx.x)	xx (xx.x)	xx (xx.x)	xx (xx.x)
Cutaneous Melanoma	xx (xx.x)	xx (xx.x)	xx (xx.x)	xx (xx.x)	xx (xx.x)	xx (xx.x)	xx (xx.x)	xx (xx.x)
Mucosal Melanoma	xx (xx.x)	xx (xx.x)	xx (xx.x)	xx (xx.x)	xx (xx.x)	xx (xx.x)	xx (xx.x)	xx (xx.x)
Other	xx (xx.x)	xx (xx.x)	xx (xx.x)	xx (xx.x)	xx (xx.x)	xx (xx.x)	xx (xx.x)	xx (xx.x)
Metastatic category at baseline (AJCC) for UM patients								
Mo								
M1								
M1a								
M1b								
M1c								
Stage at Initial Diagnosis								
Stage 0	xx (xx.x)	xx (xx.x)	xx (xx.x)	xx (xx.x)	xx (xx.x)	xx (xx.x)	xx (xx.x)	xx (xx.x)
Stage I	xx (xx.x)	xx (xx.x)	xx (xx.x)	xx (xx.x)	xx (xx.x)	xx (xx.x)	xx (xx.x)	xx (xx.x)

Stage 1A etc.	xx (xx.x)	xx (xx.x)	xx (xx.x)	xx (xx.x)	xx (xx.x)	xx (xx.x)	xx (xx.x)	xx (xx.x)
Time since initial diagnosis of primary site (months)								
N	xx	xx	xx	xx	xx	xx	xx	xx
Mean (SD)	xx (xx.x)	xx (xx.x)	xx (xx.x)	xx (xx.x)	xx (xx.x)	xx (xx.x)	xx (xx.x)	xx (xx.x)
Median	xx.x	xx.x	xx.x	xx.x	xx.x	xx.x	xx.x	xx.x
Min - Max	xx.x - xx.x	xx.x - xx.x	xx.x - xx.x	xx.x - xx.x	xx.x - xx.x	xx.x - xx.x	xx.x - xx.x	xx.x - xx.x
Time from initial diagnosis to first recurrence/relapse (months)								
N	xx	xx	xx	xx	xx	xx	xx	xx
Mean (SD)	xx (xx.x)	xx (xx.x)	xx (xx.x)	xx (xx.x)	xx (xx.x)	xx (xx.x)	xx (xx.x)	xx (xx.x)
Median	xx.x	xx.x	xx.x	xx.x	xx.x	xx.x	xx.x	xx.x
Min - Max	xx.x - xx.x	xx.x - xx.x	xx.x - xx.x	xx.x - xx.x	xx.x - xx.x	xx.x - xx.x	xx.x - xx.x	xx.x - xx.x
Time since most recent recurrence/relapse (months)								
N	xx	xx	xx	xx	xx	xx	xx	xx
Mean (SD)	xx (xx.x)	xx (xx.x)	xx (xx.x)	xx (xx.x)	xx (xx.x)	xx (xx.x)	xx (xx.x)	xx (xx.x)
Median	xx.x	xx.x	xx.x	xx.x	xx.x	xx.x	xx.x	xx.x
Min - Max	xx.x - xx.x	xx.x - xx.x	xx.x - xx.x	xx.x - xx.x	xx.x - xx.x	xx.x - xx.x	xx.x - xx.x	xx.x - xx.x
Histological Grade								
Well differentiated	xx (xx.x)	xx (xx.x)	xx (xx.x)	xx (xx.x)	xx (xx.x)	xx (xx.x)	xx (xx.x)	xx (xx.x)
Moderately differentiated	xx (xx.x)	xx (xx.x)	xx (xx.x)	xx (xx.x)	xx (xx.x)	xx (xx.x)	xx (xx.x)	xx (xx.x)
Poorly differentiated	xx (xx.x)	xx (xx.x)	xx (xx.x)	xx (xx.x)	xx (xx.x)	xx (xx.x)	xx (xx.x)	xx (xx.x)
Undifferentiated	xx (xx.x)	xx (xx.x)	xx (xx.x)	xx (xx.x)	xx (xx.x)	xx (xx.x)	xx (xx.x)	xx (xx.x)
Unknown	xx (xx.x)	xx (xx.x)	xx (xx.x)	xx (xx.x)	xx (xx.x)	xx (xx.x)	xx (xx.x)	xx (xx.x)
Stage at Time of Study Entry								

Stage 0	xx (xx.x)	xx (xx.x)	xx (xx.x)	xx (xx.x)	xx (xx.x)	xx (xx.x)	xx (xx.x)	xx (xx.x)
Stage I	xx (xx.x)	xx (xx.x)	xx (xx.x)	xx (xx.x)	xx (xx.x)	xx (xx.x)	xx (xx.x)	xx (xx.x)
Stage 1A etc.	xx (xx.x)	xx (xx.x)	xx (xx.x)	xx (xx.x)	xx (xx.x)	xx (xx.x)	xx (xx.x)	xx (xx.x)
Types of lesions at baseline-n (%)								
Target only	xx (xx.x)	xx (xx.x)	xx (xx.x)	xx (xx.x)	xx (xx.x)	xx (xx.x)	xx (xx.x)	xx (xx.x)
Non-target only	xx (xx.x)	xx (xx.x)	xx (xx.x)	xx (xx.x)	xx (xx.x)	xx (xx.x)	xx (xx.x)	xx (xx.x)
Both target and non-target	xx (xx.x)	xx (xx.x)	xx (xx.x)	xx (xx.x)	xx (xx.x)	xx (xx.x)	xx (xx.x)	xx (xx.x)
Unknown	xx (xx.x)	xx (xx.x)	xx (xx.x)	xx (xx.x)	xx (xx.x)	xx (xx.x)	xx (xx.x)	xx (xx.x)
Current extent of disease-n (%) (metastatic sites)								
Site 1	xx (xx.x)	xx (xx.x)	xx (xx.x)	xx (xx.x)	xx (xx.x)	xx (xx.x)	xx (xx.x)	xx (xx.x)
Site 2 etc.	xx (xx.x)	xx (xx.x)	xx (xx.x)	xx (xx.x)	xx (xx.x)	xx (xx.x)	xx (xx.x)	xx (xx.x)
Maximum target lesion diameter (mm)								
N	xx	xx	xx	xx	xx	xx	xx	xx
Mean (SD)	xx (xx.x)	xx (xx.x)	xx (xx.x)	xx (xx.x)	xx (xx.x)	xx (xx.x)	xx (xx.x)	xx (xx.x)
Median	xx.x	xx.x	xx.x	xx.x	xx.x	xx.x	xx.x	xx.x
Min-max	xx.x - xx.x	xx.x - xx.x	xx.x - xx.x	xx.x - xx.x	xx.x - xx.x	xx.x - xx.x	xx.x - xx.x	xx.x - xx.x

Percentages for metastatic category at baseline for UM patients will use the number of UM patients at baseline as the denominator.

Table 1-4 Prior antineoplastic therapy – Medication (Full analysis set)

Characteristic	4 mg/kg Q2W N=xx	8 mg/kg Q2W N=xx	12 mg/kg Q2W N=xx	16 mg/kg Q2W N=xx	24 mg/kg Q2W N=xx	12 mg/kg QW N=xx	16 mg/kg QW N=xx	All Subjects N=xx
Number of subjects with prior antineoplastic regimens-n (%)	xx (xx.x)	xx (xx.x)	xx (xx.x)	xx (xx.x)	xx (xx.x)	xx (xx.x)	xx (xx.x)	xx (xx.x)
Number of subjects with prior Darovarsetib	xx (xx.x)	xx (xx.x)	xx (xx.x)	xx (xx.x)	xx (xx.x)	xx (xx.x)	xx (xx.x)	xx (xx.x)
Number of subjects with prior Darovarsetib and Crizotinib	xx (xx.x)	xx (xx.x)	xx (xx.x)	xx (xx.x)	xx (xx.x)	xx (xx.x)	xx (xx.x)	xx (xx.x)
Number of subjects who received checkpoint inhibitors								
Number of subjects (%) who received PD-1 blockade +/- CTLA-4 blockade								
Number of subjects reported to have responded to any prior therapy								
Number of patients with LDH > 2x ULN at baseline								
AJCC M stage at baseline								
Largest tumor diameter at baseline								

Mean (SD)								
Median								
Q1-Q3								
Min-Max								
Number of regimens-n (%)								
0	xx (xx.x)	xx (xx.x)	xx (xx.x)	xx (xx.x)	xx (xx.x)	xx (xx.x)	xx (xx.x)	xx (xx.x)
1	xx (xx.x)	xx (xx.x)	xx (xx.x)	xx (xx.x)	xx (xx.x)	xx (xx.x)	xx (xx.x)	xx (xx.x)
2 etc	xx (xx.x)	xx (xx.x)	xx (xx.x)	xx (xx.x)	xx (xx.x)	xx (xx.x)	xx (xx.x)	xx (xx.x)
Missing	xx (xx.x)	xx (xx.x)	xx (xx.x)	xx (xx.x)	xx (xx.x)	xx (xx.x)	xx (xx.x)	xx (xx.x)
Therapy type for all treatments -n (%)								
Tebentafusp	xx (xx.x)	xx (xx.x)	xx (xx.x)	xx (xx.x)	xx (xx.x)	xx (xx.x)	xx (xx.x)	xx (xx.x)
IO	xx (xx.x)	xx (xx.x)	xx (xx.x)	xx (xx.x)	xx (xx.x)	xx (xx.x)	xx (xx.x)	xx (xx.x)
IO+TT	xx (xx.x)	xx (xx.x)	xx (xx.x)	xx (xx.x)	xx (xx.x)	xx (xx.x)	xx (xx.x)	xx (xx.x)
TT	xx (xx.x)	xx (xx.x)	xx (xx.x)	xx (xx.x)	xx (xx.x)	xx (xx.x)	xx (xx.x)	xx (xx.x)
Chemo	xx (xx.x)	xx (xx.x)	xx (xx.x)	xx (xx.x)	xx (xx.x)	xx (xx.x)	xx (xx.x)	xx (xx.x)
Unknown	xx (xx.x)	xx (xx.x)	xx (xx.x)	xx (xx.x)	xx (xx.x)	xx (xx.x)	xx (xx.x)	xx (xx.x)
Therapy type at last treatment -n (%)								
Tebentafusp	xx (xx.x)	xx (xx.x)	xx (xx.x)	xx (xx.x)	xx (xx.x)	xx (xx.x)	xx (xx.x)	xx (xx.x)
IO	xx (xx.x)	xx (xx.x)	xx (xx.x)	xx (xx.x)	xx (xx.x)	xx (xx.x)	xx (xx.x)	xx (xx.x)
IO+TT	xx (xx.x)	xx (xx.x)	xx (xx.x)	xx (xx.x)	xx (xx.x)	xx (xx.x)	xx (xx.x)	xx (xx.x)
TT	xx (xx.x)	xx (xx.x)	xx (xx.x)	xx (xx.x)	xx (xx.x)	xx (xx.x)	xx (xx.x)	xx (xx.x)
Chemo	xx (xx.x)	xx (xx.x)	xx (xx.x)	xx (xx.x)	xx (xx.x)	xx (xx.x)	xx (xx.x)	xx (xx.x)
Unknown	xx (xx.x)	xx (xx.x)	xx (xx.x)	xx (xx.x)	xx (xx.x)	xx (xx.x)	xx (xx.x)	xx (xx.x)
Setting at last treatment-n (%)								
Adjuvant	xx (xx.x)	xx (xx.x)	xx (xx.x)	xx (xx.x)	xx (xx.x)	xx (xx.x)	xx (xx.x)	xx (xx.x)

Neoadjuvant	xx (xx.x)	xx (xx.x)	xx (xx.x)	xx (xx.x)	xx (xx.x)	xx (xx.x)	xx (xx.x)	xx (xx.x)
Therapeutic	xx (xx.x)	xx (xx.x)	xx (xx.x)	xx (xx.x)	xx (xx.x)	xx (xx.x)	xx (xx.x)	xx (xx.x)
Missing	xx (xx.x)	xx (xx.x)	xx (xx.x)	xx (xx.x)	xx (xx.x)	xx (xx.x)	xx (xx.x)	xx (xx.x)
Best response at last treatment-n (%)								
Complete response (CR)	xx (xx.x)	xx (xx.x)	xx (xx.x)	xx (xx.x)	xx (xx.x)	xx (xx.x)	xx (xx.x)	xx (xx.x)
Partial response (PR)	xx (xx.x)	xx (xx.x)	xx (xx.x)	xx (xx.x)	xx (xx.x)	xx (xx.x)	xx (xx.x)	xx (xx.x)
Stable disease (SD)	xx (xx.x)	xx (xx.x)	xx (xx.x)	xx (xx.x)	xx (xx.x)	xx (xx.x)	xx (xx.x)	xx (xx.x)
Progression disease (PD)	xx (xx.x)	xx (xx.x)	xx (xx.x)	xx (xx.x)	xx (xx.x)	xx (xx.x)	xx (xx.x)	xx (xx.x)
Unknown	xx (xx.x)	xx (xx.x)	xx (xx.x)	xx (xx.x)	xx (xx.x)	xx (xx.x)	xx (xx.x)	xx (xx.x)
Not applicable	xx (xx.x)	xx (xx.x)	xx (xx.x)	xx (xx.x)	xx (xx.x)	xx (xx.x)	xx (xx.x)	xx (xx.x)
Missing	xx (xx.x)	xx (xx.x)	xx (xx.x)	xx (xx.x)	xx (xx.x)	xx (xx.x)	xx (xx.x)	xx (xx.x)
Time from last treatment to Progression (<unit>)								
n	xxx	xxx	xxx	xxx	xxx	xxx	xxx	xxx
Mean (SD)	xx.x (x.xx)	xx.x (x.xx)	xx.x (x.xx)	xx.x (x.xx)	xx.x (x.xx)	xx.x (x.xx)	xx.x (x.xx)	xx.x (x.xx)
Median	xx.x	xx.x	xx.x	xx.x	xx.x	xx.x	xx.x	xx.x
Q1-Q3	xx.x-xx.x	xx.x-xx.x	xx.x-xx.x	xx.x-xx.x	xx.x-xx.x	xx.x-xx.x	xx.x-xx.x	xx.x-xx.x
Min-Max	xx.x-xx.x	xx.x-xx.x	xx.x-xx.x	xx.x-xx.x	xx.x-xx.x	xx.x-xx.x	xx.x-xx.x	xx.x-xx.x
Duration of last response (<unit>)								
n	xxx	xxx	xxx	xxx	xxx	xxx	xxx	xxx
Mean (SD)	xx.x (x.xx)	xx.x (x.xx)	xx.x (x.xx)	xx.x (x.xx)	xx.x (x.xx)	xx.x (x.xx)	xx.x (x.xx)	xx.x (x.xx)
Median	xx.x	xx.x	xx.x	xx.x	xx.x	xx.x	xx.x	xx.x
Q1-Q3	xx.x-xx.x	xx.x-xx.x	xx.x-xx.x	xx.x-xx.x	xx.x-xx.x	xx.x-xx.x	xx.x-xx.x	xx.x-xx.x
Min-Max	xx.x-xx.x	xx.x-xx.x	xx.x-xx.x	xx.x-xx.x	xx.x-xx.x	xx.x-xx.x	xx.x-xx.x	xx.x-xx.x

Reason for discontinuation at last medication								
<reason 1>	xxx	xxx	xxx	xxx	xxx	xxx	xxx	xxx
<reason 2 etc.>	xxx	xxx	xxx	xxx	xxx	xxx	xxx	xxx

Programming Note:

1. *STL program: T_DMG001*
2. *Therapy type and sub-categories are not reported in CRF. TP to provide list of prior therapy for ML to review and classify.*

Table 1-5 Duration of exposure to DYP688 (Safety set)

	4 mg/kg Q2W N=xx	8 mg/kg Q2W N=xx	12 mg/kg Q2W N=xx	16 mg/kg Q2W N=xx	24 mg/kg Q2W N=xx	12 mg/kg QW N=xx	16 mg/kg QW N=xx	All Subjects N=xx
Total number of subjects receiving DYP688-n (%)	xx (xx.x)	xx (xx.x)	xx (xx.x)	xx (xx.x)	xx (xx.x)	xx (xx.x)	xx (xx.x)	xx (xx.x)
Duration of exposure (months)								
Mean (SD)	xx.x (x.xx)	xx.x (x.xx)	xx.x (x.xx)	xx.x (x.xx)	xx.x (x.xx)	xx.x (x.xx)	xx.x (x.xx)	xx.x (x.xx)
Median	xx.x	xx.x	xx.x	xx.x	xx.x	xx.x	xx.x	xx.x
Q1-Q3	xx.x-xx.x	xx.x-xx.x	xx.x-xx.x	xx.x-xx.x	xx.x-xx.x	xx.x-xx.x	xx.x-xx.x	xx.x-xx.x
Min-Max	xx-xx	xx-xx	xx-xx	xx-xx	xx-xx	xx-xx	xx-xx	xx-xx
Duration of exposure categories-n (%)								
< 3 months	xx (xx.x)	xx (xx.x)	xx (xx.x)	xx (xx.x)	xx (xx.x)	xx (xx.x)	xx (xx.x)	xx (xx.x)
3- < 6 months	xx (xx.x)	xx (xx.x)	xx (xx.x)	xx (xx.x)	xx (xx.x)	xx (xx.x)	xx (xx.x)	xx (xx.x)
6- < 9 months	xx (xx.x)	xx (xx.x)	xx (xx.x)	xx (xx.x)	xx (xx.x)	xx (xx.x)	xx (xx.x)	xx (xx.x)
9- < 12 months	xx (xx.x)	xx (xx.x)	xx (xx.x)	xx (xx.x)	xx (xx.x)	xx (xx.x)	xx (xx.x)	xx (xx.x)
12- <18 months	xx (xx.x)	xx (xx.x)	xx (xx.x)	xx (xx.x)	xx (xx.x)	xx (xx.x)	xx (xx.x)	xx (xx.x)
>18 months	xx (xx.x)	xx (xx.x)	xx (xx.x)	xx (xx.x)	xx (xx.x)	xx (xx.x)	xx (xx.x)	xx (xx.x)

<Subject-time> is the sum of each subject's treatment exposure in <unit>.

Table 1-6 Dose of DYP688 received (Safety set)

	4 mg/kg Q2W N=xx	8 mg/kg Q2W N=xx	12 mg/kg Q2W N=xx	16 mg/kg Q2W N=xx	24 mg/kg Q2W N=xx	12 mg/kg QW N=xx	16 mg/kg QW N=xx	All Subjects N=xx
Total number of subjects receiving DYP688-n (%)	**xx (xx.x)**	**xx (xx.x)**	**xx (xx.x)**	**xx (xx.x)**	**xx (xx.x)**	**xx (xx.x)**	**xx (xx.x)**	**xx (xx.x)**
Cumulative dose (mg/kg)								
Mean (SD)	xx.x (x.xx)	xx.x (x.xx)	xx.x (x.xx)	xx.x (x.xx)	xx.x (x.xx)	xx.x (x.xx)	xx.x (x.xx)	xx.x (x.xx)
Median	xx.x	xx.x	xx.x	xx.x	xx.x	xx.x	xx.x	xx.x
Q1-Q3	xx.x-xx.x	xx.x-xx.x	xx.x-xx.x	xx.x-xx.x	xx.x-xx.x	xx.x-xx.x	xx.x-xx.x	xx.x-xx.x
Min-Max	xx-xx	xx-xx	xx-xx	xx-xx	xx-xx	xx-xx	xx-xx	xx-xx
Dose intensity (mg/kg/cycle)								
Mean (SD)	xx.x (x.xx)	xx.x (x.xx)	xx.x (x.xx)	xx.x (x.xx)	xx.x (x.xx)	xx.x (x.xx)	xx.x (x.xx)	xx.x (x.xx)
Median	xx.x	xx.x	xx.x	xx.x	xx.x	xx.x	xx.x	xx.x
Q1-Q3	xx.x-xx.x	xx.x-xx.x	xx.x-xx.x	xx.x-xx.x	xx.x-xx.x	xx.x-xx.x	xx.x-xx.x	xx.x-xx.x
Min-Max	xx-xx	xx-xx	xx-xx	xx-xx	xx-xx	xx-xx	xx-xx	xx-xx
Relative dose intensity (%)								
Mean (SD)	xx.x (x.xx)	xx.x (x.xx)	xx.x (x.xx)	xx.x (x.xx)	xx.x (x.xx)	xx.x (x.xx)	xx.x (x.xx)	xx.x (x.xx)
Median	xx.x	xx.x	xx.x	xx.x	xx.x	xx.x	xx.x	xx.x
Q1-Q3	xx.x-xx.x	xx.x-xx.x	xx.x-xx.x	xx.x-xx.x	xx.x-xx.x	xx.x-xx.x	xx.x-xx.x	xx.x-xx.x
Min-Max	xx-xx	xx-xx	xx-xx	xx-xx	xx-xx	xx-xx	xx-xx	xx-xx
Relative dose intensity categories-n (%)								

<50%	xx (xx.x)	xx (xx.x)	xx (xx.x)	xx (xx.x)	xx (xx.x)	xx (xx.x)	xx (xx.x)	xx (xx.x)
>=50-75%	xx (xx.x)	xx (xx.x)	xx (xx.x)	xx (xx.x)	xx (xx.x)	xx (xx.x)	xx (xx.x)	xx (xx.x)
>=75-90%	xx (xx.x)	xx (xx.x)	xx (xx.x)	xx (xx.x)	xx (xx.x)	xx (xx.x)	xx (xx.x)	xx (xx.x)
>=90-110%	xx (xx.x)	xx (xx.x)	xx (xx.x)	xx (xx.x)	xx (xx.x)	xx (xx.x)	xx (xx.x)	xx (xx.x)
>=110%	xx (xx.x)	xx (xx.x)	xx (xx.x)	xx (xx.x)	xx (xx.x)	xx (xx.x)	xx (xx.x)	xx (xx.x)

Average dose does not consider drug free days, whereas dose intensity and relative dose intensity include days of zero dose in the calculation.

Table 1-7 Overview of adverse events (Safety set)

	Treatment A N=xxx		Treatment B N=xxx		Treatment C N=xxx		…….. N=xxx		All Subjects N=xxx	
**Category**	**All grades n (%)**	**Grade >=3 n (%)**	**All grades n (%)**	**Grade >=3 n (%)**	**All grades n (%)**	**Grade >=3 n (%)**	**All grades n (%)**	**Grade >=3 n (%)**	**All grades n (%)**	**Grade >=3 n (%)**
Adverse events	xx (xx.x)	xx (xx.x)	xx (xx.x)	xx (xx.x)	xx (xx.x)	xx (xx.x)	xx (xx.x)	xx (xx.x)	xx (xx.x)	xx (xx.x)
Treatment-related	xx (xx.x)	xx (xx.x)	xx (xx.x)	xx (xx.x)	xx (xx.x)	xx (xx.x)	xx (xx.x)	xx (xx.x)	xx (xx.x)	xx (xx.x)
SAEs	xx (xx.x)	xx (xx.x)	xx (xx.x)	xx (xx.x)	xx (xx.x)	xx (xx.x)	xx (xx.x)	xx (xx.x)	xx (xx.x)	xx (xx.x)
Treatment-related	xx (xx.x)	xx (xx.x)	xx (xx.x)	xx (xx.x)	xx (xx.x)	xx (xx.x)	xx (xx.x)	xx (xx.x)	xx (xx.x)	xx (xx.x)
Fatal SAEs	xx (xx.x)	xx (xx.x)	xx (xx.x)	xx (xx.x)	xx (xx.x)	xx (xx.x)	xx (xx.x)	xx (xx.x)	xx (xx.x)	xx (xx.x)
Treatment-related	xx (xx.x)	xx (xx.x)	xx (xx.x)	xx (xx.x)	xx (xx.x)	xx (xx.x)	xx (xx.x)	xx (xx.x)	xx (xx.x)	xx (xx.x)
AEs leading to treatment discontinuation	xx (xx.x)	xx (xx.x)	xx (xx.x)	xx (xx.x)	xx (xx.x)	xx (xx.x)	xx (xx.x)	xx (xx.x)	xx (xx.x)	xx (xx.x)
Treatment-related	xx (xx.x)	xx (xx.x)	xx (xx.x)	xx (xx.x)	xx (xx.x)	xx (xx.x)	xx (xx.x)	xx (xx.x)	xx (xx.x)	xx (xx.x)
AEs leading to dose adjustment/interruption	xx (xx.x)	xx (xx.x)	xx (xx.x)	xx (xx.x)	xx (xx.x)	xx (xx.x)	xx (xx.x)	xx (xx.x)	xx (xx.x)	xx (xx.x)
AEs requiring additional therapy	xx (xx.x)	xx (xx.x)	xx (xx.x)	xx (xx.x)	xx (xx.x)	xx (xx.x)	xx (xx.x)	xx (xx.x)	xx (xx.x)	xx (xx.x)
DLT	xx (xx.x)	xx (xx.x)	xx (xx.x)	xx (xx.x)	xx (xx.x)	xx (xx.x)	xx (xx.x)	xx (xx.x)	xx (xx.x)	xx (xx.x)

Numbers (n) represent counts of subjects.
A subject with multiple severity grades for an AE is only counted under the maximum grade.
MedDRA version <MedDRAVers>, CTCAE version <CTCVers>.

Note: use treatment group label 1

Programming Note:

Overview table providing high-level overview from several different AE tables.

The categories presented in this table should correspond to the types of AE tables that have been generated for the study (i.e. AEs, SAEs, AEs leading to discontinuation….)

In NovDD studies information for line "fatal SAEs" and "treatment related fatal SAEs" is taken from the EudraCT outputs (Table T_AE_320) or death listing (L_AE_301). If it can't be programmed, it can be added manually at the writing stage.

Table 1-8 Adverse events by preferred term and treatment group in dose escalation (Safety set)

	Treatment A N=xxx		Treatment B N=xxx		Treatment C N=xxx		……. N=xxx		All subjects N=xxx	
**Preferred term**	**All grades n (%)**	**≥ Grade 3 n (%)**	**All grades n (%)**	**≥ Grade 3 n (%)**	**All grades n (%)**	**≥ Grade 3 n (%)**	**All grades n (%)**	**≥ Grade 3 n (%)**	**All grades n (%)**	**≥ Grade 3 n (%)**
Number of subjects with at least one event	xxx (xx.x)	xxx (xx.x)	xxx (xx.x)	xxx (xx.x)	xxx (xx.x)	xxx (xx.x)	xxx (xx.x)	xxx (xx.x)	xxx (xx.x)	xxx (xx.x)
Fatigue	xxx (xx.x)	xxx (xx.x)	xxx (xx.x)	xxx (xx.x)	xxx (xx.x)	xxx (xx.x)	xxx (xx.x)	xxx (xx.x)	xxx (xx.x)	xxx (xx.x)
Nausea	xxx (xx.x)	xxx (xx.x)	xxx (xx.x)	xxx (xx.x)	xxx (xx.x)	xxx (xx.x)	xxx (xx.x)	xxx (xx.x)	xxx (xx.x)	xxx (xx.x)
Anaemia	xxx (xx.x)	xxx (xx.x)	xxx (xx.x)	xxx (xx.x)	xxx (xx.x)	xxx (xx.x)	xxx (xx.x)	xxx (xx.x)	xxx (xx.x)	xxx (xx.x)
Diarrhoea	xxx (xx.x)	xxx (xx.x)	xxx (xx.x)	xxx (xx.x)	xxx (xx.x)	xxx (xx.x)	xxx (xx.x)	xxx (xx.x)	xxx (xx.x)	xxx (xx.x)
Vomiting	xxx (xx.x)	xxx (xx.x)	xxx (xx.x)	xxx (xx.x)	xxx (xx.x)	xxx (xx.x)	xxx (xx.x)	xxx (xx.x)	xxx (xx.x)	xxx (xx.x)
Constipation	xxx (xx.x)	xxx (xx.x)	xxx (xx.x)	xxx (xx.x)	xxx (xx.x)	xxx (xx.x)	xxx (xx.x)	xxx (xx.x)	xxx (xx.x)	xxx (xx.x)
Pyrexia	xxx (xx.x)	xxx (xx.x)	xxx (xx.x)	xxx (xx.x)	xxx (xx.x)	xxx (xx.x)	xxx (xx.x)	xxx (xx.x)	xxx (xx.x)	xxx (xx.x)
Urinary tract infection	xxx (xx.x)	xxx (xx.x)	xxx (xx.x)	xxx (xx.x)	xxx (xx.x)	xxx (xx.x)	xxx (xx.x)	xxx (xx.x)	xxx (xx.x)	xxx (xx.x)
Injection site reaction	xxx (xx.x)	xxx (xx.x)	xxx (xx.x)	xxx (xx.x)	xxx (xx.x)	xxx (xx.x)	xxx (xx.x)	xxx (xx.x)	xxx (xx.x)	xxx (xx.x)
Thrombocytopenia	xxx (xx.x)	xxx (xx.x)	xxx (xx.x)	xxx (xx.x)	xxx (xx.x)	xxx (xx.x)	xxx (xx.x)	xxx (xx.x)	xxx (xx.x)	xxx (xx.x)
Anorexia	xxx (xx.x)	xxx (xx.x)	xxx (xx.x)	xxx (xx.x)	xxx (xx.x)	xxx (xx.x)	xxx (xx.x)	xxx (xx.x)	xxx (xx.x)	xxx (xx.x)
Dyspnoea	xxx (xx.x)	xxx (xx.x)	xxx (xx.x)	xxx (xx.x)	xxx (xx.x)	xxx (xx.x)	xxx (xx.x)	xxx (xx.x)	xxx (xx.x)	xxx (xx.x)
Stomatitis	xxx (xx.x)	xxx (xx.x)	xxx (xx.x)	xxx (xx.x)	xxx (xx.x)	xxx (xx.x)	xxx (xx.x)	xxx (xx.x)	xxx (xx.x)	xxx (xx.x)

Note: use treatment group label 1

Programming Note:

OSO reference T_AE_304X

Sort by descending frequency in All grades, Investigational drug column of highest relevance.

Include only on-treatment events (as defined in SAP). 'All grades' includes any AEs with missing grade.

Table 1-9 Adverse events, suspected to be study treatment related, by preferred term and treatment group in dose escalation (Safety set)

Please use the same shell as Table 1-8

Table 1-10 Adverse events, suspected to be treated related, by preferred term, maximum grade and treatment group in dose escalation (Safety set)

Preferred term<br>Maximum grade	Treatment A<br>N=xxx<br>n (%)	Treatment B<br>N=xxx<br>n (%)	………<br>N=xxx<br>n (%)	All subjects<br>N=xxx<br>n (%)
Number of subjects with at least one event	xxx (xx.x)	xxx (xx.x)	xxx (xx.x)	xxx (xx.x)
Grade 1	xxx (xx.x)	xxx (xx.x)	xxx (xx.x)	xxx (xx.x)
Grade 2	xxx (xx.x)	xxx (xx.x)	xxx (xx.x)	xxx (xx.x)
Grade 3	xxx (xx.x)	xxx (xx.x)	xxx (xx.x)	xxx (xx.x)
Grade 4	xxx (xx.x)	xxx (xx.x)	xxx (xx.x)	xxx (xx.x)
Grade 5	xxx (xx.x)	xxx (xx.x)	xxx (xx.x)	xxx (xx.x)
Missing	xxx (xx.x)	xxx (xx.x)	xxx (xx.x)	xxx (xx.x)
Anaemia	xxx (xx.x)	xxx (xx.x)	xxx (xx.x)	xxx (xx.x)
Grade 1	xxx (xx.x)	xxx (xx.x)	xxx (xx.x)	xxx (xx.x)
Grade 2	xxx (xx.x)	xxx (xx.x)	xxx (xx.x)	xxx (xx.x)
Grade 3	xxx (xx.x)	xxx (xx.x)	xxx (xx.x)	xxx (xx.x)
Grade 4	xxx (xx.x)	xxx (xx.x)	xxx (xx.x)	xxx (xx.x)
Grade 5	xxx (xx.x)	xxx (xx.x)	xxx (xx.x)	xxx (xx.x)
Missing	xxx (xx.x)	xxx (xx.x)	xxx (xx.x)	xxx (xx.x)

Numbers (n) represent counts of subjects.
A subject with multiple severity grades for an AE is only counted under the maximum grade.
MedDRA version <xx.x>, CTCAE version <x.xx>.

Programming Note: OSO reference T_AE_308

Note: use treatment group label 1

Table 1-11 Serious adverse events, by system organ class, preferred term, maximum grade and treatment in dose escalation (Safety set)

Primary system organ class<br>Preferred term<br>Maximum grade	Treatment A<br>N=xxx<br>n (%)	Treatment B<br>N=xxx<br>n (%)	…..<br>N=xxx<br>n (%)	All subjects<br>N=xxx<br>n (%)
Number of subjects with at least one event	xxx (xx.x)	xxx (xx.x)	xxx (xx.x)	xxx (xx.x)
Grade 1	xxx (xx.x)	xxx (xx.x)	xxx (xx.x)	xxx (xx.x)
Grade 2	xxx (xx.x)	xxx (xx.x)	xxx (xx.x)	xxx (xx.x)
Grade 3	xxx (xx.x)	xxx (xx.x)	xxx (xx.x)	xxx (xx.x)
Grade 4	xxx (xx.x)	xxx (xx.x)	xxx (xx.x)	xxx (xx.x)
Grade 5	xxx (xx.x)	xxx (xx.x)	xxx (xx.x)	xxx (xx.x)
Missing	xxx (xx.x)	xxx (xx.x)	xxx (xx.x)	xxx (xx.x)
Blood and lymphatic system disorders	xxx (xx.x)	xxx (xx.x)	xxx (xx.x)	xxx (xx.x)
Grade 1	xxx (xx.x)	xxx (xx.x)	xxx (xx.x)	xxx (xx.x)
Grade 2	xxx (xx.x)	xxx (xx.x)	xxx (xx.x)	xxx (xx.x)
Grade 3	xxx (xx.x)	xxx (xx.x)	xxx (xx.x)	xxx (xx.x)
Grade 4	xxx (xx.x)	xxx (xx.x)	xxx (xx.x)	xxx (xx.x)
Grade 5	xxx (xx.x)	xxx (xx.x)	xxx (xx.x)	xxx (xx.x)
Missing	xxx (xx.x)	xxx (xx.x)	xxx (xx.x)	xxx (xx.x)
Anaemia	xxx (xx.x)	xxx (xx.x)	xxx (xx.x)	xxx (xx.x)
Grade 1	xxx (xx.x)	xxx (xx.x)	xxx (xx.x)	xxx (xx.x)
Grade 2	xxx (xx.x)	xxx (xx.x)	xxx (xx.x)	xxx (xx.x)
Grade 3	xxx (xx.x)	xxx (xx.x)	xxx (xx.x)	xxx (xx.x)
Grade 4	xxx (xx.x)	xxx (xx.x)	xxx (xx.x)	xxx (xx.x)
Grade 5	xxx (xx.x)	xxx (xx.x)	xxx (xx.x)	xxx (xx.x)
Missing	xxx (xx.x)	xxx (xx.x)	xxx (xx.x)	xxx (xx.x)

Numbers (n) represent counts of subjects.
A subject with multiple severity grades for an AE is only counted under the maximum grade.
MedDRA version <xx.x>, CTCAE version <x.xx>.

Note: use treatment group label 1

Table 1-12 Suspected serious adverse events, by system organ class, preferred term, maximum grade and treatment (Safety set)

Please use the same shell as Table 1-11

Table 1-13 Best overall response* summary (Full analysis set)

	Treatment A N=xx n (%)	Treatment B N=xx n (%)	Treatment C N=xx n (%)	…….. N=xx n (%)	All subjectsN=xx n (%)
Number of evaluable patients	X	X	X	X	X
Subject with measurable disease at baseline	xx (xx.x)	xx (xx.x)	xx (xx.x)	xx (xx.x)	xx (xx.x)
Subject with non-measurable disease only at baseline	xx (xx.x)	xx (xx.x)	xx (xx.x)	xx (xx.x)	xx (xx.x)
Best overall response - n (%)					
Complete Response (CR)	xx (xx.x)	xx (xx.x)	xx (xx.x)	xx (xx.x)	xx (xx.x)
Partial Response (PR)	xx (xx.x)	xx (xx.x)	xx (xx.x)	xx (xx.x)	xx (xx.x)
Stable Disease (SD)	xx (xx.x)	xx (xx.x)	xx (xx.x)	xx (xx.x)	xx (xx.x)
Progressive Disease (PD)	xx (xx.x)	xx (xx.x)	xx (xx.x)	xx (xx.x)	xx (xx.x)
Not Evaluable (NE)	xx (xx.x)	xx (xx.x)	xx (xx.x)	xx (xx.x)	xx (xx.x)
Overall Response Rate (ORR: CR+PR)	xx (xx.x)	xx (xx.x)	xx (xx.x)	xx (xx.x)	xx (xx.x)
95% CI	(x.x, x.x)	(x.x, x.x)	(x.x, x.x)	(x.x, x.x)	(x.x, x.x)
Disease Control Rate (DCR: CR+PR+SD)	x (xx.x)	x (xx.x)	x (xx.x)	x (xx.x)	x (xx.x)
95% CI	(x.x,x.x)	(x.x,x.x)	(x.x,x.x)	(x.x,x.x)	(x.x,x.x)

	Treatment A N=xx n (%)	Treatment B N=xx n (%)	Treatment C N=xx n (%)	…….. N=xx n (%)	All subjectsN=xx n (%)
*by local radiology review					

n: the number of patients from each response category available by cut off date
m: the number of evaluable patients by the cut-off; evaluable patients are defined as having at least one post-baseline tumor assessment or discontinued treatment but no post-baseline tumor assessment before cut-off date
N: the total number of patients enrolled

The 95% CI for the frequency distribution of each variable were computed using Clopper-Pearson.

Note: use treatment group label 1

Programming Note:

Adjust the optional columns and/or rows for use in TCO/FD.

For the columns of the difference between treatments and p-value, the following options are available: a) not to display the columns, b) display all, or c) display selected entries from the columns.

An All subjects column can be added for studies with one treatment in different arms/groups (e.g. dose escalation phase).

Table 1-14 Time to PFS endpoint by treatment group (Full analysis set)

	Treatment A<br>N=xx	Treatment B<br>N=xx	All subjects<br>N=xx
No. of PFS events n (%)	xx (xx.x)	xx (xx.x)	xx (xx.x)
Progression	xx (xx.x)	xx (xx.x)	xx (xx.x)
Death	xx (xx.x)	xx (xx.x)	xx (xx.x)
No. Of censored	xx (xx.x)	xx (xx.x)	xx (xx.x)
PFS [months], Percentiles (95% CI)			
25th	xx.x (xx.x, xx.x)	xx.x (xx.x, xx.x)	xx.x (xx.x, xx.x)
50th	xx.x (xx.x, xx.x)	xx.x (xx.x, xx.x)	xx.x (xx.x, xx.x)
75th	xx.x (xx.x, xx.x)	xx.x (xx.x, xx.x)	xx.x (xx.x, xx.x)
KM estimates of PFS, (95% CI)			
4 months	xx.x (xx.x, xx.x)	xx.x (xx.x, xx.x)	xx.x (xx.x, xx.x)
6 months	xx.x (xx.x, xx.x)	xx.x (xx.x, xx.x)	xx.x (xx.x, xx.x)
9 months	xx.x (xx.x, xx.x)	xx.x (xx.x, xx.x)	xx.x (xx.x, xx.x)
12 months	xx.x (xx.x, xx.x)	xx.x (xx.x, xx.x)	xx.x (xx.x, xx.x)

Events are the PFS events.

PFS = (Date of event or censoring – Date of treatment start + 1)/30.4375 (months) regardless of censoring. The date of censoring is defined as the last assessment date for PFS.

Table 1-15 Duration of response (DOR) endpoint by treatment group (Full analysis set - Patients with CR and PR)

	Treatment A N*=xx	Treatment B N*=xx	All subjects N*=xx
Number of subjects-n (%)			
with events (1)	xx (xx.x)	xx (xx.x)	xx (xx.x)
with censorings	xx (xx.x)	xx (xx.x)	xx (xx.x)
DOR [months], Percentiles (95% CI)			
25th	xx.x (xx.x, xx.x)	xx.x (xx.x, xx.x)	xx.x (xx.x, xx.x)
50th	xx.x (xx.x, xx.x)	xx.x (xx.x, xx.x)	xx.x (xx.x, xx.x)
75th	xx.x (xx.x, xx.x)	xx.x (xx.x, xx.x)	xx.x (xx.x, xx.x)
KM estimates of DOR (95% CI)			
2 months	xx.x (xx.x, xx.x)	xx.x (xx.x, xx.x)	xx.x (xx.x, xx.x)
4 months	xx.x (xx.x, xx.x)	xx.x (xx.x, xx.x)	xx.x (xx.x, xx.x)
6 months	xx.x (xx.x, xx.x)	xx.x (xx.x, xx.x)	xx.x (xx.x, xx.x)

N* is the number of patients with the best overall response CR or PR in each treatment group.

(1) Events are the DOR events.

DOR = (Date of event or censoring – Date of treatment start + 1)/30.4375 (months) regardless of censoring. Date of censoring is defined as the last assessment date for DOR.

Figure 1-1 Waterfall plot for best percentage change from baseline in sum of longest diameters based on local radiology review (Full analysis set)

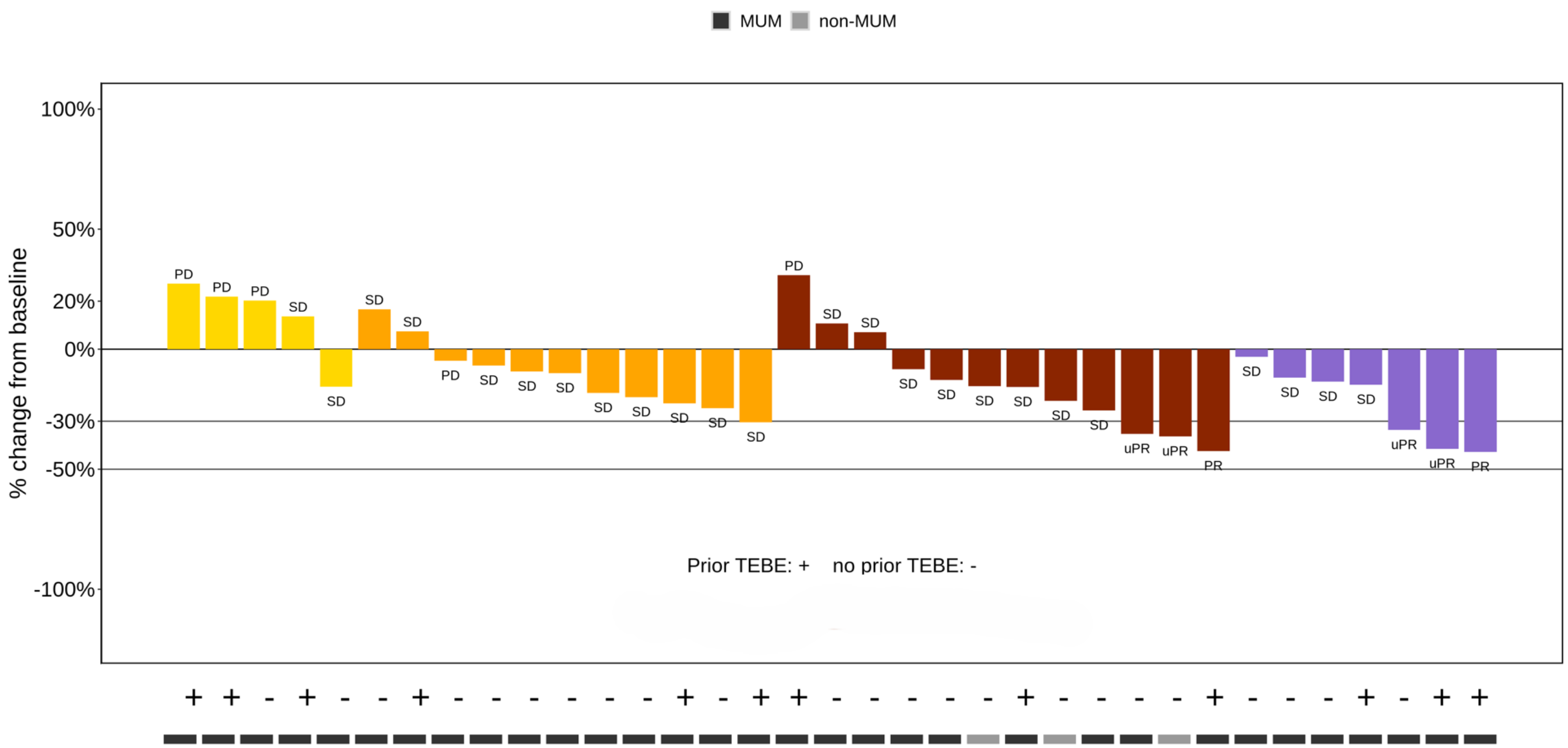

Programming notes:
Y axis: % change from baseline
legend title: Best overall response
Remove the 20% increase line.
Black and white square to denote pts who are non-MUM vs MUM
+ sign for those pts who received prior tebe
Colors for dosing cohort
GNAQ and 11 mutations (if available)
Developed and validated by Stats line function

Figure 1-2 Overall responses and percentage changes from baseline of sum of longest diameters of targeted lesions at each timepoint (Full analysis set)

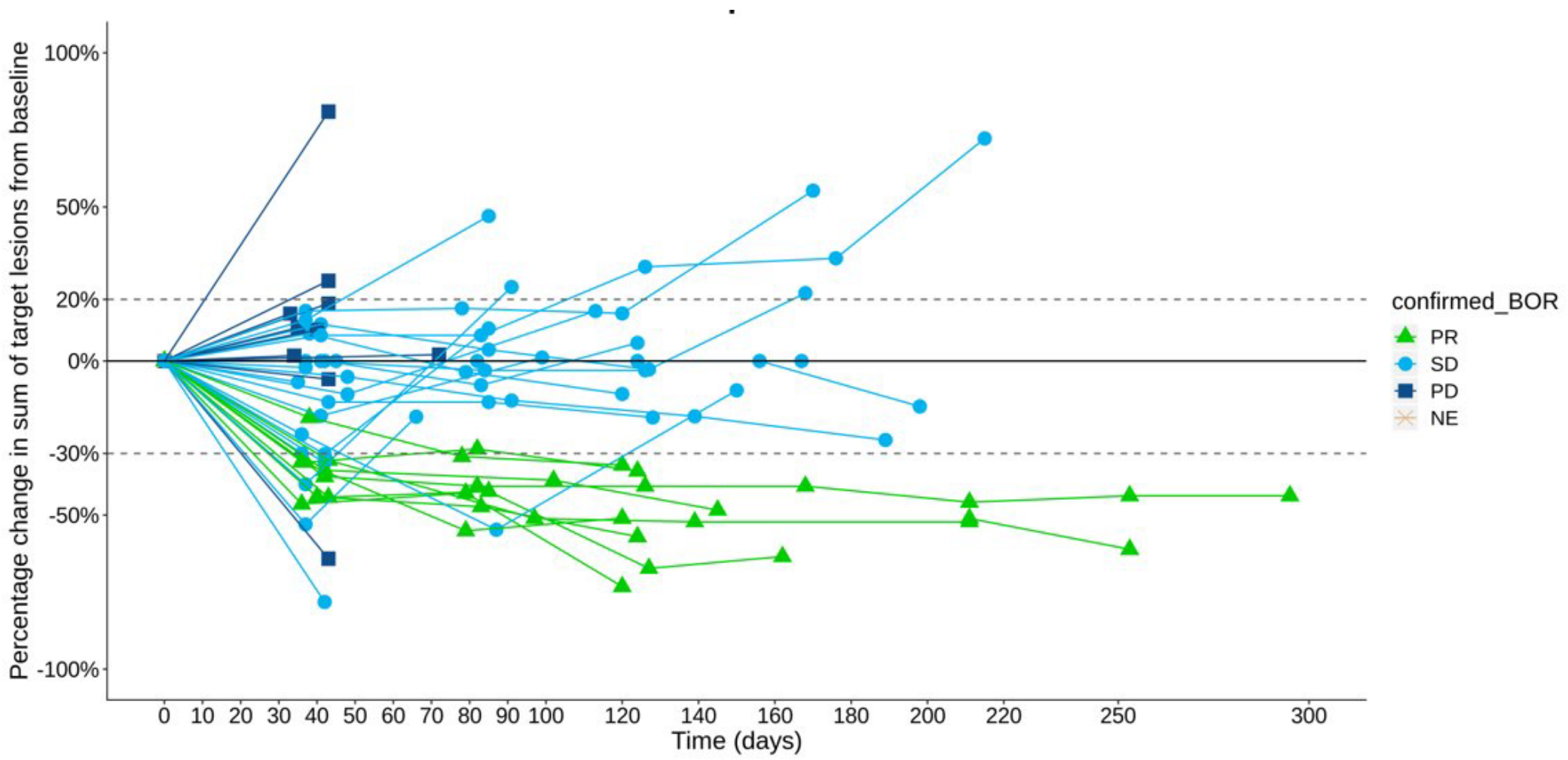

Programming notes:
Developed and validated by Stats line function

Figure 1-3 Time on study drug and duration of response (Full analysis set)

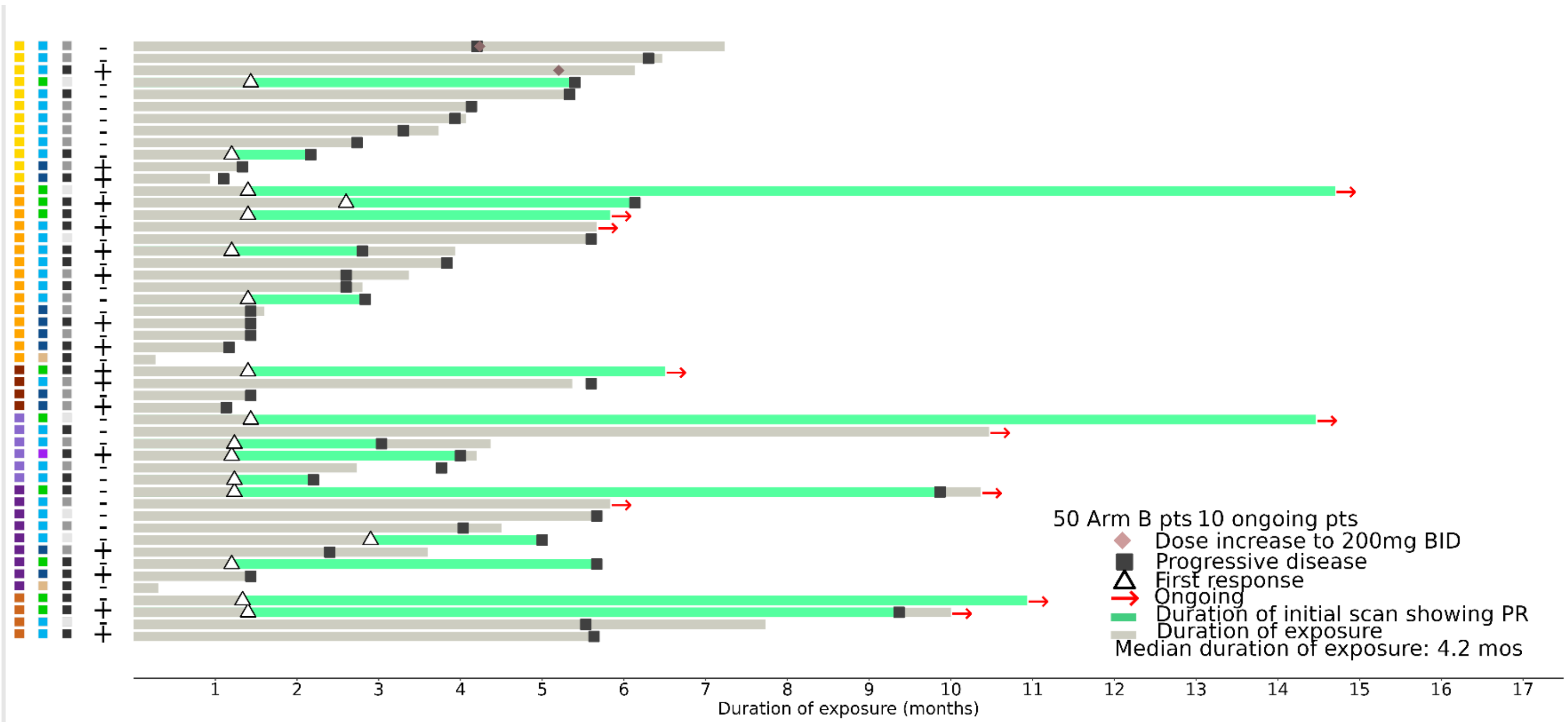

Programming notes:
Black and white square to denote pts who are non-MUM vs MUM
+ sign for those pts who received prior tebe
Colors for dosing cohort
GNAQ and 11 mutations (if available)
Colors for confirmed BOR
Developed and validated by Stats line function

Figure 1-4 Tornado plot (Full analysis set)

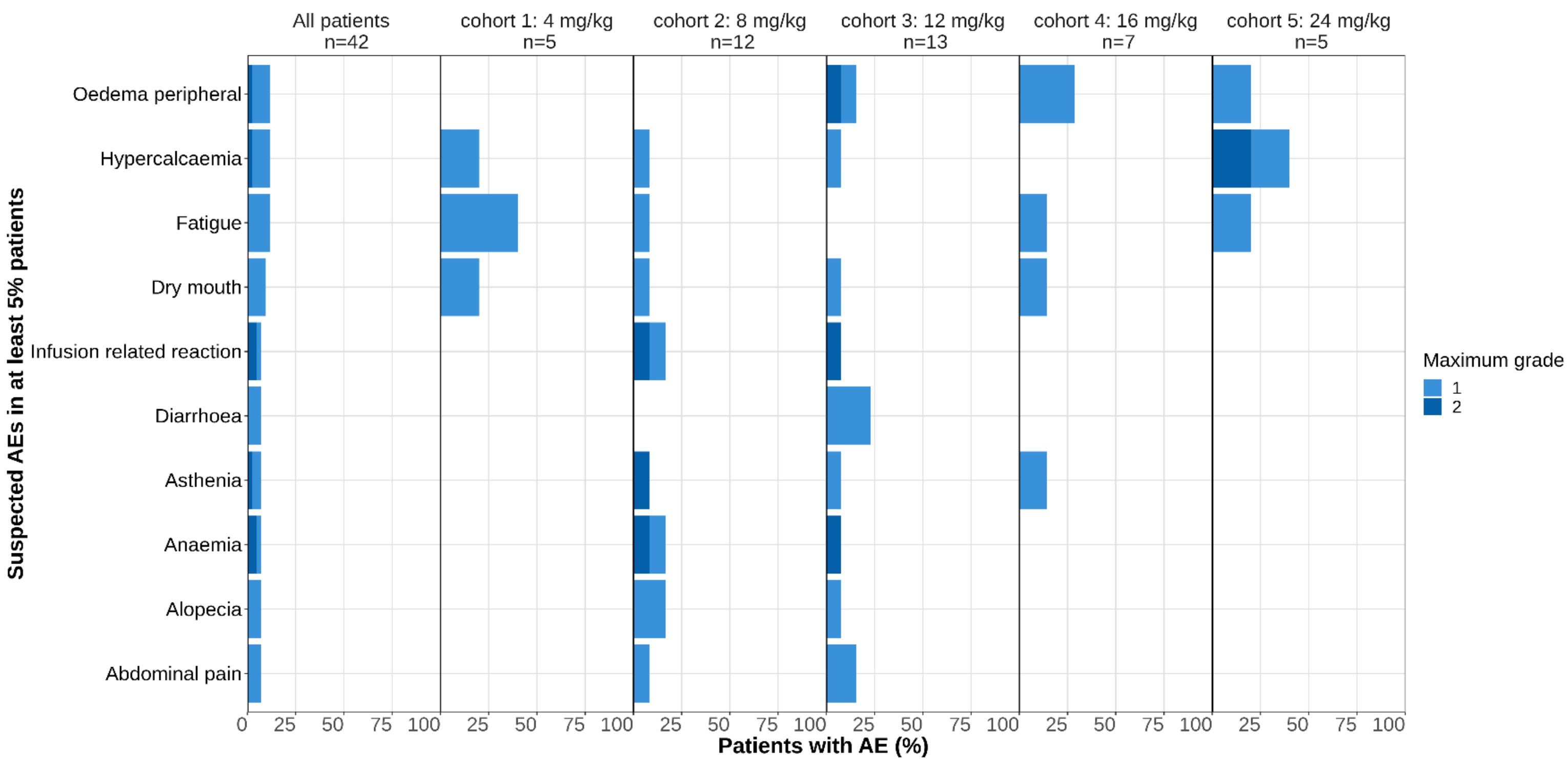

Programming notes:
Developed and validated by Stats line function

1.8 Data panels to be cleaned (RAVE)

- Disposition

- Demographics
- AE
- Death
- DAR / Study treatment infusion
- Visit and subject status
- RECIST (Image log, target lesion, non-target lesion, response assessment and new lesion assessment)
- Diagnosis and extent of cancer
- Prior medications
- Local lab data (heme, chem and thyroid)
- ECOG
- Vital signs (weight only)
