# Supplementary material for: An anti-PMEL antibody−drug conjugate with a Gq/11 inhibitor payload in GNAQ/GNA11-mutant melanomas: a phase 1 trial
Source: Nat Med. 2026 Jul 13;32(9):3248–58. doi: 10.1038/s41591-026-04518-z (PMC13577922; doi:10.1038/s41591-026-04518-z)
Supplement: Supplementary file 2 [file 41591_2026_4518_MOESM2_ESM.pdf]

nature portfolio

Corresponding author(s): Dr. Carlino

Last updated by author(s): Jun 10, 2026

Reporting Summary

Nature Portfolio wishes to improve the reproducibility of the work that we publish. This form provides structure for consistency and transparency in reporting. For further information on Nature Portfolio policies, see our Editorial Policies and the Editorial Policy Checklist.

Statistics

For all statistical analyses, confirm that the following items are present in the figure legend, table legend, main text, or Methods section.

n/a	Confirmed	
☐	☒	The exact sample size (n) for each experimental group/condition, given as a discrete number and unit of measurement
☒	☐	A statement on whether measurements were taken from distinct samples or whether the same sample was measured repeatedly
☐	☒	The statistical test(s) used AND whether they are one- or two-sided<br>*Only common tests should be described solely by name; describe more complex techniques in the Methods section.*
☒	☐	A description of all covariates tested
☒	☐	A description of any assumptions or corrections, such as tests of normality and adjustment for multiple comparisons
☐	☒	A full description of the statistical parameters including central tendency (e.g. means) or other basic estimates (e.g. regression coefficient) AND variation (e.g. standard deviation) or associated estimates of uncertainty (e.g. confidence intervals)
☐	☒	For null hypothesis testing, the test statistic (e.g. F, t, r) with confidence intervals, effect sizes, degrees of freedom and P value noted<br>*Give P values as exact values whenever suitable.*
☐	☒	For Bayesian analysis, information on the choice of priors and Markov chain Monte Carlo settings
☒	☐	For hierarchical and complex designs, identification of the appropriate level for tests and full reporting of outcomes
☐	☒	Estimates of effect sizes (e.g. Cohen's d, Pearson's r), indicating how they were calculated

Our web collection on statistics for biologists contains articles on many of the points above.

Software and code

Policy information about availability of computer code

Data collection	For the collection of clinical trial data: Medidata RAVE (EDC) 21 CFR Part 11 compliant.
Data analysis	Statistical analyses were performed using the most recent version of SAS® and R (version 4.5.3), incorporating packages such as voom (4.8.2), limma (3.66.0), and msigdbr (24.1.0). Noncompartmental pharmacokinetic analyses were conducted using Phoenix WinNonlin (version 8.3; Certara USA, Inc., Princeton, NJ, 2020). Population pharmacokinetic analyses were performed using Monolix 2023R1, and figures were generated using R (version 4.3.1).

For manuscripts utilizing custom algorithms or software that are central to the research but not yet described in published literature, software must be made available to editors and reviewers. We strongly encourage code deposition in a community repository (e.g. GitHub). See the Nature Portfolio guidelines for submitting code & software for further information.

Data

Policy information about availability of data

All manuscripts must include a data availability statement. This statement should provide the following information, where applicable:

- Accession codes, unique identifiers, or web links for publicly available datasets
- A description of any restrictions on data availability
- For clinical datasets or third party data, please ensure that the statement adheres to our policy

All data associated with this study are in the paper or the Supplementary Materials with the exception of individualized patient genomic and other exploratory

biomarker data (including SomaScan and bulk RNA-seq). No new code was generated for this study. Novartis is committed to sharing with qualified external researchers access to patient-level clinical data and supporting clinical documents from the clinical study. These requests are reviewed and approved by an independent review panel on the basis of scientific merit. All data provided are anonymized to respect the privacy of patients who have participated in the trial in line with applicable laws and regulations. However, institutional review board approval and patient consent to share individualized patient genomic data (including SomaScan and bulk RNA-Seq) are not available; therefore, in accordance with the Health Insurance Portability and Accountability Act, these data cannot be reported in a public data repository. It is also Novartis policy for small phase 1 studies to avoid sharing this data type (i.e., patient-level exploratory biomarker/genomic results) due to the risk of patient reidentification. Upon request, Novartis can provide gene-set level aggregate values. In addition, clinical data, in some cases, have been collected under contractual or consent provisions that prohibit transfer to third parties. Such restrictions may preclude granting access under these provisions. Where co-development agreements or other legal restrictions prevent sharing particular data, Novartis will work with qualified requestors to provide summary information where possible. The data availability of these trials is according to the criteria and process described at www.clinicalstudydatarequest.com, which is also where requests to access data, including gene-set level aggregate data, may be made. Any proprietary or patient-derived materials used or generated in this study are not commercially available and cannot be shared. All other materials in this study are commercially available.

Research involving human participants, their data, or biological material

Policy information about studies with human participants or human data. See also policy information about sex, gender (identity/presentation), and sexual orientation and race, ethnicity and racism.

Reporting on sex and gender	Biological sex (male and female) was self‐reported by all study participants. Sex‐based analyses were not conducted, as available published data indicate no sex‐related differences in disease incidence, with observed variability primarily associated with latitude and skin pigmentation. The Full Analysis Set (FAS) and Safety Set (SS) included all patients who received at least one dose of study treatment.
Reporting on race, ethnicity, or other socially relevant groupings	Demographics and other baseline data including disease characteristics is summarized descriptively for the FAS. Demographics data was collected for all patients (as allowed per local laws) as follows: Year of or Full Date or Year of Birth; Sex; Race/predominant ethnicity (if permitted); relevant medical history/current medical condition (until date of signature of informed consent); concomitant medications; diagnosis and extent of cancer; baseline tumor molecular characteristics; and details of prior antineoplastic treatments.
Population characteristics	Covariate relevant baseline characteristics have been included as table1 in the manuscript. Briefly the study population was heavily pretreated population (N=66) with a median age of 56.5 years (range 25–82) and balanced sex distribution (males 48.5% and females 51.5%) Most patients had uveal melanoma (90.9%), ECOG PS 0–1 (100%), and metastatic disease, with common involvement of the liver (86.4%) and lung (43.9%). Elevated LDH was observed in a substantial proportion of patients. The majority had received prior systemic therapies (92.4%), including immune checkpoint inhibitors (72.7%) and tebentafusp (33.3%), with ≥2 prior regimens in 59.1% of patients.
Recruitment	IRB/IEC approved advertisement. In addition to the limitations inherent to early-phase studies, potential sources of bias should be considered. Patients were enrolled at selected specialized centers across multiple geographic regions, which may introduce site selection bias and limit generalizability to broader clinical settings. Furthermore, participation in a Phase 1 clinical trial may be associated with self-selection of patients with better performance status, higher motivation, or greater access to specialized care. Collectively, these factors may affect the interpretation and generalizability of these findings
Ethics oversight	The ethics approval statement has been revised to include the names of the approving institutions/organizations and their respective Ethics Committees in both the manuscript and the reporting summary.<br>Revised text: This study was conducted in accordance with the International Council for Harmonisation of Technical Requirements for the Registration of Pharmaceuticals for Human Use (ICH) Harmonised Tripartite Guidelines for Good Clinical Practice, with applicable local regulations, including European Directive 2001/20/EC and US Code of Federal Regulations Title 21, and the Declaration of Helsinki. The protocol and all amendments were reviewed and approved by appropriately constituted Institutional Review Boards/Independent Ethics Committees/Research Ethics Boards (IRB/IEC/REB) prior to study initiation. The approving institutions/organizations and their respective IRB/ECs included Bellberry Human Research Ethics Committee; Peter MacCallum Cancer Centre Ethics Committee – Melbourne; the Ethics Committee assigned under the EU Clinical Trials Regulation (France); Ethik Kommission der Medizinischen Fakultät der Universität Duisburg Essen; Medisch Ethische Toetsingscommissie – Leiden, Den Haag, Delft; Columbia University IRB – Human Research Protection Office; Dana Farber Cancer Institute – Office of Human Research Studies; Memorial Sloan Kettering Cancer Center IRB/ Privacy Board; HM Hospitales CEIm – Comité de Ética de la Investigación con Medicamentos; and Kanton Zurich – Kantonale Ethikkommission. Eligible patients provided written IRB/IEC/REB-approved informed consent before study initiation.

Note that full information on the approval of the study protocol must also be provided in the manuscript.

Field-specific reporting

Please select the one below that is the best fit for your research. If you are not sure, read the appropriate sections before making your selection.

☒ Life sciences ☐ Behavioural & social sciences ☐ Ecological, evolutionary & environmental sciences

For a reference copy of the document with all sections, see nature.com/documents/nr-reporting-summary-flat.pdf

Life sciences study design

All studies must disclose on these points even when the disclosure is negative.

Sample size	Dose escalation decision was guided by BHLRM. Cohort of 3 to 6 evaluable patients were enrolled in the dose escalation portion of the study. Additional cohorts of 1 to 6 patients were enrolled at dose level that satisfies the EWOC criteria.
Data exclusions	In differential expression analysis, as described in the Methods: For statistical testing, only liver samples were included because healthy tissue cells on the slide could not be excluded from the assay and their gene expression data could lead to extensive signal unrelated to the tumor gene expression. Importantly, 20 of 23 patients with known sampling location had liver samples in the data set. For the exposure-response figures, one patient treated at the lowest dose who had high clearance and apparent Target?Mediated Drug Disposition (TMDD) was excluded from the analysis as their PK could not be described using the linear PK model that describes the rest of the population. There were no data exclusions in clinical summary tables.
Replication	Not applicable, as this was a clinical study involving individual patients. All analyses have been validated by an independent subject matter expert and was reproduced by the validator.
Randomization	No IRT was utilized (non-randomized.) Patients were allocated to a Cohort based on sponsor and investigator discretion.
Blinding	Open label

Reporting for specific materials, systems and methods

We require information from authors about some types of materials, experimental systems and methods used in many studies. Here, indicate whether each material, system or method listed is relevant to your study. If you are not sure if a list item applies to your research, read the appropriate section before selecting a response.

Materials & experimental systems

n/a	Involved in the study	
☐	☒	Antibodies
☒	☐	Eukaryotic cell lines
☒	☐	Palaeontology and archaeology
☒	☐	Animals and other organisms
☐	☒	Clinical data
☒	☐	Dual use research of concern
☒	☐	Plants

Methods

n/a	Involved in the study	
☒	☐	ChIP-seq
☒	☐	Flow cytometry
☐	☒	MRI-based neuroimaging

Antibodies

Antibodies used	Anti-PMEL (gp100) antibody (Clone HMB45) from Dako Systems (Cat # M0634) was used at 1:50 dilution. The Isotype control used was from Dako systems (Clone DA1E, Cat# X093101)
Validation	The assay, along with the antibody was validated for accuracy, precision, sensitivity, specificity, reproducibility and repeatability. The antibody HMB45 was purchased from Dako Systems and is widely used IVD antibody with an extensive publication record (Am J Pathol 1986; 123(2):195-203). Details of the antibody are available at the Agilent website (https://www.agilent.com/cs/library/packageinsert/public/Copy%20of%20303299EN_04.pdf)

Clinical data

Policy information about clinical studies

All manuscripts should comply with the ICMJE guidelines for publication of clinical research and a completed CONSORT checklist must be included with all submissions.

Clinical trial registration	ClinicalTrials.gov identifier: NCT05415072
Study protocol	CDYP688A12101
Data collection	Multi-center Global Trial. Data were collected at hospitals, academic medical centers, and clinics across multiple global sites (given below). Recruitment occurred from 04 July 2022 (first patient first visit) to 08 July 2025 (enrollment halt), consistent with ClinicalTrials.gov.<br>1. Westmead Hospital<br>2. Peter MacCallum Cancer Institute<br>3. Institut Curie<br>4. Universitaetsklinikum Essen Westdeutsches Tumorzentrum

5. Universitaetsklinikum Heidelberg Nat. Centrum f. Tumorerkrank
6. Leids Universitair Medisch Centrum
7. Columbia University Medical Center- New York Presbyterian Onc Dept
8. Massachusetts General Hospital Hematology Oncology
9. Memorial Sloane Kettering Cancer Center MSKCC
10. Centro Integral Oncologico HM CIOCC CIOCC START Madrid
11. Universitaetsspital Zuerich Dermatologische Klinik

Outcomes

The primary and secondary outcomes were prospectively defined in the protocol. For the dose escalation phase, the primary objective was to characterize safety and tolerability and determine the MTD and/or recommended dose(s), with DLTs during the first 28 days as the primary endpoint, alongside additional safety and tolerability measures (AEs, SAEs, laboratory parameters, vital signs, ECG, and dose modifications).
Secondary objectives included characterization of pharmacokinetics, evaluation of preliminary antitumor activity (BOR and ORR per RECIST v1.1), and assessment of immunogenicity. Exploratory endpoints included PFS, OS, and pharmacodynamic and biomarker analyses. Outcomes were assessed using standard criteria, and statistical analyses were primarily descriptive, with continuous variables summarized as medians and ranges and categorical variables as counts and percentages; time to event endpoints were analyzed using Kaplan–Meier methods, and exploratory analyses were presented descriptively without formal hypothesis testing.

Plants

Seed stocks

Report on the source of all seed stocks or other plant material used. If applicable, state the seed stock centre and catalogue number. If plant specimens were collected from the field, describe the collection location, date and sampling procedures.

Novel plant genotypes

Describe the methods by which all novel plant genotypes were produced. This includes those generated by transgenic approaches, gene editing, chemical/radiation-based mutagenesis and hybridization. For transgenic lines, describe the transformation method, the number of independent lines analyzed and the generation upon which experiments were performed. For gene-edited lines, describe the editor used, the endogenous sequence targeted for editing, the targeting guide RNA sequence (if applicable) and how the editor was applied.

Authentication

Describe any authentication procedures for each seed stock used or novel genotype generated. Describe any experiments used to assess the effect of a mutation and, where applicable, how potential secondary effects (e.g. second site T-DNA insertions, mosiacism, off-target gene editing) were examined.

Magnetic resonance imaging

Experimental design

Design type

MRI data was submitted to central vendor after being locally acquired in DICOM format.

Design specifications

Specify the number of blocks, trials or experimental units per session and/or subject, and specify the length of each trial or block (if trials are blocked) and interval between trials.

Behavioral performance measures

State number and/or type of variables recorded (e.g. correct button press, response time) and what statistics were used to establish that the subjects were performing the task as expected (e.g. mean, range, and/or standard deviation across subjects).

Acquisition

Imaging type(s)

Contrast Enhanced MRI (Abdomen/Pelvis); Brain pre (optional) and post contrast enhanced CT as clinically indicated; Localized Bone Scan per site standard of care (e.g., FDG-PET, CT, MRI or x-rays as clinically indicated .)

Field strength

No central reading was utilized. Images were locally obtained and centrally collected.
TR and TE collected according to manufacturer specific and field strength recommendations: Approx Field of view (38 cm); Slice thickness (5); Slice, Gap Spacing (0); Acquisition Matrix (>/=256x160); NEX/NSA (1); Comments (Prescribe slices Superior to Inferior)

Sequence & imaging parameters

See above

Area of acquisition

Chest
Pelvis/Abdomen (top of liver to symphysis pubis) required
Axial T1 or T2 Weighted/Fat Sat
Pulse Sequence: Acquire images with breath hold or respiratory trigger, monitor (PACE) depending on MRI system capabilities. For multiple series coverage ensure series overalps by minimum of 3 slices.
Brain and localized bone scans as clinically indicated

Diffusion MRI ☐ Used ☒ Not used

Preprocessing

Preprocessing software	Local software per institutions local practice and guidelines.
Normalization	*If data were normalized/standardized, describe the approach(es): specify linear or non-linear and define image types used for transformation OR indicate that data were not normalized and explain rationale for lack of normalization.*
Normalization template	*Describe the template used for normalization/transformation, specifying subject space or group standardized space (e.g. original Talairach, MNI305, ICBM152) OR indicate that the data were not normalized.*
Noise and artifact removal	*Describe your procedure(s) for artifact and structured noise removal, specifying motion parameters, tissue signals and physiological signals (heart rate, respiration).*
Volume censoring	*Define your software and/or method and criteria for volume censoring, and state the extent of such censoring.*

Statistical modeling & inference

Model type and settings	*Specify type (mass univariate, multivariate, RSA, predictive, etc.) and describe essential details of the model at the first and second levels (e.g. fixed, random or mixed effects; drift or auto-correlation).*
Effect(s) tested	*Define precise effect in terms of the task or stimulus conditions instead of psychological concepts and indicate whether ANOVA or factorial designs were used.*

Specify type of analysis: ☐ Whole brain ☐ ROI-based ☐ Both

Statistic type for inference (See Eklund et al. 2016)	*Specify voxel-wise or cluster-wise and report all relevant parameters for cluster-wise methods.*
Correction	*Describe the type of correction and how it is obtained for multiple comparisons (e.g. FWE, FDR, permutation or Monte Carlo).*

Models & analysis

n/a	Involved in the study
☐	☐ Functional and/or effective connectivity
☐	☐ Graph analysis
☐	☐ Multivariate modeling or predictive analysis

Functional and/or effective connectivity	*Report the measures of dependence used and the model details (e.g. Pearson correlation, partial correlation, mutual information).*
Graph analysis	*Report the dependent variable and connectivity measure, specifying weighted graph or binarized graph, subject- or group-level, and the global and/or node summaries used (e.g. clustering coefficient, efficiency, etc.).*
Multivariate modeling and predictive analysis	*Specify independent variables, features extraction and dimension reduction, model, training and evaluation metrics.*
