# Supplementary material for: An anti-PMEL antibody−drug conjugate with a Gq/11 inhibitor payload in GNAQ/GNA11-mutant melanomas: a phase 1 trial
Source: Nat Med. 2026 Jul 13;32(9):3248–58. doi: 10.1038/s41591-026-04518-z (PMC13577922; doi:10.1038/s41591-026-04518-z)
Supplement: Supplementary file 3 [file 41591_2026_4518_MOESM3_ESM.pdf]

nature portfolio

Peer Review File

An anti-PMEL antibody-drug conjugate with a GNAQ/11 inhibitor payload for mutant melanomas: a Phase 1 trial

Corresponding Author: Dr Matteo Carlino

This file contains all reviewer reports in order by version, followed by all author rebuttals in order by version.

Version 0:

Reviewer comments:

Reviewer #1

(Remarks to the Author)
Overall comments:

This first-in-human phase 1 study evaluated DYP688, a PMEL-targeted antibody drug conjugate delivering a Gq/11 inhibitor (SDZ475) in patients with metastatic uveal melanoma (mUM) and other GNAQ/11-mutant melanomas. Among 66 heavily pretreated patients, DYP688 demonstrated a favorable safety profile with mainly grade 2 or lower AEs. Importantly, pharmacokinetic analysis confirmed effective payload delivery with minimal systemic exposure to free drug, which is critical based upon the toxicity of untargeted SDZ475. Preliminary efficacy was modest with objective responses in 18.2% of patients, but some patients demonstrated ongoing responses >1 year which is encouraging for targeted therapies, especially in a highly treatment resistant cancer like mUM which has few options. The report is succinct and informative, however there are some issues that require clarification as described below:

Major Comments:

1. The authors state that patients with high baseline tumor burden, high lactate dehydrogenase (LDH), and high ctDNA fraction were less likely to respond/show tumor regression to treatment. To help the reader understand which patients benefited, please include uveal melanoma AJCC M staging to the demographic table. (i.e. M1a, b, c)
2. To help understand the nature of the confirmed responses, please provide the sites that showed regression (liver vs lung vs other).
3. Since all patients were screened to have GNAQ/11 driver mutations, please provide hypotheses for the modest ORR.
4. Melanocytic antigen expression of PMEL, MART-1, and tyrosinase are known to have variable intra-tumoral and inter-tumoral protein expression and to demonstrate tumor cells with no expression based upon protein and transcriptomic expression analysis. Since antigen density is critical to the MOA of DYP688, greater clarity of the PMEL expression would be helpful. Although the authors cite IHC H score in Fig 4c, it would be important to also present the % of cells with surface PMEL and intensity of PMEL staining as individual metrics.
5. To understand the immunogenicity of the DYP688 and the feasibility of repeat dosing, were neutralizing antibodies identified after repeat dosing that would limit treatment duration?
6. The safety of DYP688 is encouraging, but it is curious that PMEL expressed by normal melanocytes in the skin showed no toxicity. Were any skin punch biopsies performed? Is there evidence that the drug may sequester in the skin.
7. Please add statistical testing to support that “mutations in BAP1 but not SF3B1 were associated with lesser best percentage change in tumor diameters” as described in Supplementary Fig. 5.

Reviewer #2

(Remarks to the Author)
In this article, Carlino et al., demonstrate the activity of DYP688, a novel double-target anti-PMEL ADC for metastatic uveal melanoma and other similar tumours. In brief, the article is novel in both describing the structure and activity of the ADC and is a welcome addition to the armamentarium for uveal melanoma. The methodology is appropriate and my comments below are partly designed to improve the impact of the publication across a number of issues

MAJOR

1. The nature of the high rates oh hypercalcemia are unclear from the text - is this something that was expected from the preclinical data?
2. given the interest in the field, coudl the authors comment in the article if any responses were seen in any primary lesions that may have been still in situ?
3. There appears to be an overemphasis on the prognosis of the BAP1 mutations in the text - the supporting figure , supplemental figure 5 isn't statistically justified and it appears there is a lot of overlap. There is also the issue that some BAP1 genes may be methylated or limited analytically ? VUS ? other by the ctDNA assay used suggesting that some of the BAP1 WT patients may in fact have mutated proteins
4. Can the authors provide information (perhaps in a supplement) just for the uveal patients on the study as these are certainly the patients with the greatest unmet need
5. the discussion Lines 296-300 doesn't mention the combination of PKC and MET inhibition, but rather only the more ineffectual PKC/AKT inhibition - its not clear why these are separated in the text
6. especially because of the de facto comparison (lines 337-340) the authors should include somewhere in the article, the effectiveness of the combination in patients pre-treated with Darovasertib/Crizotinib. This could go in the figure 3a (similar to the Tebentafusp) for example
7. Usually articles such as this one, end with a plan for future development. This detail is sorely missing. Can this be added?
8. Did the post samples acquired highlight any possibilities for resistance pathways? lines 250-252. IN particular, MEK pathway activation such as was foound with Darovasertib alone

MINOR
1. Reference 9 is an old reference outlining the relapse risk of melanomas, please use a more contemporary reference that may suggest that the relapse risk is indeed likely higher when patients are followed with contemporary imaging
2. Line 78, it is not clear if the hotspot mutations mentioned are in the GNAQ or GNA11 from the way the text is written. please rewrite.
3. Especially in the introduction there is switching between the PMEL term, and using gp100 as an equivalent. Please harmonise
4. Line 106, nature of SDZ475 already mentioned earlier ? need to repeat
5. line 195- IM not clear why these fractions were not converted into fractions (the first one was only?)
6. line 196 - the protocol deviation was that the patient was enrolled with non RECIST disease not that they had cutaneous melanoma - please rewrite text ?
7. it is unclear what the authors mean by "dynamic interaction" - they are referring to the hypothesis on line 236. Could these be rewritten?
8. How is "responded to any prior therapy" defined? does it include SD > 12 weeks?
9 A graphic outlining the MOA of this drug and others in the class would be helpful for the readership

Reviewer #3

(Remarks to the Author)
This phase 1 clinical trial reports describes a novel antibody-drug conjugate engineered to deliver a Gq/11 inhibitor SDZ475 to uveal melanoma cells via PMEL binding. This is a novel therapeutic explored in a drug development space with significant patient need. The therapy was very well-tolerated with impressive efficacy. The data were presented clearly with extensive discussion of PK, PD, and biomarkers above even an expectation in phase 1 studies. A few comments for review below.

Line 121: Should say "Patient characteristics"
Line 123: replace . after 2022 with ,
Line 136: This sentence is redundant unless additional information should have been included.
Line 138: How was best response to prior therapy determined? All treatments were on trial or was this investigator-assessment? Does this include prior liver-directed therapy?
Line 211: Was there a difference in response based on hepatic vs extrahepatic disease?
Line 295: Recommend rephrasing since reported response rate for darovasertib/crizotinib is higher than reported here despite downstream blockade. Also 74% of patients had confirmed disease progression with DYP688 so patients are also primarily progressing on this therapy, as with darovasertib/crizotinib, which indicates development of resistance regardless of sites of blockade.
Line 339: Were any patients previously treated with darovasertib/crizotinib? Did they respond? Would be interested in comment based on mechanisms of action and potential pathways of resistance
Line 692: Please distinguish what +/- indicates.
Line 694: Recommend changing color for first CR since difficult to distinguish between PR and CR on chart
Line 710: Was there a difference in baseline or on treatment in patients that received prior GNAQ/11 targeting therapy (daro/criz)?
Line 859: A waterfall plot for change in ctDNA on treatment color-coded by response by imaging may be easier to interpret than supplementary figure 2.
Line 861: Recommend revising statement saying "treatment seems to".
Line 867: Clarify how a patient is distinguished as "high or low"
Line 875: Supplementary figure 4 is difficult to demonstrate trends. I suggest removing.
Line 888: Supplementary figure 5 caption notes that BAP1 mutation is associated with a worse response but the median is still a % decrease. Recommend re-wording assessment.

Version 1:

Reviewer comments:

Reviewer #1

(Remarks to the Author)
The responses to my queries are satisfactory and revisions appropriate.

Reviewer #2

(Remarks to the Author)
I am generally satisfied with the responses to the revisions. My comments are

1. Approximately 85%–90% of UMs harbor mutually exclusive activating mutations in either GNAQ or GNA11, most commonly affecting the Q209 (~80%) or R183 hotspots." - Again, in which gene is the Q209 and R183 hotspots? Do the authors need to add the word "respectively"
2. Figure 3a/b - please separate out Darovasertib ONLY or Darovasertib AND Crizotinib pre-treatments. The responses for the doublet shoudl be explicitly called out
3. The authors need to update the manuscript with the recent press release from Ideaya about the efficacy of Darovasertib and Crizotinib in the discussion
4. the graphic is great!

Reviewer #3

(Remarks to the Author)
Supplementary Table 3 lists 1 patient with non-measurable disease, however, for Figure 3a describes 2 patients that were non-evaluable. It seems an additional patient should be included as non-measurable at baseline.

Supplementary Table 5 does not add substantively to the manuscript but will defer to the authors if it is felt to contribute.

Open Access This Peer Review File is licensed under a Creative Commons Attribution 4.0 International License, which permits use, sharing, adaptation, distribution and reproduction in any medium or format, as long as you give appropriate credit to the original author(s) and the source, provide a link to the Creative Commons license, and indicate if changes were made.
In cases where reviewers are anonymous, credit should be given to 'Anonymous Referee' and the source.
The images or other third party material in this Peer Review File are included in the article's Creative Commons license, unless indicated otherwise in a credit line to the material. If material is not included in the article's Creative Commons license and your intended use is not permitted by statutory regulation or exceeds the permitted use, you will need to obtain permission directly from the copyright holder.

To view a copy of this license, visit https://creativecommons.org/licenses/by/4.0/

Response to referees

Reviewers' Comments:

Reviewer #1 (Remarks to the Author):

Overall comments:

This first-in-human phase 1 study evaluated DYP688, a PMEL-targeted antibody drug conjugate delivering a Gq/11 inhibitor (SDZ475) in patients with metastatic uveal melanoma (mUM) and other GNAQ/11-mutant melanomas. Among 66 heavily pretreated patients, DYP688 demonstrated a favorable safety profile with mainly grade 2 or lower AEs. Importantly, pharmacokinetic analysis confirmed effective payload delivery with minimal systemic exposure to free drug, which is critical based upon the toxicity of untargeted SDZ475. Preliminary efficacy was modest with objective responses in 18.2% of patients, but some patients demonstrated ongoing responses >1 year which is encouraging for targeted therapies, especially in a highly treatment resistant cancer like mUM which has few options. The report is succinct and informative, however there are some issues that require clarification as described below:

Major Comments:

1. The authors state that patients with high baseline tumor burden, high lactate dehydrogenase (LDH), and high ctDNA fraction were less likely to respond/show tumor regression to treatment. To help the reader understand which patients benefited, please include uveal melanoma AJCC M staging to the demographic table. (i.e. M1a, b, c)

Author response: We thank the reviewer for this suggestion. AJCC v8 M staging for uveal melanoma has now been added to Table 1.

Baseline characteristics	4 mg/kg Q2W N=5	8 mg/kg Q2W N=12	12 mg/kg Q2W N=13	16 mg/kg Q2W N=14	24 mg/kg Q2W N=11	12 mg/kg QW N=5	16 mg/kg QW N=6	All patients N=66
Number of UM patients	5	12	9	13	10	5	6	60
Metastatic category at baseline (AJCC) for UM patients, n (%)								
M0	0	0	0	1 (7.7)	0	0	0	1 (1.7)
M1	1 (20.0)	0	0	0	0	0	0	1 (1.7)
M1a	1 (20.0)	2 (16.7)	4 (44.4)	4 (30.8)	3 (30.0)	2 (40.0)	3 (50.0)	19 (31.7)
M1b	1 (20.0)	5 (41.7)	2 (22.2)	5 (38.5)	5 (50.0)	2 (40.0)	1 (16.7)	21 (35.0)
M1c	2 (40.0)	5 (41.7)	3 (33.3)	3 (23.1)	1 (10.0)	1 (20.0)	2 (33.3)	17 (28.3)
Unknown	0	0	0	0	1 (10.0)	0	0	1 (1.7)

2. To help understand the nature of the confirmed responses, please provide the sites that showed regression (liver vs lung vs other).

Author response: We thank the author for this suggestion. Upon analyzing the best change in lesion size by location, no clear trend was observed between lesions located in the liver, lung, lymph node vs other sites. We have included the text in *green font* in the "Results, Efficacy" section of the manuscript.

Revised text (*green font*): Best percentage change from baseline in sum of longest target lesion diameters based on local radiology review is shown in Fig. 3a. Tumor size reduction was observed in 47/66 (71.2%) patients. *An analysis of the best percent change in target lesion size by anatomical location showed tumor shrinkage across liver, lung, lymph node, and other sites, with no evidence of preferential response by organ site (data not shown).* Fourteen (21.2%) patients remained on treatment for ≥ 12 months (Fig.3b).

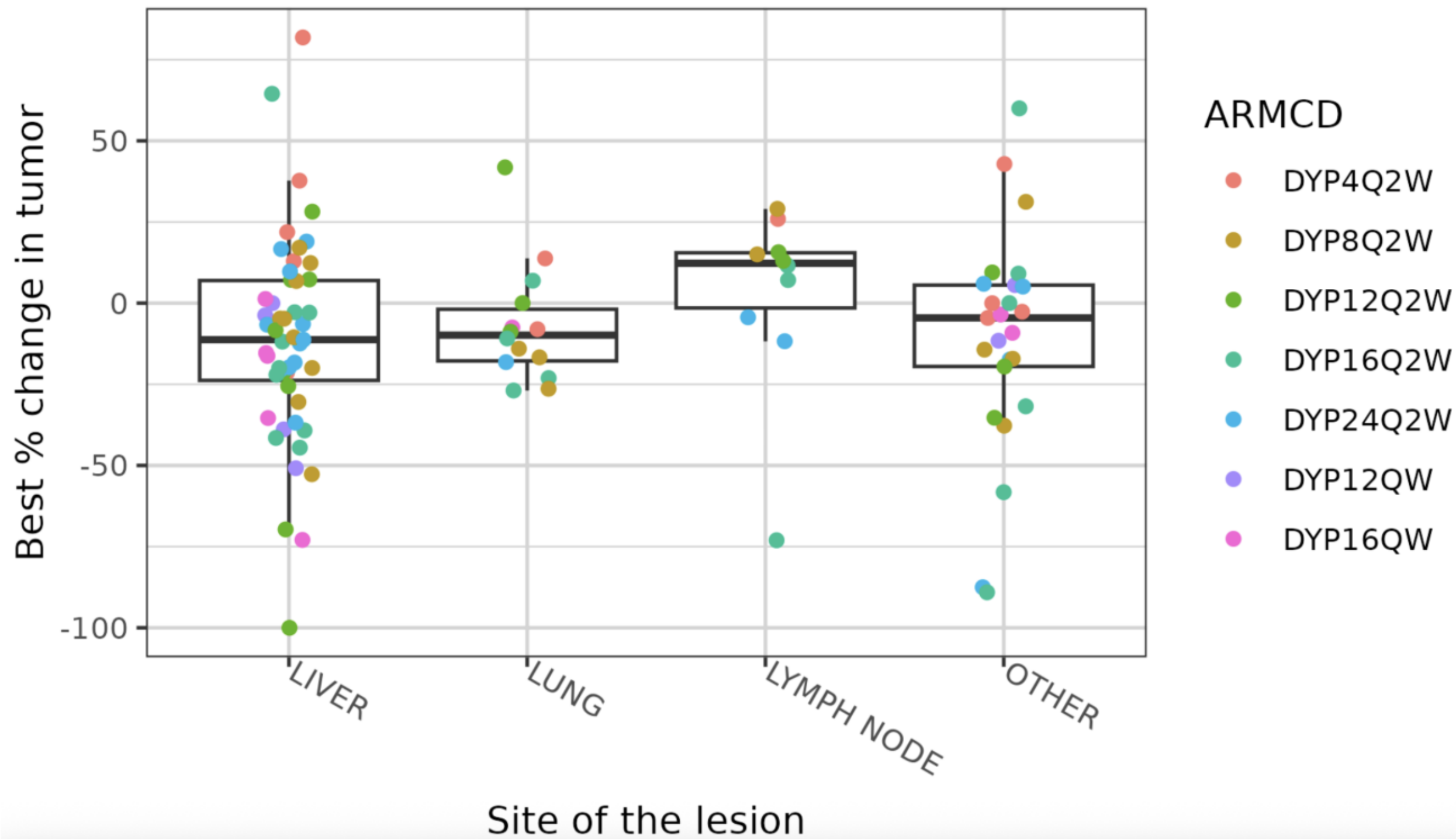

Figure: Best percentage change in target lesion size by anatomical location. Best percent change in tumor size from baseline is shown for individual target lesions stratified by lesion location (liver, lung, lymph node, and other). Each point represents an individual lesion, colored by DYP688 dosing regimen, and boxplots summarize the distribution within each site.

3. Since all patients were screened to have GNAQ/11 driver mutations, please provide hypotheses for the modest ORR.

Author response: We thank the reviewer for this observation. Although the objective response rate (ORR) was approximately 20%, the disease control rate was close to 82%, with a substantial proportion of patients experiencing stable disease and measurable tumor shrinkage, indicating meaningful clinical benefit beyond confirmed responses. Thus, the vast majority of patients derived some level of benefit from treatment. The absence of objective responses (OR) in some patients is not unexpected and is a common observation across oncology therapies, even when tumors harbor the presumed driver mutation. In this study, mutation screening was required for non-uveal melanoma patients, whereas in uveal

melanoma, where approximately 95% of patients harbor GNAQ or GNA11 mutations, screening was not required. Importantly, the presence of a dominant driver mutation does not preclude additional genetic or nongenetic factors, including sub clonal heterogeneity, that may influence treatment response.

Furthermore, the modest ORR for all patients includes patients treated at dose levels below the biologically active range. As is typical in phase 1 dose escalation- studies, early cohorts received subtherapeutic or minimally active doses, which may underestimate the true antitumor activity of the investigational agent. At the recommended dose for expansion, response rates were numerically higher (36%), supporting the conclusion that limited activity at lower dose levels contributed to the modest ORR.

Taken together, these factors suggest that while GNAQ/GNA11 mutations are key oncogenic drivers, they are not singular determinants of treatment response, and the precise reasons for non-response in some patients remain multifactorial and incompletely understood.

We have included the explanation in the “Discussion” section of the manuscript.

Revised discussion text (*green font*): Anti-tumor activity was observed at doses ≥8 mg/kg with objective responses in patients who commenced treatment at doses ≥12mg/kg or after intra-patient dose escalation to 24mg/kgQ2W. *Although the ORR was ~20%, the DCR was high (~82%), with many patients experiencing SD and measurable tumor shrinkage, indicating clinically meaningful benefit beyond confirmed responses. The modest ORR likely reflects several factors, including the inclusion of patients treated in early dose-escalation cohorts who received doses below the biologically active range, intra- and intertumoral heterogeneity influencing response despite the presence of GNAQ/GNA11 driver mutations, and the potential emergence of resistance, among other factors.* The MTD was not identified, as DYP688 demonstrated an acceptable safety and tolerability profile across all administered dose levels and schedules.

4. Melanocytic antigen expression of PMEL, MART-1, and tyrosinase are known to have variable intra-tumoral and inter-tumoral protein expression and to demonstrate tumor cells with no expression based upon protein and transcriptomic expression analysis. Since antigen density is critical to the MOA of DYP688, greater clarity of the PMEL expression would be helpful. Although the authors cite IHC H score in Fig 4c, it would be important to also present the % of cells with surface PMEL and intensity of PMEL staining as individual metrics.

Author response: The authors thank the reviewer for the suggestion highlighting the importance of antigen density for the mechanism of action of DYP688.

In addition to the IHC H score- presented in Fig. 4c, we now report the percentage of tumor cells with detectable surface PMEL expression at baseline. As shown in the below figure, this information is presented alongside best overall response and relevant prior therapies for each patient, allowing a more granular assessment of the relationship between PMEL expression and clinical outcome.

As seen from the figure, most patients had high PMEL expression at screening. Response in patients seem to be independent of their PMEL expression. We do not observe a difference in PMEL expression relative to the patient’s prior treatment.

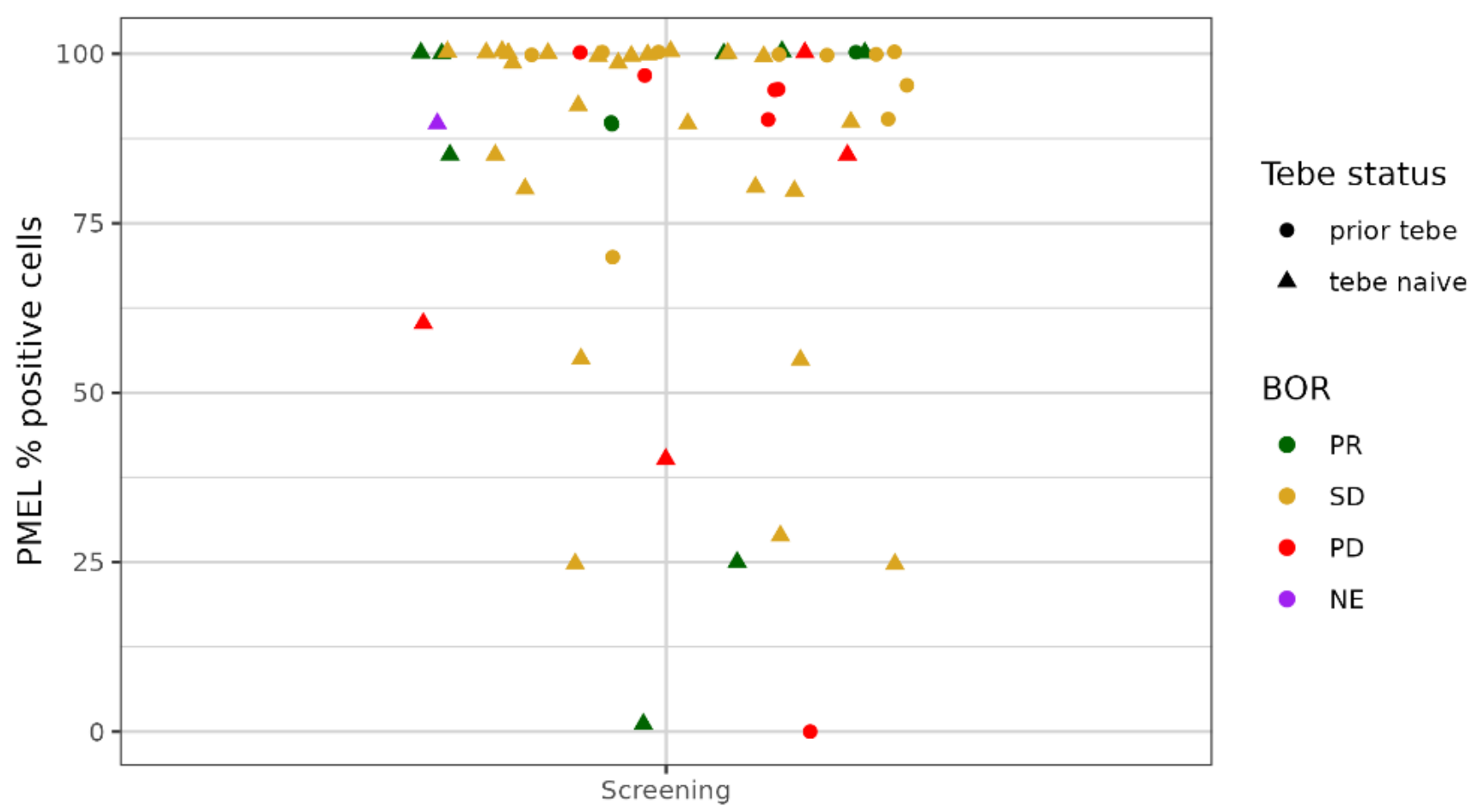

Fig 4d: Scatter plot showing the percentage of PMEL positive tumor cells at screening for individual patients. Each symbol represents one patient and is annotated by prior tebentafusp treatment (shape) and BOR (color).

We have included the data in the “Results, Biomarker analysis” section and the figure as Fig. 4d to the manuscript.

Revised Results text (*green font*): …. hypothesis that changes in circulating PMEL likely stem from engagement of the protein by DYP688, rather than changes in expression of PMEL by the tumor. *In addition to the IHC H score analysis, the percentage of tumor cells with detectable surface PMEL expression at baseline is shown in Fig. 4d. This information is presented alongside best overall response and prior tebentafusp therapy for each patient, enabling a more granular evaluation of the relationship between baseline PMEL expression and clinical outcome. Most patients exhibited high levels of PMEL expression at screening. Clinical responses appeared independent of baseline PMEL expression, and no clear differences in PMEL expression were observed according to prior treatment history.*

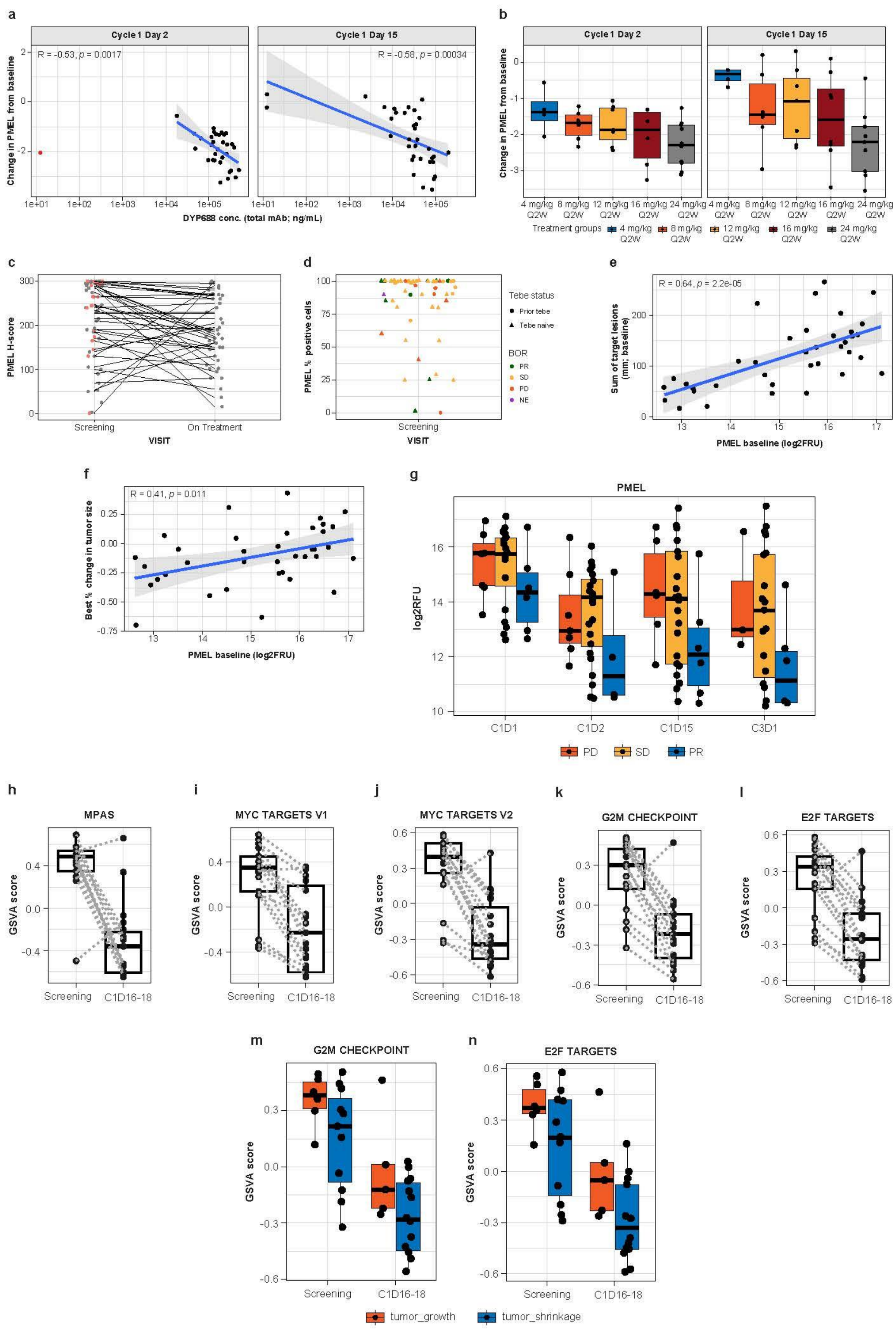
a
Cycle 1 Day 2
Cycle 1 Day 15
R = -0.53, p = 0.0017
R = -0.58, p = 0.00034
Change in PMEL from baseline
DYP688 conc. (total mAb; ng/mL)
b
Cycle 1 Day 2
Cycle 1 Day 15
Change in PMEL from baseline
4 mg/kg Q2W
8 mg/kg Q2W
12 mg/kg Q2W
16 mg/kg Q2W
24 mg/kg Q2W
Treatment groups
c
PMEL H-score
Screening
On Treatment
VISIT
d
PMEL % positive cells
Screening
VISIT
Tebe status
Prior tebe
Tebe naive
BOR
PR
SD
PD
NE
e
R = 0.64, p = 2.2e-05
Sum of target lesions (mm; baseline)
PMEL baseline (log2FRU)
f
R = 0.41, p = 0.011
Best % change in tumor size
PMEL baseline (log2FRU)
g
PMEL
log2RFU
C1D1
C1D2
C1D15
C3D1
PD
SD
PR
h
MPAS
i
MYC TARGETS V1
j
MYC TARGETS V2
k
G2M CHECKPOINT
l
E2F TARGETS
GSVA score
Screening
C1D16-18
m
G2M CHECKPOINT
n
E2F TARGETS
GSVA score
Screening
C1D16-18
tumor_growth
tumor_shrinkage

5. To understand the immunogenicity of the DYP688 and the feasibility of repeat dosing, were neutralizing antibodies identified after repeat dosing that would limit treatment duration?

Author response: The immunogenicity assays employed in this study follow a three-tiered approach, comprising screening, confirmatory, and titer assessments. Neutralizing antibody testing was not conducted in this study and would need to be assessed in future studies.

Available preliminary data indicate low immunogenicity of DYP688, with anti-drug antibody (ADA) incidence of 6.3%.

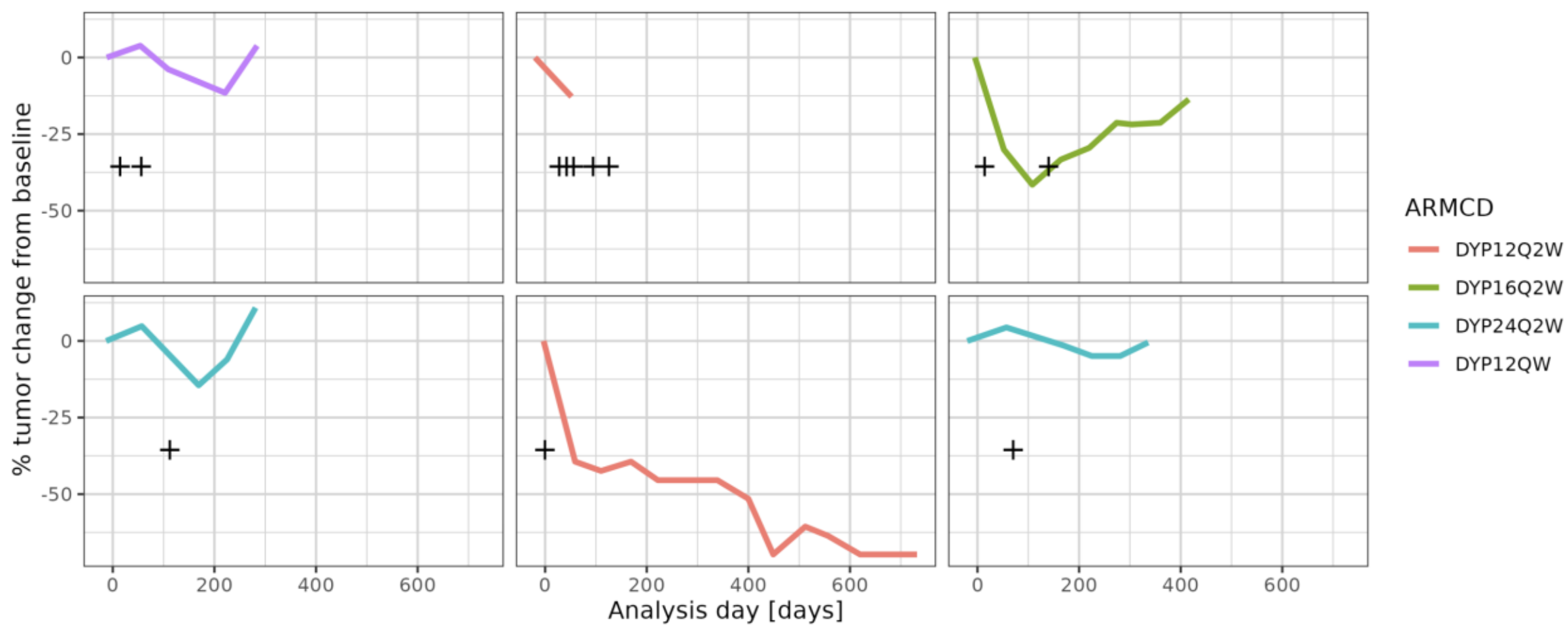

The spider plots illustrate the percentage change from baseline in tumor size over time for each patient. Each panel represents an individual patient, with colors indicating the dosing regimen. Line denotes ADA status, and black plus signs indicate the time point of ADA detection. We do not observe an impact of ADA detection on response.

We have included the below text (*green font)* in the Results Pharmacokinetics " section" of the manuscript.

Immunogenicity was assessed using a three-tiered approach comprising screening, confirmatory, and titer assays. Based on available data as of the data cut-off date, DYP688 demonstrates low immunogenicity, with an anti-drug antibody (ADA) incidence of less than 10%.

6. The safety of DYP688 is encouraging, but it is curious that PMEL expressed by normal melanocytes in the skin showed no toxicity. Were any skin punch biopsies performed? Is there evidence that the drug may sequester in the skin.

Author response: Thirteen patients (19.7%) experienced nonserious dermatologic toxicities (all ≤ Grade 2) considered related to study treatment. These included three events of vitiligo (4.5%), all reported as Grade 1. Although no punch biopsies were performed, no confirmatory documentation is available for these cases.

The generally low incidence of skin toxicity may be partly explained by the fact that normal melanocytes do not carry GNAQ/GNA11 mutations and therefore would not be expected to exhibit meaningful susceptibility to DYP688. This contrasts with T cell–mediated mechanisms seen with tebentafusp, in which vitiligo is more frequently observed due to targeted destruction of melanocytes. In the present study, even if the payload were to reach the skin, melanocytes are unlikely to rely on GNAQ signaling, which could contribute to the minimal cutaneous effects observed.

The absence of significant dermatologic toxicity may therefore be viewed as supportive evidence of targeting of payload with activity selective in tumor cell context. .

We have included the data in the "Results, Safety" section of the manuscript.

Updated manuscript text (*green font*): TRAEs of any grade were reported in 60 (90.9%) patients. All TRAEs were ≤ grade2 severity, except for five grade 3 events: hypotension, asymptomatic hypercalcemia, anemia, increased gamma-glutamyl transferase (GGT) and decreased lymphocyte count. The most common TRAEs (all grades, all doses) were hypercalcemia (n=19, 28.8%), dry mouth, fatigue (n=15 each, 22.7%), peripheral edema (n=12, 18.2%), anemia (n=10, 15.2%), increased aspartate aminotransferase, constipation (n=8 each, 12.1%), increased alanine aminotransferase, epistaxis, nasal congestion (n=7 each, 10.6%), asthenia, hypophosphatemia, nausea and thrombocytopenia (n=6 each, 9.1%). *Dermatologic TRAEs were infrequent and nonserious, with all events reported as Grade ≤2. Vitiligo was observed in three patients (4.5%), all Grade 1.* TRAEs of any grade reported in ≥5% of patients in treatment groups 12,16 and 24 mg/kg Q2W, 12 and 16 mg/kg QW, and all patients are shown in Fig.1.

7. Please add statistical testing to support that "mutations in BAP1 but not SF3B1 were associated with lesser best percentage change in tumor diameters" as described in Supplementary Fig. 5.

Author response: We thank the reviewer for this suggestion. In response, we have added statistical comparisons to Supplementary Figure 5 (Supplementary Fig. 6 in revised draft) to formally assess differences in best percentage change across BAP1/SF3B1 mutation groups.

In addition, we have revised the accompanying text (*green font*) to more accurately reflect the data by emphasizing the exploratory nature of these analyses and the relative attenuation of response observed in tumors harboring BAP1 mutations, particularly in rare combination with SF3B1 mutations, rather than implying a definitive or independent effect of BAP1 mutation alone.

Revised text: Results: *Additionally, exploratory analyses suggest differences in the distribution of best percentage change in tumor diameters across BAP1/SF3B1 mutation groups, with attenuated reductions observed in tumors harboring BAP1 mutations, particularly in rare combination with SF3B1 mutations (**Supplementary Fig. 6**).*

Revised text: Discussion: *Exploratory analyses suggested that tumors harboring BAP1 mutations detected in ctDNA at baseline, particularly in rare combination with SF3B1 mutations, tend to exhibit attenuated depth of response, although substantial overlap was observed across mutation groups. These findings should be interpreted cautiously given the limited sample size and the analytical constraints of ctDNA-based mutation assessment. Nevertheless, they are directionally consistent with prior literature showing that germline or tissue-based BAP1 alterations are associated with more aggressive disease biology in uveal melanoma, including earlier onset, larger tumors, increased ciliary body involvement, and higher metastatic risk. Indeed, BAP1 mutations have been associated with significantly reduced survival and an increased risk of metastasis compared with BAP1-intact tumors*

Revised Supplementary figure 6.

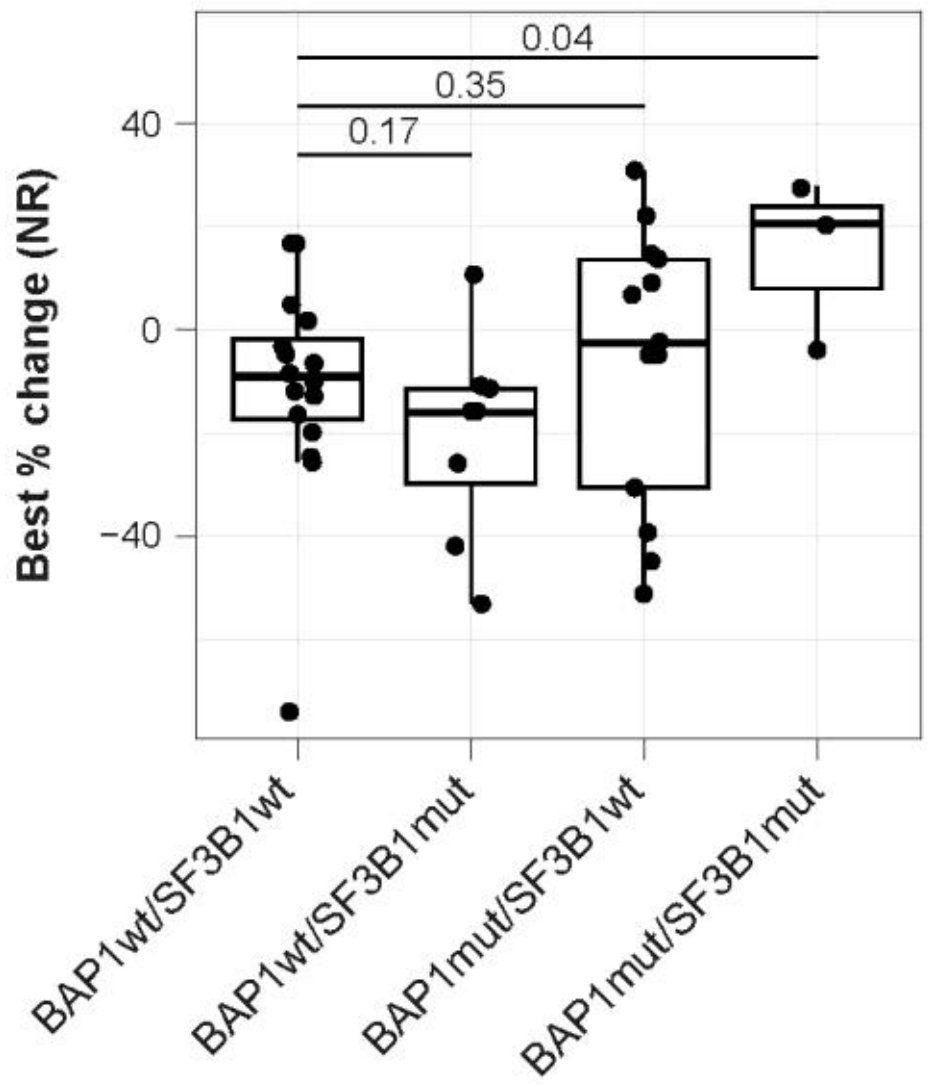

Supplementary Fig. 6. Box plot illustrates the relationship between baseline BAP1/SF3B1 mutation (mut) status in ctDNA and best percentage change in nadir response (NR; only a single patient showed EIF1AX mutation here, precluding the inclusion of EIF1AX mutation status as additional columns in the plot). In particular in rare combination with SF3B1 mutation, BAP1 mutation appears to be associated with attenuated depth of response, with the double-mutant group showing a shift toward tumor growth compared with BAP1/SF3B1 wildtype tumors. We included only patients with a baseline ctDNA sample showing a GNAQ/11 mutation, ensuring that all selected patients had detectable ctDNA and minimizing issues related to low detection rate in ctDNA from patients with low levels of DNA shedding. A two-sample Wilcoxon rank sum test was used to derive p-values for each comparison depicted by the horizontal lines. N = 16 (BAP1wt/SF3B1wt), 9 (BAP1wt/SF3B1mut), 13 (BAP1mut/SF3B1wt), and 3 (BAP1mut/SF3B1mut.

Reviewer #2 (Remarks to the Author):

In this article, Carlino et al., demonstrate the activity of DYP688, a novel double-target anti-PMEL ADC for metastatic uveal melanoma and other similar tumours. In brief, the article is novel in both describing the structure and activity of the ADC and is a welcome addition to the armamentarium for uveal melanoma. The methodology is appropriate and my comments below are partly designed to improve the impact of the publication across a number of issues

MAJOR
1. The nature of the high rates oh hypercalcemia are unclear from the text - is this something that was expected from the preclinical data?

Author response: Thank you for the comment. The episodes of hypercalcemia are hypothesized to result from potential effects of DYP688 on the calcium-sensing receptor (CaSR) pathway. CaSR normally signals through the G-protein subunit Gα11 (encoded by GNA11) as part of its physiological regulation of serum calcium levels. Disruption of this signaling pathway could theoretically shift the calcium-sensing set point and thereby dysregulate calcium homeostasis. Elevations in serum calcium and magnesium are well-documented consequences of impaired CaSR signaling. Notably, loss-of-function mutations in

GNA11 cause familial hypocalciuric hypercalcemia type 2 (FHH2), characterized by hypercalcemia, hypocalciuria, and often mild hypermagnesemia. Conversely, gain-of-function mutations in GNA11 lead to autosomal dominant hypocalcemia type 2 (ADH2). These established genetic disorders illustrate how altered Gα11-mediated CaSR signaling can raise or lower serum calcium depending on whether the functional change reduces or enhances receptor signaling.

Revised discussion text (*green font*): DYP688 is well tolerated with most AEs considered related to the study drug were Grade ≤2 and no MTD was determined. Only five grade 3 events reported: hypotension (lasting one day at 24 mg/kg Q2W), asymptomatic hypercalcemia, anemia, increased GGT, and decreased lymphocyte count. *The episodes of hypercalcemia are hypothesized to result from potential effects of SDZ475 on the calcium-sensing receptor (CaSR) pathway, which signals through Gα11 to regulate calcium homeostasis (41).*

Reference added:

41.Nesbit, M. A., Hannan, F. M., Howles, S. A., Babinsky, V. N., Cranston, T., Rust, N. et al. Mutations affecting G-protein subunit α11 in hypercalcemia and hypocalcemia. N. Engl. J. Med. 368, 2476–2486 (2013). https://doi.org/10.1056/NEJMoa1300253.

The relatively good tolerance is likely related to the PK characteristics of DYP688. DYP688 demonstrated a dose-dependent increase in systemic PK exposure (AUC) in all analyses, including total antibody….

2. given the interest in the field, coudl the authors comment in the article if any responses were seen in any primary lesions that may have been still in situ?

<u>Author response:</u> Because the standard of care for uveal melanoma involves achieving local control of the primary tumor—typically through radiotherapy and/or surgery—patients had already received treatment for their primary lesion prior to trial enrollment. As a result, assessment of DYP688 activity in primary tumors was not feasible in this study, as the dataset included almost exclusively patients with previously treated primaries and documented metastatic disease.

We have revised the "Methods, Participants" section of manuscript (*green font*):

Participants

The study recruited adult patients with mUM and other GNAQ/11-mutant melanomas. Eligible patients had an Eastern Cooperative Oncology Group Performance Status score of ≤1, with histologically or cytologically confirmed metastatic disease measurable by RECIST v1.1. Patients with mUM were either treatment-naive or had progressed on their most recent prior therapy. Patients with advanced cutaneous or other melanoma with GNAQ/11 mutations had to have progressed on all available standard-of-care treatments. *The majority of patients had received prior local treatment (radiotherapy and/or surgery) for the primary uveal melanoma in accordance with standard of care; therefore, DYP688 treatment response in primary lesions could not be evaluated.*

3. There appears to be an overemphasis on the prognosis of the BAP1 mutations in the text - the supporting figure , supplemental figure 5 isn't statistically justified and it appears there is a lot of overlap. There is also the issue that some BAP1 genes may be methylated or limited analytically ? VUS ? other by the ctDNA assay used suggesting that some of the BAP1 WT patients may in fact have mutated proteins

<u>Author response:</u> We thank the reviewer for this thoughtful and important comment. In response, we have taken several steps to address these concerns. We have added statistical comparisons to Supplementary

Figure 5 to better contextualize the observed differences and to make clear the degree of overlap between groups.

We have also now revised and softened the accompanying text to avoid over-emphasizing the prognostic value of BAP1 mutations detected in ctDNA and now describe these findings as exploratory and descriptive rather than definitive.

The revised Supplementary Figure 5 (Supplementary Fig 6 in revised draft) and corresponding legend have been included in response to the comment #7 from reviewer # 1

4. Can the authors provide information (perhaps in a supplement) just for the uveal patients on the study as these are certainly the patients with the greatest unmet need

Author response:

We appreciate the reviewer's suggestion. We have added a supplementary table that provides efficacy outcomes specifically for patients with uveal melanoma, including objective response rate and disease control rate. These data have been incorporated as Supplementary Table 3, highlighting the clinical activity of DYP688 in this population with high unmet medical need.

We have included the below text (*green font*) in the Results , 'Efficacy" section" of the manuscript.

Revised text: Progressive disease (PD) was seen in 11/66 (16.7%) patients and stable disease (SD) in 41/66 (62.1%) patients. *Efficacy outcomes for UM patients are summarized in* ***Supplementary Table 3***.

*Supplementary Table 3. Efficacy outcomes in uveal melanoma patients**

	4 mg/kg Q2W N=5 n (%)	*8 mg/kg Q2W N=12 n (%)*	*12 mg/kg Q2W N=9 n (%)*	*16 mg/kg Q2W N=13 n (%)*	*24 mg/kg Q2W N=10 n (%)*	*12 mg/kg QW N=5 n (%)*	*16 mg/kg QW N=6 n (%)*	*All UM patients N= 60 n (%)*
Number of evaluable patients	*5*	*12*	*9*	*13*	*10*	*5*	*6*	*60*
Patient with measurable disease at baseline	*5 (100)*	*12 (100)*	*9 (100)*	*13 (100)*	*10 (100)*	*5 (100)*	*6 (100)*	*60 (100)*
Patient with non-measurable disease only at baseline	*0*	*0*	*0*	*0*	*0*	*0*	*0*	*0*
Best overall response - n (%)								
Partial Response (PR)	*0*	*2 (16.7)*	*2 (22.2)*	*5 (38.5)*	*0*	*2 (40.0)*	*1 (16.7)*	*12 (20.0)*
Stable Disease (SD)	*2 (40.0)*	*8 (66.7)*	*6 (66.7)*	*7 (53.8)*	*7 (70.0)*	*2 (40.0)*	*5 (83.3)*	*37 (61.7)*
Progressive Disease (PD)	*3 (60.0)*	*1 (8.3)*	*1 (11.1)*	*1 (7.7)*	*3 (30.0)*	*1 (20.0)*	*0*	*10 (16.7)*
Not Evaluable (NE)	*0*	*1 (8.3)*	*0*	*0*	*0*	*0*	*0*	*1 (1.7)*
Overall Response Rate (ORR: CR+PR)	*0*	*2 (16.7)*	*2 (22.2)*	*5 (38.5)*	*0*	*2 (40.0)*	*1 (16.7)*	*12 (20.0)*

	4 mg/kg Q2W N=5 n (%)	8 mg/kg Q2W N=12 n (%)	12 mg/kg Q2W N=9 n (%)	16 mg/kg Q2W N=13 n (%)	24 mg/kg Q2W N=10 n (%)	12 mg/kg QW N=5 n (%)	16 mg/kg QW N=6 n (%)	All UM patients N= 60 n (%)
95% CI	(0.0,52.2)	(2.1,48.4)	(2.8,60.0)	(13.9,68.4)	(0.0,30.8)	(5.3,85.3)	(0.4,64.1)	(10.8,32.3)
Disease Control Rate (DCR: CR+PR+SD)	2 (40.0)	10 (83.3)	8 (88.9)	12 (92.3)	7 (70.0)	4 (80.0)	6 (100)	49 (81.7)
95% CI	(5.3,85.3)	(51.6,97.9)	(51.8,99.7)	(64.0,99.8)	(34.8,93.3)	(28.4,99.5)	(54.1,100.0)	(69.6,90.5)

Only subjects with disease of uveal melanoma.

**by local radiology review*

CI, confidence interval; CR, complete response; DCR, disease control rate; NE, not evaluable; ORR, overall response rate; PD, progressive disease; PR, partial response; QW, once weekly; Q2W, every 2 weeks; SD, stable disease.

5. the discussion Lines 296-300 doesn't mention the combination of PKC and MET inhibition, but rather only the more ineffectual PKC/AKT inhibition - its not clear why these are separated in the text

Author response: We thank the reviewer for this helpful observation. The manuscript text is now revised to incorporate the emerging evidence supporting PKC + MET inhibition-based strategies.

Revised text (*green font*): Data from previous studies have showed that MEK inhibition alone failed to mimic GNAQ silencing, whereas combined MEK and AKT inhibition induced synergistic cell death, underscoring the need to simultaneously block parallel survival pathways (33). Similarly, PKC inhibitors such as darovasertib and sotrastaurin have aimed to suppress MAPK signaling downstream of GNAQ/11, with variable efficacy and emerging resistance (36-37). *More recently, PKC inhibition in combination with the tyrosine kinase inhibitor, crizotinib, has been investigated as a means to deepen pathway suppression and overcome adaptive resistance. Crizotinib has activity against ALK, ROS1, and MET (38), and while the precise contributors to clinical benefit remain uncertain, preclinical studies suggest that the addition of a tyrosine kinase inhibitor to PKC blockade more effectively attenuates compensatory signaling through MAPK and PI3K pathways, resulting in enhanced apoptotic responses compared with PKC inhibition alone. Consistent with this, early clinical experience with darovasertib plus crizotinib has indicated improved tumor shrinkage and more durable disease control compared with single agent PKC inhibition, supporting the rationale for targeting both PKC- dependent signaling and MET- -mediated pathways (39).* Epigenetic modulation using-histone deacetylase inhibitors in combination with MEK inhibitors has shown promise in overcoming such resistance (36). Compared to these approaches, DYP688 offers a more direct and potentially durable strategy by targeting the root oncogenic driver, thereby attenuating multiple downstream pathways simultaneously without the toxicity challenges typically seen with targeted therapy combinations.

References added:

38 Blackhall F, Cappuzzo F. Crizotinib: from discovery to accelerated development to front-line treatment.

Annals of Oncology. 2016;27(Suppl 3):iii35–iii41. doi:10.1093/annonc/mdw304

39 Mckean, M. et al. 1081O ctDNA reduction and clinical efficacy of the darovasertib + crizotinib (daro + crizo) combination in metastatic uveal melanoma (MUM). Ann. Oncol. 34, S651 (2023).

6. especially because of the de facto comparison (lines 337-340) the authors should include somewhere in the article, the effectiveness of the combination in patients pre-treated with Darovasertib/Crizotinib. This could go in the figure 3a (similar to the Tebentafusp) for example

Author response: We thank the reviewer for this suggestion. We have now included the effectiveness of the DYP688 in patients pre-treated with darovasertib only or darovasertib and crizotinib in Figure 3a and 3b, similar to the tebentafusp-pretreated patients.

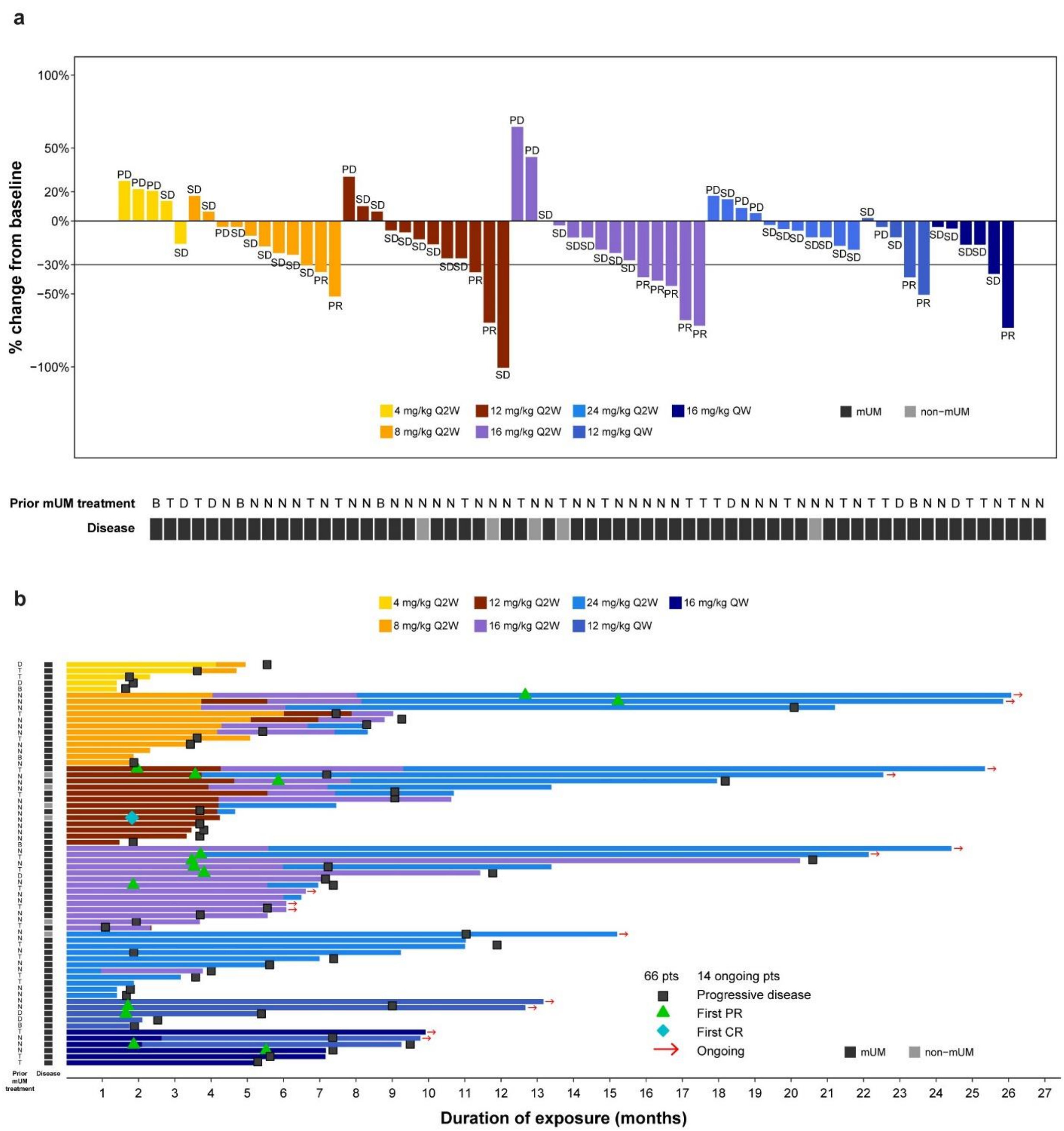

Fig. 3. (a) Percentage change from baseline in sum of the longest diameters by radiology review. The percentage of tumor size reduction is illustrated on the y axis. Every bar is one patient. The horizontal line represents at least a 30% decrease in the sum of the diameters of target lesions in patients. 1 patient was non-evaluable at the only post baseline scan as one of the target lesions could not be measured and 1 patient had non-measurable disease at baseline.(b) Duration of treatment. The x-axis denotes time in months, while each horizontal bar on the y-axis represents a single patient. mUM, metastatic uveal melanoma; PD, progressive disease; PR, partial response; pts, patients; QW, once weekly; Q2W, every 2 weeks; SD, stable disease*; B, darovasertib/crizotinib and tebentafusp prior treatment; D, darovasertib only or darovasertib and crizotinib prior treatment; N, no prior darovasertib/ crizotinib or tebentafusp treatment; T, tebentafusp prior treatment.*

7. Usually articles such as this one, end with a plan for future development. This detail is sorely missing. Can this be added?

Author response: Thank you for this helpful suggestion. We have updated (*green font*) the concluding statement in the manuscript outlining the intended future development of DYP688. Separately, we would like to emphasize that Novartis is actively exploring opportunities to advance the clinical development of DYP688; however, details regarding partnership discussions remain confidential at this time.

Revised text: The observed activity and favorable safety profile support further investigation of DYP688 as both a single agent and, potentially, in combination regimens for patients with mUM.

8. Did the post samples acquired highlight any possibilities for resistance pathways? lines 250-252. IN particular, MEK pathway activation such as was foound with Darovasertib alone

Author response: The on-treatment samples were collected 16-18 days after study start. We do not expect acquired resistance mechanisms to be evident this early in the treatment cycle, and analysis of on-treatment samples did not reveal activation of MAPK/MEK signaling as an apparent resistance mechanism: on-treatment MPAS GSVA scores were generally reduced relative to baseline, with overlapping distributions between tumors that shrank (in terms of best percent change in size) compared to those that did not. Please note that the MAPK pathway activity score (MPAS) was developed specifically to quantify the effect of MEK or BRAF inhibition on MAPK targets, but not other inhibitors targeting PI3K, AKT and other pathways (https://doi.org/10.1038/s41698-018-0051-4). Analysis of ctDNA from patients at later timepoints did not indicate the emergence of potential resistant mutations in GNAQ/11 (Supplementary Figure 7).

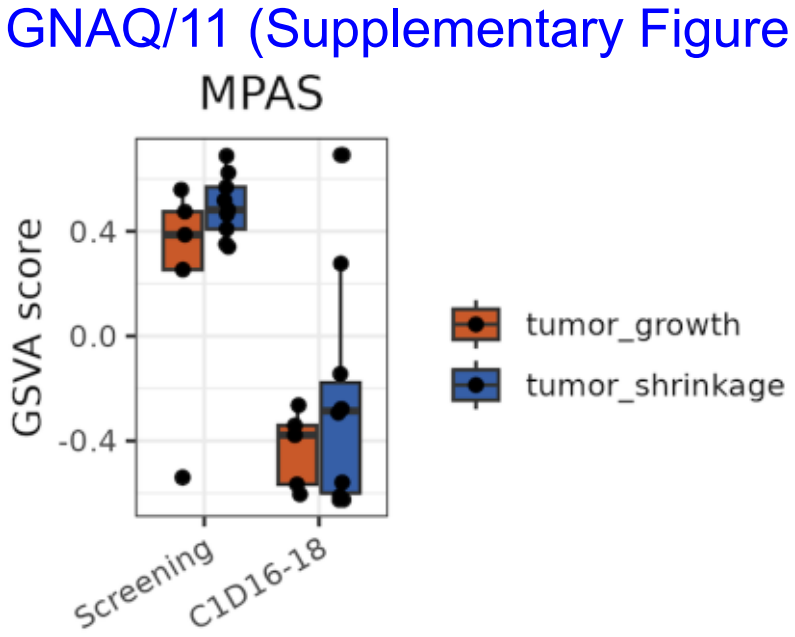

MINOR
1. Reference 9 is an old reference outlining the relapse risk of melanomas, please use a more contemporary reference that may suggest that the relapse risk is indeed likely higher when patients are followed with contemporary imaging

Author response: We thank the reviewer for this helpful suggestion. We agree that relapse (metastatic) risk estimates from older uveal melanoma cohorts (for example, the COMS era) likely underestimate

current detection rates because modern follow up more routinely uses sensitive cross-sectional imaging, such as contrast enhanced liver MRI or CT, and sometimes PET or CT. Recent consensus statements and data sets indicate that contemporary surveillance detects more metastatic events and smaller liver metastases than older ultrasound or exam-based protocols.

We have therefore included along with the COMS era citation. contemporary evidence and revised the text (*green font*) to reflect current practice.

Revised text: "Despite effective clinical management of primary UM, a substantial proportion of patients ultimately develop distant metastases. Historical reports have cited metastatic rates of approximately 25% to 34% within 5 to 10 years following diagnosis (9). *However, modern risk-adapted surveillance strategies that incorporate high-sensitivity cross-sectional imaging detect metastatic recurrences earlier and at smaller volumes than older approaches, suggesting that earlier estimates may have under-detected metastatic disease. Consequently, the true lifetime risk is likely closer to up to 50%, depending on tumor genetics and other prognostic features" (10-11).*

References added:

10. Yeşiltaş YS, Zabor EC, Wrenn J, Oakey Z, Singh AD. Surveillance for Metastasis in High-Risk Uveal Melanoma Patients: Standard versus Enhanced Protocols. Cancers. 2023; 15(20):5025. https://doi.org/10.3390/cancers15205025.

11. Alban J, Bowen RC, Reichstein DA, McKean M, Lutzky J, Weis E, Carvajal RD, Dulka S, Morse BG, Butler MO, et al. Metastatic Uveal Melanoma Surveillance: A Delphi Panel Consensus. Cancers. 2026; 18(1):121. https://doi.org/10.3390/cancers18010121

2. Line 78, it is not clear if the hotspot mutations mentioned are in the GNAQ or GNA11 from the way the text is written. please rewrite.

Author response: We thank the reviewer for this helpful comment. We agree that the original sentence may have been ambiguous regarding whether the hotspot mutations occur in GNAQ, GNA11, or both. We have therefore revised the text (*green font*) as:

"Approximately 85%–90% of UMs harbor mutually exclusive activating mutations in either GNAQ or GNA11, most commonly affecting the Q209 (~80%) or R183 hotspots."

3. Especially in the introduction there is switching between the PMEL term and using gp100 as an equivalent. Please harmonise

Author response: We thank the reviewer for this valuable comment. We agree that the alternating use of the terms PMEL and gp100 in the introduction is confusing, and we have revised the text to ensure consistent terminology throughout. In the revised version, we use "PMEL (PMEL17/gp100)" at first mention and thereafter maintain one harmonized term (PMEL) to improve clarity and readability.

We have revised the text (green font) as

To overcome the limitations of systemic Gq/11 inhibition, we explored tumor-specific payload delivery targeting ~~gp100~~ PMEL (PMEL17/gp100), a melanocyte lineage-specific protein that is highly expressed in both cutaneous and UMs, as well as in normal melanocytes of the retina, skin, and substantia nigra (17-18). PMEL is expressed mainly in the melanosome, an organelle within melanocytes involved in the synthesis and storage of melanin and is also found on the cell surface (19).

The clinical relevance of ~~gp100~~ PMEL as a therapeutic target has been validated by the success of tebentafusp, a bispecific ~~gp100~~ PMEL × CD3 T-cell engager, which has demonstrated a significant OS

benefit in human leukocyte antigen (HLA)-A*02:01-positive patients with metastatic UM (mUM) and has received regulatory approval for this indication (20-21). However, its mechanism of action relies on the presence of HLA allele to present the ~~gp100~~ PMEL peptide to T cells and so its use is limited to HLA-A*02:01-positive patients, excluding over half of the mUM population (20), who currently have no approved systemic therapies for metastatic disease (20-23).

The efficacy of tebentafusup highlights the potential of ~~gp100~~ PMEL-targeting to selectively engage tumor cells while sparing normal tissues, paving the way for novel approaches such as antibody-drug conjugates (ADCs) that deliver cytotoxic agents directly to ~~gp100~~ PMEL-expressing tumor cells.

DYP688 is a first-in-class PMEL ~~(PMEL17/gp100)~~ targeting ADC composed of a highly selective humanized cysteine-engineered PMEL monoclonal antibody, conjugated at a drug-antibody ratio (DAR) of 2 on its engineered sites to SDZ475, via the maleimide-based stable VaLCit linker (16), which is a potent, selective and targeted Gq/11 inhibitor (15). DYP688 is an ADC with a biology-matched payload that is first "localized to" PMEL (17-18). Upon ADC binding to PMEL on the target cells and internalization, the linker is cleaved to release the GQ/11 inhibitor SDZ475, which inhibits GNAQ/GNA11 mediated oncogenic signaling, resulting in dose-dependent apoptosis (16). DYP688 showed dose-dependent anti-tumor activity across a panel of mUM xenografts, including mouse models expressing various levels of PMEL, and patient-derived xenografts of mUM. Preclinical toxicology studies showed DYP688 was well tolerated with minimal and reversible clinical and histopathological findings, supporting the notion that DYP688 can specifically target GNAQ/GNA11-mutant melanocytic tumors with a favorable safety profile in humans (16).

4. Line 106, nature of SDZ475 already mentioned earlier ? need to repeat

Author response: We appreciate the reviewer's comment. The nature and description of SDZ475 is introduced earlier in the manuscript; however, we had retained the brief reiteration here to ensure clarity when describing the full composition of DYP688 as an ADC. To avoid redundancy, we have now streamlined the sentence so that SDZ475 is referenced only in relation to its conjugation within DYP688.

We have revised (*green font*) as follows: ~~DYP688 is a first-in-class PMEL (PMEL17/gp100) targeting ADC composed of a highly selective humanized cysteine-engineered PMEL monoclonal antibody, conjugated at a drug-antibody ratio (DAR) of 2 on its engineered sites to SDZ475, via the maleimide-based stable VaLCit linker (16), which is a potent, selective and targeted Gq/11 inhibitor (15)."~~ *DYP688 is a first in- class PMEL targeting ADC composed of a highly selective humanized cysteine- -engineered PMEL monoclonal antibody, conjugated at a drug-antibody ratio (DAR) of 2 via a maleimide -based stable VaLCit linker (16) to the Gq/11-inhibitory payload SDZ475 (15).*

5. line 195- IM not clear why these fractions were not converted into fractions (the first one was only?)

Author response: We thank the reviewer for the comment. To ensure consistency, we have now presented all dose level results uniformly as fractions (responders/total patients) and- percentages.

Revised text (*green font*): Confirmed objective responses were seen in 1*3*/66 (19.7%) patients with *2*/12 (*16.7%)* at 8 mg/kg Q2W, 3/13 (*23.0%*) at 12 mg/kg Q2W, 5/14 (*35.7%*) at 16 mg/kg Q2W, 2/5 (*40.0%*) at 12 mg/kg QW and 1/6 (*16,6%*) at 16 mg/kg QW, with evidence of deepening response over time in some patients.

6. line 196 - the protocol deviation was that the patient was enrolled with non-RECIST disease not that they had cutaneous melanoma - please rewrite text?

Author response: We thank the reviewer for the clarification. The text has been corrected to reflect that the protocol deviation pertained to the patient being enrolled with non-measurable (non-RECIST) disease, not due to having cutaneous melanoma. The sentence has been revised accordingly (*green font*)

~~One patient with cutaneous melanoma (enrollment was a protocol deviation) and non-measurable disease per Response Evaluation Criteria in Solid Tumors (RECIST) v1.1 at baseline achieved a confirmed complete response (CR) at 12 mg/kg Q2W.~~ *One cutaneous melanoma patient with non-measurable disease per Response Evaluation Criteria in Solid Tumors (RECIST) v1.1 at baseline (enrolled outside protocol-specified criteria) achieved a confirmed complete response (CR) at 12 mg/kg Q2W.*

7. it is unclear what the authors mean by "dynamic interaction" - they are referring to the hypothesis on line 236. Could these be rewritten?

Author response: We thank the reviewer for this comment. We have revised the text to clarify the intended meaning. The term "dynamic interaction" has been removed and text revised.

Revised text (*green font*): " *At lower DYP688 doses, a rebound in unbound PMEL was observed at C1D15 (Fig. 4b) indicating that there is insufficient DYP688 to bind and engage the newly synthesized and/or shed PMEL".*

8. How is "responded to any prior therapy" defined? does it include SD > 12 weeks?

Author response: Responded to any prior therapy" was defined according to RECIST-categories and only included patients with a Complete Response (CR) or Partial Response (PR). If Progressive Disease (PD) was observed within 2 months of initiating a treatment regimen, the best overall response was classified as PD. Patients whose best response to prior therapy was Stable Disease (SD) or Non-CR/Non-PD were classified as having SD as their best response. These response assessments were reported by the study sites based on patient medical records and local investigator assessment.

9 A graphic outlining the MOA of this drug and others in the class would be helpful for the readership

Author response: We thank the reviewer for the suggestion. We have now included a graphic outlining the mechanism of action (MoA) of DYP688, as supplementary figure 1.

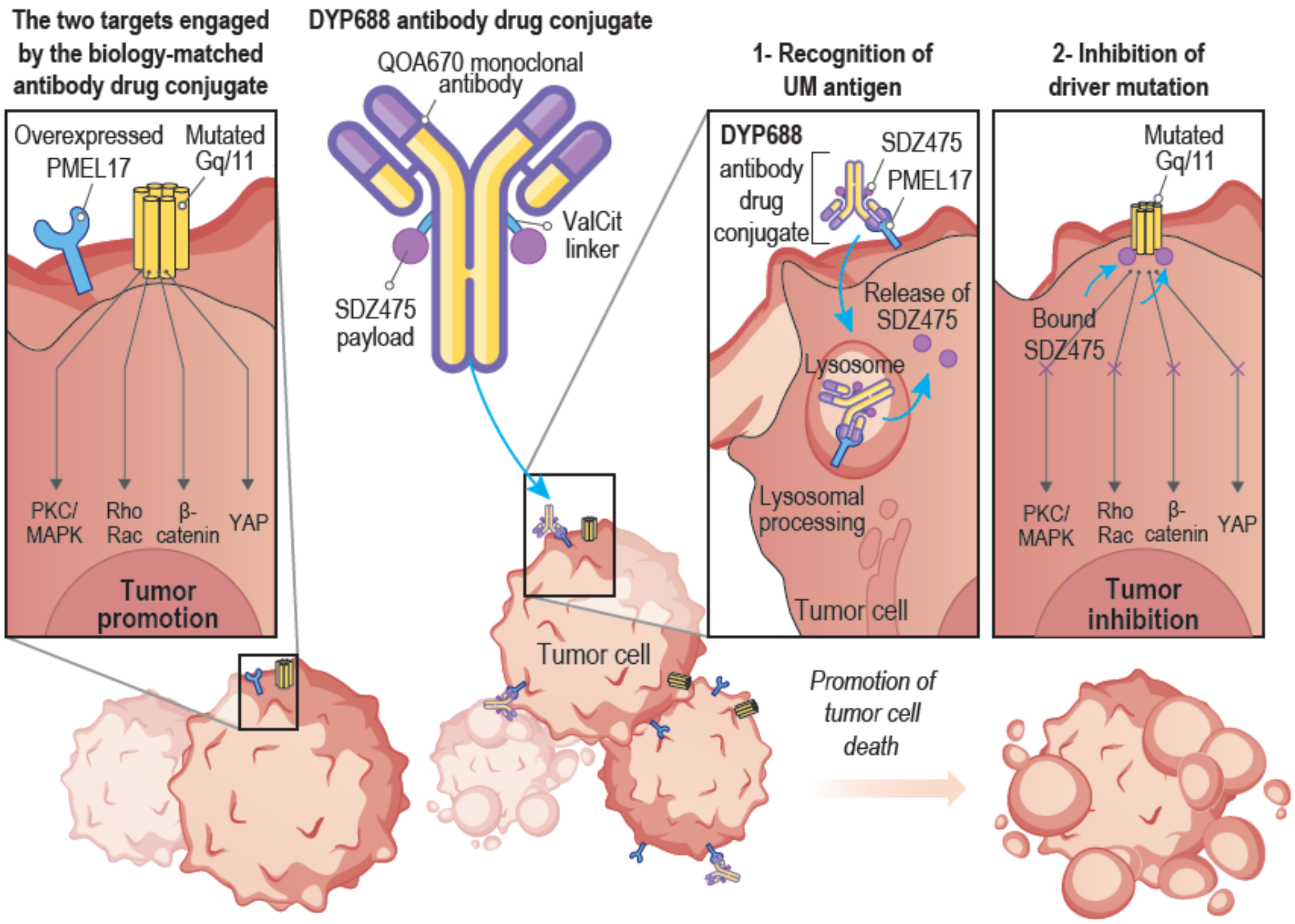

Supplementary Figure 1. Mechanism of action of DYP688, a PMEL17-targeting antibody–drug conjugate.

The biology matched- antibody–drug conjugate DYP688 engages two targets in uveal melanoma: overexpressed PMEL17 and mutant Gq-/11. SDZ475 is active against both mutant and wildtype Gαq/11 proteins; however, its antitumor effects are observed- selectively in tumor cells that are dependent on Gαq/11 signaling for proliferation (i.e., those harboring driver mutations), while normal wildtype GNAQ/11 cells are largely unaffected.

(Left) Tumor cells overexpress PMEL17 and harbor activating Gq/11 mutations, which drive oncogenic signaling through PKC/MAPK, Rho/Rac, β-catenin, and YAP pathways, promoting tumor growth. (Center) DYP688 comprises the QOA670 monoclonal antibody conjugated via a ValCit linker to the SDZ475 payload. Upon binding PMEL17 on tumor cells, the antibody–drug conjugate is internalized and undergoes lysosomal processing, releasing SDZ475. (Right) Released SDZ475 binds Gq/11 and inhibits downstream oncogenic signaling, resulting in tumor growth suppression and promotion of tumor cell death. β-catenin, beta-catenin; MAPK, mitogen-activated protein kinase; PKC, protein kinase C; PMEL17, pre melanosome protein 17; Rac, rac GTPase; Rho, rho GTPase; UM, uveal melanoma; YAP, Yes-associated protein.

Reviewer #3 (Remarks to the Author):

This phase 1 clinical trial reports describes a novel antibody-drug conjugate engineered to deliver a Gq/11 inhibitor SDZ475 to uveal melanoma cells via PMEL binding. This is a novel therapeutic explored in a drug development space with significant patient need. The therapy was very well-tolerated with impressive efficacy. The data were presented clearly with extensive discussion of PK, PD, and biomarkers

above even an expectation in phase 1 studies. A few comments for review below.

Line 121: Should say "Patient characteristics"

Author response: Thank you for pointing this out. As suggested, we have corrected the text to read 'Patient characteristics' in the revised manuscript."

Revised subheading: Patient characteristics

Line 123: replace . after 2022 with ,

Author response: Thank you for your attention to detail. As suggested, we have replaced the period after '2022' with a comma in the revised manuscript.

*Revised text in the manuscript: "*A total of 66 patients were treated with DYP688 in the dose escalation part of this study, which began enrolling patients on 4 July 2022*,"*

Line 136: This sentence is redundant unless additional information should have been included.

Author response: We thank the reviewer for the comment. We agree that the sentence was redundant and have removed the sentence.

We have revised the text in the manuscript as:

*Revised text: "*The majority of patients (61, 92.4%) had received prior antineoplastic therapy; most of them were heavily pretreated with 39 (59.1%) patients having received ≥2 prior lines of systemic therapy: 48 (72.7%) had received prior checkpoint inhibitors, 22 (33.3%) had received prior tebentafusp, 9 (13.6%) prior darovasertib and 5 (7.6%) prior darovasertib and crizotinib*. ~~Other commonly received therapies included checkpoint inhibitors (n=48, 72.7%)."~~*

Line 138: How was best response to prior therapy determined? All treatments were on trial or was this investigator-assessment? Does this include prior liver-directed therapy?

Author response: The best response to prior therapy was obtained from historical data in the patients' medical records and was documented in the eCRF by the local investigators based on the historical local assessment. All treatment modalities were collected, including systemic therapies, as well as local therapies such as surgical interventions and radiation therapy.

Line 211: Was there a difference in response based on hepatic vs extrahepatic disease?

Author response: We do not observe a difference in the best tumor change from baseline for the lesions depending on the site of its location. We have included the data in the "Results, Efficacy" section of the manuscript.

Revised text (*green font*): Best percentage change from baseline in sum of longest target lesion diameters based on local radiology review is shown in Fig. 3a. Tumor size reduction was observed in 47/66 (71.2%) patients. *An analysis of best percent change in target lesion size by anatomical location showed tumor shrinkage across liver, lung, lymph node, and other sites, with no evidence of preferential response by organ site (data not shown).* Fourteen (21.2%) patients remained on treatment for ≥ 12 months (Fig.3b).

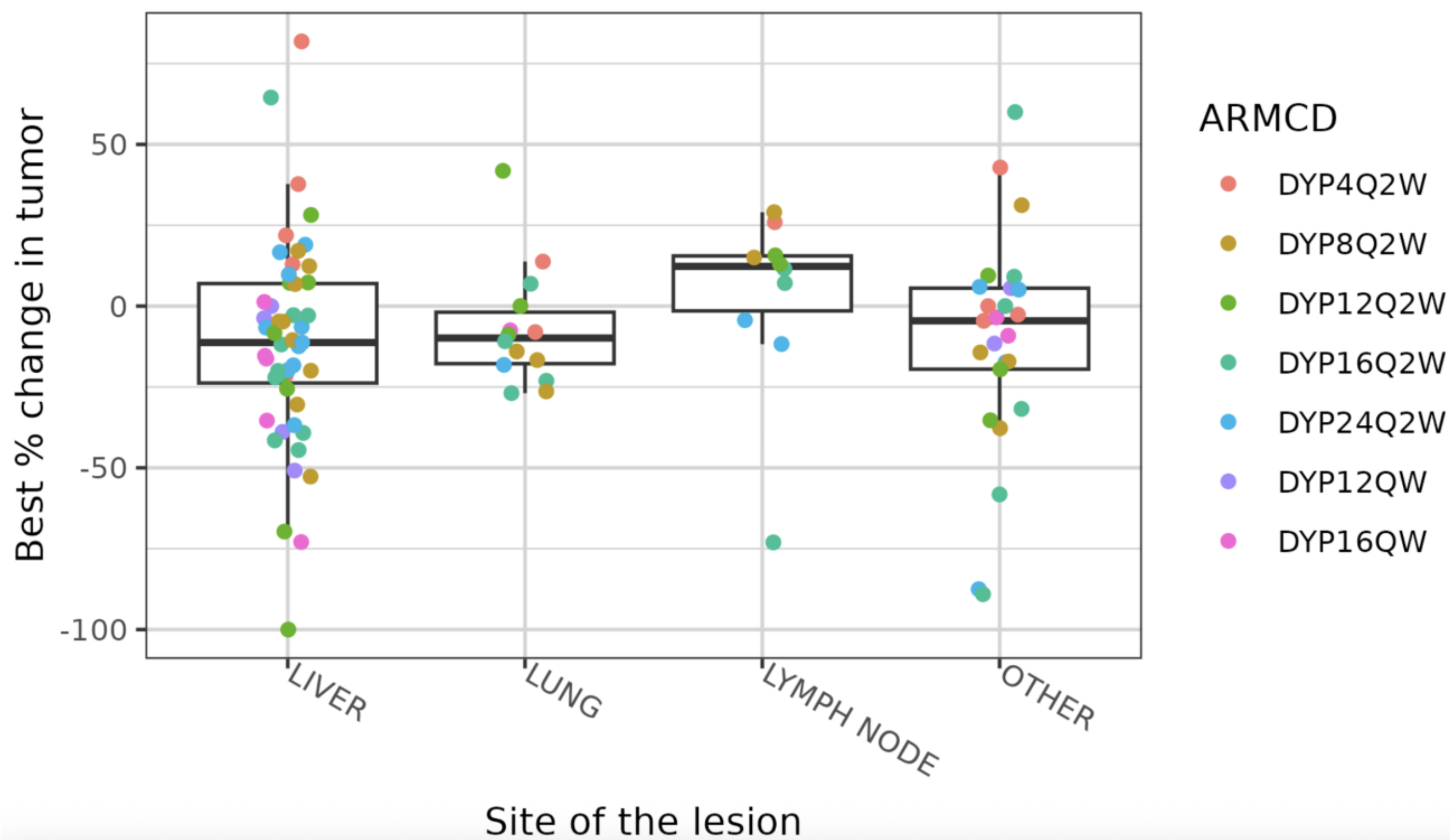

Figure. Best percentage change in target lesion size by anatomical location. Best percent change in tumor size from baseline is shown for individual target lesions stratified by lesion location (liver, lung, lymph node, and other). Each point represents an individual lesion, colored by DYP688 dosing regimen, and boxplots summarize the distribution within each site.

Line 295: Recommend rephrasing since reported response rate for darovasertib/crizotinib is higher than reported here despite downstream blockade. Also 74% of patients had confirmed disease progression with DYP688 so patients are also primarily progressing on this therapy, as with darovasertib/crizotinib, which indicates development of resistance regardless of sites of blockade.

Author response: "We thank the reviewer for this comment. We have revised the text as (*green font*):

Revised text: "Similarly, PKC inhibitors such as darovasertib and sotrastaurin have aimed to suppress MAPK signaling downstream of GNAQ/11, with variable efficacy and emerging resistance (34-35). Epigenetic modulation using-histone deacetylase inhibitors in combination with MEK inhibitors has shown promise in overcoming such resistance (36). Compared to these approaches, DYP688 offers a more direct and potentially durable strategy by targeting the root oncogenic driver, thereby attenuating multiple downstream pathways simultaneously without the toxicity challenges typically seen with targeted therapy combinations. *However, as with other targeted approaches, resistance can still develop."*

Line 339: Were any patients previously treated with darovasertib/crizotinib? Did they respond? Would be interested in comment based on mechanisms of action and potential pathways of resistance
Line 692: Please distinguish what +/- indicates.

Author response: We thank the reviewer for these comments. Patients previously treated with darovasertib only or darovasertib and crizotinib prior treatment; were included in the study, we have now included the effectiveness of DYP688 in patients pre-treated with darovasertib only or darovasertib and crizotinib prior treatment; in revised Figure 3a and 3b, similar to the presentation for tebentafusp-pretreated patients.

As shown in the updated waterfall plot, tumor shrinkage was observed in several patients previously exposed to darovasertib and/or crizotinib, indicating that DYP688 retains clinical activity in this setting.

Line 692: The “+/–” symbols have been removed from the figure to improve clarity. Prior treatment status is now annotated as follows: D, darovasertib only or darovasertib and crizotinib prior treatment; T, prior tebentafusp treatment; and N, no prior darovasertib, crizotinib, or tebentafusp treatment. The figure and legend have been updated accordingly.

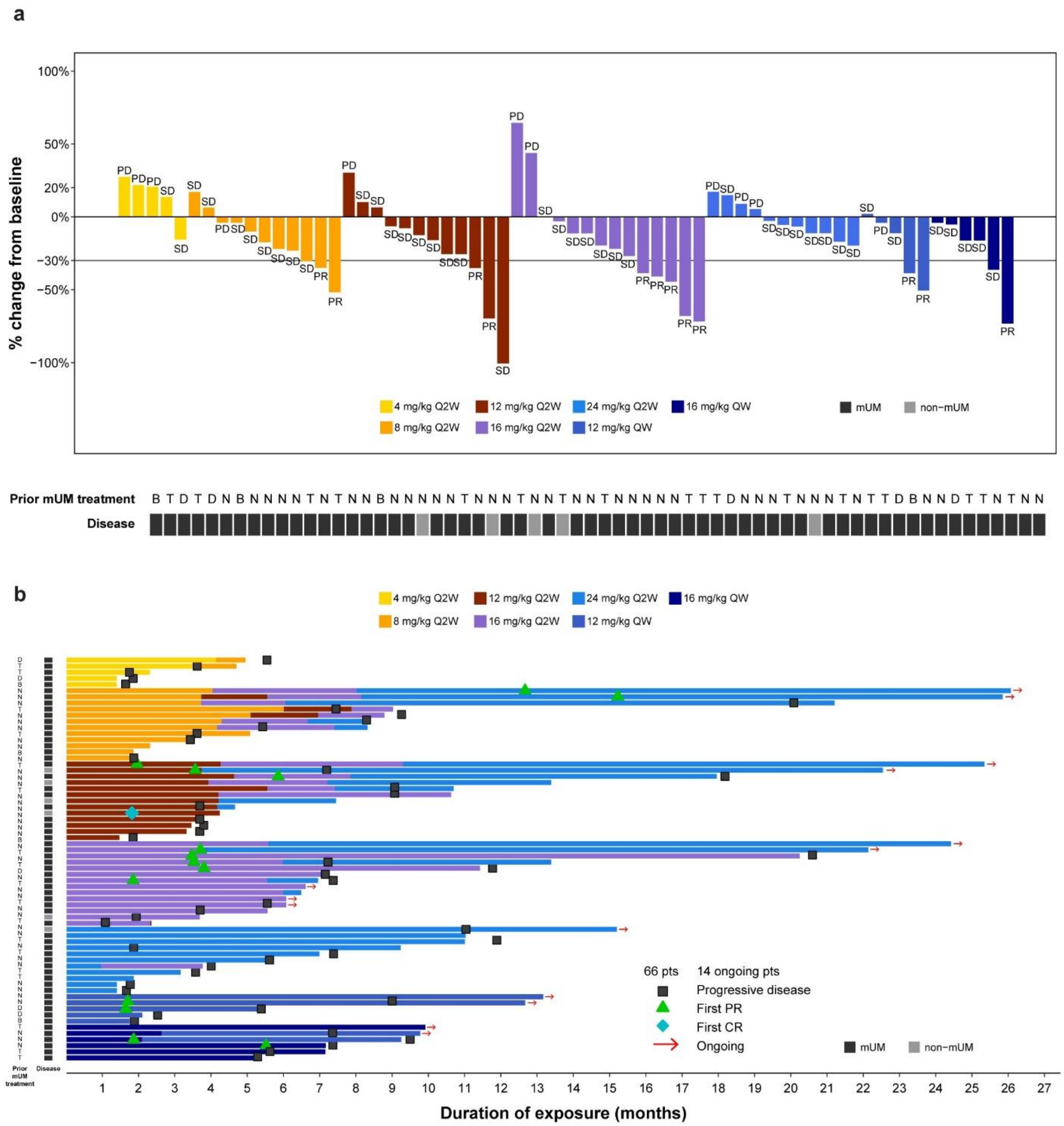

Fig. 3. (a) Percentage change from baseline in sum of the longest diameters by radiology review. The percentage of tumor size reduction is illustrated on the y axis. Every bar is one patient. The horizontal line

represents at least a 30% decrease in the sum of the diameters of target lesions in patients. 1 patient was non-evaluable at the only post baseline scan as one of the target lesions could not be measured and 1 patient had non-measurable disease at baseline.(b) Duration of treatment. The x-axis denotes time in months, while each horizontal bar on the y-axis represents a single patient. mUM, metastatic uveal melanoma; PD, progressive disease; PR, partial response; pts, patients; QW, once weekly; Q2W, every 2 weeks; SD, stable disease; *B, darovasertib/crizotinib and tebentafusp prior treatment; D, darovasertib only or darovasertib and crizotinib prior treatment; N, no prior darovasertib/ crizotinib or tebentafusp treatment; T, tebentafusp prior treatment.*

Line 694: Recommend changing color for first CR since difficult to distinguish between PR and CR on chart

Author response: Thank you for your valuable suggestion. Accordingly, we have changed the color for the first CR (light blue) to improve clarity and visual distinction in the revised figure.

Line 710: Was there a difference in baseline or on treatment in patients that received prior GNAQ/11 targeting therapy (daro/criz)?

Author response: There was no clear difference in the baseline PMEL expression or the on-treatment changes in patients that were naive or treated with prior GNAQ/11 targeted therapy. As seen shown below, there were not enough patients pre-treated with daro or criz in the bulk RNA-seq dataset for conclusion to be made (N = 2 pre-treated patients at screening and 3 at C1D16-18).

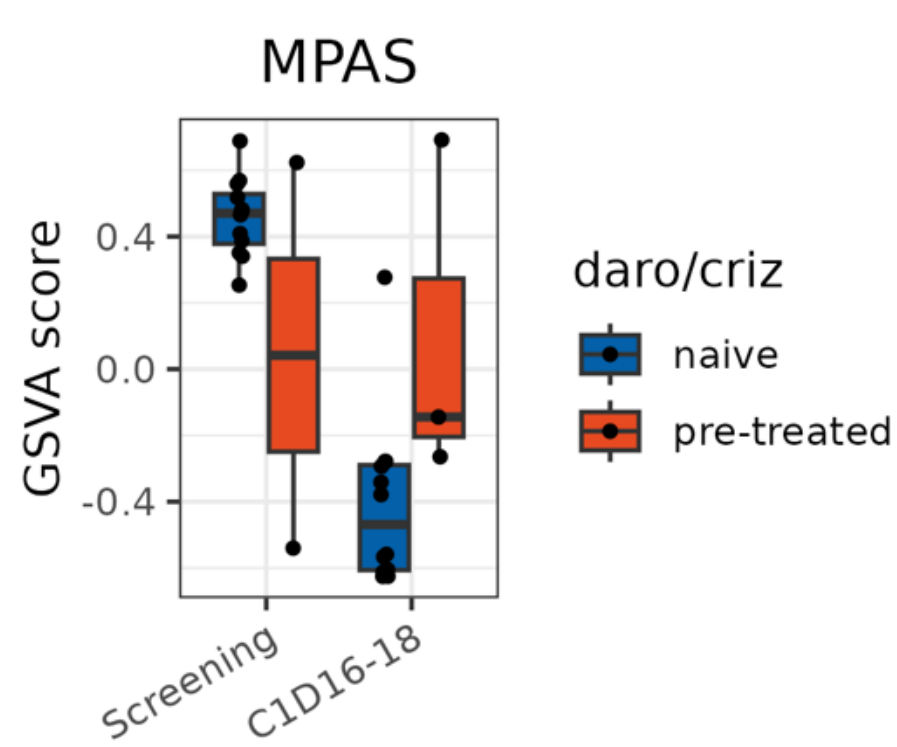

Line 859: A waterfall plot for change in ctDNA on treatment color-coded by response by imaging may be easier to interpret than supplementary figure 2.

Author response: We thank the reviewer for this suggestion. We have revised Supplementary Figure 2 (Supplementary Fig. 3 in revised draft) to a waterfall plot showing the change in ctDNA fraction from C1D1 to C3D1 for individual patients, ordered by magnitude of change and color-coded by best overall response. We believe this representation improves interpretability of on-treatment ctDNA changes in relation to imaging-based response:

We have now included below the revised Supplementary Figure 2 (Supplementary Fig. 3 in revised draft) and its legend in the manuscript.

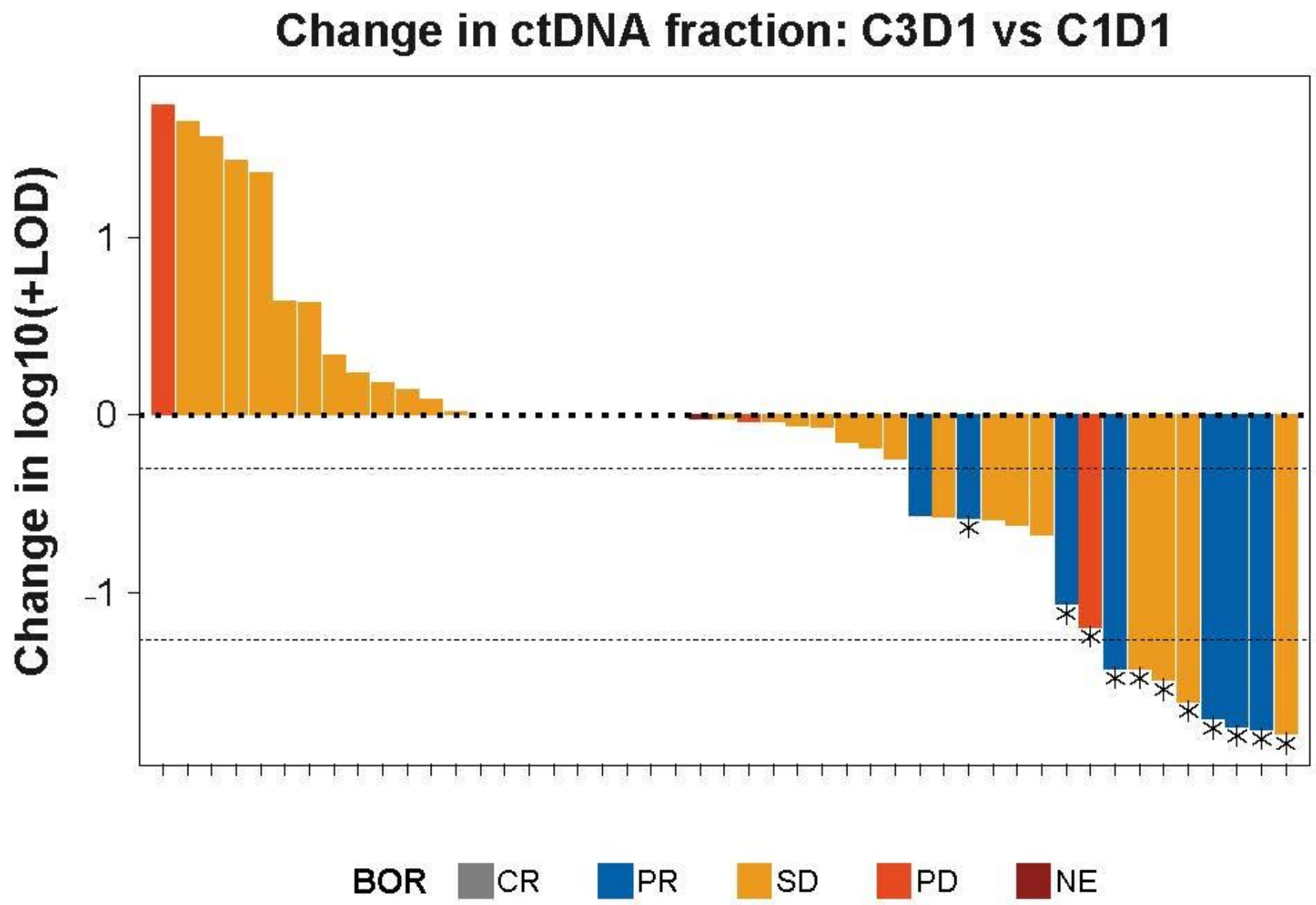

Supplementary Fig. 3. Partial responders more frequently exhibit reductions in ctDNA fraction compared with patients with stable disease or disease progression. Change in circulating tumor DNA (ctDNA) fraction from baseline to Cycle 3 Day 1. Waterfall plot showing the change in log10-transformed ctDNA fraction between Cycle 1 Day 1 (C1D1) and Cycle 3 Day 1 (C3D1) for individual patients with paired samples at both timepoints. ctDNA fractions were log10-transformed after addition of the assay limit of detection (LOD; 0.005) to enable inclusion of low or undetectable values. Bars are ordered by magnitude of change and colored by best overall response (BOR). Negative values indicate reduction in ctDNA fraction relative to baseline. The dotted horizontal line at 0 indicates no change from baseline; additional dotted lines indicate approximate 50% and 95% reductions in ctDNA fraction. Asterisks denote patients with ctDNA clearance at C3D1, defined as ctDNA fraction below the LOD with a reduction relative to baseline. Only patients with evaluable ctDNA measurements at both C1D1 and C3D1 are shown. The CR patient had undetectable baseline ctDNA with no change on treatment. C, cycle; D, day; ctDNA, circulating tumor DNA; BOR, best overall response; CR, complete response; PR, partial response; SD, stable disease; PD, progressive disease; NE, not evaluable; LOD, limit of detection.

Line 861: Recommend revising statement saying "treatment seems to".

Author response: We thank the reviewer for this request. In response, we have revised both the figure and its accompanying legend to remove speculative language. Specifically, Supplementary Figure 2 (Supplementary Fig 3 in the revised draft) has been updated from box plots to a waterfall plot depicting the change in ctDNA fraction from baseline (C1D1) to Cycle 3 Day 1 (C3D1) for individual patients with paired samples. The revised legend now uses descriptive language, stating that reductions in ctDNA fraction are more frequently observed among patients with partial response compared with those with stable or progressive disease, without implying causality or treatment effect. This revision more accurately reflects the observational nature of the data and improves clarity and interpretability.

The revised Supplementary Figure 2 (Supplementary Fig 3 in the revised draft) and corresponding legend have been included in response to the previous comment.

Line 867: Clarify how a patient is distinguished as "high or low"

Author response: Patients were stratified into ctDNA high or low at baseline depending on whether their ctDNA was lower or greater than or equal to the median value (ctDNA = 0.132) among all patients enrolled in the trial. This definition has now been added to the figure legend for clarity.

Revised figure legend (*green font*): Supplementary Fig. 3. (Supplementary Fig 4 in the revised draft) Kaplan-Meier curve illustrating the baseline circulating tumor DNA (ctDNA) as a prognostic marker of PFS. Patients with high ctDNA levels are represented by the red curve, while those with low ctDNA levels are shown in blue. *Patients were stratified into ctDNA high or low at baseline depending on whether their ctDNA was lower or greater than or equal to the median value (ctDNA = 0.132) among all patients enrolled in the trial.* A statistically significant difference in PFS is observed between the two groups (p = 0.00084). The table below the curves indicates the number of patients at risk at each time point for both ctDNA groups. Patients with a lower baseline ctDNA have a longer PFS. The group with a low ctDNA level comprises of patients with undetectable ctDNA and those with detectable ctDNA with a value less than the median (median ctDNA: 0.132). ctDNA, circulating tumor DNA; PFS, progression-free survival.0.132). ctDNA, circulating tumor DNA; PFS, progression-free survival.

Line 875: Supplementary figure 4 is difficult to demonstrate trends. I suggest removing.

Author response: We thank the reviewer for this comment. Supplementary Fig 4. is Supplementary Fig 5 in the revised draft. Just as a clarification, the purpose of this figure was to not demonstrate trends in the baseline prognostic factors with respect to dose. It was an attempt to illustrate how the patients enrolled at 24 mg/kg Q2W had slightly worse prognosis compared to the patients enrolled at other doses. The patients enrolled at 24 mg/kg Q2W had higher baseline ctDNA and LDH compared to the other doses, which could partially explain why the response observed at that dose was inferior. If the reviewer feels it is not necessary, we could remove the figure.

Line 888: Supplementary figure 5 caption notes that BAP1 mutation is associated with a worse response but the median is still a % decrease. Recommend re-wording assessment.

Author response: We thank the reviewer for this important clarification. In response, we have revised the Supplementary Figure 5 (Supplementary Fig 6 in the revised draft) caption to avoid implying that BAP1 mutation is associated with an absolute lack of response. The legend is re-worded to reflect a relative difference in best percentage change in nadir response, emphasizing that although median responses remain negative across groups, tumors harboring BAP1 mutations—particularly in rare combination with SF3B1 mutations—exhibit less pronounced tumor shrinkage compared with BAP1/SF3B1 wild-type tumors. This revised wording more accurately reflects the distributions shown in the box plots and avoids over-interpretation of the median values.

The revised Supplementary Figure 5 (Supplementary Fig 6 in the revised draft) and corresponding legend have been included in response to the comment #7 from reviewer # 1

Additional changes to the revised manuscript

- A data discrepancy between the text in the Results (Efficacy) subsection and Supplementary Table 2 has been identified and corrected. The revised text now consistently reports that confirmed objective responses were observed in 1~~2~~3/66 patients (19.7~~18.2~~%) with ~~1~~2/12 (16.7%) at 8 mg/kg Q2W. The data is corrected in Abstract as well 1~~2~~3/66 patients (19.7~~18.2~~%)
- Minor formatting corrections have been made throughout the manuscript (highlighted in the revised version).
- As new references, figures, and tables were added, the numbering of references, figures, and tables has been updated accordingly.

Response to referees

Reviewers' Comments:

Reviewer #1 (Remarks to the Author):

The responses to my queries are satisfactory and revisions appropriate.

Reviewer #2 (Remarks to the Author):

I am generally satisfied with the responses to the revisions. My comments are

1. Approximately 85%–90% of UMs harbor mutually exclusive activating mutations in either GNAQ or GNA11, most commonly affecting the Q209 (~80%) or R183 hotspots." - Again, in which gene is the Q209 and R183 hotspots? Do the authors need to add the word "respectively"

Author response: We thank the reviewer for this valuable comment. The residues Q209 and R183 are conserved hotspots present in both GNAQ and GNA11; therefore, mutations at these positions can occur in either gene. To improve clarity, we have revised the text as follows:

Revised text (*Green font*):

Approximately 85%–90% of UMs harbor mutually exclusive activating mutations in either GNAQ or GNA11, most commonly affecting conserved hotspot residues Q209 *and, less frequently, R183. These mutations result in constitutive activation of the GTPase domain, driving oncogenic signaling early in tumor development. These residues are shared across both proteins and may be affected in either gene (14-15).*

2. Figure 3a/b - please separate out Darovasertib ONLY or Darovasertib AND Crizotinib pre-treatments. The responses for the doublet shoudl be explicitly called out

Author response: We thank the reviewer for this valuable suggestion. In response, we have revised Figures 3a and 3b to clearly distinguish between pre-treatment groups. Specifically, patients who received darovasertib only are denoted as D, whereas those who received darovasertib in combination with crizotinib are denoted as D* and or B*

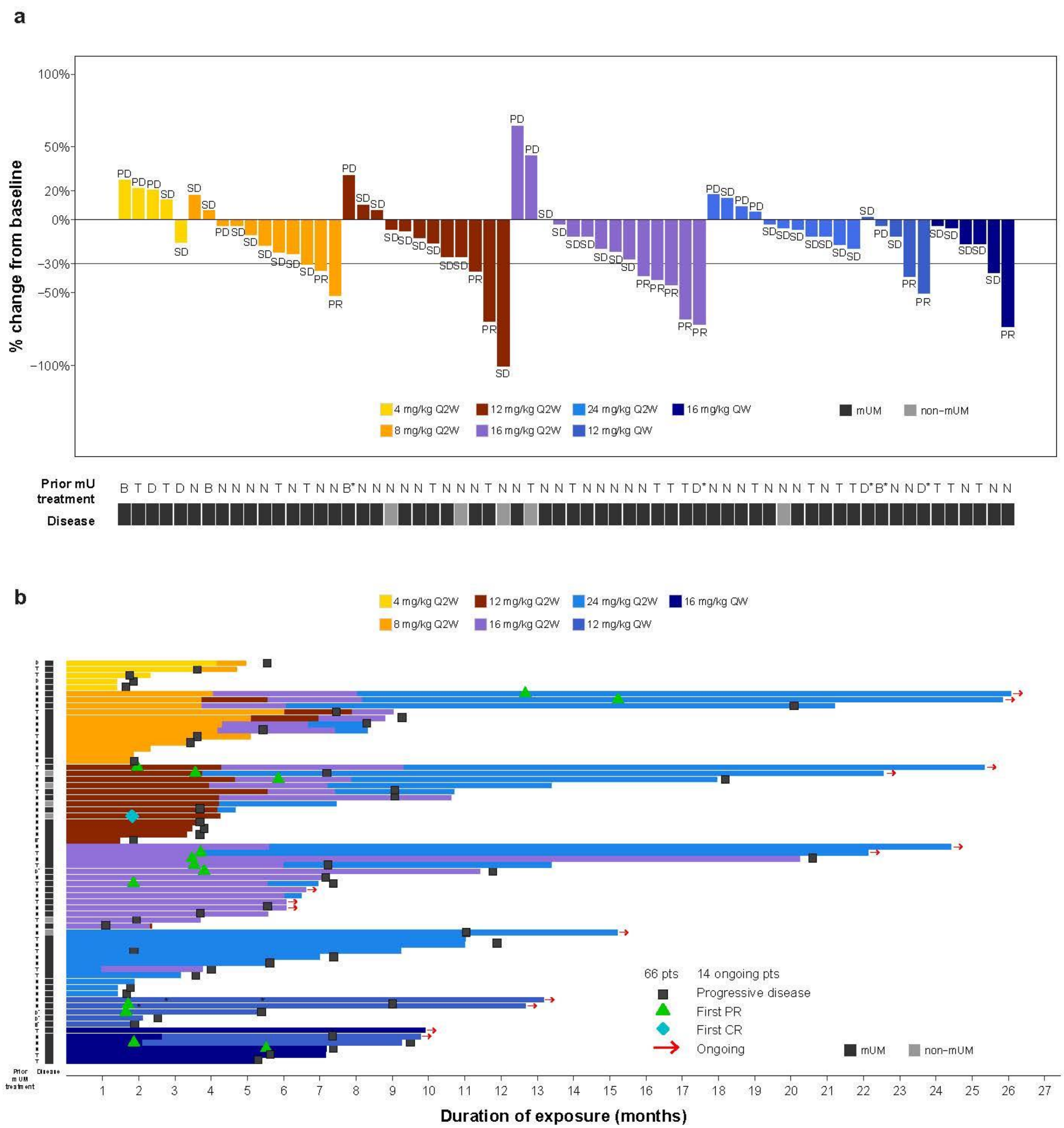

Fig. 3 ***Tumor Response and Duration of Exposure.*** (**a**) Percentage change from baseline in sum of the longest diameters by radiology review. The percentage of tumor size reduction is illustrated on the *y* axis. Every bar is one patient. The horizontal line represents at least a 30% decrease in the sum of the diameters of target lesions in patients. 1 patient was non-evaluable at the only post baseline scan as one of the target lesions could not be measured and 1 patient had non-measurable disease at baseline.(**b**) Duration of treatment. The x-axis denotes time in months, while each horizontal bar on the y-axis represents a single patient. *B, darovasertib and tebentafusp prior treatment; B*, darovasertib plus*

crizotinib and tebentafusp prior treatment; D, darovasertib prior treatment; D, darovasertib plus crizotinib prior treatment;* N, no prior darovasertib/crizotinib or tebentafusp treatment; *and tebentafusp prior treatment;* T, tebentafusp prior treatment; mUM, metastatic uveal melanoma; PD, progressive disease; PR, partial response; SD, stable disease; pts, patients; QW, once weekly; Q2W, every 2 weeks.

3. The authors need to update the manuscript with the recent press release from Ideaya about the efficacy of Darovasertib and Crizotinib in the discussion

Author response: We thank the reviewer for this suggestion. We have updated the discussion section and cited the OptimUM-02 trial abstract presented at ASCO 2026.

Revised text in Discussion (*Green font*): *Consistent with this, recent clinical data from darovasertib plus crizotinib has demonstrated a significant improvement in efficacy compared to investigator's choice, supporting the rationale for targeting both PKC - dependent signaling and MET-mediated pathways (39, 40).*

Reference added:

40. Orloff, M.M. et al. Darovasertib plus crizotinib vs investigator's choice as first-line treatment for patients with HLA-A2–negative metastatic uveal melanoma: primary results from the OptimUM-02 trial. J. Clin. Oncol. 44, suppl 17, LBA9503 (2026). https://doi.org/10.1200/JCO.2026.44.17_suppl.LBA9503

4. the graphic is great!

Reviewer #3 (Remarks to the Author):

Supplementary Table 3 lists 1 patient with non-measurable disease, however, for Figure 3a describes 2 patients that were non-evaluable. It seems an additional patient should be included as non-measurable at baseline.

Author response: We thank the reviewer for highlighting this point. We would like to clarify that Figure 3a corresponds to the data presented in Extended data Table 2 (Best overall response summary for all patients), which includes two patients: one with non-measurable disease at baseline and one who was classified as non-evaluable. In contrast, Supplementary Table 4 presents best overall response in the UM patients and includes only one patient who was non-evaluable.

Supplementary Table 5 does not add substantively to the manuscript but will defer to the authors if it is felt to contribute.

Author response: We thank the reviewer for this helpful suggestion. We believe the reviewer is referring to Extended Data Figure 5. We would prefer to retain this figure, as it provides important context by demonstrating that patients enrolled in the 24 mg/kg Q2W cohort had a slightly poorer baseline prognosis compared with those in other dose groups. Specifically, these patients exhibited higher baseline ctDNA and LDH levels, which may partially account for the comparatively lower response observed at this dose. However, if the reviewer considers this figure non-essential, we would be willing to remove it accordingly.
